# Moisture modulates soil reservoirs of active DNA and RNA viruses

**DOI:** 10.1038/s42003-021-02514-2

**Published:** 2021-08-26

**Authors:** Ruonan Wu, Michelle R. Davison, Yuqian Gao, Carrie D. Nicora, Jason E. Mcdermott, Kristin E. Burnum-Johnson, Kirsten S. Hofmockel, Janet K. Jansson

**Affiliations:** 1 Earth and Biological Sciences Directorate, Pacific Northwest National Laboratory, Richland, WA USA; 2 Department of Agronomy, Iowa State University, Ames, IA USA

**Keywords:** Ecology, Microbiology

## Abstract

Soil is known to harbor viruses, but the majority are uncharacterized and their responses to environmental changes are unknown. Here, we used a multi-omics approach (metagenomics, metatranscriptomics and metaproteomics) to detect active DNA viruses and RNA viruses in a native prairie soil and to determine their responses to extremes in soil moisture. The majority of transcribed DNA viruses were bacteriophage, but some were assigned to eukaryotic hosts, mainly insects. We also demonstrated that higher soil moisture increased transcription of a subset of DNA viruses. Metaproteome data validated that the specific viral transcripts were translated into proteins, including chaperonins known to be essential for virion replication and assembly. The soil viral chaperonins were phylogenetically distinct from previously described marine viral chaperonins. The soil also had a high abundance of RNA viruses, with highest representation of *Reoviridae*. *Leviviridae* were the most diverse RNA viruses in the samples, with higher amounts in wet soil. This study demonstrates that extreme shifts in soil moisture have dramatic impacts on the composition, activity and potential functions of both DNA and RNA soil viruses.

## Abstract (web summary)

Wu et al. use a multi-omics approach (metagenomics, metatranscriptomics, and metaproteomics) to characterize soil viral responses to soil moisture. The results indicate that extreme shifts in soil moisture have dramatic impacts on the composition, activity and potential functions of both DNA and RNA soil viruses.

## Introduction

Viruses are ubiquitous and abundant in nature^1^. However, they are underexplored in soil environments due to the experimental and computational difficulties in extracting and decoding their genetic complexity^2–6^. With increasing access to large sequence datasets, i.e., metagenomes and metatranscriptomes, it has become possible to perform more high-throughput and detailed surveys of viruses in soil environments^2,4^.

Metagenomes, while valuable for assembling DNA viral genomes, do not reveal whether the viruses detected are actively contributing to ecosystem functions at the time of collection. By contrast, study of viral community expression through metatranscriptomics and metaproteomics has the potential to reveal which viruses are active and what genes are expressed under given environmental conditions. In addition, metatranscriptome data can also be used to reassemble RNA viruses. Our knowledge of soil RNA viruses is currently even more limited than for DNA viruses, due to greater challenges in extraction of viral RNA from natural environments^7^. However, in the time of COVID-19, the knowledge of environmental RNA viruses is of utmost importance, given that soil and other natural ecosystems could function as environmental reservoirs for potentially pathogenic RNA viruses. To date, there has been only one survey of metatranscriptomes for RNA viruses in soil microcosms^3^, but their types and abundances in natural field sites and in other soil types are yet to be investigated. Metaproteomics provides an additional validation of viral activity, including which viral proteins are produced and evidence of lytic viral lifestyles. Metaproteomics is, however, challenging to perform in soil and there have been few successful examples to date^8–10^. However, improving techniques and new methodologies in proteomics have recently made it possible to delve deeper into soil proteomes for the detection of less-abundant proteins^11,12^. Therefore, here we used a multi-omics approach, combining DNA and RNA sequencing with metaproteomics, leveraging the strength of each omics method, to better understand how soil viruses respond to environmental changes.

In this study, we aimed to address how differences in soil moisture influence the activities of soil DNA and RNA viruses. We focused our study on a native prairie soil at the Konza Experimental Station in Kansas, which sits at the crossroads for predicted shifts in precipitation with climate change, either increasing drought towards the southwest, or increasing rainfall to the northeast^13^. To investigate both possible scenarios, we hypothesized that wet and dry soils would harbor different soil viruses and that their respective activities would be influenced by soil moisture. To address this hypothesis, we leveraged existing data from a simple experiment that compared Konza prairie soil that was saturated to soil that was desiccated^8,13–15^. Briefly, three replicate soil samples from two independent field locations in the Konza prairie were either wet to saturation or air-dried to represent wet and dry soil treatments, respectively^13^, resulting in a total of 12 soil microcosms. After a 15-day incubation at 21 °C under wet or dry conditions, DNA, RNA, and protein were extracted from each of the soil microcosms and the expression data (metatranscriptome and metaproteome) were mapped to metagenomes that were constructed from the original soil prior to incubation. The DNA was used for 16S rRNA gene sequencing as previously described^13^. The resultant multi-omics data were then used to screen for soil viruses and to assess their activities in response to differences in soil moisture.

## Results and discussion

### A diverse and active DNA virosphere

We first leveraged two existing metagenomes that were constructed from the Konza native prairie soil^14,15^ to screen for viral sequences at the site. Each of the metagenomes was obtained from a composite of all the replicate soils collected at ambient field moisture conditions. One of the metagenomes was de novo assembled from deep sequence data (1.1 Tb)^14^ and the second was a hybrid assembly of short and long reads (267.0 Gb)^16^. The combination of the two metagenomes was used to maximize the coverage of viral sequences from the Konza prairie site. To balance between the detection limits of the viral detection tools and the wide range of viral genome size, the viral contigs > 2.5 kb in length were combined with those obtained from screening of the two largest public viral databases (i.e., IMG/VR^17^ and NCBI Virus^16^) to further increase the coverage of DNA viral sequences. We acknowledge that the length cutoff of 2.5 kb would preclude detection of some ssDNA viruses with small segmented genome sizes (e.g., *Nanoviridae*^18^). As a result, a DNA viral database for the site was curated that included 726,108 de-replicated viral contigs. The DNA viral database then served as a scaffold for mapping of metatranscriptome and metaproteome datasets to determine the activities of soil DNA viruses and their responses to differences in soil moisture. This approach was also recently applied to detect the transcriptional activity of marine prokaryotic and eukaryotic viruses^19–22^ and giant viruses in soil^5^.

The metatranscriptome reads from both wet and dry treatments were mapped to a total of 416 unique DNA viral contigs using stringent criteria (% sequence identity > 95% and % sequence coverage > 80%). The 416 DNA viral contigs with an average sequence length of 19 kb were highly diverse and grouped into 139 clusters, with 111 of the clusters being singletons (Supplementary Data 1).

**Fig. 1:**
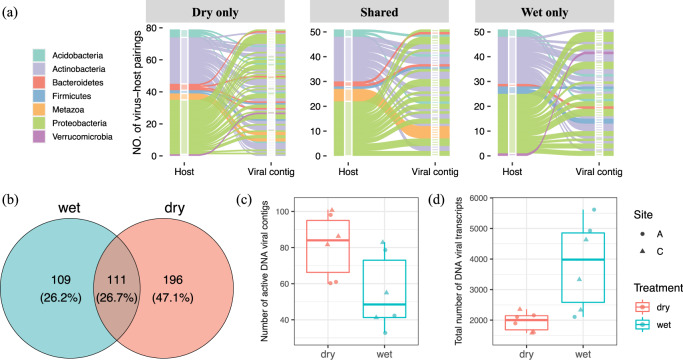
Transcribed DNA viral communities and their responses to wet and dry soil conditions. **a** An alluvium plot that illustrates pairings of the transcribed DNA viral contigs to putative host phyla. The transcribed DNA viral community was comprised of viral contigs from the curated DNA viral databases that were mapped by quality-filtered metatranscriptomic reads. The alluvia are colored by host taxa (first *x* axis of each sub-panel) assigned to respective transcribed DNA viral contigs (second *x* axis of each sub-panel). **b** A Venn diagram showing the number of unique transcribed DNA viral contigs detected in both wet and dry soils and ones exclusively detected in one of the soils. **c** Number of unique DNA viral contigs detected. A *t*-Test shows significantly more DNA contigs were transcribed in dry soil (*p* = 0.044). **d** Number of transcripts that mapped to the DNA viral contigs. For panels (**c**) and (**d**), the two independent field sites of Konza Experimental Field Station are indicated as site A (circles) and site C (triangles), with the wet soil in blue and dry soil in red.

We aimed to assign putative host taxa to the viral clusters by combining several approaches: CRISPR spacer matching, and screening for host and viral sequence similarities to respective databases (details in ‘Methods’). As a result, we assigned putative viral host taxa to 160 out of the 416 transcribed DNA viral contigs. Some of these were assigned to more than one host (Supplementary Data 1), resulting in a total of 181 virus–host pairings (Fig. 1a). Of these, 79 host–virus pairs were detected only in the dry soil treatment, 51 were only in the wet soil treatment, and an additional 51 were found in both dry and wet treatments (Fig. 1a). Consistent with previous reports^4^, the majority of the transcribed DNA viral contigs were annotated as bacteriophage sequences. Different sets of transcribed DNA viral contigs were unique to wet or dry soils and assigned to specific hosts at the phylum level, whereas others were shared (Fig. 1a). However, the dominant soil taxa, i.e., *Proteobacteria* and *Actinobacteria* that were previously identified by 16S rRNA gene sequencing in this soil environment, were predicted as hosts under both wet and dry conditions (Supplementary Fig. 1a). Eukaryotic DNA viruses, such as *Bracovirus* and *Ichnovirus* belonging to a family of insect viruses within the *Polydnaviridae* family, were also transcribed in the soils (Fig. 1a and Supplementary Data 1). Most of these insect viruses were only detected in dry soil conditions. These differences in virus–host pairings suggest that some of the respective hosts were impacted differently by the dry and wet incubation conditions.

There were 21 DNA viral contigs that were assigned to hosts across multiple bacterial phyla suggesting the presence of viral generalists^1,23^ (Supplementary Data 1). We recognize that host assignment based on CRISPR spacer matching, however, is limited to detection of recent or historical virus–host interactions that were captured at the time of sampling^24^. As bioinformatics assignment of virus–host linkages only suggests possible pairings based on sequence features, there are also chances of introducing false positives. However, we applied the most stringent criteria possible to provide confident host assignments.

### Increased activity of a subset of DNA viruses in wet soil

Soil moisture has a strong influence on the community structures of transcribed DNA viruses. The majority of the transcriptionally active DNA viral contigs were unique to wet or dry conditions, with only 111 viral contigs (~ 26.7%) detected in both wet and dry soils, suggesting that the different soil moisture conditions may shape the activity of the DNA viral community differently (Fig. 1b). Interestingly, although a significantly higher number of transcribed DNA viral contigs were detected in dry soils (Fig. 1b, c), the levels of transcriptional activity were significantly higher (based on the normalized abundance of RNA reads that mapped to the viral contigs) for DNA viruses in wet soils irrespective of sampling site location (Fig. 1d). DNA viral contigs with mapped transcripts could represent either prophages that are passively replicated along with their host genomes, or (lytic) viruses that are actively regulating early/middle/late expression of viral gene clusters^25^. In soil, a lysogenic lifestyle is considered to be an adaptive strategy for viruses to cope with long periods of low host activity^26,27^. Therefore, the 1.5-fold increase in the number of transcribed DNA viral contigs representing transcriptionally active DNA viruses, but with lower levels of overall transcription, in dry soil suggests that the increase was due to a higher prevalence of lysogeny in dry conditions. This hypothesis is strengthened by our finding of a 20-fold increase in transcripts for lysogenic markers (i.e., integrase and excisionase) in one of our replicates (A-2) in dry compared to wet conditions (Supplementary Data 2). High number of lysogenic phages were also previously reported in dry Antarctic soils using a cultivation-independent induction assay^28^. By contrast, under wet soil conditions we found a 2-fold increase in transcription of fewer viral contigs representing a subset of DNA viruses, suggesting that those viruses were more transcriptionally active in response to higher soil moisture. In addition, there was a higher correlation between prokaryotic abundances, as estimated by 16S rRNA gene sequencing, with DNA viral transcript counts in wet soils (*R*^2^ = 0.593, Supplementary Fig. 1d) in comparison to dry soils (*R*^2^ = 0.069, Supplementary Fig. 1d), supporting this hypothesis.

**Fig. 2:**
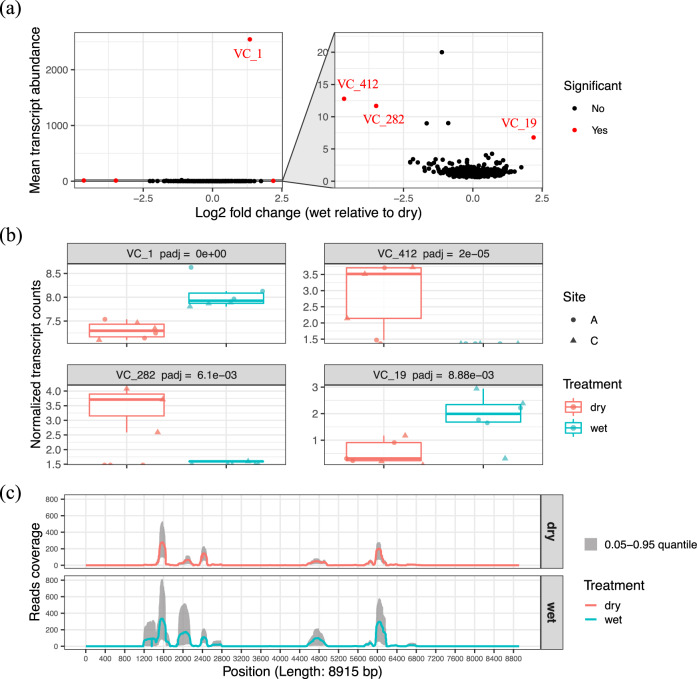
DNA viral contigs with differential transcription in wet and dry soil treatments. **a** Transcript abundance profiling of the identified DNA viral contigs. The mean transcript abundance of each DNA viral contig detected in all soils was plotted along the *y* axis in the sub-panel on the left. The normalized transcript abundances of each DNA viral contig were compared across treatments (wet and dry soils) and transformed into log_2_ fold change (wet relative to dry). The viral contigs with significantly differential transcript counts across treatments (*p* < 0.05) are highlighted in red in the sub-panel on the left. A zoomed-in panel on the right shows the viral contigs with lower transcript counts. **b** Four DNA viral contigs that were detected with differential transcript abundances in wet (blue) and dry (red) soils are shown. **c** Quality-filtered metatranscriptomic read coverage for the VC_1 sequence (total length of 8915 bp); the sequence with the highest number of transcripts mapped in (**a**). The solid line represents the mean read coverage per position detected in all replicates for each treatment (red = dry soil; blue = wet soil). The gray shading shows the range of read coverage distribution per position (0.05–0.95 quantile).

We then identified which soil DNA viruses were most transcriptionally active and how they responded to the differences in soil moisture. As the majority of the transcribed DNA viral contigs (97%) were environmental viruses with unclassified taxonomy assignment, we were not able to calculate the taxonomic abundance of each and instead compared the differential abundances of the transcribed viral contigs. There were four DNA viral contigs with significantly different levels of transcription under wet and dry conditions (VC_1, VC_19, VC_282, VC_412; Fig. 2a). Contigs VC_1 and VC_19 correspond to unclassified viral contigs deposited in IMG/VR (identifiers of ‘REF:2547132004_2547132004’ and ‘3300010038_Ga0126315_10000854’) that were previously detected in metagenomes from the Rifle site^29^ and from serpentine soil in the UC McLaughlin Reserve^30^, respectively. Contigs VC_282 and VC_412 were extracted from our Kansas metagenomes. Contigs VC_1 and VC_19 had significantly higher levels of transcriptional activity in wet soils compared to dry soils (*p* < 0.01, Fig. 2b), whereas VC_412 had significantly more transcripts in dry soils (*p* < 0.01, Fig. 2b). However, VC_282 was detected with higher transcript levels only in the dry soil replicates from site C. Five specific regions of contig VC_1 had the highest transcriptional activity in both wet and dry soil conditions, with lengths of 546, 338, 175, 420, and 663 bp, respectively (Fig. 2c). The finding that the same specific regions had differential transcript mapping frequencies is suggestive of active/lytic viruses with highly regulated transcription of early and/or late genes^25,31^, in comparison to lysogenic viruses that are passively transcribed along with their host genomes. VC_1 was originally detected from the Rifle site, an aquifer environment. Therefore, this virus–host pair may be better adapted to wet conditions, reflecting our finding of higher transcription levels for VC-1 in wet soil (*p* < 0.05, Fig. 2b). A similar increase in activity of DNA viruses together with a bloom of their respective hosts has also previously been observed following laboratory wetting of soil biocrusts^32^, suggesting that this may be a common phenomenon to soil wetting.

**Fig. 3:**
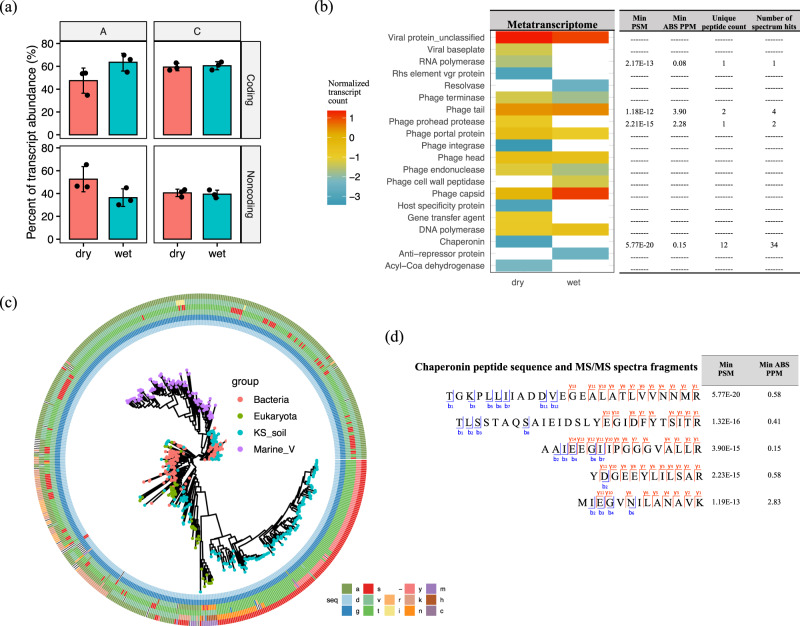
Functional characterization of viral transcripts and proteins. **a** The percentage of the quality-filtered transcripts that mapped to gene-coding and noncoding regions of DNA viral contigs. The percentage of noncoding transcripts trended towards higher, but not significant, levels in drier soils at site A. **b** Counts of viral structural/functional groups that were detected in both the metatranscriptomes (heatmap on the left) and metaproteomes (table on the right). **c** A phylogenetic tree based on the protein alignment of bacterial (red), eukaryotic (green), and soil (blue)/marine (purple) viral chaperonins (GroEL-like). The soil viral chaperonin protein sequences were translated from the predicted genes in transcribed DNA viral contigs. An example of a conserved region (position 1–6 of the trimmed multiple sequence alignments in Supplementary Data 6) is shown in a six-track ring outside the tree and the six tracks represent the six amino acids from that region in order from the inner to the outer rings. The corresponding amino acid of the conserved region in each chaperonin sequence is color-coded and specified in the figure key. **d** Examples of highly confident viral chaperonin peptide sequences with their observed fragmentation ions (blue for b-ions and red for y-ions) in MS/MS spectra, along with their minimal peptide-spectrum match (PSM) scores and minimal mass error of precursor ions (PPM) from MSGF+ search results.

It is interesting to note that the quality-filtered transcripts were mapped to both protein coding and noncoding regions of the DNA viral contigs. Noncoding RNAs with a phage origin have previously been reported to regulate viral replication at the early stage of infection and to maintain a lysogenic state by silencing the expression of late structural genes^33,34^. We observed a trend towards a higher percentage of viral noncoding RNAs in drier soils from site A, along with higher transcriptional levels of lysogenic markers (i.e., integrase and excisionase) in these samples (Fig. 3a and Supplementary Data 2). These findings suggest higher levels of lysogenic phages in these samples, but this hypothesis needs further experimental validation. Interestingly, recent studies identified prophage-encoded noncoding RNAs that can also contribute to the virulence of bacterial hosts^35^ and protective functions such as superinfection-immunity^36^. Future studies are therefore needed to better characterize the functions of viral noncoding RNAs.

After removal of noncoding RNAs, the remaining transcripts were mapped to 314 viral genes. Only 149 of those transcribed viral genes were annotated to functional gene categories (Supplementary Data 3), reflecting that a large proportion of viral genes remain uncharacterized. The annotated viral genes with transcripts detected are shown in Fig. 3b with the majority having low transcript read depths (< 20 per sample). A range of viral structural genes (e.g., phage tail, head, capsid), genes encoding DNA/RNA polymerase, and genes related to DNA recombination or re-arrangement (resolvase, Rhs element vgr protein) were transcribed, indicative of active viruses in the soil incubations. After verifying the gene positions on the respective viral contigs, one auxiliary metabolic gene (AMG), acyl-CoA dehydrogenase, was also found to be transcribed (Fig. 3b and Supplementary Data 3). This gene encodes an enzyme that is involved in initial steps of fatty acid metabolism. Similarly, virus-encoded AMGs involved in the fatty acid oxidation were previously reported in viromes from the Pacific Ocean^37^ and have been reported as a conserved strategy for a wide range of viruses in ocean^38^. Our findings suggest that viruses may similarly be involved in host metabolism in soil.

In addition to transcripts, we detected several viral peptides in our soil metaproteome data that were indicative of viruses with a lytic lifestyle. To our knowledge this is the first report of using an untargeted, metaproteomic approach to detect viral proteins in soil, although it has previously been used with success for aquatic samples^39^. For highly confident assignment of viral peptides with low abundances in the metaproteome, quality searching criteria (i.e., 5% false discovery rate (FDR), < 5 ppm mass error) were applied with manual inspection (Supplementary Data 4 and 6). Confident peptides for phage tail proteins, a virus-encoded protease, an RNA polymerase, and numerous chaperonins are captured in Fig. 3b. Inferring viral activity from metaproteomic data is supported by the fact that substantial components of viral structures (e.g., capsids and tails) are comprised of proteins^39^. Due to the low abundances of viral proteins detected, we refrained from statistical comparisons of the impact of soil wetting and drying on the proteome data.

Interestingly, we detected a collection of chaperonin-like genes (K01802, K03554, K04043, and K04077) that were expressed by soil viruses at both the transcript and peptide level. Examples of confidently identified viral chaperonin peptide sequences (PSM < 1.19E-13, PPM < 2.83) are shown in Fig. 3d. These soil viral chaperonins shared similar sequence regions with bacterial, eukaryotic, and marine viral GroELs (Group I chaperonin), and also contained conserved features that clearly distinguished them as a novel group of soil viral chaperonins, as shown in the alignments (Supplementary Data 6). One example of a conserved region is displayed as an outer circle of the GroEL phylogenetic tree (Fig. 3c). Chaperonins have previously been expressed from a phage^40^ and a plant virus (*Closteroviruses*)^41^. A recent study also reported a high prevalence of viral chaperonins including ones related to bacterial (GroELs) and archaeal (thermosome) chaperonins in an aquatic system^42^. Our phylogenetic analysis of bacterial, viral and eukaryotic GroEL genes (Fig. 3c) suggests that although chaperonins from the marine ecosystem are more phylogenetically similar to bacterial chaperonins, the majority of the soil viral chaperonins that we detected were most likely derived from eukaryotes, with some having bacterial origins. Such an apparent evolutionary separation of viral chaperonins in marine and soil ecosystems warrants further study. Because of the recognized functional importance of viral chaperonins in viral assembly and a lysogenic-lytic lifestyle transition^42^, the detection of soil viral GroEL at all levels, genomic, transcriptional, and translational, in our study suggests that the respective DNA viruses were actively infecting their hosts.

### A diverse soil RNA virosphere

Our second aim was to examine the soil metatranscriptome data for RNA viruses. To date there have been few reports of RNA viruses in soil. Recently, RNA viruses in a marine study were found to be more abundant than DNA viruses implying important ecological functions in marine ecosystems^7^. The current knowledge of RNA viruses in soil is fragmentary, mainly focusing on culturable viruses and crop pathogens^43,44^. Recently, Starr et al. (2019) reported detection of RNA viruses from metatranscriptomes of a California annual grassland soil^3^. Here we reassembled RNA viruses from the quality-filtered metatranscriptomic reads and uncovered a diverse RNA viral community in the Kansas native prairie soil.

**Fig. 4:**
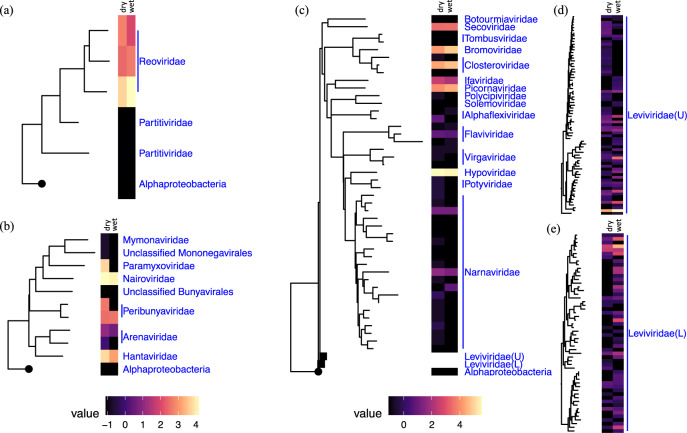
Phylogenetic diversity of RNA viruses and their estimated abundances. Phylogenetic placement and abundance estimates of the detected: **a** double-stranded RNA viruses; **b** negative single-stranded RNA viruses; and **c**–**e** positive single-stranded RNA viruses. Each of the RNA viral phylogenetic trees was constructed based on the aligned RNA-dependent RNA polymerase (RdRP) genes assigned to the identified RNA viral contigs and re-rooted by an RNA-dependent DNA polymerase (APO57079.1) of an Alphaproteobacterium (circular tree node). The abundance estimates for each taxon shown on the tree were measured by taking the average of the estimated average base-coverage per RNA viral contig mapping to this cluster of viruses detected under ‘dry’ or ‘wet’ conditions (left and right sections of each heatmap, respectively). The abundance estimates for each condition were then log transformed and illustrated in the heatmaps that are aligned to the respective tree tips. Two *Leviviridae* clades are collapsed in panel (**c**) for ease of visualization (tree nodes in rectangles); upper clade is noted as *Leviviridae*(U) and lower clade as *Leviviridae*(L). The phylogenetic structures and the abundance estimates for *Leviviridae*(U) and *Leviviridae*(L) are shown in panels (**d**) and (**e**), respectively.

The taxonomies of the identified soil RNA viral contigs are shown in rooted phylogenetic trees based on the alignments of the marker gene, RNA-dependent RNA polymerase (RdRP) for double-stranded RNA viruses, negative single-stranded RNA viruses, and positive single-stranded RNA viruses^45^ (Fig. 4). Most transcripts from double-stranded RNA viruses were mapped to viral contigs annotated as *Reoviridae*, which are known to infect a wide variety of eukaryotic hosts^46^ (Fig. 4a). One *Reoviridae* member that is closely related to a Bluetongue virus (MH559812, max. E-value of 1.86E-09, min. % identity of 88%) had the highest cumulative genomic coverage (Fig. 4a and Supplementary Data 7). This could be explained as either due to a bloom of the respective host during incubation, or due to their highly segmented genomes, i.e., ~10 dsRNA segments per genome, making them more resilient to sample processing steps^47^. This is in contrast to Starr et al. (2019) who did not detect *Reoviridae* in the California grassland study^3^. The negative single-stranded RNA viruses had highest representation of *Nairoviridae, Peribunyaviridae*, and *Hantaviridae* across both wet and dry soils and *Paramyxovirade* in dry soil only (Fig. 4b). The positive single-stranded RNA viruses had highest representation of *Secoviridae, Bromoviridae, Closteroviridae, Ifaviridae, Piconaviridae, Hypoviridae*, and *Leviviridae* (Fig. 4c). The *Picornavirales*-like RNA viruses detected in Kansas soil were not previously found in California soil^2^. More study is therefore needed to further extend our understanding of soil RNA viruses and their potential ecological functions.

More than half of the identified RNA viral sequences (124 out of 209) were assigned to *Leviviridae*, a family of negative-sense single-stranded RNA bacteriophages and the most diverse family found in Kansas soils (Fig. 4). The *Leviviridae*-like RNA viral sequences were clustered into two deeply branched major clades (Fig. 4d, e), supporting the recent hypothesis that the current *Leviviridae* family is evolving into two distinct lineages^45,48^. Similar to our study, Starr et al. (2019) also found *Leviviridae* phages were the most diverse in California grassland soil. *Leviviridae* are only known to infect *Proteobacteria*^49^ (Virus–Host DB, https://www.genome.jp/virushostdb/, accessed on 11 August 2020), one of the most abundant and diverse bacteria phyla in the Kansas soil (898 out of the total 4419 OTUs at 97% 16S rRNA gene sequence identity, Supplementary Fig. 1a). Similar observations of environmental viruses assigned to the most diverse and abundant taxa (e.g., *Proteobacteria*) have also been reported previously^1,2,4^.

### RNA viruses are responsive to soil moisture

**Fig. 5:**
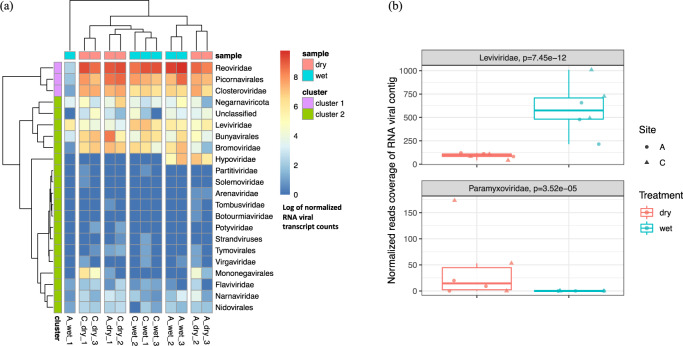
Composition profiling of the RNA viral community and its abundance shift in response to wet and dry soil treatments. **a** The RNA viral abundance of each phylogenetic group across all soil samples were summarized and clustered by composition similarity. **b** Two phylogenetic groups were detected with differential abundances in soils with different moistures (site A, circles; site C, triangles). Significantly more *Leviviridae* and significantly fewer *Paramyxoviridae* were detected in wet soils (blue) compared to dry (red) soils (*p* < 0.05).

Soil wetting and drying treatments impacted the RNA viral communities. Soil moisture treatments shaped the RNA viral communities from each of the Kansas soil locations (A and C) differently (Fig. 5a). The RNA viral communities generally grouped together according to sampling locations, except for one outlier from site A (Fig. 5a). The total RNA viral abundances were strongly correlated with the abundance of active eukaryotic species (based on 18S rRNA gene transcript counts from the metatranscriptomes, *R*^2^ = 0.829 in Supplementary Fig. 1e). The correlation was higher in soils under wet conditions (*R*^2^ = 0.856 in Supplementary Fig. 1f) compared to dry conditions (*R*^2^ = 0.811 in Supplementary Fig. 1f). Among the detected RNA viruses, there were two viral families that responded significantly to the experimental soil wetting and drying. The estimated abundances of *Leviviridae* were higher in wet soils (*p* < 0.01, Fig. 5b). By contrast, a family of eukaryotic viruses (*Paramyxoviridae*) was more abundant in dry soils (*p* < 0.01, Fig. 5b). Due to the predominantly lytic lifestyle ascribed to *Leviviridae*^3^, higher abundances in wet soil compared to dry soil may reflect a higher degree of host lysis in wet soils. *Proteobacteria*, the host of *Leviviridae*^50^, were also more abundant in wet soil (Supplementary Fig. 1a). Sampling across multiple time-points is needed to specifically investigate the continuous population dynamics of the virus–host communities, to better resolve interactions and transcriptional regulation at the community scale, and to elucidate infection dynamics.

## Conclusion

In summary, the highly diverse DNA and RNA viral composition, the increased viral activity in wet soil, and the potential virus–host interactions discovered here bring new knowledge about the ecological importance of the soil virosphere. As we demonstrate in this study, integration of information from multi-omics data is a promising approach for identifying both DNA and RNA viruses and assessing their potential activities in response to environmental changes. It has not escaped our notice during the global COVID-19 pandemic that the approaches described here can also be applied to detect novel pathogenic viral reservoirs in the environment. Because the soil we analyzed was a native prairie soil, with low levels of anthropogenic impacts, the majority of the active DNA viruses that we detected were bacteriophage or eukaryotic viruses known to have insect hosts. We found a high diversity of RNA viruses, including a group of relatively undefined eukaryotic viruses. Therefore, it would be interesting to compare this study to other soil environments, including those that are impacted by anthropogenic disturbances to further characterize the soil virosphere, and investigate their potential as reservoirs of viral pathogens.

## Methods

### Sampling site and soil treatment

Soil samples (top 0–15 cm) were collected in three locations at least 10 m apart from each of two sites (noted as sites A: 39°06'11ʺN, 96°36'48ʺW, 339 meters above sea level (MASL) and site C: 39°04'20ʺN, 96°34'33ʺW, 415 MASL in our previous study^13^) from native grassland locations at the Konza Experimental Field Station in Kansas. The soil samples from sites A and C were processed separately. For each site, the three soil samples were combined, homogenized, frozen and shipped in 1 gal Ziploc bags on dry ice to PNNL. Upon receipt at the laboratory, the samples were quickly processed through a 2 mm sieve. The soils from the two field locations (sites A and C) were separately aliquoted into ~ 50 g portions in Falcon tubes, flash frozen in liquid nitrogen and stored at −80 °C until use.

DNA was extracted from the original composite soil that was collected from the Konza field site for construction of two metagenomes as previously described^13^. One of the metagenomes was constructed for deep sequence coverage, with 1.1 Tb of sequence data^14^. The second metagenome used a hybrid assembly approach with long reads and short reads for improved assembly^15^.

Soil microcosms were prepared for each site (A and C) in three replicates and were either air-dried (dry treatment) or wet to saturation (wet treatment) and incubated at 21 °C for 15 days, as previously described^13^. DNA, RNA, and protein were extracted from each of the 12 microcosms: 2 field locations × 2 treatments × 3 replicates. The DNA was used for 16S rRNA gene sequencing as previously described^13^. RNA was extracted from 2 g of soil from each microcosm using the PowerSoil RNA extraction kit (MoBio Laboratories Inc., Carlsbad, CA, USA), according to the manufacturers’ instructions. Protein was extracted using the MPLex approach, as previously described^11^.

### Mining viral sequences from metagenome assemblies

DNA viral sequences (> 2500 bp) were mined from assembled contigs of the two existing metagenomes^14,15^ described above. The de novo assembled contigs were screened for viral sequences by three independent methods including: (1) a probabilistic approach (taxonomy assignment based on multiple open reading frames, modified from Paez-Espino et al. (2017)); (2) public database searches (VirSorter^51^ and IMG/VR^17^); and (3) machine learning (VirFinder^52^). The three methods were integrated and viral contigs were specified using four criteria: (1) contigs had more genes with viral origins than ‘bacterial or archaeal-like’ origins; (2) contigs were classified as viral contigs by VirSorter; (3) contigs had E-value cutoffs ≤1.0e^−05^ after searching the IMG/VR database; and (4) contigs had *p*-values less than 0.05 and a VirFinder score greater than 0.90. Contigs that fulfilled at least three out of these four criteria were classified as viral contigs. Details of the different methods are provided below:

1. Probabilistic method
  Protein sequences of the open reading frames were predicted and translated using Prodigal^53^ and searched against viral, bacterial, and archaeal genome databases using *hmmsearch* (Hmmer v3.1b2^54^, E-value cutoff of 1.0e^−05^). The five databases searched included three viral databases: (1) a collection of 25,218 viral protein Hidden Markov Models (HMMs) built upon viral protein-coding genes from NCBI viral genomes^55^; (2) a custom database comprised of 1147 curated viral protein families (Pfam^56^, Supplementary Data 8); and (3) the existing Nucleo-Cytoplasmic Viruses Orthologous Groups (NCVOG)^57^. In addition, the EggNOG bacterial and archaeal database^58^ were used to annotate the proteins that are more likely from bacterial or archaeal genomes. The bit scores from searching against the five databases per protein were ranked and taxon annotation was assigned based on the search with the highest bit score. Modified from the previous study with arbitrary cutoffs^55^, we retained the contigs with more ‘viral-like’ than ‘bacterial or archaeal-like’ genes on check and considered them as likely viral candidates, thus fulfilling our first criterion.
2. Public database searching
  Similar to the ‘Probabilistic method’, we also screened for viral contigs using VirSorter^51^, one of the most widely used viral classification tools. VirSorter searches against profile HMMs and protein sets curated from public databases of NCBI RefSeqVirus (v69, January 2014) and virome sequences from selected habitats, which complement the databases searched in the ‘Probabilistic method’. The contigs classified as Category 1–3 by the VirSorter scoring matrix were retained as filling our second criterion above.
  The contigs were also searched against IMG/VR (released on 1 July 2018), the largest publicly available database that includes both cultured and uncultured viral sequences^17^. Contigs having E-value cutoffs ≤1.0e^−05^ fulfilled our third criterion and were retained.
3. Machine learning method
  To access as many viral features as possible, contigs were also screened by VirFinder^52^, a machine learning-based method of modeling viral tetranucleotide patterns. Contigs with *p*-values less than 0.05 and a VirFinder score greater than 0.90 passed our fourth criterion.

After examination by the four criteria, the length-filtered assemblies (> 2500 bp) satisfying at least three out of the four criteria were identified as the viral contigs used for the following analyses.

### DNA viral database curation

To expand the DNA viral reference dataset for transcript searching, the resulting viral contigs identified from the two metagenomes constructed from the source soil as described above, were combined with the two largest public viral databases, IMG/VR (released on 1 July 2018) and NCBI Virus^16^. The combined DNA viral sequences were then de-replicated to remove the exact and sub-strings of the sequences (VSEARCH v2.13.4, derep_fulllength) and curated into a larger DNA viral database including 726,108 unique DNA viral sequences. The newly curated DNA viral database was searched against the metatranscriptomes sequenced from wet and dry treatments for a comparison of active DNA viral communities.

### Quality control of metatranscriptomic data

The existing metatranscriptomic data^13^ were screened both to determine which DNA viruses were transcriptionally active and to determine the RNA virosphere in wet and dry soil treatments. As described above, the metatranscriptomes were obtained from three replicate samples for each of the two independent locations after the wet and dry soil incubations, resulting in a total of 12 metatranscriptomes. The raw cDNA sequence reads were trimmed to remove the Illumina adapters and to retain high-quality reads (score > 30 and length > 36 bases), as recommended by Trimmomatic (v0.33). The metatranscriptomic reads were mapped to PhiX genome using Burrows-Wheeler Aligner (BWA, v0.7.17) and the exact matches were removed. The quality-filtered reads were then separated into reads corresponding to ribosomal and non-ribosomal RNA using SortMeRNA (version 2.1b). The ribosomal RNA reads were then filtered for 18S rRNA transcripts by mapping to a SILVA database including a set of well-curated 18S rRNA reference genes (SortMeRNA version 2.1b, silva-euk-18s-id95 database) to determine the transcriptionally active eukaryotic community composition (more details in Supplementary Fig. 1b). The remaining reads, corresponding to non-ribosomal RNA, were used to determine which DNA viruses were transcriptionally active and also to assemble RNA viruses.

### Transcriptional activity and diversity of DNA viruses

The non-ribosomal RNA reads were mapped to our self-curated DNA viral database using BamM (v1.7.3, bamm make, https://github.com/Ecogenomics/BamM) and the mapping was filtered using stringent cutoffs, i.e., % identity > 0.95 and % alignment > 0.80 (BamM v1.7.3, bamm filter). DNA viral sequences that were mapped by the quality-filtered metatranscriptomic reads were considered as transcribed DNA viruses. Transcriptional activity was estimated by the average base coverage of the viral sequences (samtools v1.9, samtools depth, http://www.htslib.org/doc/) normalized by the total counts of reads per sample and the length of the searched DNA viral contigs.

In addition, transcripts of lysogenic markers, integrase and excisionase, were recruited from the metatranscriptomes. A total of 20,712 unique viral integrases and 329 unique excisionases were collected from NCBI Virus (https://www.ncbi.nlm.nih.gov/labs/virus/vssi/#/, accessed on 16 December 2020; sequence de-replication using VSEARCH v2.13.4, derep_fulllength). The same transcript read mapping strategies and quality filtering criteria as mentioned above were applied. The transcript counts of the lysogenic markers were normalized by the total number of reads per sample.

The diversity of the transcriptionally active DNA viruses was evaluated by clustering the viral sequences based on their average amino acid identities (AAIs) using the Ruby script in enveomics toolbox^59^ and converting the output into a distance matrix. The single linkage clustering in scikit-learn (v0.23.1) was applied to the matrix to cluster the viral sequences at the cutoff of 70% AAI. Because the viral genome assemblies from the soil metagenomes were incomplete, fragments from different regions of the same viral genome were not clustered together based on AAI, when no significant amounts of proteins/genes were shared. We also incorporated a complementary method for clustering the viral contigs according to their tetranucleotide patterns. The tetranucleotide frequency-based method was previously shown to make linkages between nonoverlapping but related viral contigs^60^ and is the main principle underlying bioinformatics tools like VirFinder^52^ and PhaMers^61^. Pair-wise Pearson correlations were calculated between *z*-score distributions for every tetranucleotide in the screened viral contigs (pyani v0.2.9, https://github.com/widdowquinn/pyani). The viral contigs that were not clustered by the AAI method were assigned to the clusters containing the paired contigs with the highest correlation coefficients of the tetranucleotide frequency *z*-scores.

### Transcription of coding and noncoding regions of DNA viruses

The quality-filtered transcripts that mapped to the DNA viral contigs as mentioned above (% identity > 0.95 and % alignment > 0.80) were further separated into those that mapped to gene-coding and noncoding regions of the DNA viral contigs (predicted by Prodigal, v2.6.3) and counted individually. Potential AMGs were screened from the transcribed genes of the DNA viral contigs. The most confident AMG candidates were recognized as those that were annotated with auxiliary metabolic functions (E-value cutoff of 1 × 10^−5^) and located on viral contigs with ‘viral-like’ genes both up and downstream.

### Assembly of RNA viruses from metatranscriptome data

The quality-filtered metatranscriptomic reads were also de novo assembled by Trinity (v2.8.5). RNA viruses were screened from the de novo assemblies in two ways: (1) searching for the marker gene, RdRP, using a set of curated HMMs, and (2) the whole-sequence similarity searches against NCBI RNA viral genomes^16^ (E-value cutoff of 1 × 10^−5^). Taxonomy annotation of the identified RNA viral contigs was based on RdRP gene similarity, which has been used as a phylogenetic marker for RNA viruses^3^. Due to the incomplete assembly of the viral genomes, some identified RNA viral contigs were not detected by RdRP gene-screening using HMMs, but were instead annotated by their significant hits in NCBI RNA viral database (E-value cutoff of 1 × 10^−5^). Multiple Alignment with the Fast Fourier Transform (MAFFT v7) program was used to align the RdRP genes and a phylogenetic tree was constructed using the maximum-likelihood method via FastTree (v2.1.10) to display the RNA virosphere. The quality-filtered metatranscriptomic reads were mapped to the detected RNA viral assemblies and filtered with stringent criteria (95% identity, 80% coverage) by BamM (v1.7.3, bamm make and bamm filter, https://github.com/Ecogenomics/BamM). The abundances of the RNA contigs were estimated by the average base coverage of the mined RNA viral sequences after filtering the mapping at % identity > 0.95 and % alignment > 0.80 (samtools v1.9, samtools depth, http://www.htslib.org/doc/). The relative abundance of each taxon was measured by taking the average of the estimated coverage of RNA viral contigs assigned to the taxon. The genome coverage of each RNA viral taxon was used to represent the composition of the detected RNA viral community under each treatment.

### Mining viral peptides from MS/MS spectra

Metaproteomes were obtained from the same soil samples as for the metatranscriptomes, resulting in 12 matched metaproteomes (three replicates each for wet and dry incubations and two locations). The LC-MS/MS were performed using two different approaches as described previously^8^. Briefly, all of the samples were analyzed individually with one-dimensional LC-MS/MS. In addition, the pooled replicate samples went through off-line liquid-chromatography-based fractionation followed by LC-MS/MS analysis^8^.

We first curated a viral protein database including the proteins predicted from contigs in DNA viral database mentioned above (Prodigal, v2.6.3), plus a set of unique reference viral structural proteins collected from NCBI Virus (a total of 63,088 capsid proteins, 25 envelope and 15,817 tail proteins). The LC-MS/MS spectra were searched against the metaproteome database using MS-GF+ search engine^62^. The spectrum level peptide confidence score of the peptide-spectrum match (PSM; i.e., MSGFDB_SpecProb in MS-GF+) and mass error (in ppm) of the precursor peptide ion (i.e., DelM_PPM in MS-GF+) were optimized to achieve the highest number of peptide identification within each dataset while keeping the target-decoy-based FDR of peptide identification below 5%. In order to obtain the most confident viral identification, any redundant peptide identifications between the viral metaproteome and the rest of the metaproteome were excluded. The remaining peptide identification were from the viral metaproteome only. The data also went through a manual quality control process to remove any PSM with low confidence based on MS/MS fragmentation coverage of the peptide and MS peak quality. Final spectra are shown in Supplementary Data 5, and their qualification are shown in Supplementary Data 4.

### Host prediction of identified DNA viruses

An integrated approach was applied to assign host taxa to the DNA viral contigs. DNA viral contigs were first searched for exact matches in the spacers detected from the clustered regulatory interspaced short palindromic repeat (CRISPR) regions on the non-viral contigs from the two metagenomes (Method 1). Spacers were detected using MinCED (https://github.com/ctSkennerton/minced) and searched against the viral contigs using optimized parameters as previously described^63,64^. The viral contigs were assigned to hosts based on the taxonomy annotations of the respective non-viral contigs extracted with the spacer hits using the Contig Annotation Tool (CAT, v4.6, https://github.com/dutilh/CAT). Sequence similarity searches to non-viral contigs (potential hosts) and NCBI reference viruses were then applied to the remaining viral contigs that were not assigned by the spacer hits. A non-viral contig was assigned to a putative viral host when they shared a high sequence similarity (Method 2, BLASTN, E-value threshold = 10^−3^ and bit score = 50). Alternatively, when a viral contig had a close hit in the NCBI viral genome database (BLASTN, E-value threshold = 10^−3^ and bit score = 50), we adopted the host information of the closest reference virus (Method 3). In addition, we trained homogeneous Markov models (Method 4) using 1853 NCBI RefSeq genomes and applied a probabilistic approach coded in WIsh^65^, known for accurate host predictions of short viral contigs. We only retained the virus–host pairings that were detected using our nested workflow (Methods 1-2-3) and WIsh^65^ (Method 4) for highly confident host assignments.

### Phylogenetic placement of soil viral chaperonin

First, 4821 bacterial and eukaryotic Group I chaperonin (GroEL) protein sequences were extracted from the NCBI Reference Sequences (https://www.ncbi.nlm.nih.gov/refseq/, accessed July 2020) and de-replicated using VSEARCH (v2.13.4). A total of 235 bacterial and eukaryotic GroEL protein sequences from unique Orders were selected to represent the phylogenetic diversity. An additional 108 viral chaperonins from an intensive study of marine viruses possessing a bacterial-derived GroEL^42^ were added. Protein sequences predicted from the Kansas viral contigs that were annotated as chaperonins and detected in either metatranscriptome or metaproteome were aligned together with the bacterial, eukaryotic and marine viral GroELs using MAFFT (v7). The alignment with the estimated sequence conservation was visualized in Jalview (v2.11.1.0). A phylogenetic tree of GroEL across kingdoms was constructed using maximum-likelihood method via FastTree (v2.1.10).

### Statistics and reproducibility

*t*-Tests were conducted to assess the significance of the different counts of viral sequences and transcripts mapped under wet and dry treatments. The DESeq2 package was used for differential gene expression analysis. Differential abundances with *p*-values that were less than 0.05 were considered as significant. Sample size and number of replicates are described in ‘Sampling site and soil treatment’ and provided in the results and figure legends. The experiment was run using replicate samples for each treatment and at least reproducible results were found when comparing between replicates.

### Reporting summary

Further information on research design is available in the Nature Research Reporting Summary linked to this article.

## Supplementary information

Supplementary information.

Description of Supplementary Files.

Supplementary Data 1. Summary of the 416 transcribed DNA viral contigs.

Supplementary Data 2. Transcript reads of lysogenic marker genes encoding viral integrase and excisionase.

Supplementary Data 3. Annotations and normalized transcript counts of DNA viral genes.

Supplementary Data 4. Viral peptides detected in metaproteomes.

Supplementary Data 5. Raw spectrum graphs of the 60 highly confident mass spectrometry spectral hits.

Supplementary Data 6. The alignments of bacterial, eukaryotic, marine and soil viral chaperonin protein sequences.

Supplementary Data 7 RNA viral contigs identified from the Kansas grassland soil.

Supplementary Data 8. Viral Pfam list for viral gene annotation.

Reporting summary.

## Data Availability

The two published metagenomes can be downloaded from the PNNL Data Hub (https://data.pnnl.gov/project/12620) and White et al.^15^. The metatranscriptome sequence data and the 16S rRNA sequence data are available in Chowdhury et al.^13^. The MS proteomics data have been deposited to the ProteomeXchange Consortium via the MASSIVE partner repository with the accession number of MSV000086144 (https://massive.ucsd.edu/ProteoSAFe/dataset.jsp?task=500afc8e54e1481a9d90fcd35b556511). The DNA and RNA viral sequences identified from de novo assemblies are deposited under NCBI BioProject accession number PRJNA744880. All of the curated databases, including the reference viral structural proteins, viral lysogenic gene markers and the curated DNA viral database, and source data that were used to plot the figures are packaged at the public repository Zenodo^66^.

## Code availability

The R codes used to plot the main and supplementary figures are available at Zenodo with no restriction to access^66^.
