# Supplementary material for: Moisture modulates soil reservoirs of active DNA and RNA viruses
Source: Commun Biol. 2021 Aug 26;4:992. doi: 10.1038/s42003-021-02514-2 (PMC8390657; doi:10.1038/s42003-021-02514-2)
Supplement: Supplementary file 1 — Supplementary information. [file 42003_2021_2514_MOESM1_ESM.pdf]

Supplementary information

Moisture Modulates Soil Reservoirs of Active DNA and RNA Viruses

Ruonan Wu[1], Michelle R. Davison[1], Yuqian Gao[1], Carrie D. Nicora[1], Jason E. Mcdermott[1], Kristin E. Burnum-Johnson[1], Kirsten S. Hofmockel[1, 2], Janet K. Jansson[1*]

1. Earth and Biological Sciences Directorate, Pacific Northwest National Laboratory, Richland, WA 99352, USA
2. Department of Agronomy, Iowa State University, Ames, IA 50010, USA

*Corresponding author

E-mail: janet.jansson@pnnl.gov

Supplementary Methods

Re-analyzing the existing 16S rRNA amplicon data

We analyzed existing 16S rRNA amplicon sequence data[1] to determine prokaryotic community compositions and their responses to the changes in soil moisture after incubation. The 16S rRNA amplicon sequences were processed using our in-house Hundo pipeline[2] (v1.2.8). In brief, the sequences were first quality filtered to remove the adaptors and contaminated reads from Phix genomes by BBDuk2[3]. The passing reads were merged and checked for chimeras, and clustered into OTUs by VSEARCH[4] using the default parameters. The lowest common ancestor (LCA) algorithm[5] was applied to the lineage information of the sequences in each OTU to conservatively assign host taxonomy. The abundance of each OTU was estimated by read coverage of the OTU representative sequences (VSEARCH).

Detection of potentially active eukaryotic species

After quality control of the metatranscriptomic data as mentioned in the main text, the remaining transcript reads were mapped to a well-curated reference database of 18S rRNA genes in the Silva Database (silva-euk-18s-id95) using BamM (v1.7.3, bamm make, https://github.com/Ecogenomics/BamM) and filtered by percent identity higher than 0.95 and percent alignment greater than 0.80 (BamM v1.7.3, bamm filter). The extracted 18S rRNA transcripts metatranscriptomic reads were used to inform the potentially active eukaryotic species. The abundances of the eukaryotes were estimated by the average base coverage of the mapped 18S rRNA reference sequences (samtools v1.9, samtools depth, http://www.htslib.org/doc/) normalized by the total counts of reads per sample.

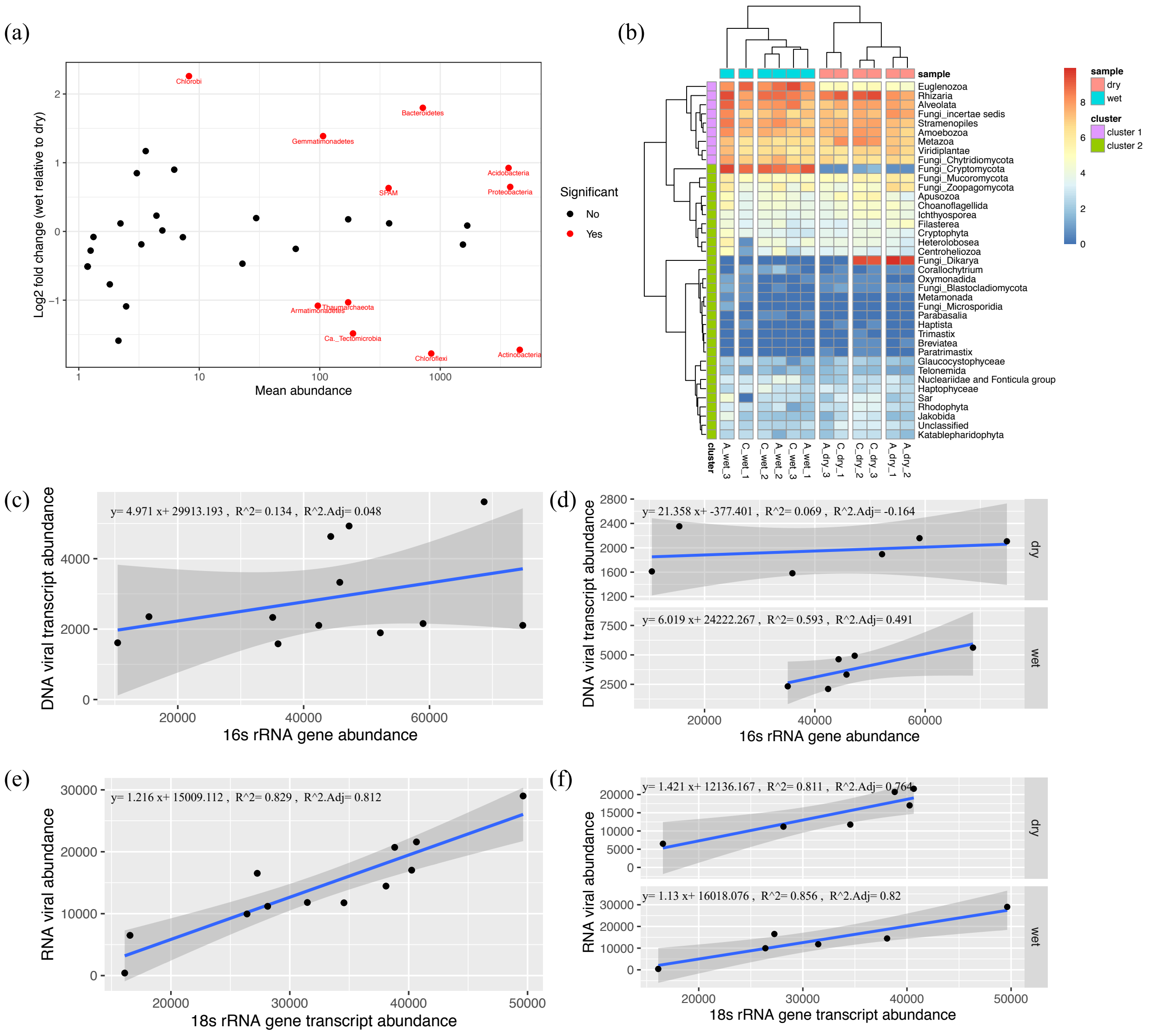

Supplementary figure 1. Methods and results of analyzing 16S rRNA gene amplicon sequence data and 18S rRNA transcriptomic reads.

The abundance shifts of the prokaryotic community and the compositional dynamics of the transcribed eukaryotic community in response to wet and dry treatments of Kansas grassland soils.

(a) The mean 16S rRNA gene abundances of each bacterial phylum detected in all soils were plotted along the x axis. The normalized abundances of each bacterial phylum were compared across treatments (wet and dry soils) and those that were statistically different ($p < 0.05$) are highlighted in red. The datapoints with positive y values represent taxa that were more abundant in wet soils and those with

negative y values were more abundant in dry soil. (b) The potentially active eukaryotic groups were detected based on 18S rRNA gene transcripts from the metatranscriptomes across all the samples. The community compositions of the transcribed eukaryotic species are shown in the heatmap with log transformed transcript coverages that are color-coded (the warmer the color, the higher the coverage). The transcribed eukaryotic communities detected from all of the samples are clustered by composition similarities. The correlation of 16S rRNA gene abundance (x-axis) with the transcript abundance of the DNA viral contigs (y-axis) is shown in (c). The same correlation per treatment was calculated and is shown in (d) with dry treatment on the top channel ($R^2 = 0.069$) and wet treatment on the bottom channel ($R^2 = 0.593$). (e) demonstrates the correlation between 18S rRNA gene transcript abundance and the abundance of RNA viral contigs ($R^2 = 0.829$). The correlation was also calculated separately per treatment as shown in (f) with a R^2 of 0.811 under dry conditions (top channel) and a R^2 of 0.856 under wet conditions (bottom channel). All of the regression equations and R^2 values are shown on the left top of each panel.

Supplementary References

1 Chowdhury, T. R. *et al.* Metaphenomic responses of a native prairie soil microbiome to moisture perturbations. *Msystems* **4** (2019).

2 Brown, J., Zavoshy, N., Brislawn, C. J. & McCue, L. A. Hundo: a Snakemake workflow for microbial community sequence data. Report No. 2167-9843, (PeerJ Preprints, 2018).

3 Bushnell, B. BBMap: a fast, accurate, splice-aware aligner. (Lawrence Berkeley National Lab.(LBNL), Berkeley, CA (United States), 2014).

4 Rognes, T., Flouri, T., Nichols, B., Quince, C. & Mahé, F. VSEARCH: a versatile open source tool for metagenomics. *PeerJ* **4**, e2584 (2016).

5 Aho, A. V., Hopcroft, J. E. & Ullman, J. D. On finding lowest common ancestors in trees. *SIAM Journal on computing* **5**, 115-132 (1976).
