# Supplementary material for: Moisture modulates soil reservoirs of active DNA and RNA viruses
Source: Commun Biol. 2021 Aug 26;4:992. doi: 10.1038/s42003-021-02514-2 (PMC8390657; doi:10.1038/s42003-021-02514-2)
Supplement: Supplementary file 2 — Description of Supplementary Files. [file 42003_2021_2514_MOESM2_ESM.pdf]

Description of Additional Supplementary files

File name: Supplementary Data 1

Description: *Summary of the 416 transcribed DNA viral contigs.* A full list of the 416 transcribed DNA viral contigs including information of contig lengths ('Length'), method ('Clustering_method') and result ('Singleton', 'Cluster_index') of clustering and host assignments.

File name: Supplementary Data 2

Description: Transcript reads of lysogenic marker genes encoding viral integrase and excisionase.

File name: Supplementary Data 3

Description: *Annotations and normalized transcript counts of DNA viral genes.* A full list of annotations for all of the 314 transcribed DNA viral genes with normalized transcript counts.

File name: Supplementary Data 4

Description: *Viral peptides detected in metaproteomes.* A total of 60 highly confident mass spectrometry spectral hits containing 27 unique viral peptides with minimum peptide-spectrum match scores of 5.77E-20 and minimal absolute mass error of precursor ions of 0.08.

File name: Supplementary Data 5

Description: Raw spectrum graphs of the 60 highly confident mass spectrometry spectral hits.

File name: Supplementary Data 6

Description: The alignments of bacterial, eukaryotic, marine and soil viral chaperonin protein sequences.

File name: Supplementary Data 7

Description: RNA viral contigs identified from the Kansas grassland soil.

File name: Supplementary Data 8

Description: Viral Pfam list for viral gene annotation.
