# Supplementary material for: Moisture modulates soil reservoirs of active DNA and RNA viruses
Source: Commun Biol. 2021 Aug 26;4:992. doi: 10.1038/s42003-021-02514-2 (PMC8390657; doi:10.1038/s42003-021-02514-2)
Supplement: Supplementary file 7 — Supplementary Data 5. Raw spectrum graphs of the 60 highly confident mass spectrometry spectral hits. [file 42003_2021_2514_MOESM7_ESM.pdf]

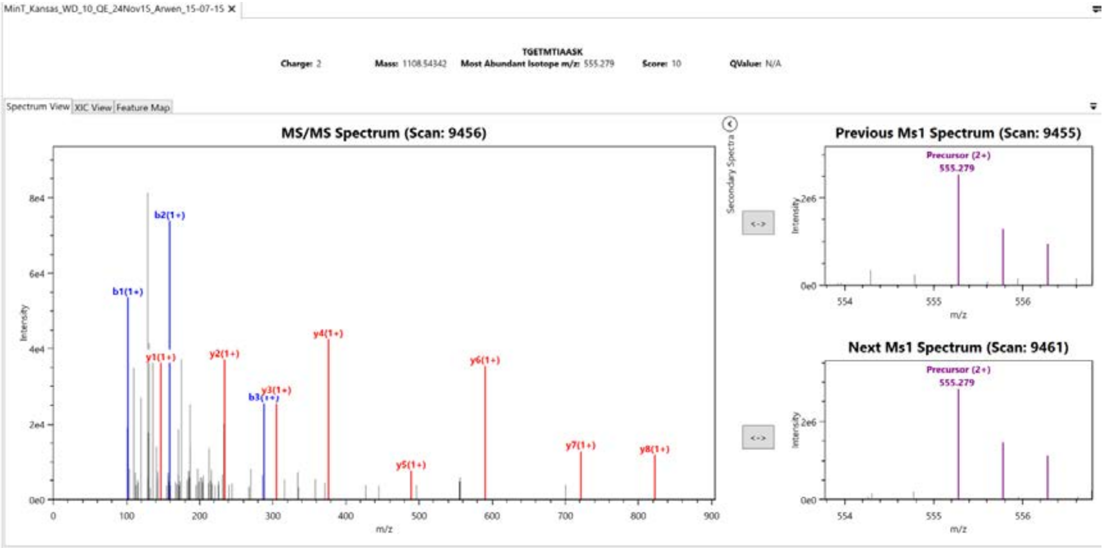
MinT_Kansas_WD_10_QE_24Nov15_Arwen_15-07-15
TGETMTIAASK
Charge: 2
Mass: 1108.54342
Most Abundant Isotope m/z: 555.279
Score: 10
QValue: N/A
Spectrum View
XIC View
Feature Map
MS/MS Spectrum (Scan: 9456)
Intensity
m/z
b1(1+)
b2(1+)
b3(1+)
y1(1+)
y2(1+)
y3(1+)
y4(1+)
y5(1+)
y6(1+)
y7(1+)
y8(1+)
Secondary Spectra
Previous Ms1 Spectrum (Scan: 9455)
Precursor (2+)
555.279
Next Ms1 Spectrum (Scan: 9461)
Precursor (2+)
555.279

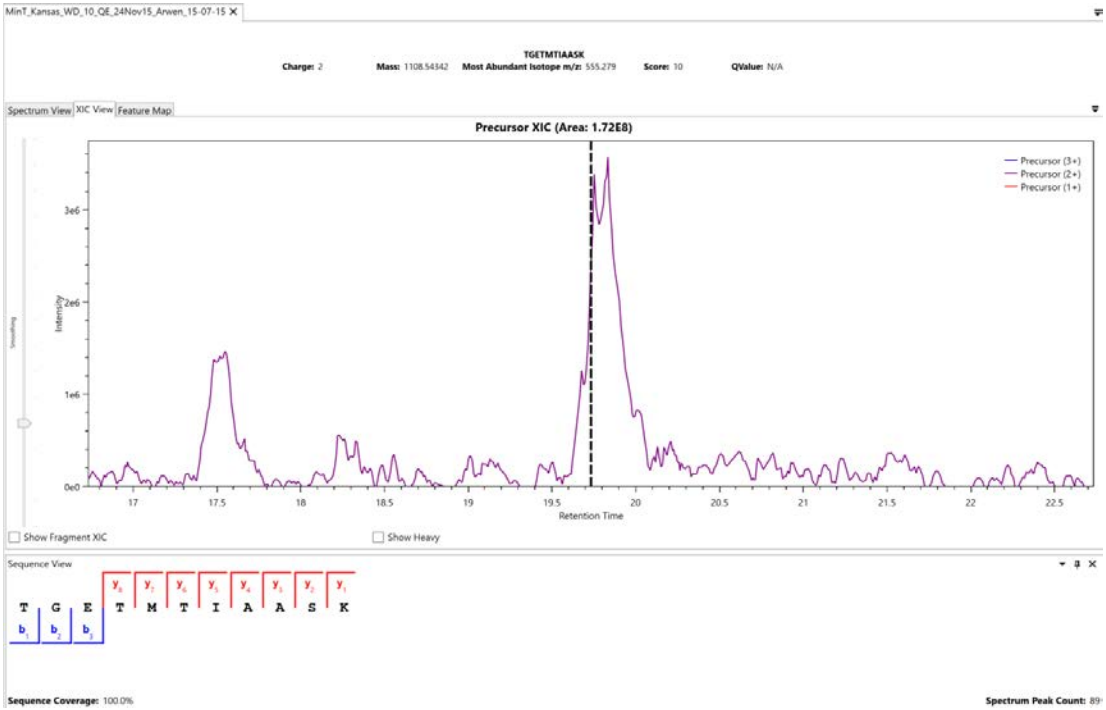
MinT_Kansas_WD_10_QE_24Nov15_Arwen_15-07-15
TGETMTIAASK
Charge: 2
Mass: 1108.54342
Most Abundant Isotope m/z: 555.279
Score: 10
QValue: N/A
Spectrum View
XIC View
Feature Map
Precursor XIC (Area: 1.72E8)
Precursor (3+)
Precursor (2+)
Precursor (1+)
Intensity
Retention Time
Show Fragment XIC
Show Heavy
Sequence View
T G E T M T I A A S K
Sequence Coverage: 100.0%
Spectrum Peak Count: 89

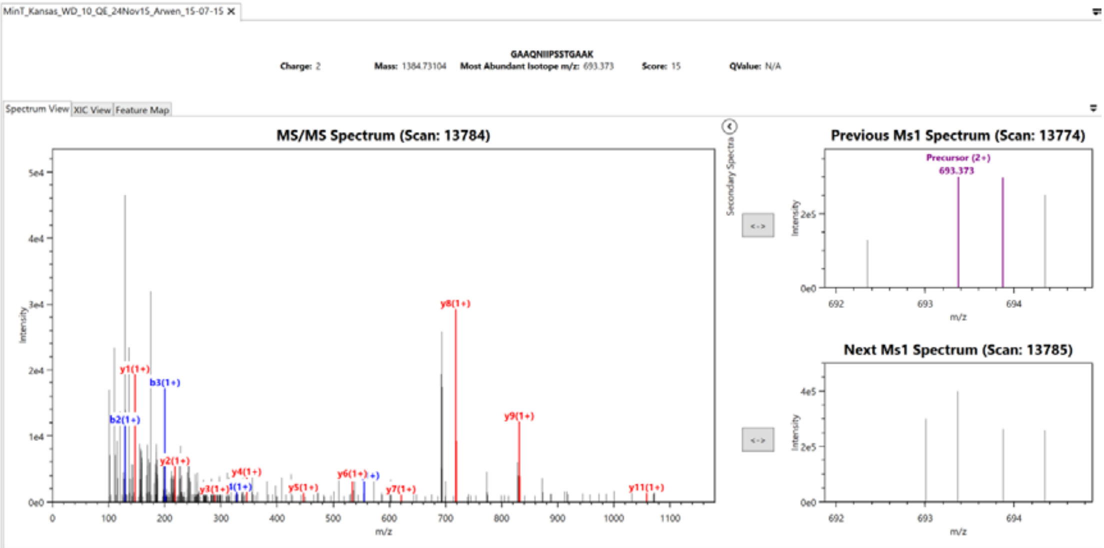
MinT_Kansas_WD_10_QE_24Nov15_Arwen_15-07-15
GAAQNIIPSSTGAAK
Charge: 2
Mass: 1384.73104
Most Abundant Isotope m/z: 693.373
Score: 15
QValue: N/A
Spectrum View
XIC View
Feature Map
MS/MS Spectrum (Scan: 13784)
Previous Ms1 Spectrum (Scan: 13774)
Precursor (2+)
693.373
Next Ms1 Spectrum (Scan: 13785)
Secondary Spectra
Intensity
m/z
y1(1+)
b2(1+)
b3(1+)
y2(1+)
y3(1+)
y4(1+)
y5(1+)
y6(1+)
y7(1+)
y8(1+)
y9(1+)
y11(1+)

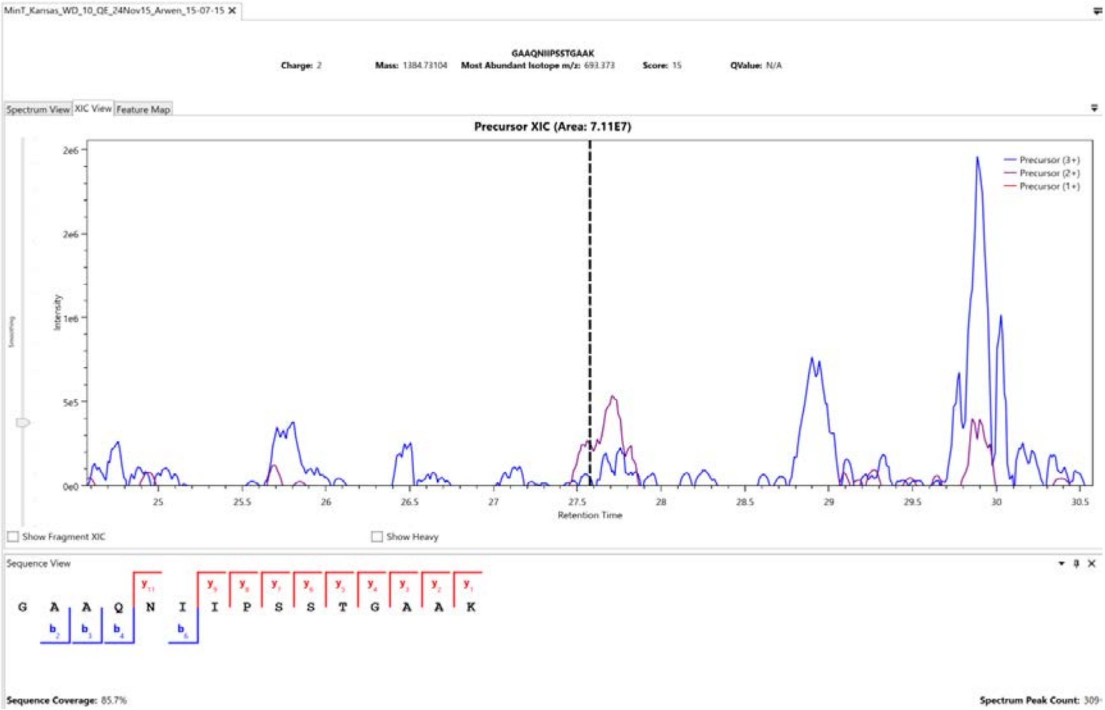
MinT_Kansas_WD_10_QE_24Nov15_Arwen_15-07-15
GAAQNIIPSSTGAAK
Charge: 2
Mass: 1384.73104
Most Abundant Isotope m/z: 693.373
Score: 15
QValue: N/A
Spectrum View
XIC View
Feature Map
Precursor XIC (Area: 7.11E7)
Precursor (3+)
Precursor (2+)
Precursor (1+)
Intensity
Retention Time
Smoothing
Show Fragment XIC
Show Heavy
Sequence View
G A A Q N I I P S S T G A A K
Sequence Coverage: 85.7%
Spectrum Peak Count: 309

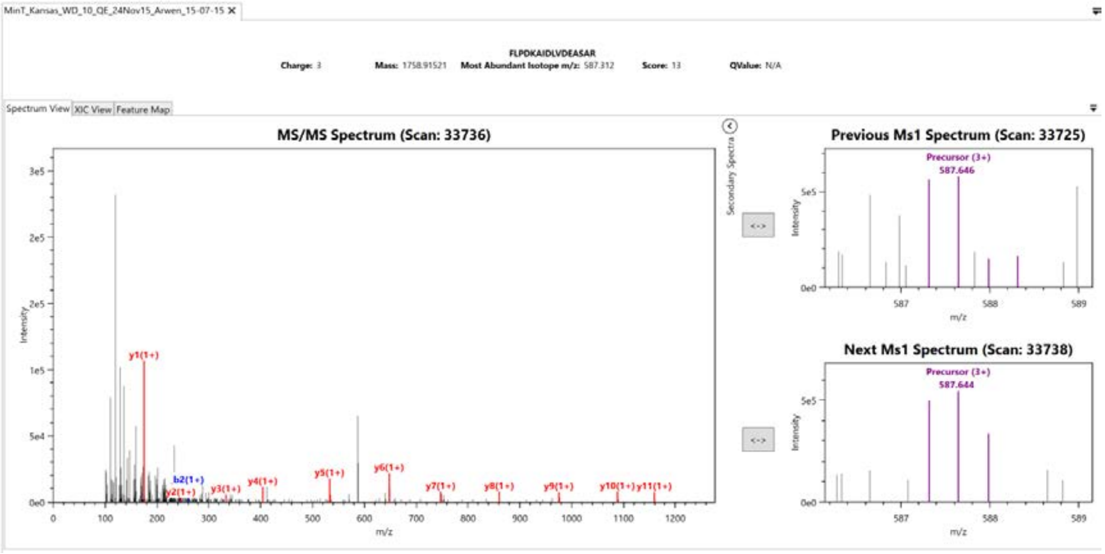
MinT_Kansas_WD_10_QE_24Nov15_Arwen_15-07-15
FLPDKAIDLVDEASAR
Charge: 3
Mass: 1758.91521
Most Abundant Isotope m/z: 587.312
Score: 13
QValue: N/A
Spectrum View
XIC View
Feature Map
MS/MS Spectrum (Scan: 33736)
Intensity
m/z
y1(1+)
b2(1+)
y2(1+)
y3(1+)
y4(1+)
y5(1+)
y6(1+)
y7(1+)
y8(1+)
y9(1+)
y10(1+)
y11(1+)
Secondary Spectra
Previous Ms1 Spectrum (Scan: 33725)
Precursor (3+)
587.646
Next Ms1 Spectrum (Scan: 33738)
Precursor (3+)
587.644

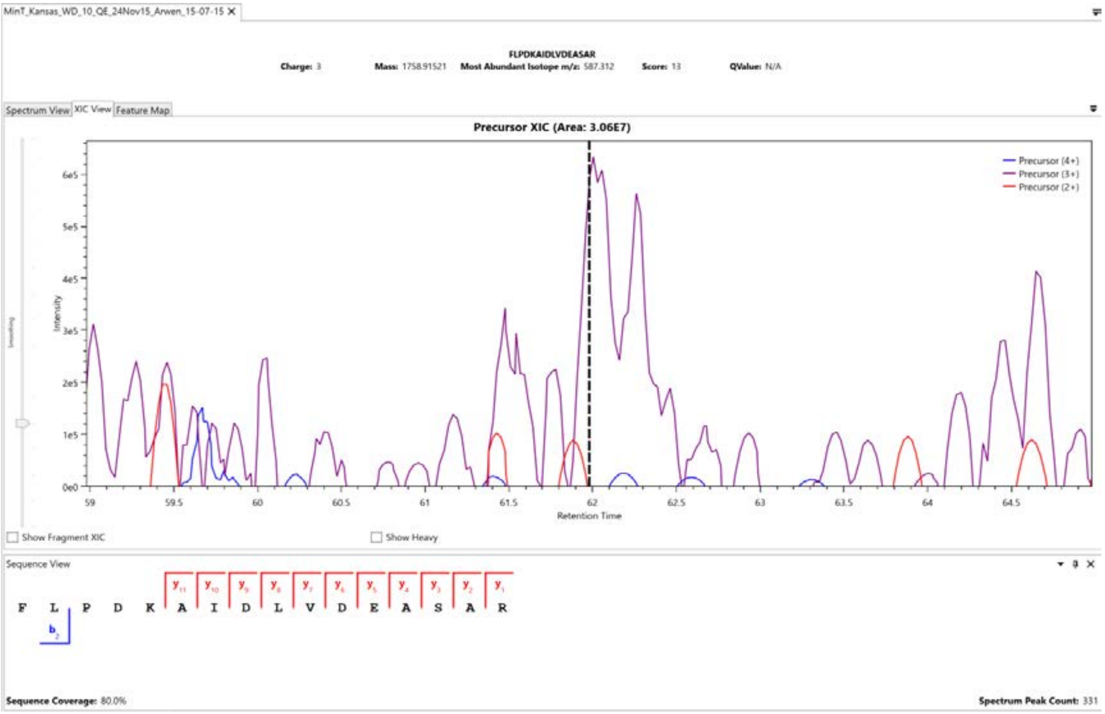
MinT_Kansas_WD_10_QE_24Nov15_Arwen_15-07-15
FLPDKAIDLVDEASAR
Charge: 3
Mass: 1758.91521
Most Abundant Isotope m/z: 587.312
Score: 13
QValue: N/A
Spectrum View
XIC View
Feature Map
Precursor XIC (Area: 3.06E7)
Precursor (4+)
Precursor (3+)
Precursor (2+)
Intensity
Retention Time
Show Fragment XIC
Show Heavy
Sequence View
F L P D K A I D L V D E A S A R
Sequence Coverage: 80.0%
Spectrum Peak Count: 331

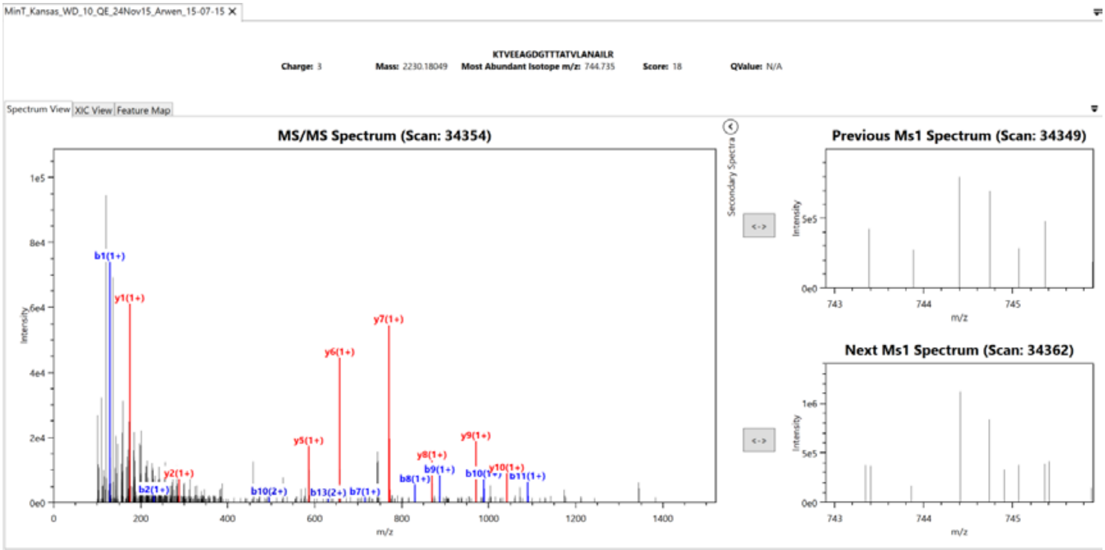
MinT_Kansas_WD_10_QE_24Nov15_Arwen_15-07-15
KTVEEAGDGTTTATVLANAILR
Charge: 3
Mass: 2230.18049
Most Abundant Isotope m/z: 744.735
Score: 18
QValue: N/A
Spectrum View
XIC View
Feature Map
MS/MS Spectrum (Scan: 34354)
Intensity
m/z
Secondary Spectra
Previous Ms1 Spectrum (Scan: 34349)
Next Ms1 Spectrum (Scan: 34362)

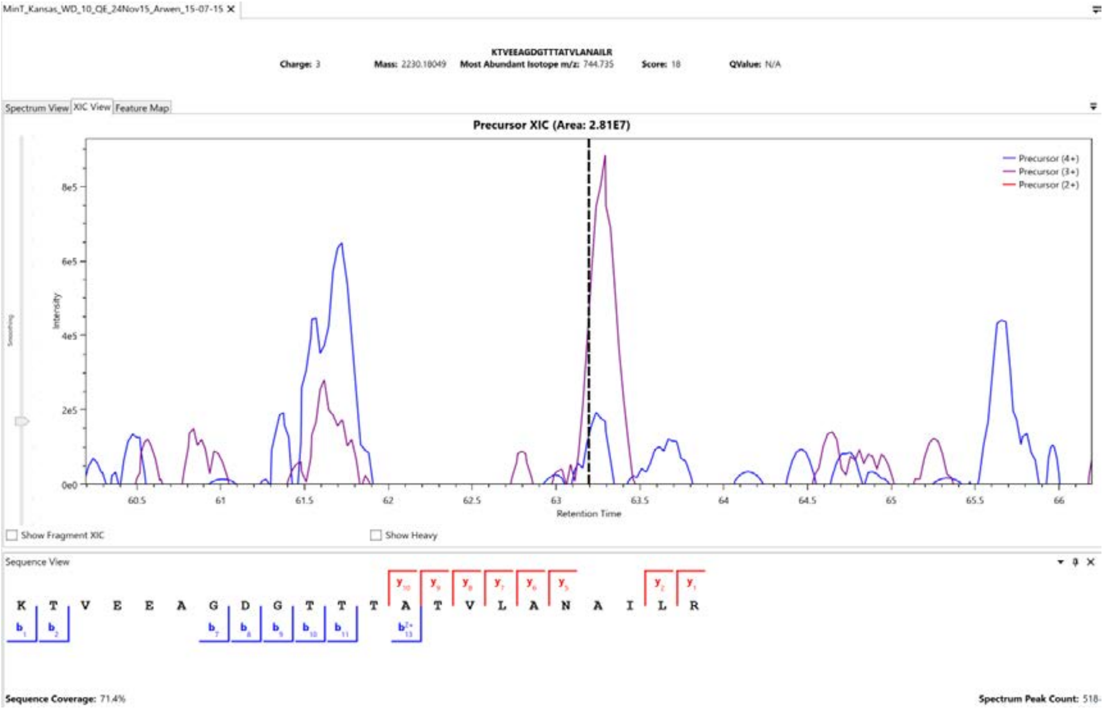
MinT_Kansas_WD_10_QE_24Nov15_Arwen_15-07-15
KTVEEAGDGTTTATVLANAILR
Charge: 3
Mass: 2230.18049
Most Abundant Isotope m/z: 744.735
Score: 18
QValue: N/A
Spectrum View
XIC View
Feature Map
Precursor XIC (Area: 2.81E7)
Precursor (4+)
Precursor (3+)
Precursor (2+)
Intensity
Retention Time
Smoothing
Show Fragment XIC
Show Heavy
Sequence View
K T V E E A G D G T T T A T V L A N A I L R
Sequence Coverage: 71.4%
Spectrum Peak Count: 518

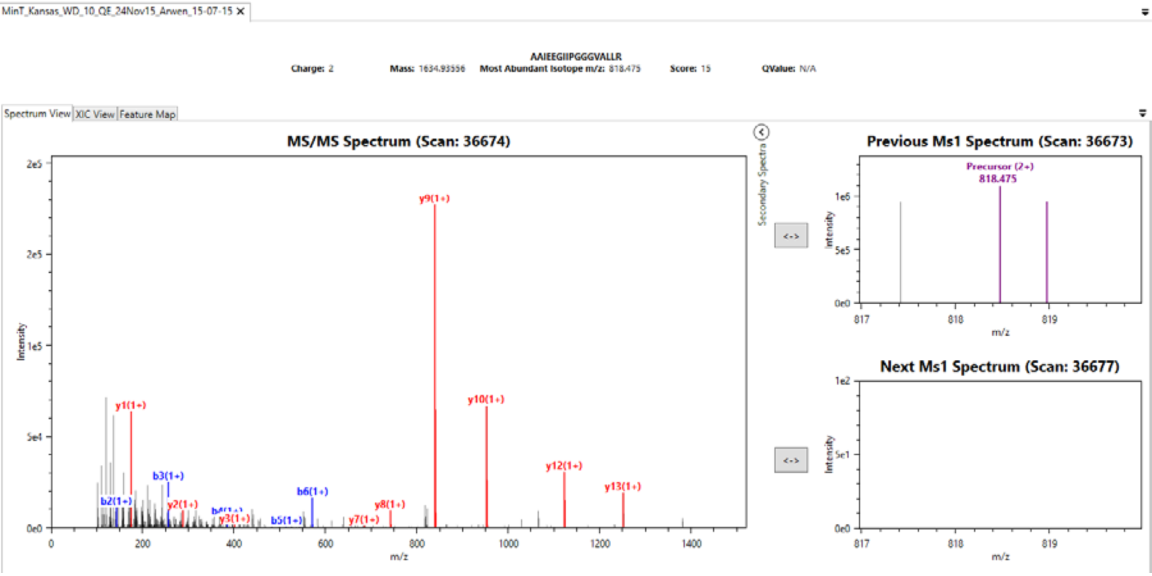
MinT_Kansas_WD_10_QE_24Nov15_Arwen_15-07-15
AAIEEGIIPGGGVALLR
Charge: 2
Mass: 1634.93556
Most Abundant Isotope m/z: 818.475
Score: 15
QValue: N/A
Spectrum View
XIC View
Feature Map
MS/MS Spectrum (Scan: 36674)
Previous Ms1 Spectrum (Scan: 36673)
Next Ms1 Spectrum (Scan: 36677)
Precursor (2+)
818.475
Intensity
m/z
Secondary Spectra
y9(1+)
y10(1+)
y1(1+)
y12(1+)
y13(1+)
b3(1+)
b6(1+)
b2(1+)
y2(1+)
y8(1+)
y3(1+)
y7(1+)
b5(1+)

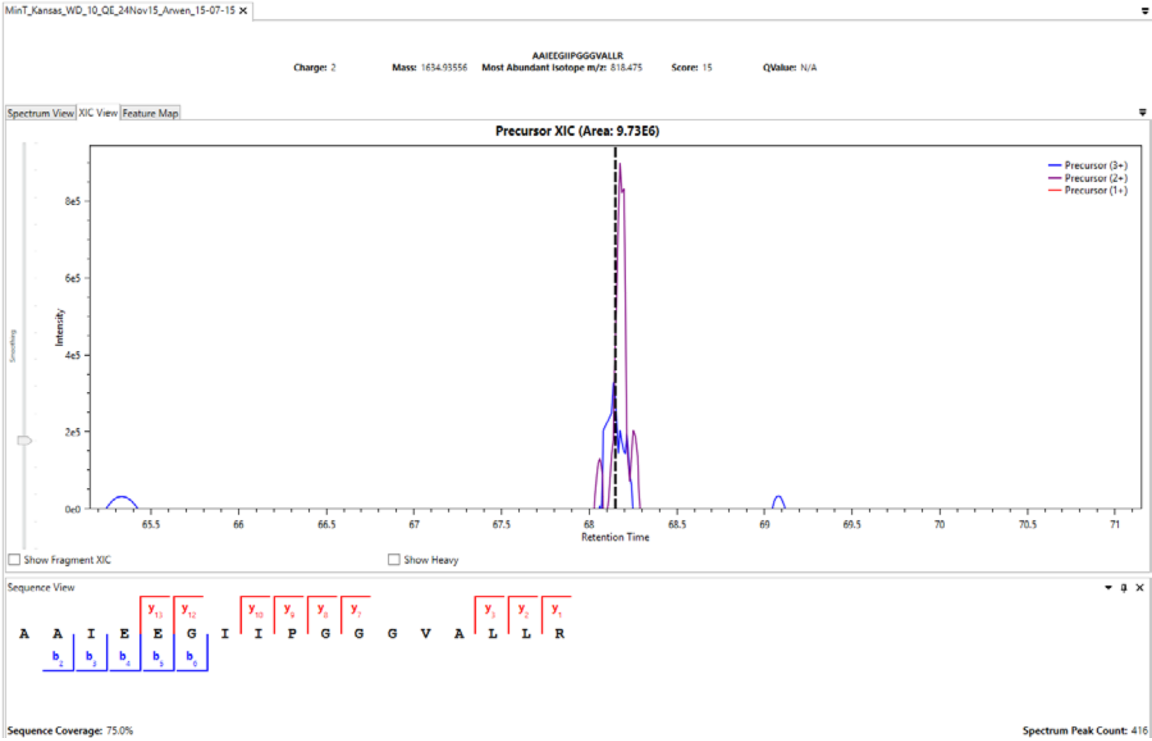
MinT_Kansas_WD_10_QE_24Nov15_Arwen_15-07-15
AAIEEGIIPGGGVALLR
Charge: 2
Mass: 1634.93556
Most Abundant Isotope m/z: 818.475
Score: 15
QValue: N/A
Spectrum View
XIC View
Feature Map
Precursor XIC (Area: 9.73E6)
Precursor (3+)
Precursor (2+)
Precursor (1+)
Intensity
Retention Time
Show Fragment XIC
Show Heavy
Sequence View
A A I E E G I I P G G G V A L L R
Sequence Coverage: 75.0%
Spectrum Peak Count: 416

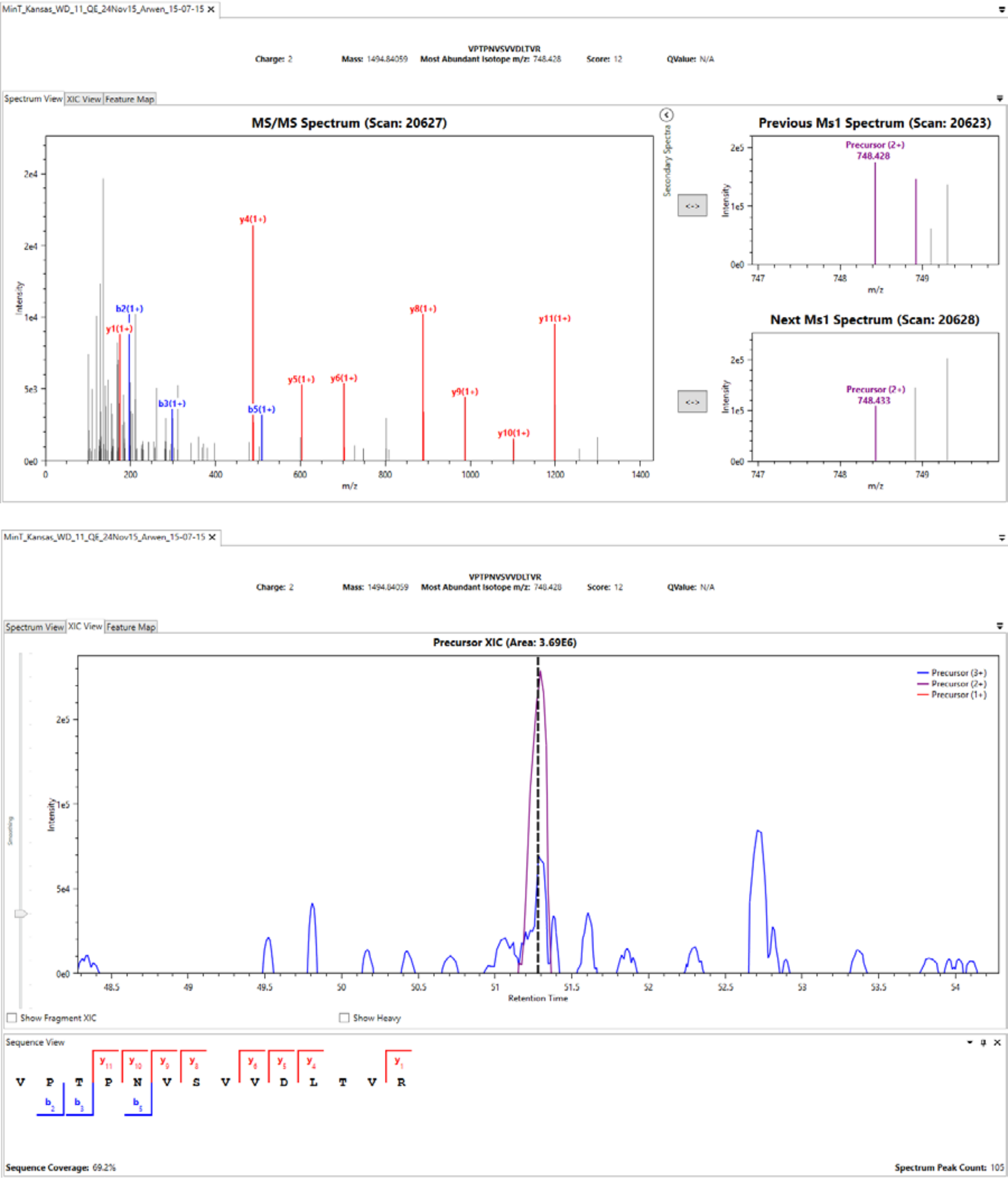

MinT_Kansas_WD_11_QE_24Nov15_Arwen_15-07-15
VPTPNVSVVDLTVR
Charge: 2
Mass: 1494.84059
Most Abundant Isotope m/z: 748.428
Score: 12
QValue: N/A
Spectrum View
XIC View
Feature Map
MS/MS Spectrum (Scan: 20627)
Intensity
m/z
y4(1+)
b2(1+)
y1(1+)
y8(1+)
y11(1+)
b3(1+)
b5(1+)
y5(1+)
y6(1+)
y9(1+)
y10(1+)
Secondary Spectra
Previous Ms1 Spectrum (Scan: 20623)
Precursor (2+)
748.428
Next Ms1 Spectrum (Scan: 20628)
Precursor (2+)
748.433
Precursor XIC (Area: 3.69E6)
Precursor (3+)
Precursor (2+)
Precursor (1+)
Retention Time
Smoothing
Show Fragment XIC
Show Heavy
Sequence View
V P T P N V S V V D L T V R
Sequence Coverage: 69.2%
Spectrum Peak Count: 105

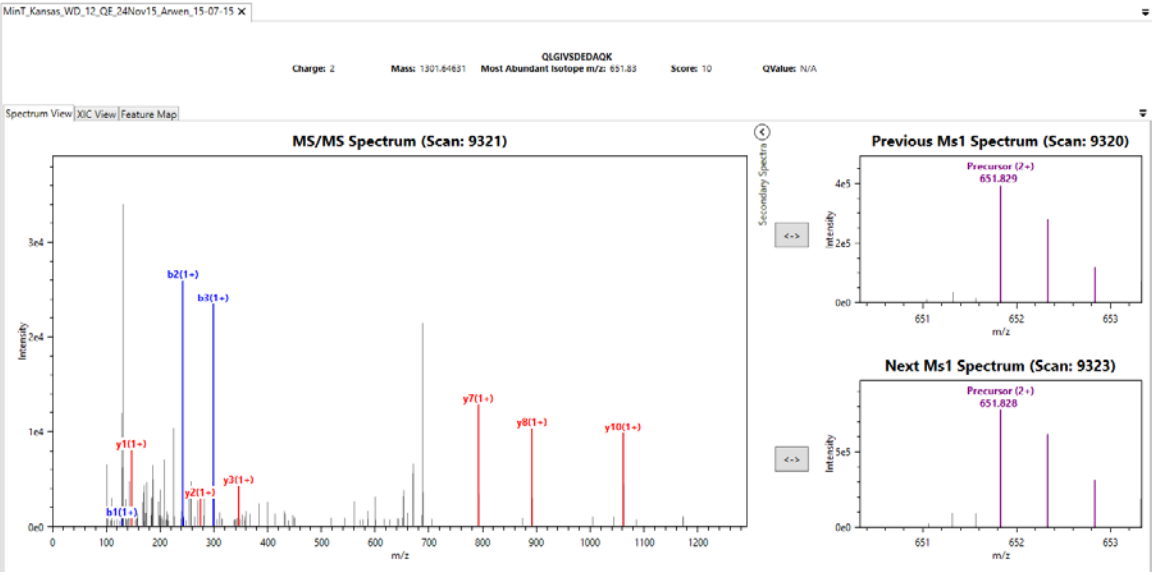
MinT_Kansas_WD_12_QE_24Nov15_Arwen_15-07-15
QLGIVSDEDAQK
Charge: 2
Mass: 1301.64631
Most Abundant Isotope m/z: 651.83
Score: 10
QValue: N/A
Spectrum View
XIC View
Feature Map
MS/MS Spectrum (Scan: 9321)
Intensity
m/z
b1(1+)
b2(1+)
b3(1+)
y1(1+)
y2(1+)
y3(1+)
y7(1+)
y8(1+)
y10(1+)
Secondary Spectra
Previous Ms1 Spectrum (Scan: 9320)
Precursor (2+)
651.829
Next Ms1 Spectrum (Scan: 9323)
Precursor (2+)
651.828

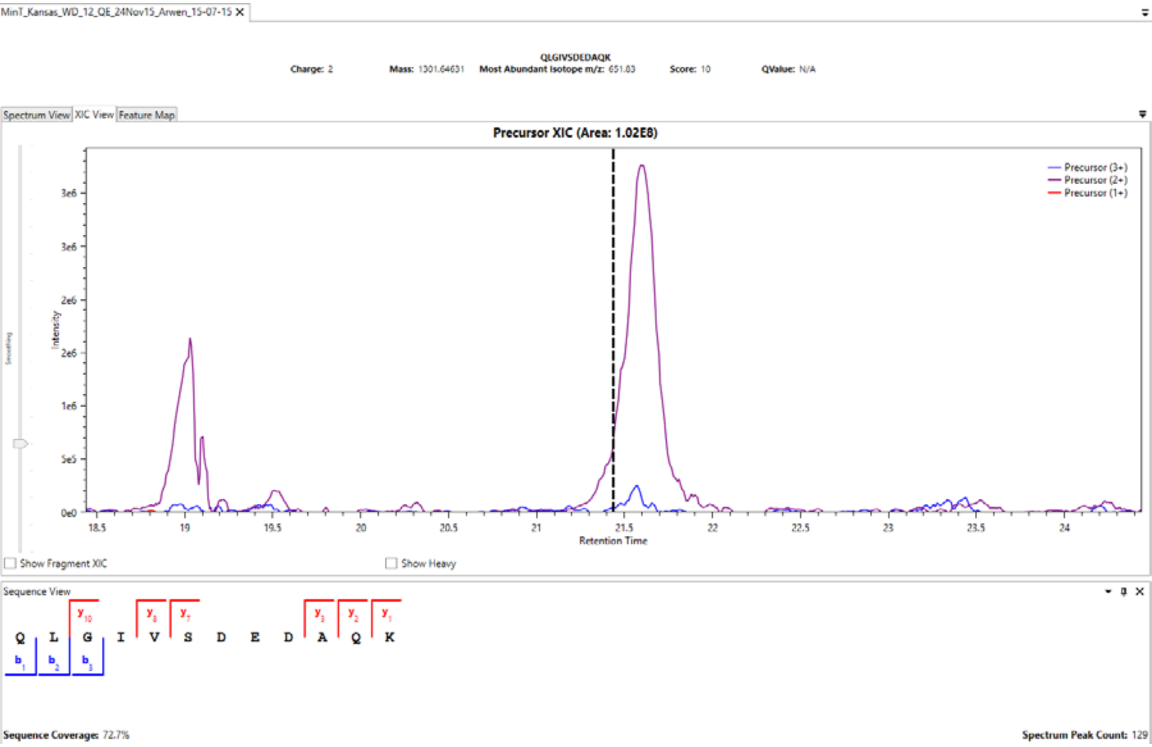
MinT_Kansas_WD_12_QE_24Nov15_Arwen_15-07-15
QLGIVSDEDAQK
Charge: 2
Mass: 1301.64631
Most Abundant Isotope m/z: 651.83
Score: 10
QValue: N/A
Spectrum View
XIC View
Feature Map
Precursor XIC (Area: 1.02E8)
Intensity
Retention Time
Precursor (3+)
Precursor (2+)
Precursor (1+)
Show Fragment XIC
Show Heavy
Sequence View
Q L G I V S D E D A Q K
Sequence Coverage: 72.7%
Spectrum Peak Count: 129

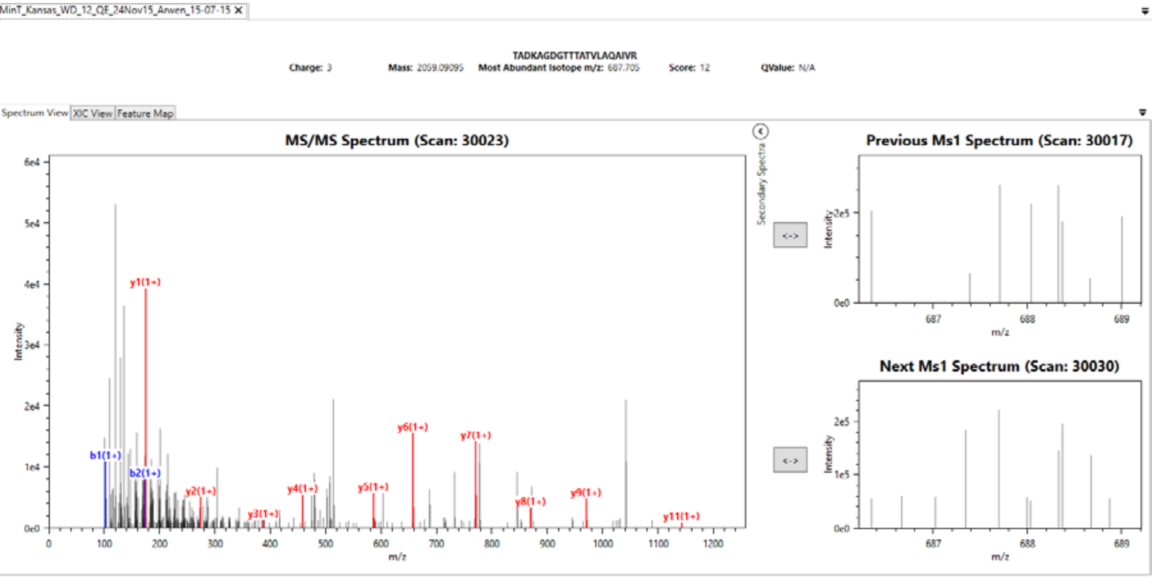
MinT_Kansas_WD_12_QE_24Nov15_Arwen_15-07-15
TADKAGDGTTTATVLAQAIVR
Charge: 3
Mass: 2059.09095
Most Abundant Isotope m/z: 687.705
Score: 12
QValue: N/A
Spectrum View
XIC View
Feature Map
MS/MS Spectrum (Scan: 30023)
Intensity
m/z
b1(1+)
b2(1+)
y1(1+)
y2(1+)
y3(1+)
y4(1+)
y5(1+)
y6(1+)
y7(1+)
y8(1+)
y9(1+)
y11(1+)
Secondary Spectra
Previous Ms1 Spectrum (Scan: 30017)
Next Ms1 Spectrum (Scan: 30030)

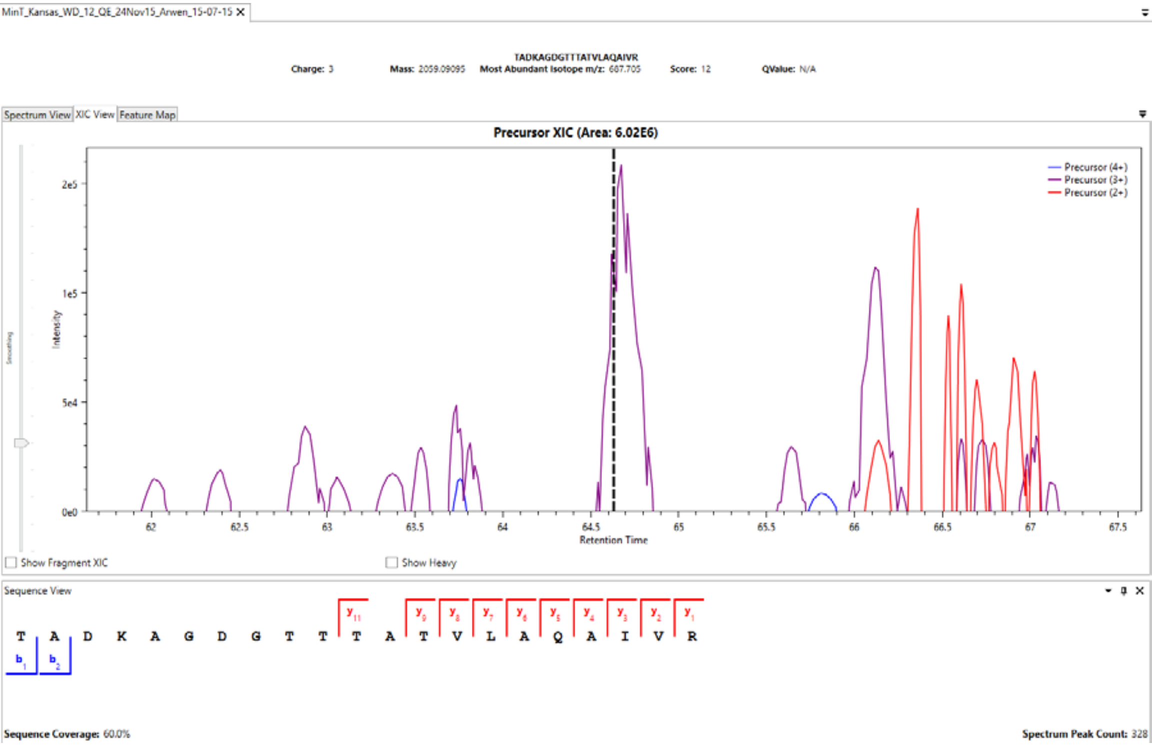
MinT_Kansas_WD_12_QE_24Nov15_Arwen_15-07-15
TADKAGDGTTTATVLAQAIVR
Charge: 3
Mass: 2059.09095
Most Abundant Isotope m/z: 687.705
Score: 12
QValue: N/A
Spectrum View
XIC View
Feature Map
Precursor XIC (Area: 6.02E6)
Precursor (4+)
Precursor (3+)
Precursor (2+)
Intensity
Retention Time
Show Fragment XIC
Show Heavy
Sequence View
T A D K A G D G T T T A T V L A Q A I V R
Sequence Coverage: 60.0%
Spectrum Peak Count: 328

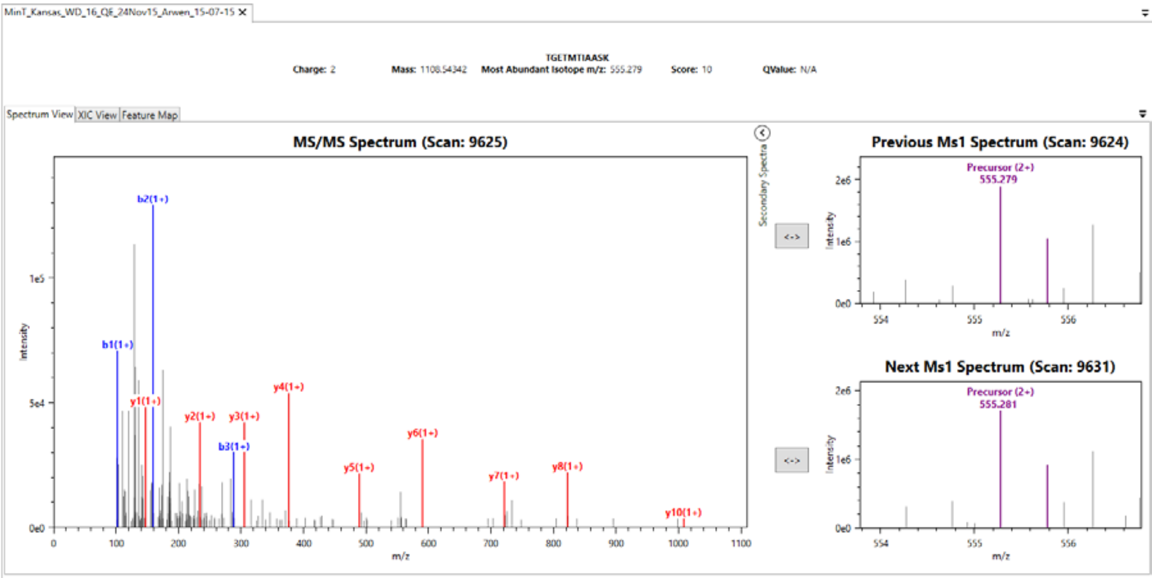
MinT_Kansas_WD_16_QE_24Nov15_Arwen_15-07-15
TGETMTIAASK
Charge: 2
Mass: 1108.54342
Most Abundant Isotope m/z: 555.279
Score: 10
QValue: N/A
Spectrum View
XIC View
Feature Map
MS/MS Spectrum (Scan: 9625)
b2(1+)
b1(1+)
y1(1+)
y2(1+)
y3(1+)
b3(1+)
y4(1+)
y5(1+)
y6(1+)
y7(1+)
y8(1+)
y10(1+)
Intensity
m/z
Secondary Spectra
Previous Ms1 Spectrum (Scan: 9624)
Precursor (2+)
555.279
Next Ms1 Spectrum (Scan: 9631)
Precursor (2+)
555.281

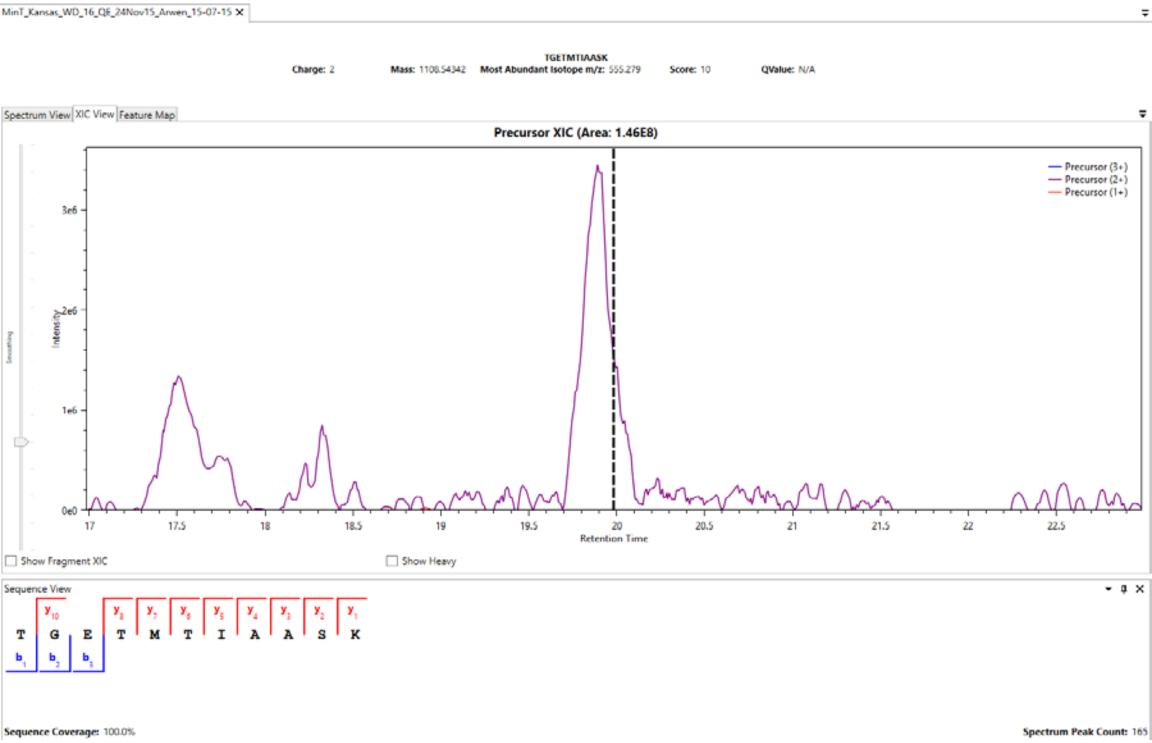
MinT_Kansas_WD_16_QE_24Nov15_Arwen_15-07-15
TGETMTIAASK
Charge: 2
Mass: 1108.54342
Most Abundant Isotope m/z: 555.279
Score: 10
QValue: N/A
Spectrum View
XIC View
Feature Map
Precursor XIC (Area: 1.46E8)
Precursor (3+)
Precursor (2+)
Precursor (1+)
Intensity
Smoothing
Retention Time
Show Fragment XIC
Show Heavy
Sequence View
T G E T M T I A A S K
Sequence Coverage: 100.0%
Spectrum Peak Count: 165

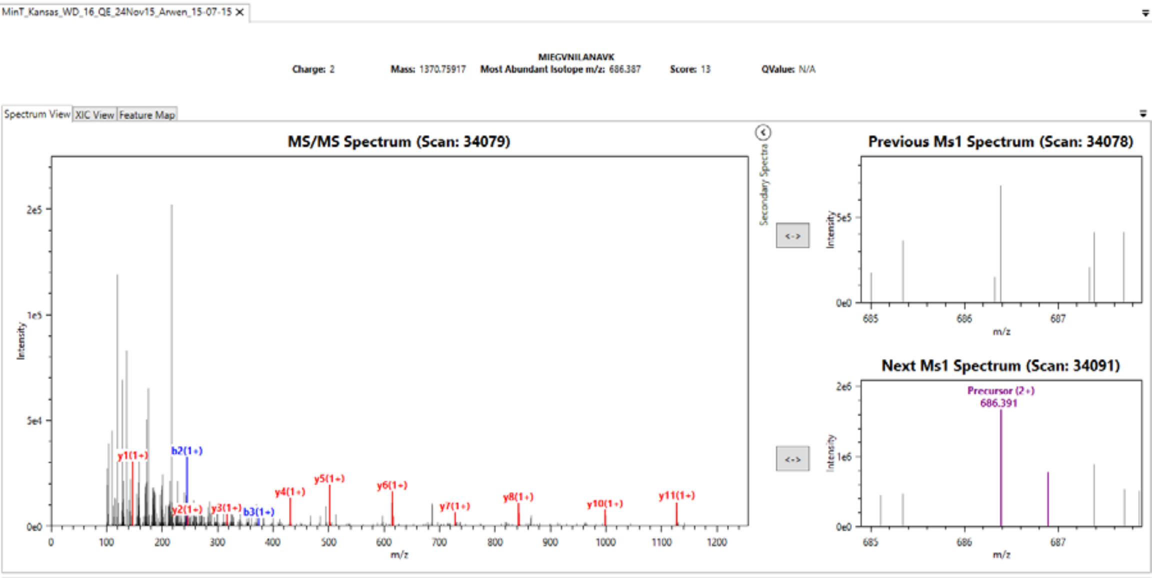

MinT_Kansas_WD_16_QE_24Nov15_Arwen_15-07-15
MIEGVNILANAVK
Charge: 2
Mass: 1370.75917
Most Abundant Isotope m/z: 686.387
Score: 13
QValue: N/A
Spectrum View
XIC View
Feature Map
MS/MS Spectrum (Scan: 34079)
Intensity
m/z
y1(1+)
b2(1+)
y2(1+)
y3(1+)
b3(1+)
y4(1+)
y5(1+)
y6(1+)
y7(1+)
y8(1+)
y10(1+)
y11(1+)
Secondary Spectra
Previous Ms1 Spectrum (Scan: 34078)
Next Ms1 Spectrum (Scan: 34091)
Precursor (2+)
686.391

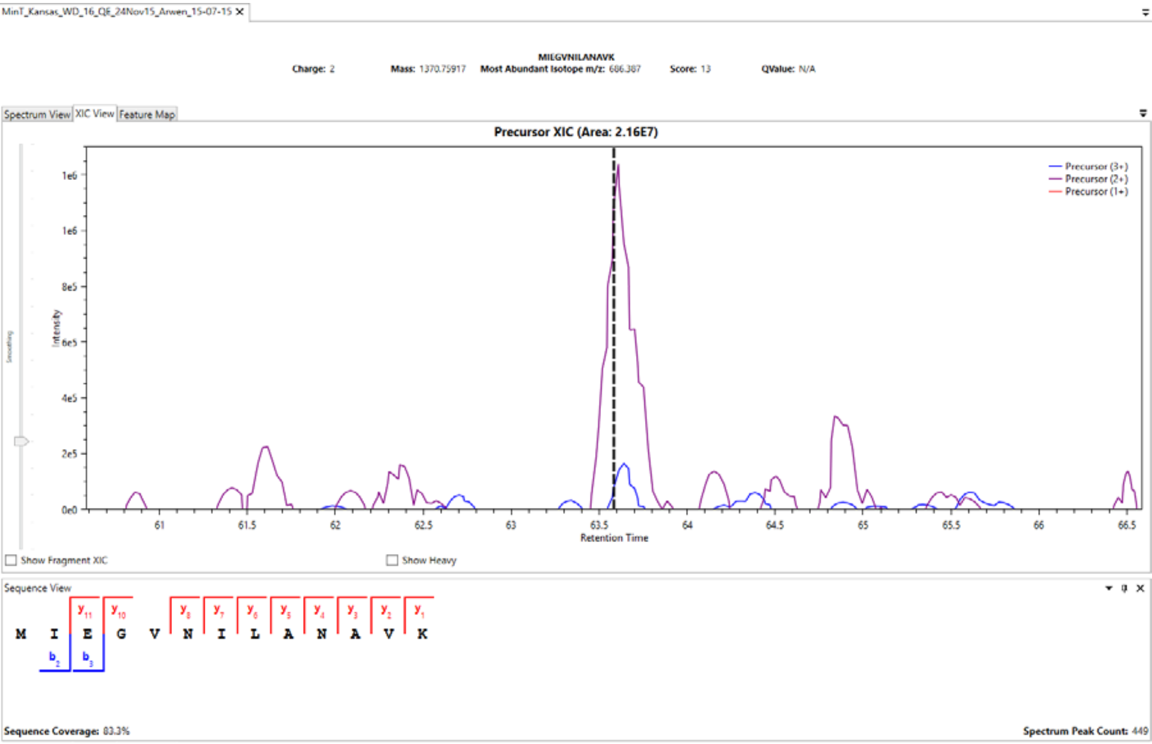

MinT_Kansas_WD_16_QE_24Nov15_Arwen_15-07-15
MIEGVNILANAVK
Charge: 2
Mass: 1370.75917
Most Abundant Isotope m/z: 686.387
Score: 13
QValue: N/A
Spectrum View
XIC View
Feature Map
Precursor XIC (Area: 2.16E7)
Precursor (3+)
Precursor (2+)
Precursor (1+)
Intensity
Retention Time
Show Fragment XIC
Show Heavy
Sequence View
M I E G V N I L A N A V K
Sequence Coverage: 83.3%
Spectrum Peak Count: 449

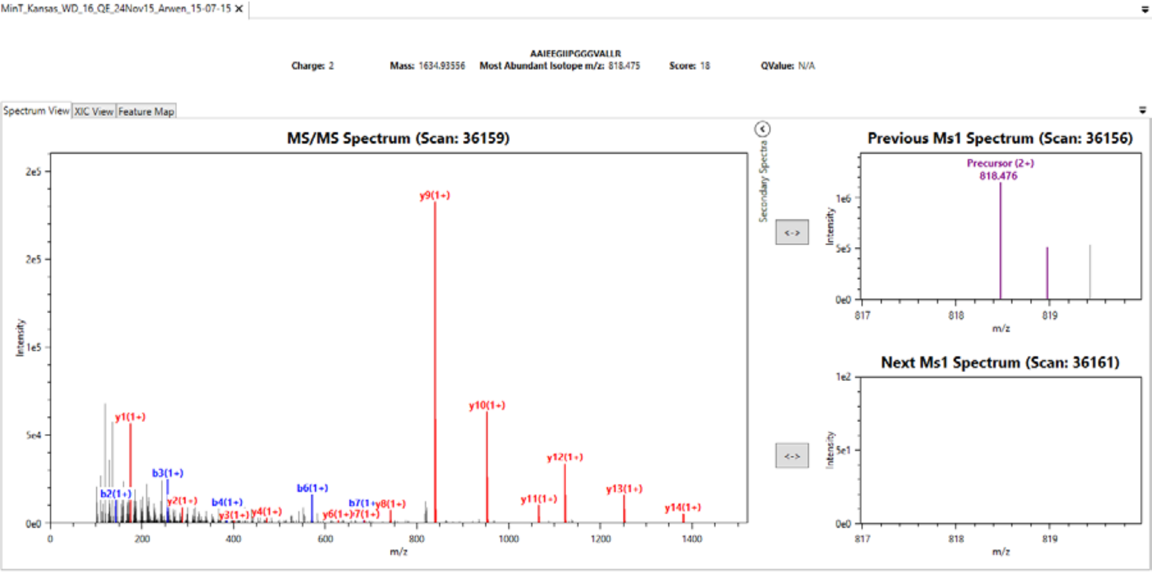
MinT_Kansas_WD_16_QE_24Nov15_Arwen_15-07-15
AAIEEGIIPGGGVALLR
Charge: 2
Mass: 1634.93556
Most Abundant Isotope m/z: 818.475
Score: 18
QValue: N/A
Spectrum View
XIC View
Feature Map
MS/MS Spectrum (Scan: 36159)
Intensity
m/z
y9(1+)
y10(1+)
y1(1+)
b3(1+)
b2(1+)
y2(1+)
b4(1+)
y3(1+)
y4(1+)
b6(1+)
y6(1+)
b7(1+)
y7(1+)
y8(1+)
y11(1+)
y12(1+)
y13(1+)
y14(1+)
Secondary Spectra
Previous Ms1 Spectrum (Scan: 36156)
Precursor (2+)
818.476
Next Ms1 Spectrum (Scan: 36161)

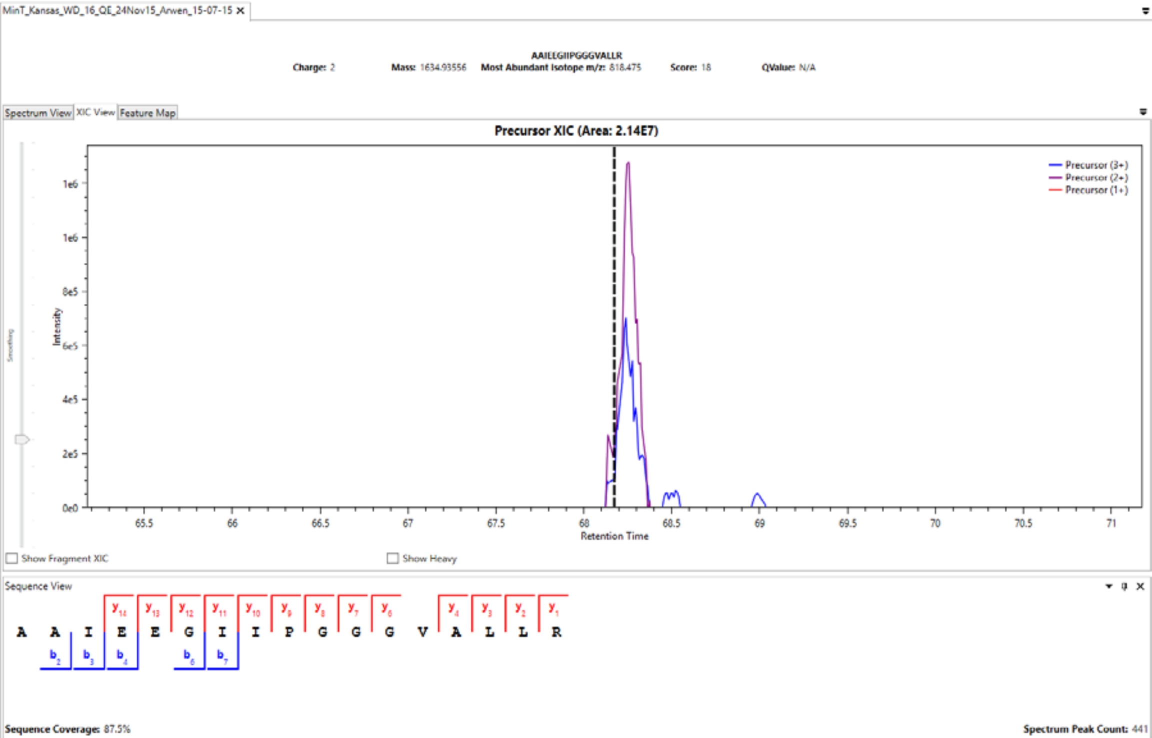
MinT_Kansas_WD_16_QE_24Nov15_Arwen_15-07-15
AAIEEGIIPGGGVALLR
Charge: 2
Mass: 1634.93556
Most Abundant Isotope m/z: 818.475
Score: 18
QValue: N/A
Spectrum View
XIC View
Feature Map
Precursor XIC (Area: 2.14E7)
Intensity
Retention Time
Precursor (3+)
Precursor (2+)
Precursor (1+)
Show Fragment XIC
Show Heavy
Sequence View
A A I E E G I I P G G G V A L L R
Sequence Coverage: 87.5%
Spectrum Peak Count: 441

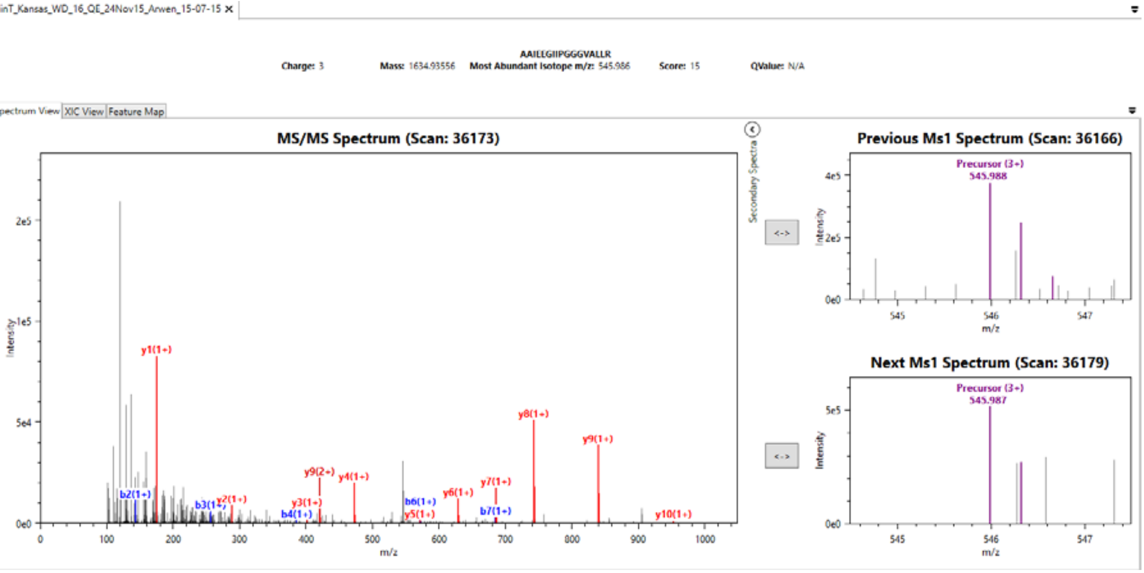

inT_Kansas_WD_16_QE_24Nov15_Arwen_15-07-15
AAIEEGIIPGGGVALLR
Charge: 3
Mass: 1634.93556
Most Abundant Isotope m/z: 545.986
Score: 15
QValue: N/A
pectrum View
XIC View
Feature Map
MS/MS Spectrum (Scan: 36173)
Intensity
m/z
y1(1+)
y8(1+)
y9(1+)
y9(2+)
y4(1+)
y7(1+)
y6(1+)
b2(1+)
b3(1+)
y2(1+)
y3(1+)
b4(1+)
b6(1+)
y5(1+)
b7(1+)
y10(1+)
Secondary Spectra
Previous Ms1 Spectrum (Scan: 36166)
Precursor (3+)
545.988
Next Ms1 Spectrum (Scan: 36179)
Precursor (3+)
545.987

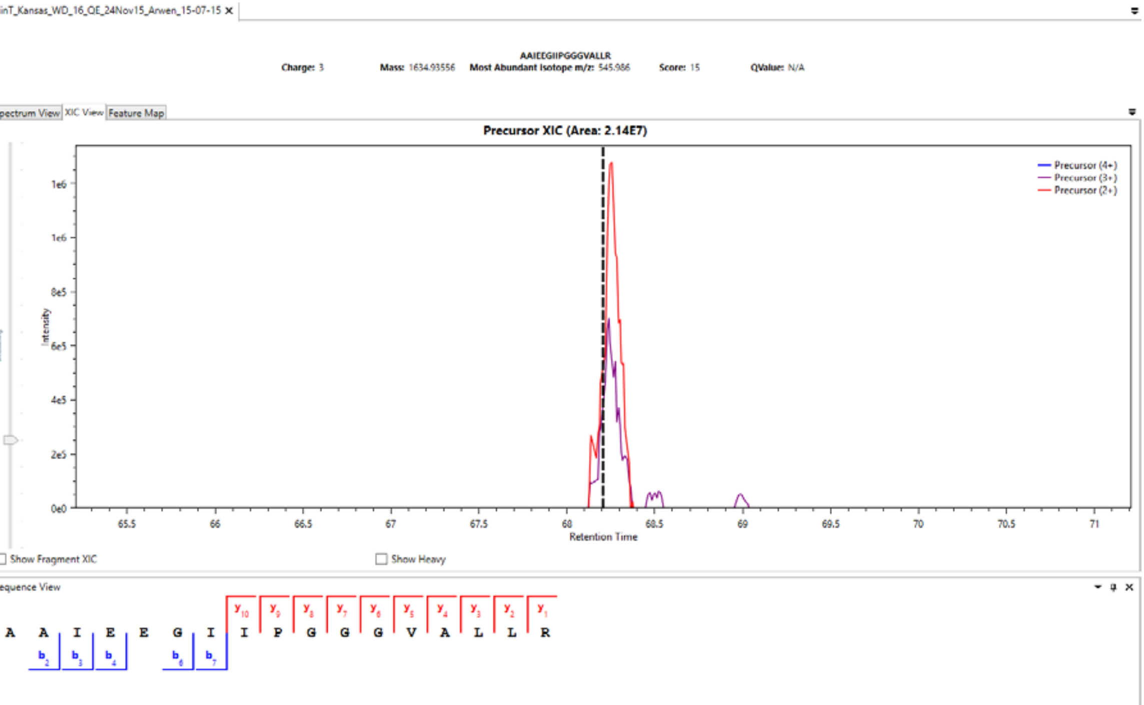

inT_Kansas_WD_16_QE_24Nov15_Arwen_15-07-15
AAIEEGIIPGGGVALLR
Charge: 3
Mass: 1634.93556
Most Abundant Isotope m/z: 545.986
Score: 15
QValue: N/A
pectrum View
XIC View
Feature Map
Precursor XIC (Area: 2.14E7)
Precursor (4+)
Precursor (3+)
Precursor (2+)
Intensity
Retention Time
Show Fragment XIC
Show Heavy
equence View
A A I E E G I I P G G G V A L L R

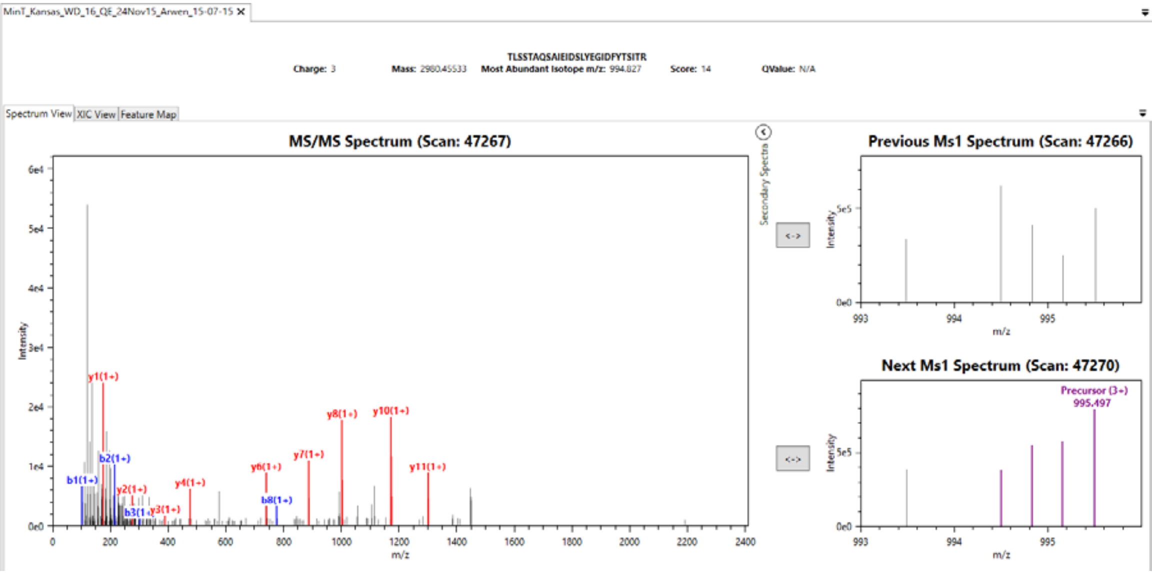

MinT_Kansas_WD_16_QE_24Nov15_Arwen_15-07-15
TLSSTAQSAIEIDSLYEGIDFYTSITR
Charge: 3
Mass: 2980.45533
Most Abundant Isotope m/z: 994.827
Score: 14
QValue: N/A
Spectrum View
XIC View
Feature Map
MS/MS Spectrum (Scan: 47267)
Intensity
m/z
y1(1+)
b2(1+)
b1(1+)
y2(1+)
b3(1+)
y3(1+)
y4(1+)
y6(1+)
b8(1+)
y7(1+)
y8(1+)
y10(1+)
y11(1+)
Secondary Spectra
Previous Ms1 Spectrum (Scan: 47266)
Next Ms1 Spectrum (Scan: 47270)
Precursor (3+)
995.497

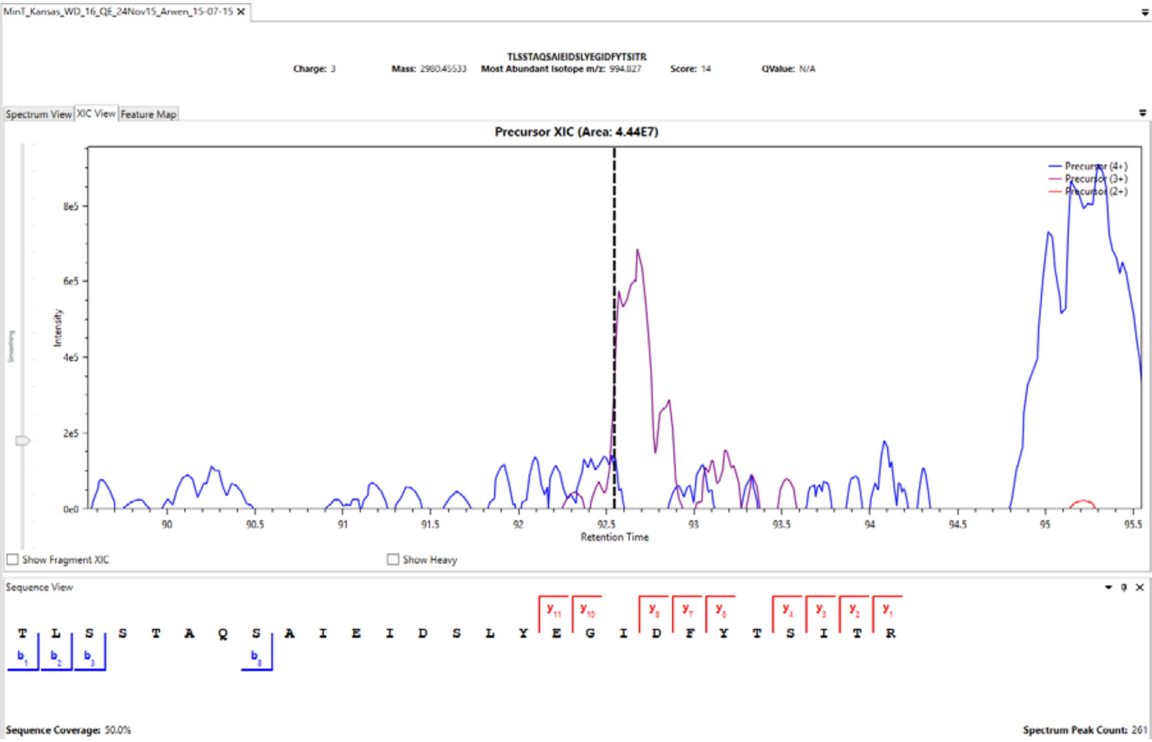

MinT_Kansas_WD_16_QE_24Nov15_Arwen_15-07-15
TLSSTAQSAIEIDSLYEGIDFYTSITR
Charge: 3
Mass: 2980.45533
Most Abundant Isotope m/z: 994.827
Score: 14
QValue: N/A
Spectrum View
XIC View
Feature Map
Precursor XIC (Area: 4.44E7)
Intensity
Retention Time
Precursor (4+)
Precursor (3+)
Precursor (2+)
Show Fragment XIC
Show Heavy
Sequence View
T L S S T A Q S A I E I D S L Y E G I D F Y T S I T R
Sequence Coverage: 50.0%
Spectrum Peak Count: 261

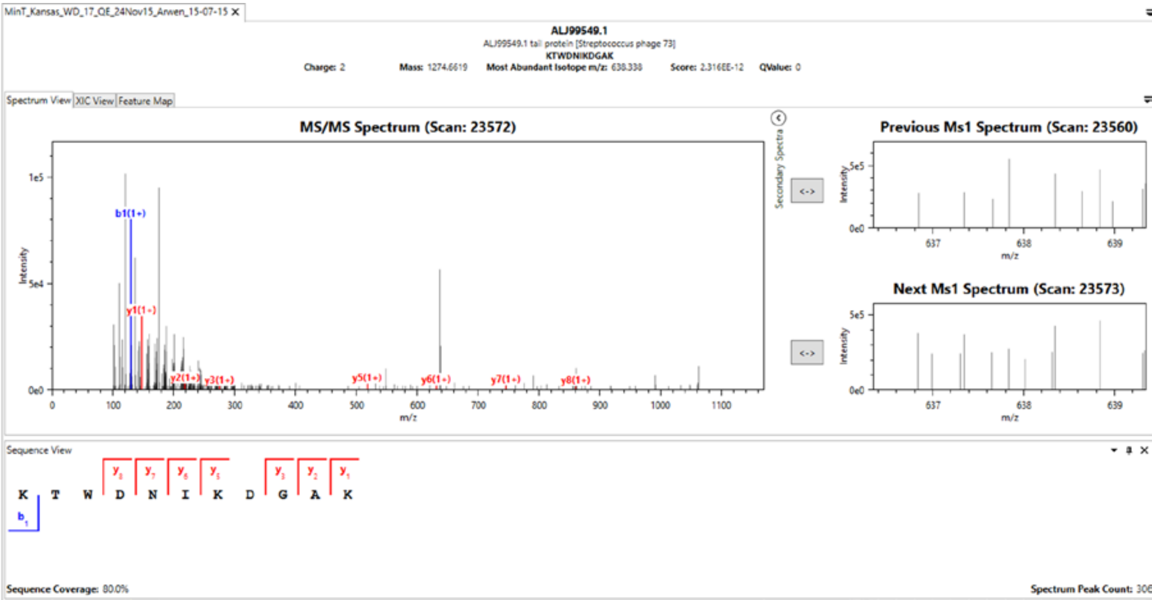
MinT_Kansas_WD_17_QE_24Nov15_Arwen_15-07-15
ALJ99549.1
ALJ99549.1 tail protein [Streptococcus phage 73]
KTWDNIKDGAK
Charge: 2
Mass: 1274.6619
Most Abundant Isotope m/z: 638.338
Score: 2.316EE-12
QValue: 0
Spectrum View
XIC View
Feature Map
MS/MS Spectrum (Scan: 23572)
Previous Ms1 Spectrum (Scan: 23560)
Next Ms1 Spectrum (Scan: 23573)
Secondary Spectra
Intensity
m/z
b1(1+)
y1(1+)
y2(1+)
y3(1+)
y5(1+)
y6(1+)
y7(1+)
y8(1+)
Sequence View
K T W D N I K D G A K
Sequence Coverage: 80.0%
Spectrum Peak Count: 306

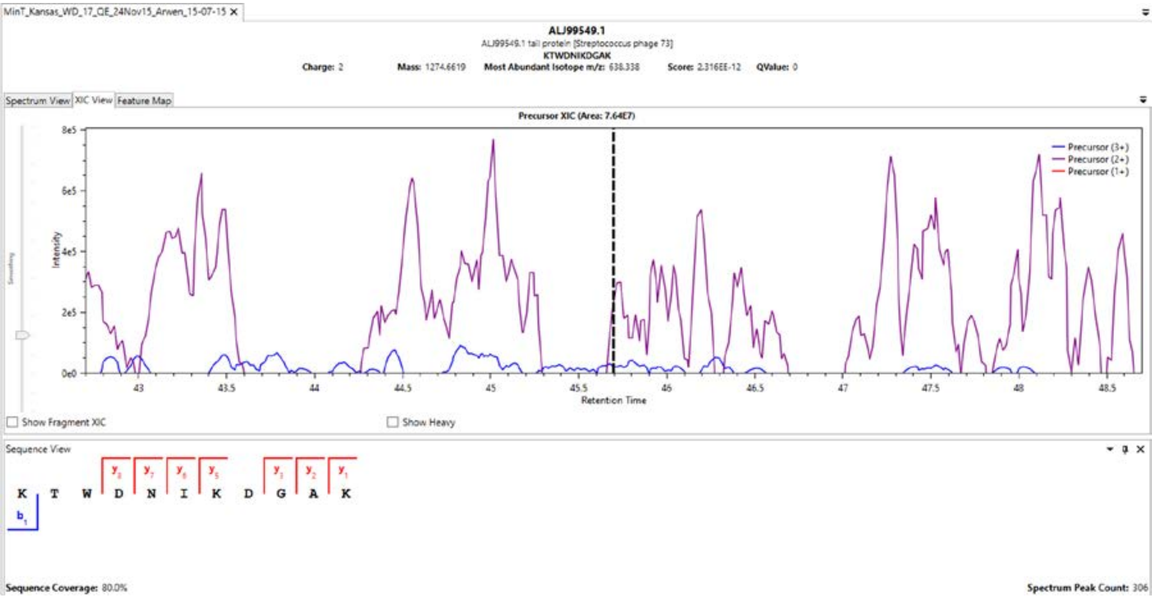
MinT_Kansas_WD_17_QE_24Nov15_Arwen_15-07-15
ALJ99549.1
ALJ99549.1 tail protein [Streptococcus phage 73]
KTWDNIKDGAK
Charge: 2
Mass: 1274.6619
Most Abundant Isotope m/z: 638.338
Score: 2.316EE-12
QValue: 0
Spectrum View
XIC View
Feature Map
Precursor XIC (Area: 7.64E7)
Precursor (3+)
Precursor (2+)
Precursor (1+)
Intensity
Retention Time
Show Fragment XIC
Show Heavy
Sequence View
K T W D N I K D G A K
Sequence Coverage: 80.0%
Spectrum Peak Count: 306

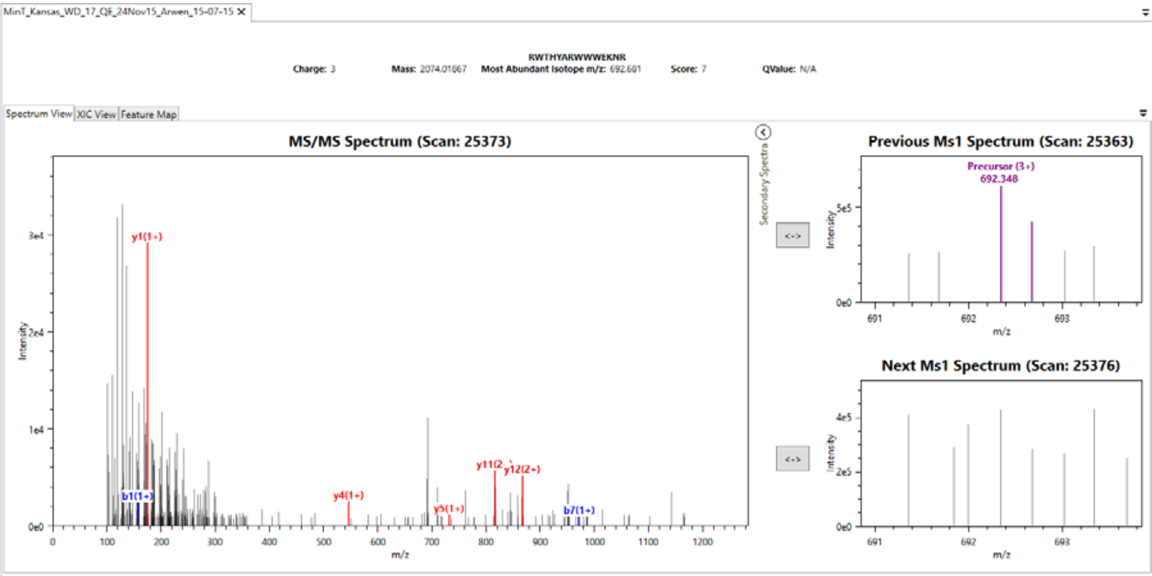
MinT_Kansas_WD_17_QE_24Nov15_Arwen_15-07-15
RWTHYARWWWEKNR
Charge: 3
Mass: 2074.01867
Most Abundant Isotope m/z: 692.681
Score: 7
QValue: N/A
Spectrum View
XIC View
Feature Map
MS/MS Spectrum (Scan: 25373)
Intensity
m/z
y1(1+)
b1(1+)
y4(1+)
y5(1+)
y12(2+)
b7(1+)
Secondary Spectra
Previous Ms1 Spectrum (Scan: 25363)
Precursor (3+)
692.348
Next Ms1 Spectrum (Scan: 25376)

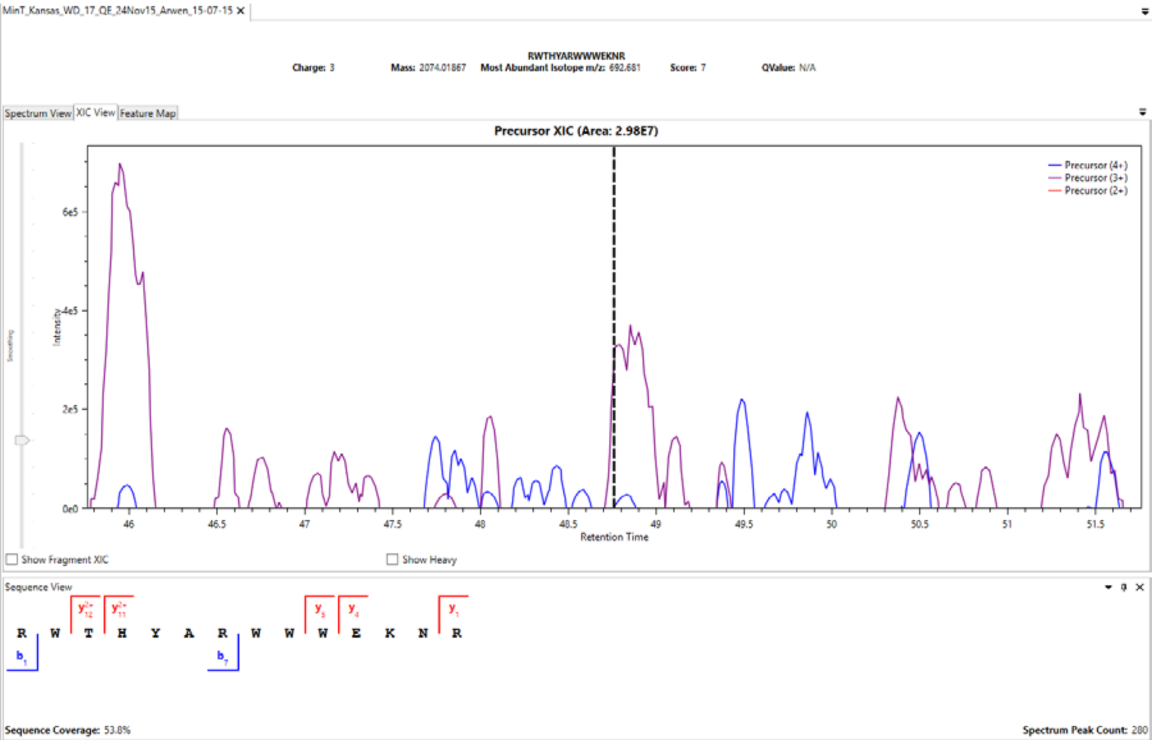
MinT_Kansas_WD_17_QE_24Nov15_Arwen_15-07-15
RWTHYARWWWEKNR
Charge: 3
Mass: 2074.01867
Most Abundant Isotope m/z: 692.681
Score: 7
QValue: N/A
Spectrum View
XIC View
Feature Map
Precursor XIC (Area: 2.98E7)
Intensity
Retention Time
Precursor (4+)
Precursor (3+)
Precursor (2+)
Show Fragment XIC
Show Heavy
Sequence View
R W T H Y A R W W W E K N R
Sequence Coverage: 53.8%
Spectrum Peak Count: 280

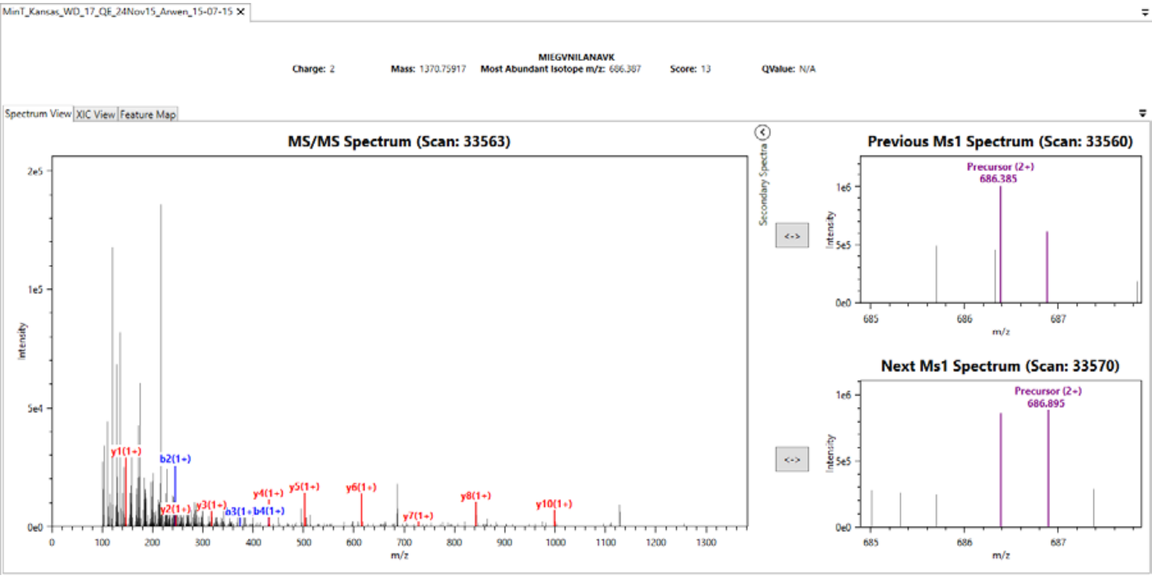
MinT_Kansas_WD_17_QE_24Nov15_Arwen_15-07-15
MIEGVNILANAVK
Charge: 2
Mass: 1370.75917
Most Abundant Isotope m/z: 686.387
Score: 13
QValue: N/A
Spectrum View
XIC View
Feature Map
MS/MS Spectrum (Scan: 33563)
Intensity
m/z
y1(1+)
b2(1+)
y2(1+)
y3(1+)
a3(1+)
b4(1+)
y4(1+)
y5(1+)
y6(1+)
y7(1+)
y8(1+)
y10(1+)
Secondary Spectra
Previous Ms1 Spectrum (Scan: 33560)
Precursor (2+)
686.385
Next Ms1 Spectrum (Scan: 33570)
Precursor (2+)
686.895

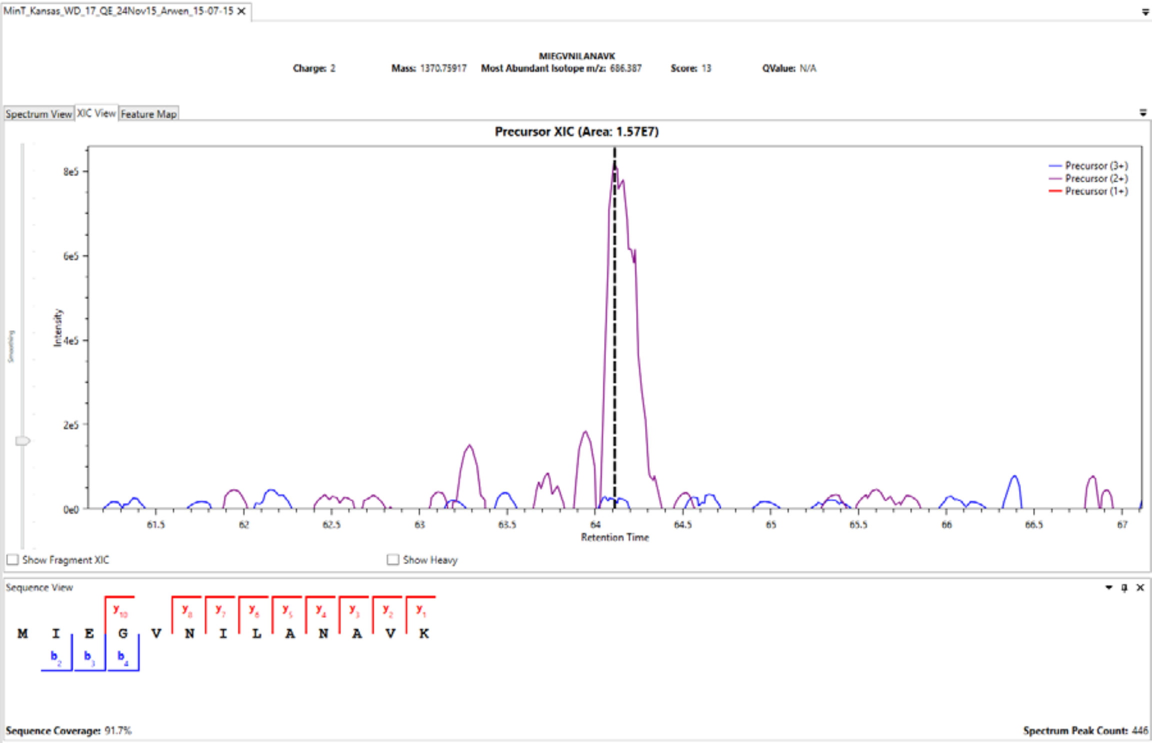
MinT_Kansas_WD_17_QE_24Nov15_Arwen_15-07-15
MIEGVNILANAVK
Charge: 2
Mass: 1370.75917
Most Abundant Isotope m/z: 686.387
Score: 13
QValue: N/A
Spectrum View
XIC View
Feature Map
Precursor XIC (Area: 1.57E7)
Precursor (3+)
Precursor (2+)
Precursor (1+)
Intensity
Retention Time
Show Fragment XIC
Show Heavy
Sequence View
M I E G V N I L A N A V K
Sequence Coverage: 91.7%
Spectrum Peak Count: 446

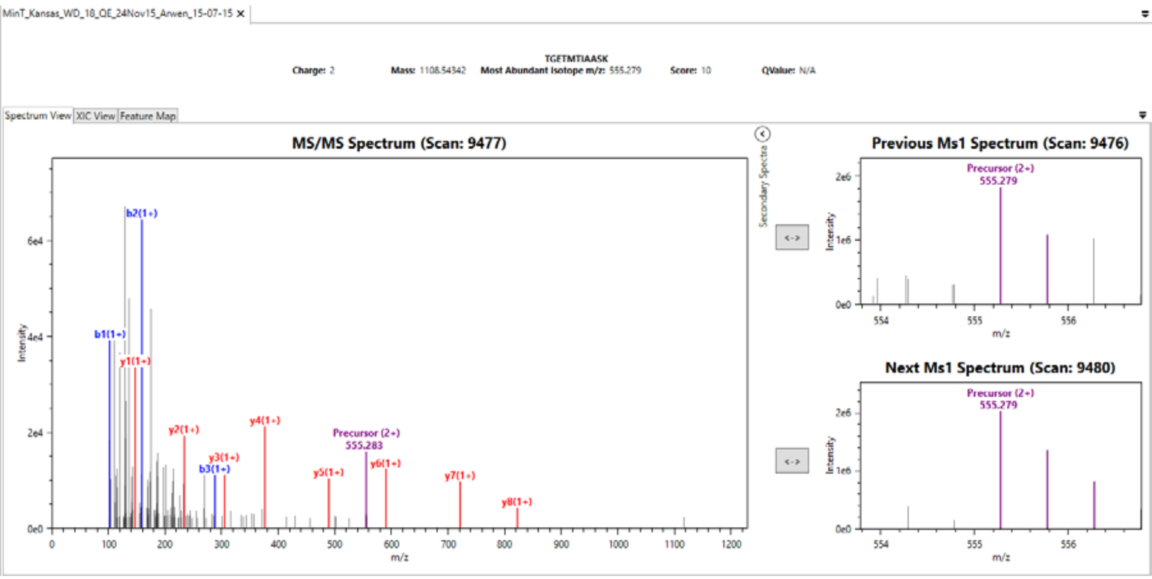
MinT_Kansas_WD_18_QE_24Nov15_Arwen_15-07-15
TGETMTIAASK
Charge: 2
Mass: 1108.54342
Most Abundant Isotope m/z: 555.279
Score: 10
QValue: N/A
Spectrum View
XIC View
Feature Map
MS/MS Spectrum (Scan: 9477)
Intensity
m/z
b1(1+)
b2(1+)
b3(1+)
y1(1+)
y2(1+)
y3(1+)
y4(1+)
y5(1+)
y6(1+)
y7(1+)
y8(1+)
Precursor (2+)
555.283
Secondary Spectra
Previous Ms1 Spectrum (Scan: 9476)
Precursor (2+)
555.279
Next Ms1 Spectrum (Scan: 9480)
Precursor (2+)
555.279

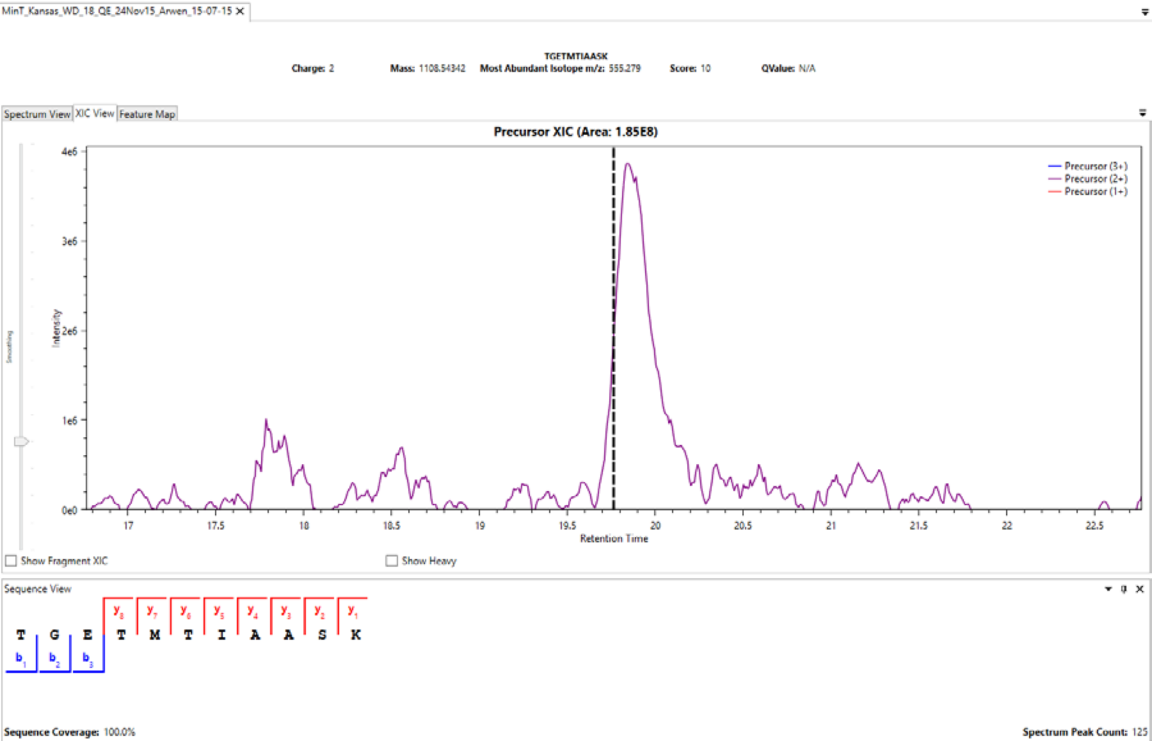
MinT_Kansas_WD_18_QE_24Nov15_Arwen_15-07-15
TGETMTIAASK
Charge: 2
Mass: 1108.54342
Most Abundant Isotope m/z: 555.279
Score: 10
QValue: N/A
Spectrum View
XIC View
Feature Map
Precursor XIC (Area: 1.85E8)
Intensity
Retention Time
Precursor (3+)
Precursor (2+)
Precursor (1+)
Show Fragment XIC
Show Heavy
Sequence View
T G E T M T I A A S K
Sequence Coverage: 100.0%
Spectrum Peak Count: 125

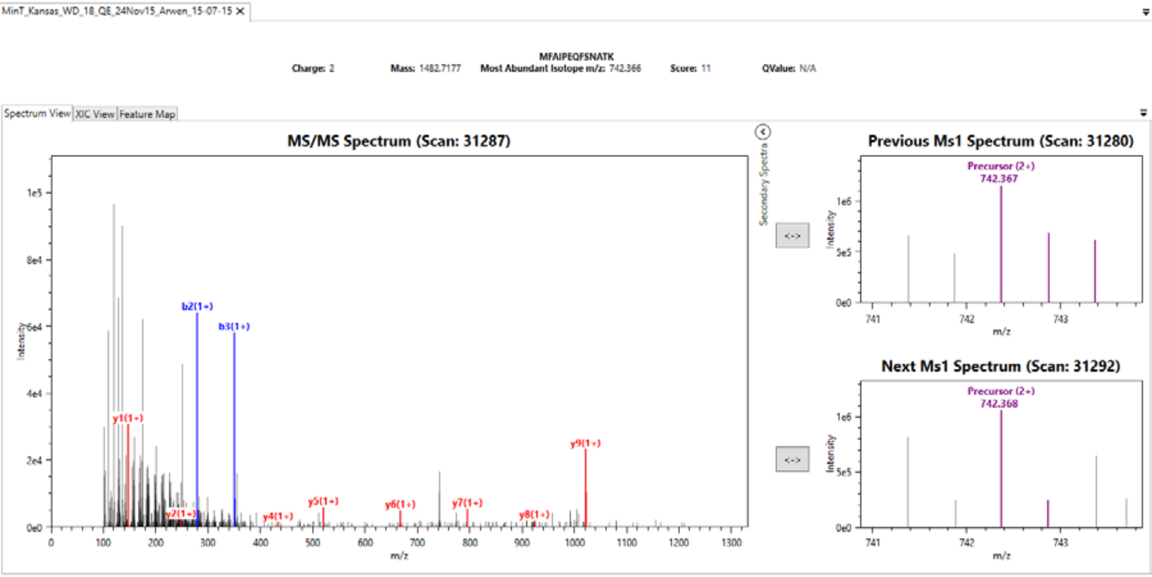
MinT_Kansas_WD_18_QE_24Nov15_Arwen_15-07-15
MFAIPEQFSNATK
Charge: 2
Mass: 1482.7177
Most Abundant Isotope m/z: 742.366
Score: 11
QValue: N/A
Spectrum View
XIC View
Feature Map
MS/MS Spectrum (Scan: 31287)
Intensity
m/z
b2(1+)
b3(1+)
y1(1+)
y2(1+)
y4(1+)
y5(1+)
y6(1+)
y7(1+)
y8(1+)
y9(1+)
Secondary Spectra
Previous Ms1 Spectrum (Scan: 31280)
Precursor (2+)
742.367
Next Ms1 Spectrum (Scan: 31292)
Precursor (2+)
742.368

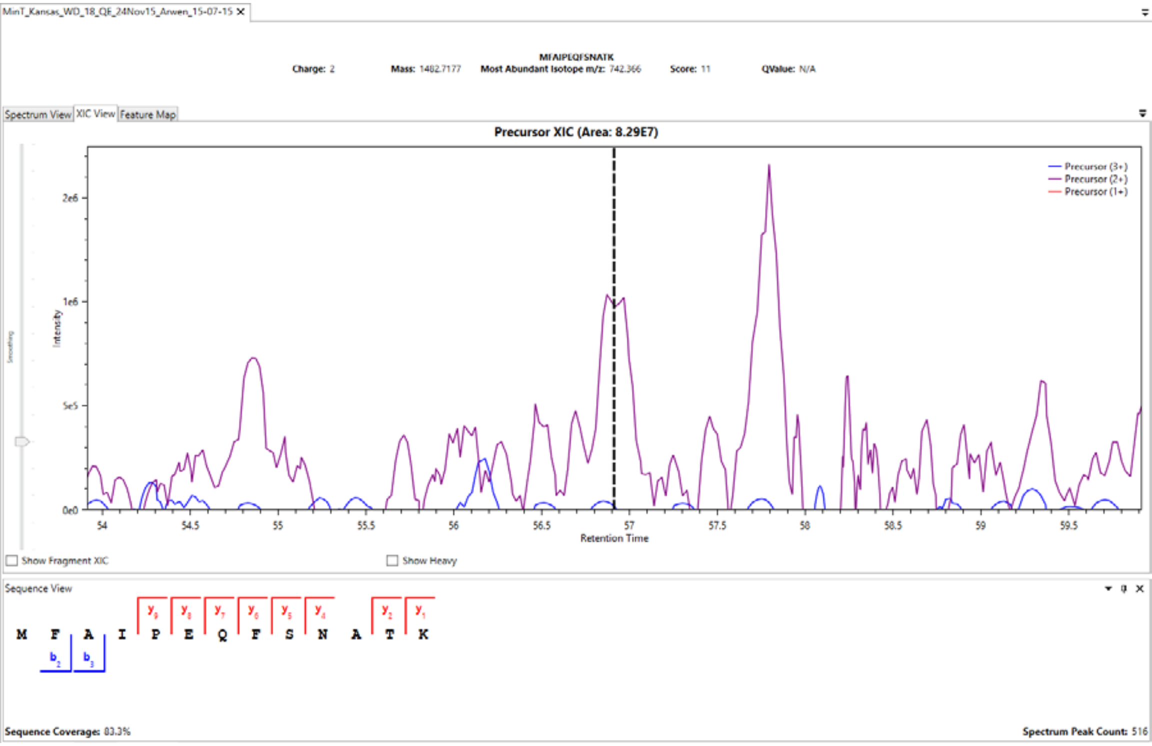
MinT_Kansas_WD_18_QE_24Nov15_Arwen_15-07-15
MFAIPEQFSNATK
Charge: 2
Mass: 1482.7177
Most Abundant Isotope m/z: 742.366
Score: 11
QValue: N/A
Spectrum View
XIC View
Feature Map
Precursor XIC (Area: 8.29E7)
Precursor (3+)
Precursor (2+)
Precursor (1+)
Intensity
Retention Time
Show Fragment XIC
Show Heavy
Sequence View
M F A I P E Q F S N A T K
Sequence Coverage: 83.3%
Spectrum Peak Count: 516

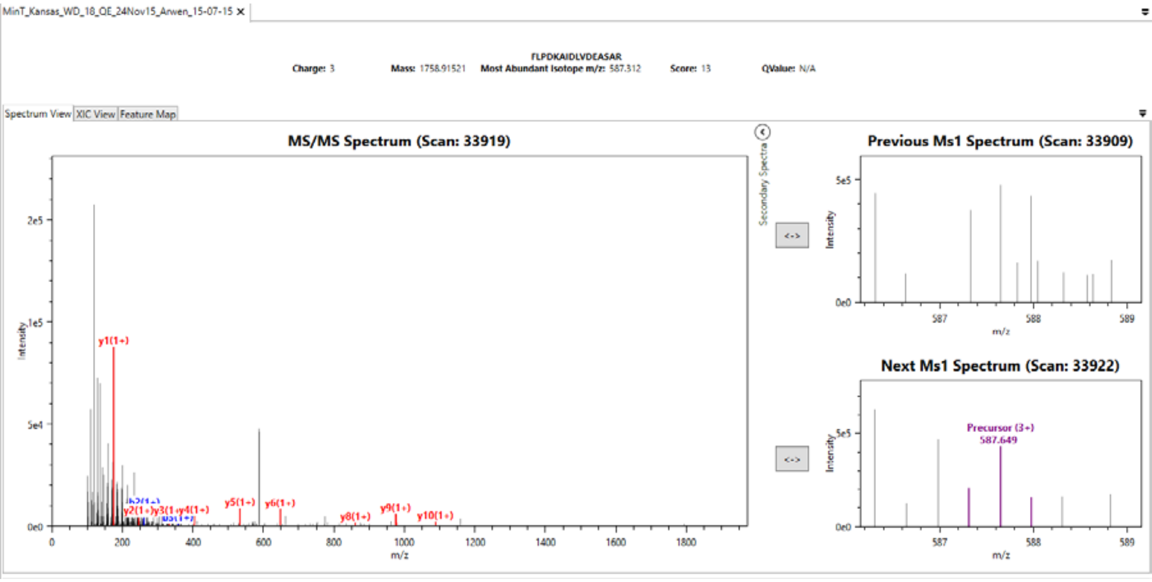
MinT_Kansas_WD_18_QE_24Nov15_Arwen_15-07-15
FLPDKAIDLVDEASAR
Charge: 3 Mass: 1758.91521 Most Abundant Isotope m/z: 587.312 Score: 13 QValue: N/A
Spectrum View XIC View Feature Map
MS/MS Spectrum (Scan: 33919)
Previous Ms1 Spectrum (Scan: 33909)
Next Ms1 Spectrum (Scan: 33922)
Precursor (3+) 587.649
Secondary Spectra
Intensity
m/z
y1(1+) y2(1+) y5(1+) y6(1+) y8(1+) y9(1+) y10(1+)

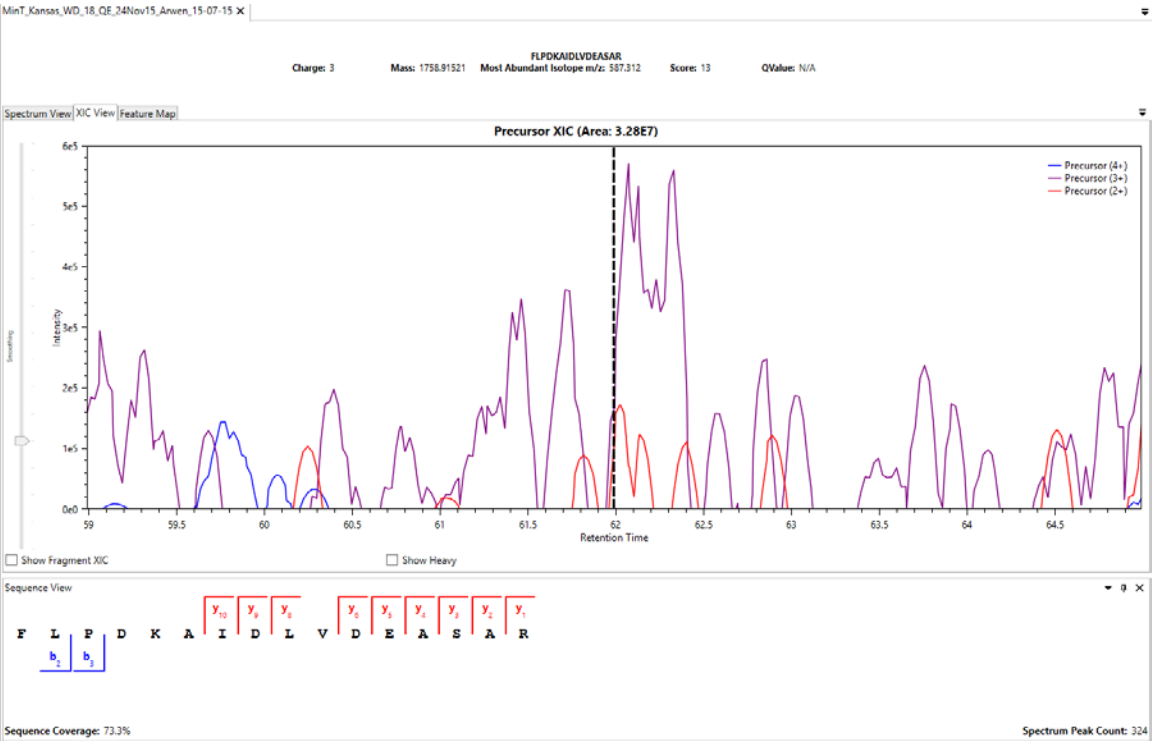
MinT_Kansas_WD_18_QE_24Nov15_Arwen_15-07-15
FLPDKAIDLVDEASAR
Charge: 3 Mass: 1758.91521 Most Abundant Isotope m/z: 587.312 Score: 13 QValue: N/A
Spectrum View XIC View Feature Map
Precursor XIC (Area: 3.28E7)
Precursor (4+)
Precursor (3+)
Precursor (2+)
Intensity
Retention Time
Show Fragment XIC
Show Heavy
Sequence View
F L P D K A I D L V D E A S A R
Sequence Coverage: 73.3%
Spectrum Peak Count: 324

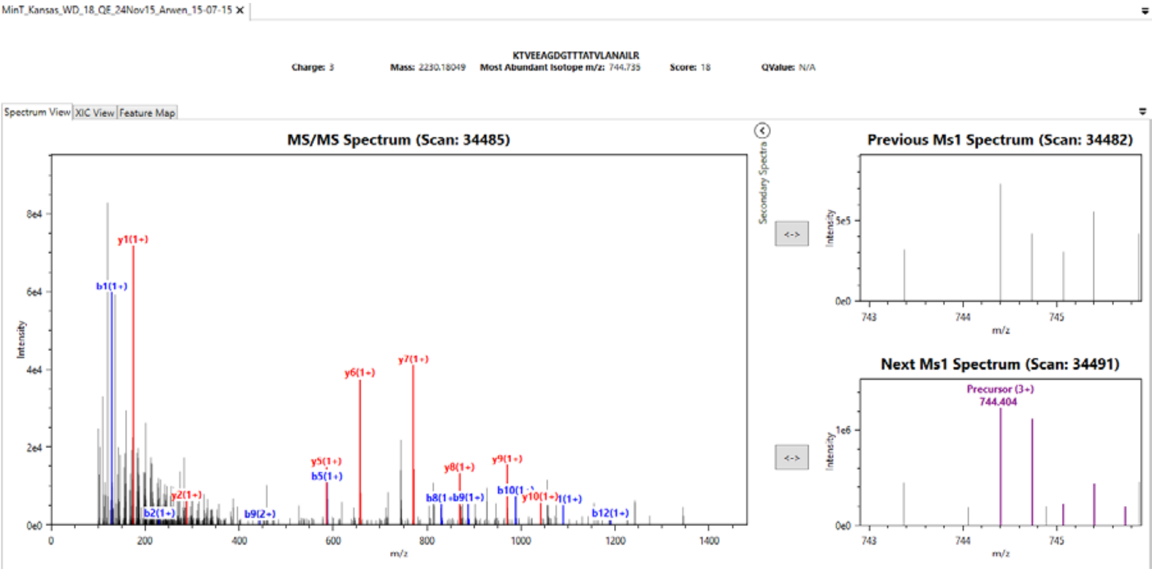
MinT_Kansas_WD_18_QE_24Nov15_Arwen_15-07-15
KTVEEAGDGTTTATVLANAILR
Charge: 3
Mass: 2230.18049
Most Abundant Isotope m/z: 744.735
Score: 18
QValue: N/A
Spectrum View
XIC View
Feature Map
MS/MS Spectrum (Scan: 34485)
Intensity
m/z
y1(1+)
b1(1+)
y6(1+)
y7(1+)
y5(1+)
b5(1+)
y8(1+)
y9(1+)
b8(1+)
b9(1+)
y10(1+)
b12(1+)
y2(1+)
b2(1+)
b9(2+)
Secondary Spectra
Previous Ms1 Spectrum (Scan: 34482)
Next Ms1 Spectrum (Scan: 34491)
Precursor (3+)
744.404

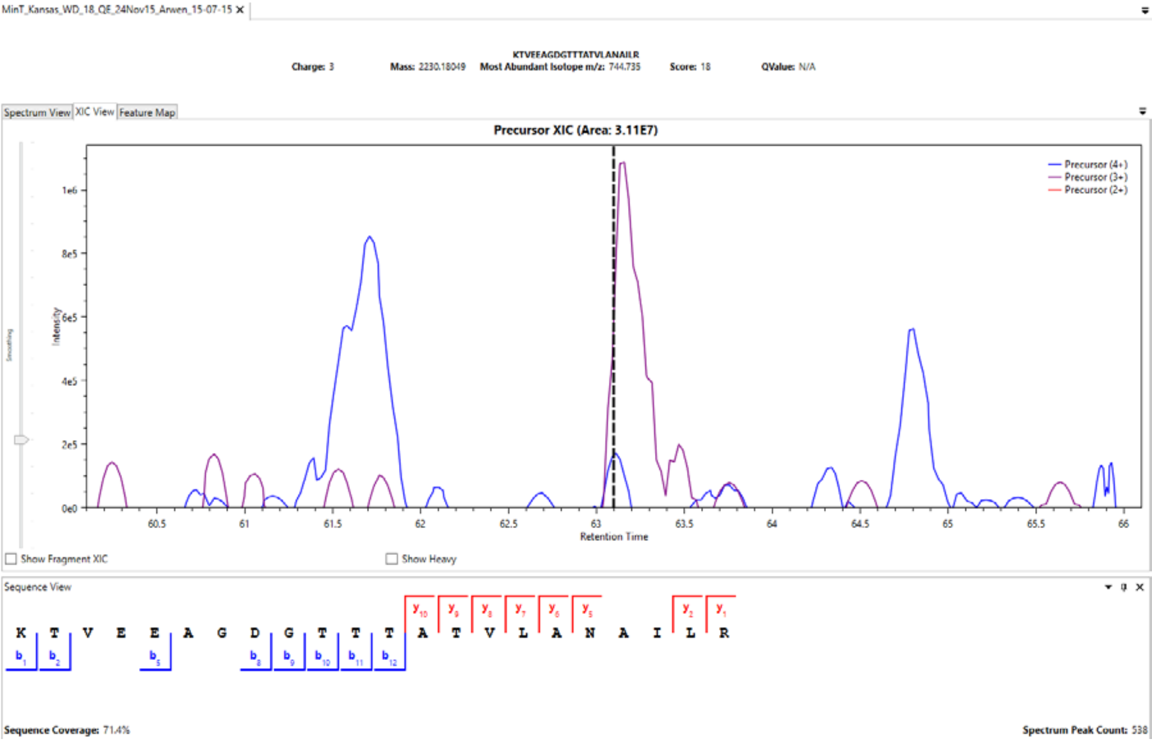
MinT_Kansas_WD_18_QE_24Nov15_Arwen_15-07-15
KTVEEAGDGTTTATVLANAILR
Charge: 3
Mass: 2230.18049
Most Abundant Isotope m/z: 744.735
Score: 18
QValue: N/A
Spectrum View
XIC View
Feature Map
Precursor XIC (Area: 3.11E7)
Intensity
Retention Time
Precursor (4+)
Precursor (3+)
Precursor (2+)
Show Fragment XIC
Show Heavy
Sequence View
K T V E E A G D G T T T A T V L A N A I L R
Sequence Coverage: 71.4%
Spectrum Peak Count: 538

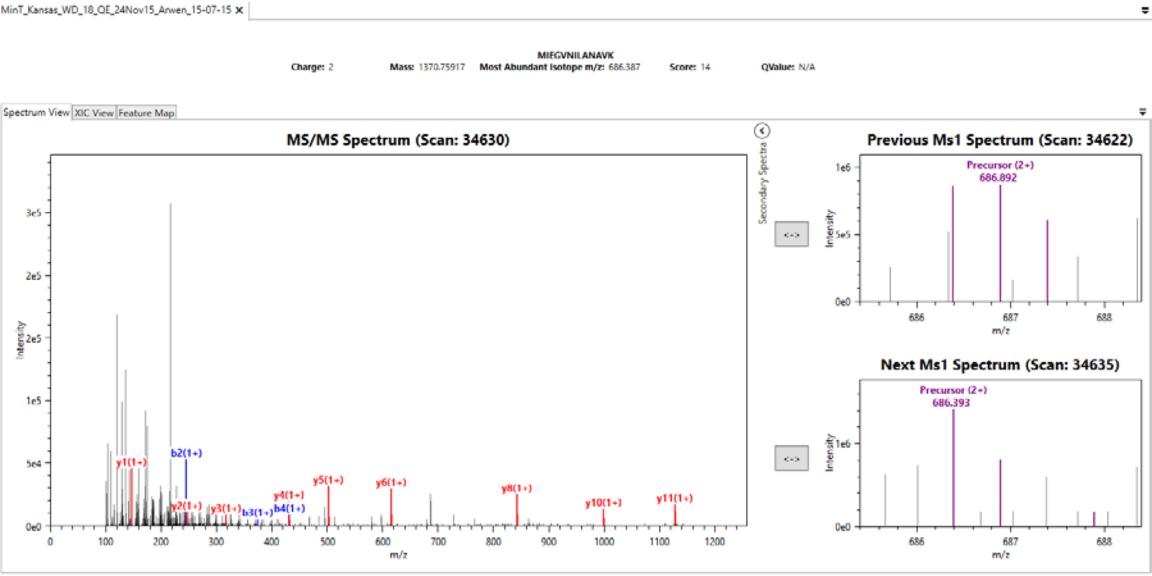
MinT_Kansas_WD_18_QE_24Nov15_Arwen_15-07-15
MIEGVNILANAVK
Charge: 2
Mass: 1370.75917
Most Abundant Isotope m/z: 686.387
Score: 14
QValue: N/A
Spectrum View
XIC View
Feature Map
MS/MS Spectrum (Scan: 34630)
Intensity
m/z
y1(1+)
b2(1+)
y2(1+)
y3(1+)
b3(1+)
b4(1+)
y4(1+)
y5(1+)
y6(1+)
y8(1+)
y10(1+)
y11(1+)
Secondary Spectra
Previous Ms1 Spectrum (Scan: 34622)
Precursor (2+)
686.892
Next Ms1 Spectrum (Scan: 34635)
Precursor (2+)
686.393

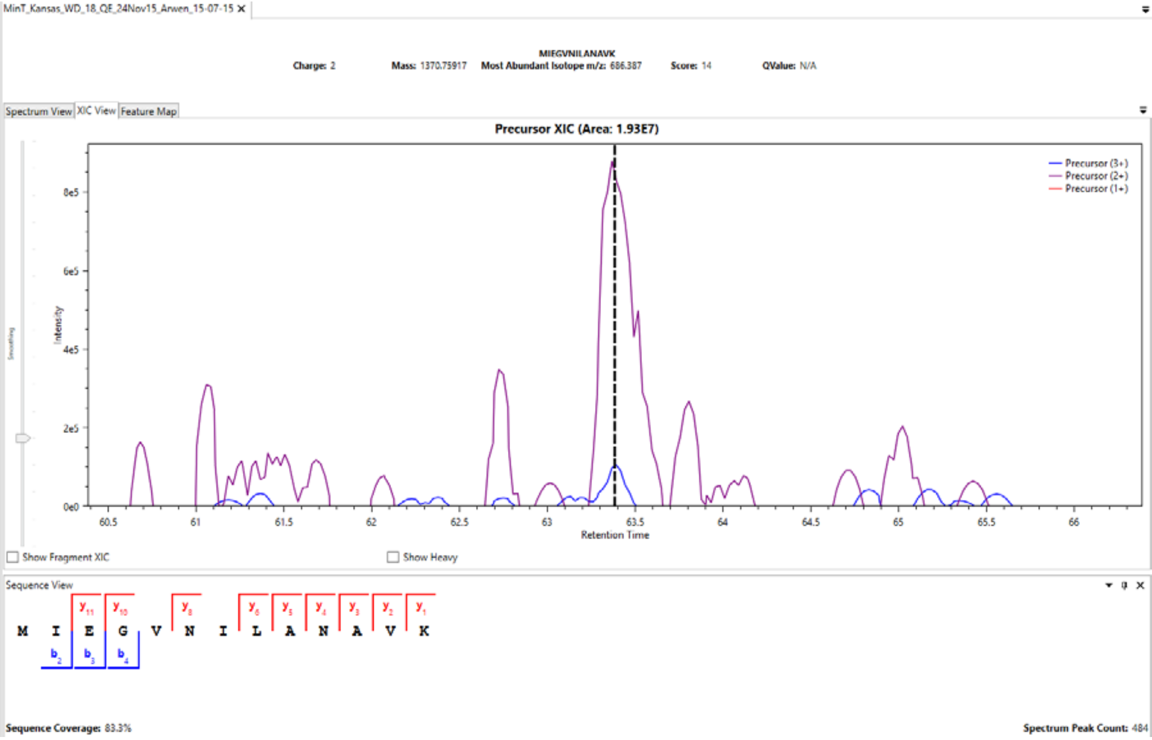
MinT_Kansas_WD_18_QE_24Nov15_Arwen_15-07-15
MIEGVNILANAVK
Charge: 2
Mass: 1370.75917
Most Abundant Isotope m/z: 686.387
Score: 14
QValue: N/A
Spectrum View
XIC View
Feature Map
Precursor XIC (Area: 1.93E7)
Intensity
Retention Time
Precursor (3+)
Precursor (2+)
Precursor (1+)
Show Fragment XIC
Show Heavy
Sequence View
M I E G V N I L A N A V K
Sequence Coverage: 83.3%
Spectrum Peak Count: 484

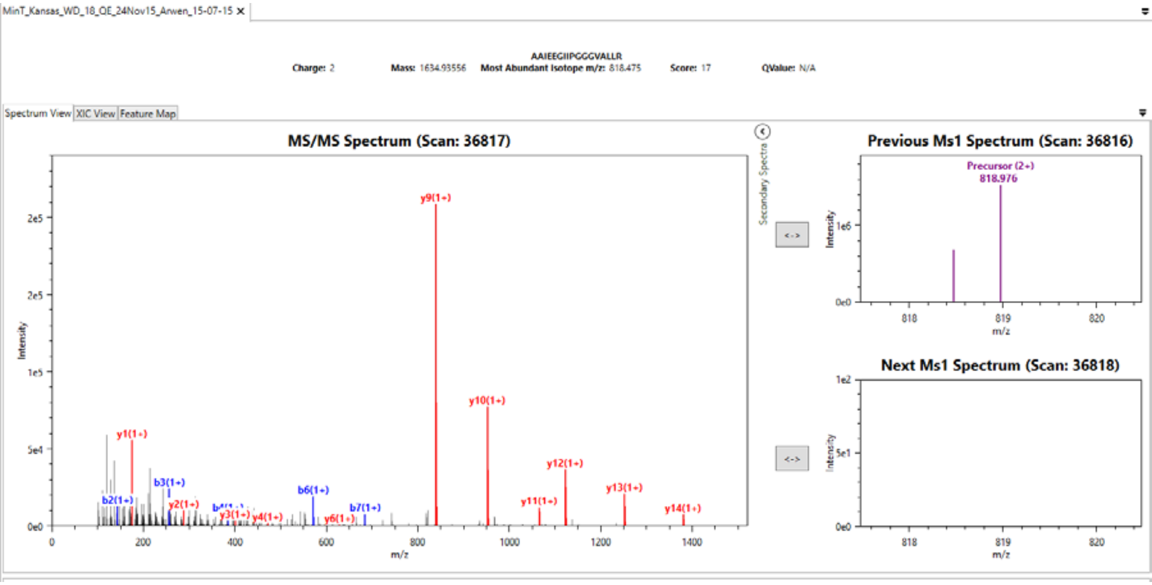
MinT_Kansas_WD_18_QE_24Nov15_Arwen_15-07-15
AAIEEGIIPGGGVALLR
Charge: 2
Mass: 1634.93556
Most Abundant Isotope m/z: 818.475
Score: 17
QValue: N/A
Spectrum View
XIC View
Feature Map
MS/MS Spectrum (Scan: 36817)
Intensity
m/z
y9(1+)
y10(1+)
y1(1+)
y12(1+)
y11(1+)
y13(1+)
y14(1+)
b2(1+)
b3(1+)
y2(1+)
y3(1+)
y4(1+)
b6(1+)
b7(1+)
y6(1+)
Secondary Spectra
Previous Ms1 Spectrum (Scan: 36816)
Precursor (2+)
818.976
Next Ms1 Spectrum (Scan: 36818)

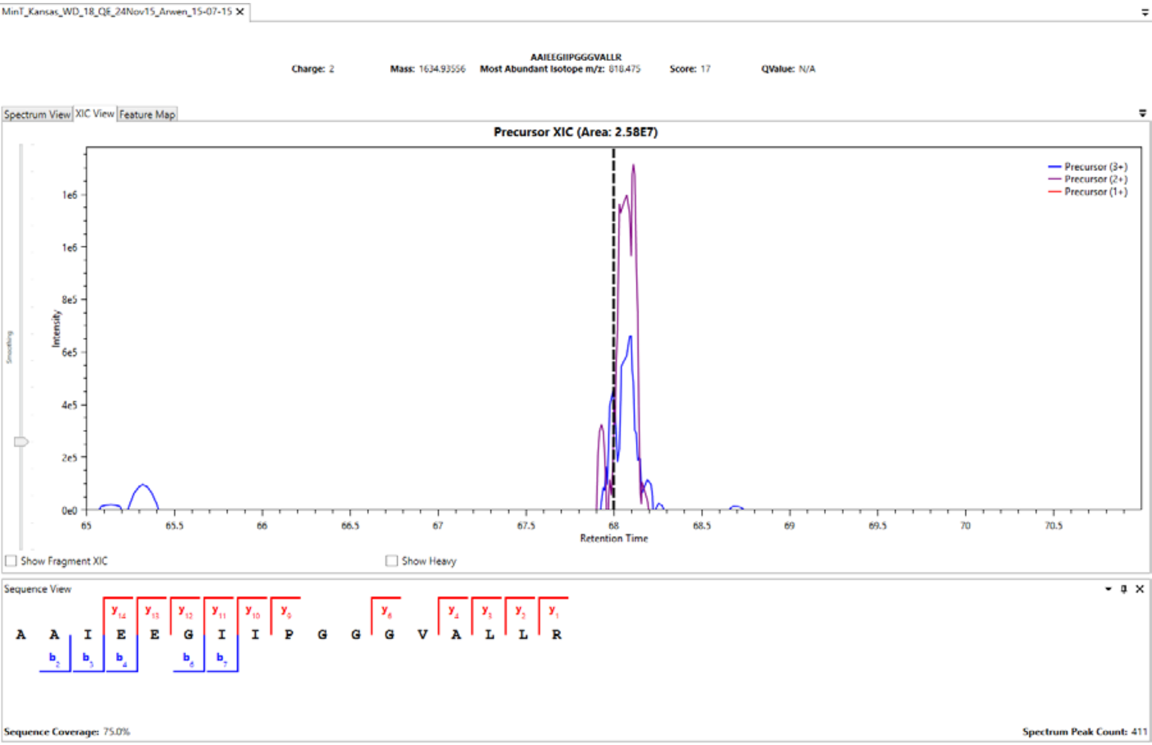
MinT_Kansas_WD_18_QE_24Nov15_Arwen_15-07-15
AAIEEGIIPGGGVALLR
Charge: 2
Mass: 1634.93556
Most Abundant Isotope m/z: 818.475
Score: 17
QValue: N/A
Spectrum View
XIC View
Feature Map
Precursor XIC (Area: 2.58E7)
Precursor (3+)
Precursor (2+)
Precursor (1+)
Intensity
Retention Time
Smoothing
Show Fragment XIC
Show Heavy
Sequence View
A A I E E G I I P G G G V A L L R
Sequence Coverage: 75.0%
Spectrum Peak Count: 411

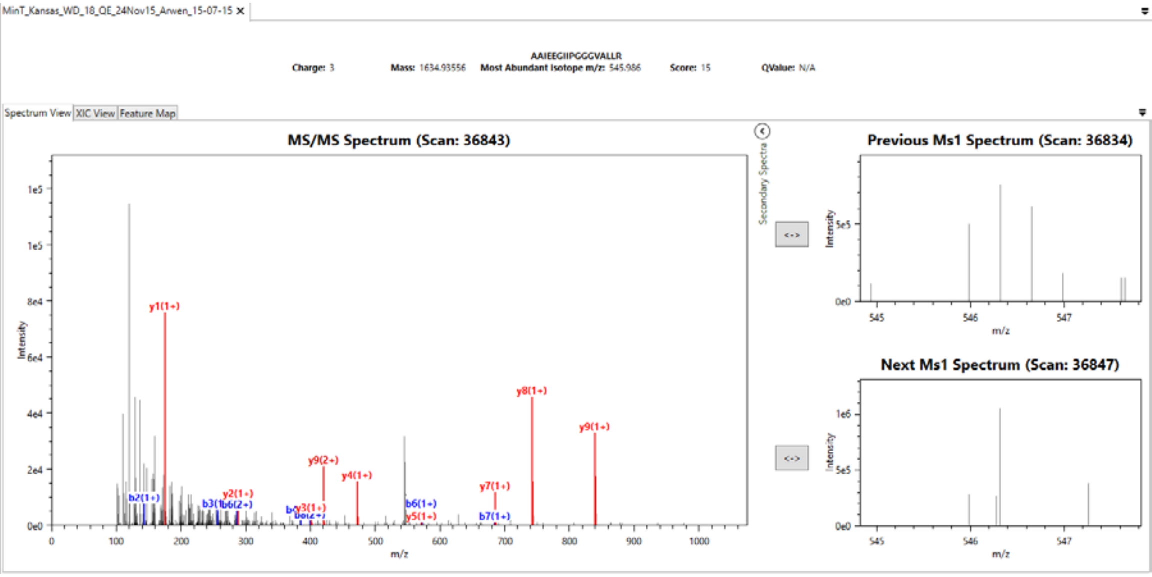
MinT_Kansas_WD_18_QE_24Nov15_Arwen_15-07-15
AAIEEGIIPGGGVALLR
Charge: 3
Mass: 1634.93556
Most Abundant Isotope m/z: 545.986
Score: 15
QValue: N/A
Spectrum View
XIC View
Feature Map
MS/MS Spectrum (Scan: 36843)
Intensity
m/z
y1(1+)
y8(1+)
y9(1+)
y9(2+)
y4(1+)
y7(1+)
y2(1+)
b2(1+)
b6(1+)
y5(1+)
b7(1+)
Secondary Spectra
Previous Ms1 Spectrum (Scan: 36834)
Next Ms1 Spectrum (Scan: 36847)

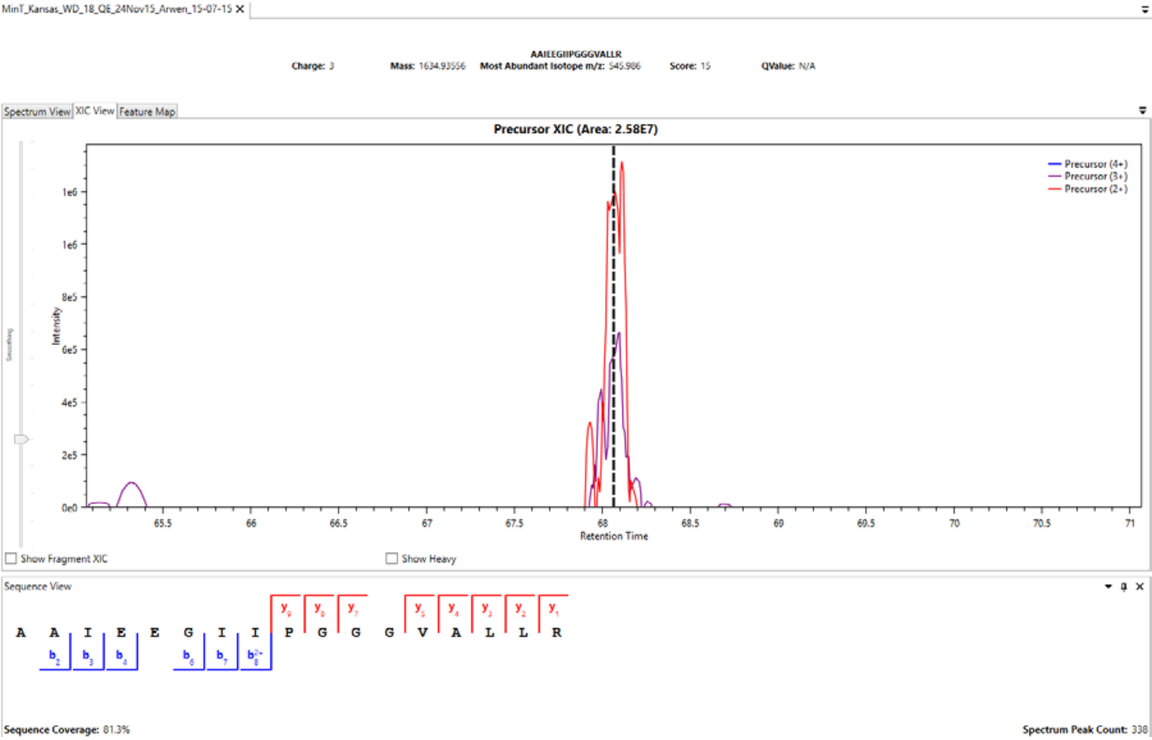
MinT_Kansas_WD_18_QE_24Nov15_Arwen_15-07-15
AAIEEGIIPGGGVALLR
Charge: 3
Mass: 1634.93556
Most Abundant Isotope m/z: 545.986
Score: 15
QValue: N/A
Spectrum View
XIC View
Feature Map
Precursor XIC (Area: 2.58E7)
Precursor (4+)
Precursor (3+)
Precursor (2+)
Intensity
Retention Time
Show Fragment XIC
Show Heavy
Sequence View
A A I E E G I I P G G G V A L L R
Sequence Coverage: 81.3%
Spectrum Peak Count: 338

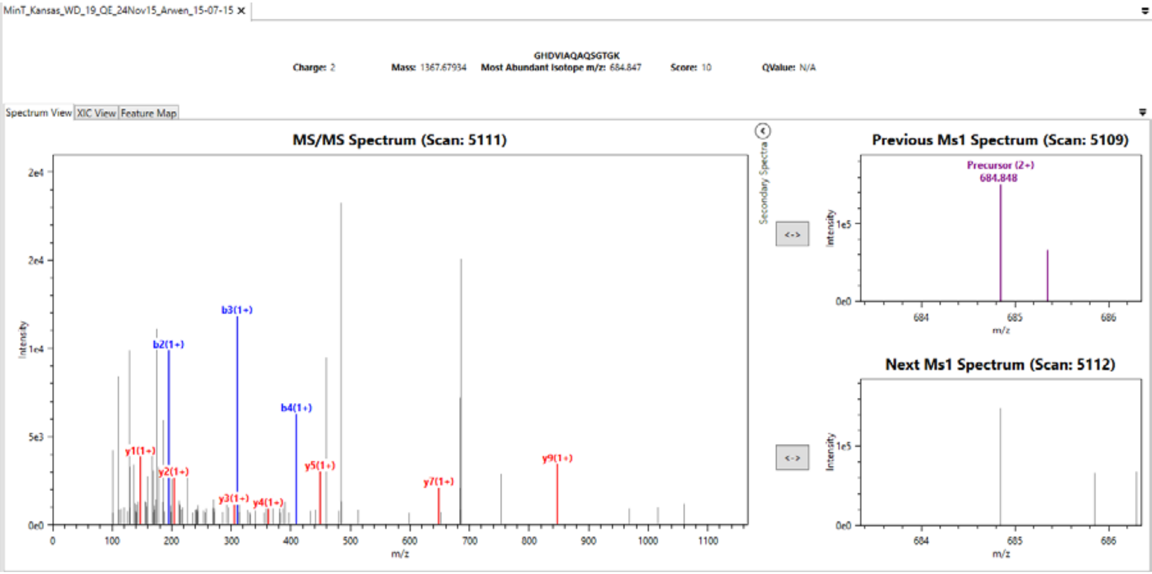
MinT_Kansas_WD_19_QE_24Nov15_Arwen_15-07-15
GHDVIAQAQSGTGK
Charge: 2
Mass: 1367.67934
Most Abundant Isotope m/z: 684.847
Score: 10
QValue: N/A
Spectrum View
XIC View
Feature Map
MS/MS Spectrum (Scan: 5111)
Intensity
m/z
b2(1+)
b3(1+)
b4(1+)
y1(1+)
y2(1+)
y3(1+)
y4(1+)
y5(1+)
y7(1+)
y9(1+)
Secondary Spectra
Previous Ms1 Spectrum (Scan: 5109)
Precursor (2+)
684.848
Next Ms1 Spectrum (Scan: 5112)

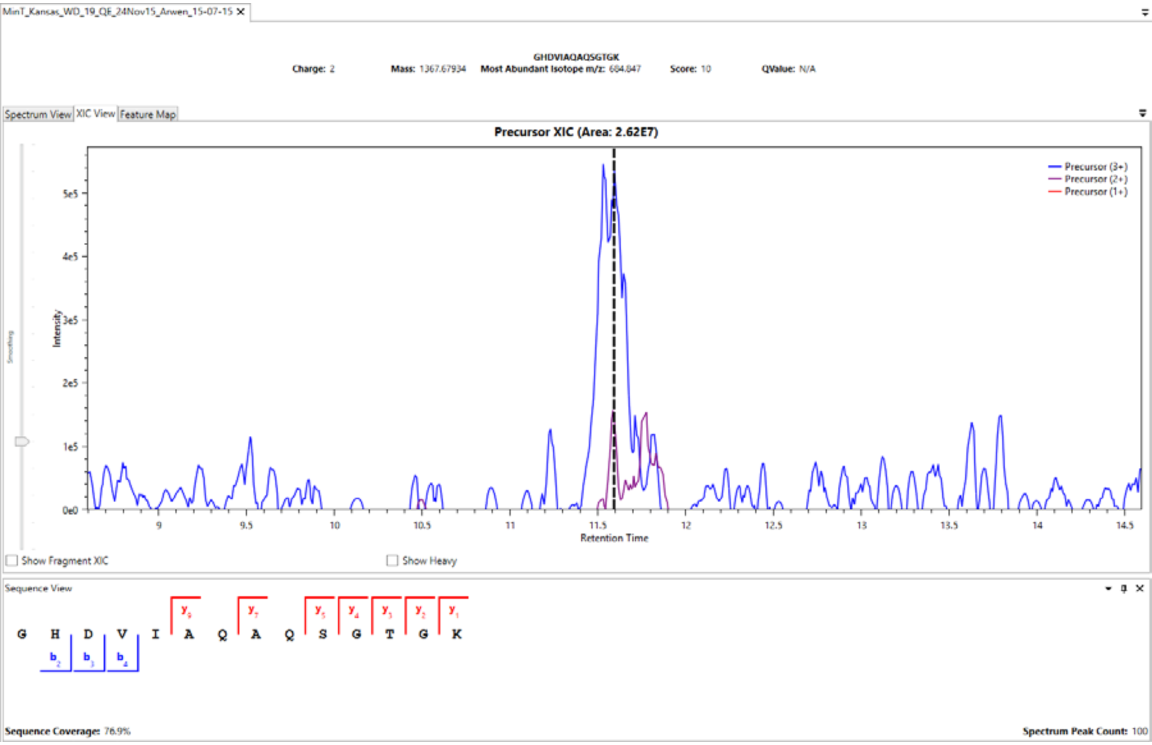
MinT_Kansas_WD_19_QE_24Nov15_Arwen_15-07-15
GHDVIAQAQSGTGK
Charge: 2
Mass: 1367.67934
Most Abundant Isotope m/z: 684.847
Score: 10
QValue: N/A
Spectrum View
XIC View
Feature Map
Precursor XIC (Area: 2.62E7)
Precursor (3+)
Precursor (2+)
Precursor (1+)
Intensity
Retention Time
Show Fragment XIC
Show Heavy
Sequence View
G H D V I A Q A Q S G T G K
Sequence Coverage: 76.9%
Spectrum Peak Count: 100

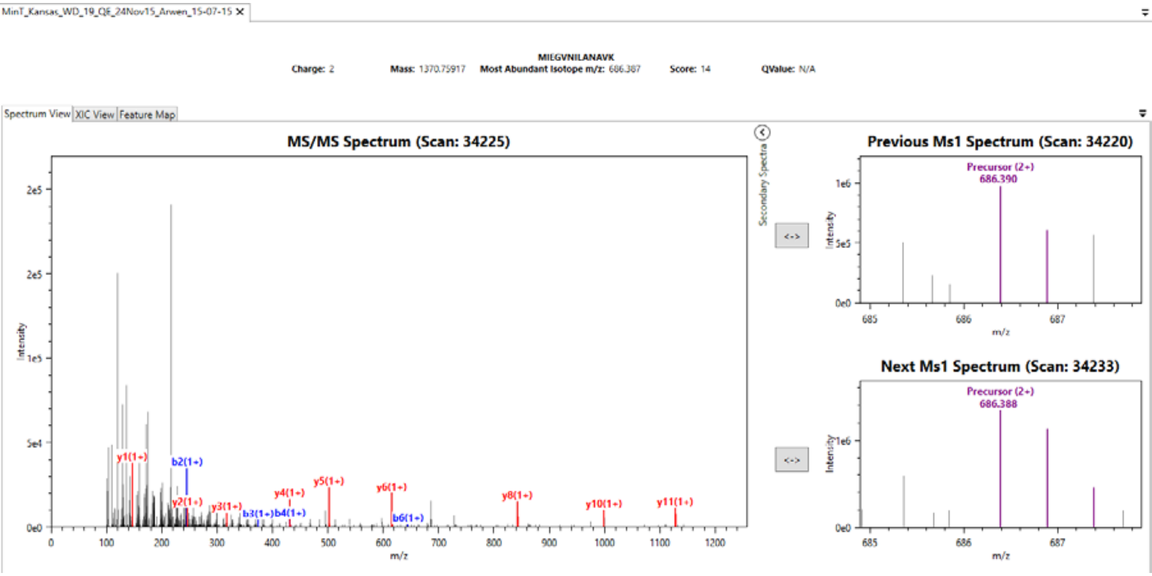
MinT_Kansas_WD_19_QE_24Nov15_Arwen_15-07-15
MIEGVNILANAVK
Charge: 2
Mass: 1370.75917
Most Abundant Isotope m/z: 686.387
Score: 14
QValue: N/A
Spectrum View
XIC View
Feature Map
MS/MS Spectrum (Scan: 34225)
Intensity
m/z
y1(1+) b2(1+) y2(1+) y3(1+) b3(1+) b4(1+) y4(1+) y5(1+) y6(1+) b6(1+) y8(1+) y10(1+) y11(1+)
Secondary Spectra
Previous Ms1 Spectrum (Scan: 34220)
Precursor (2+)
686.390
Next Ms1 Spectrum (Scan: 34233)
Precursor (2+)
686.388

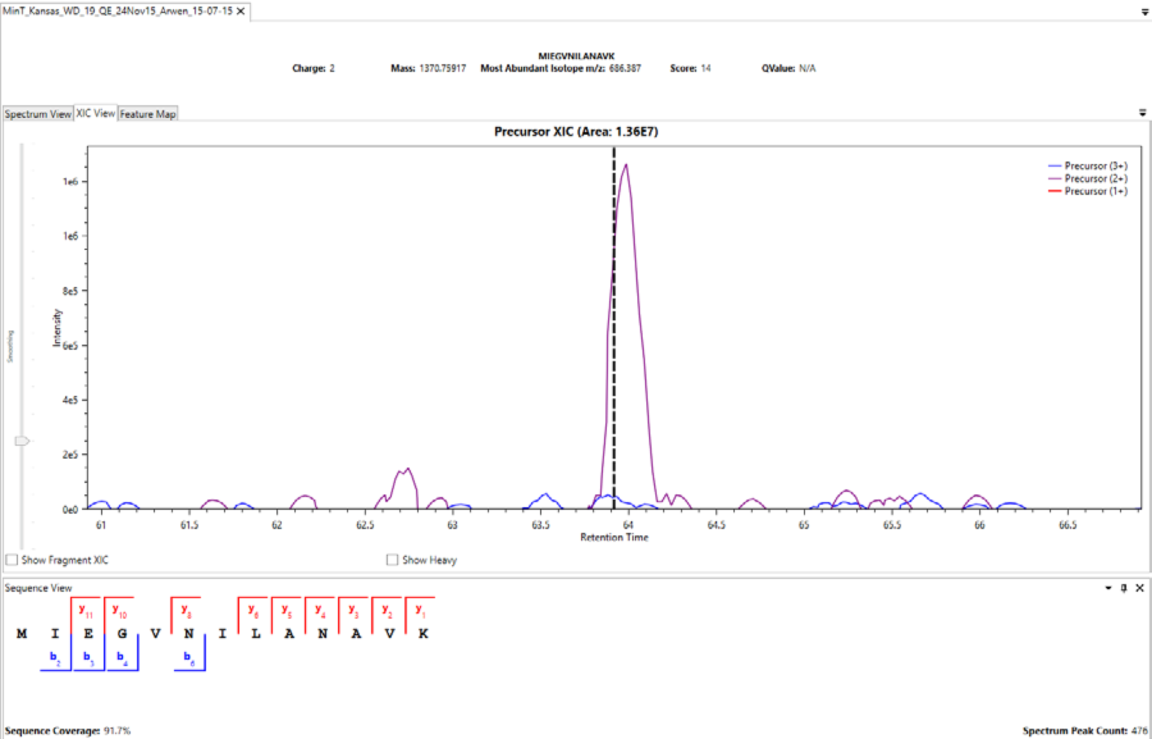
MinT_Kansas_WD_19_QE_24Nov15_Arwen_15-07-15
MIEGVNILANAVK
Charge: 2
Mass: 1370.75917
Most Abundant Isotope m/z: 686.387
Score: 14
QValue: N/A
Spectrum View
XIC View
Feature Map
Precursor XIC (Area: 1.36E7)
Intensity
Retention Time
Precursor (3+)
Precursor (2+)
Precursor (1+)
Show Fragment XIC
Show Heavy
Sequence View
M I E G V N I L A N A V K
Sequence Coverage: 91.7%
Spectrum Peak Count: 476

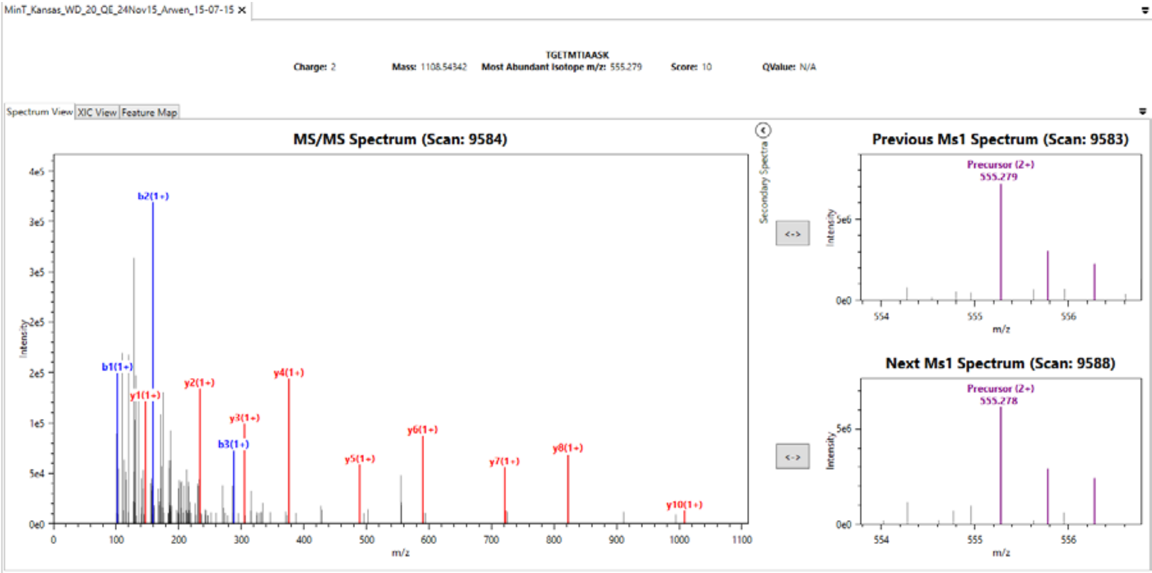
MinT_Kansas_WD_20_QE_24Nov15_Arwen_15-07-15
TGETMTIAASK
Charge: 2
Mass: 1108.54342
Most Abundant Isotope m/z: 555.279
Score: 10
QValue: N/A
Spectrum View
XIC View
Feature Map
MS/MS Spectrum (Scan: 9584)
Intensity
m/z
b1(1+)
b2(1+)
b3(1+)
y1(1+)
y2(1+)
y3(1+)
y4(1+)
y5(1+)
y6(1+)
y7(1+)
y8(1+)
y10(1+)
Secondary Spectra
Previous Ms1 Spectrum (Scan: 9583)
Precursor (2+)
555.279
Next Ms1 Spectrum (Scan: 9588)
Precursor (2+)
555.278

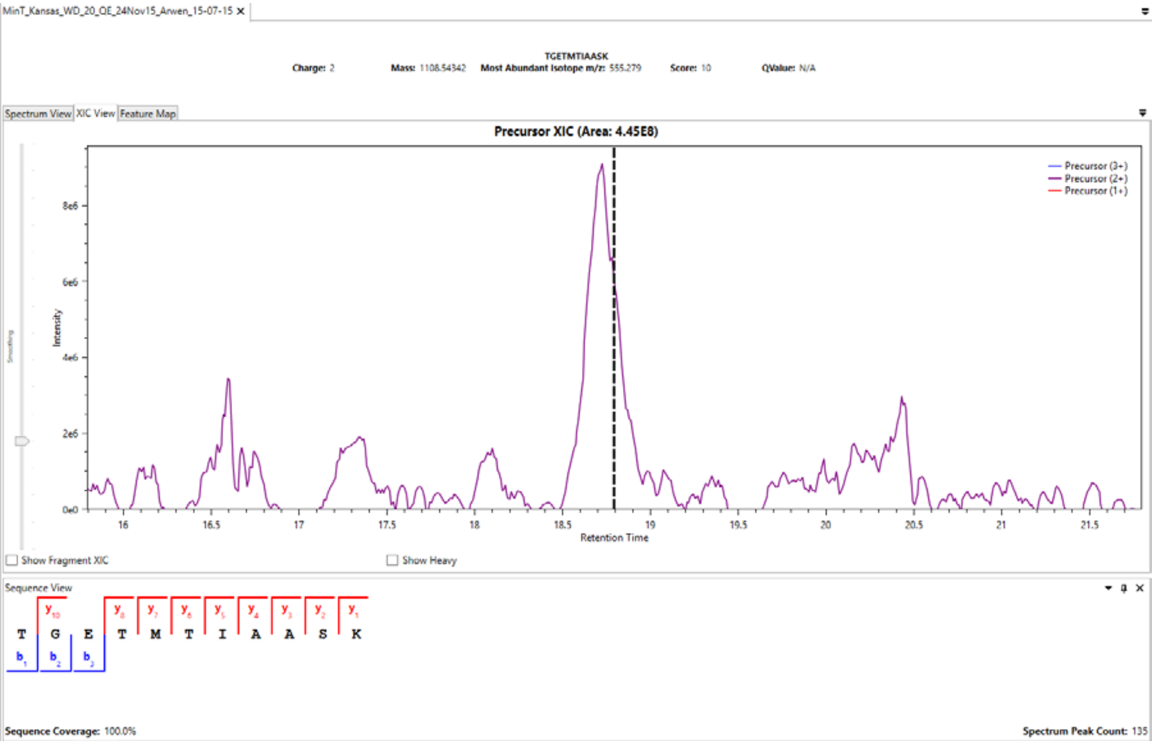
MinT_Kansas_WD_20_QE_24Nov15_Arwen_15-07-15
TGETMTIAASK
Charge: 2
Mass: 1108.54342
Most Abundant Isotope m/z: 555.279
Score: 10
QValue: N/A
Spectrum View
XIC View
Feature Map
Precursor XIC (Area: 4.45E8)
Precursor (3+)
Precursor (2+)
Precursor (1+)
Intensity
Retention Time
Smoothing
Show Fragment XIC
Show Heavy
Sequence View
T G E T M T I A A S K
Sequence Coverage: 100.0%
Spectrum Peak Count: 135

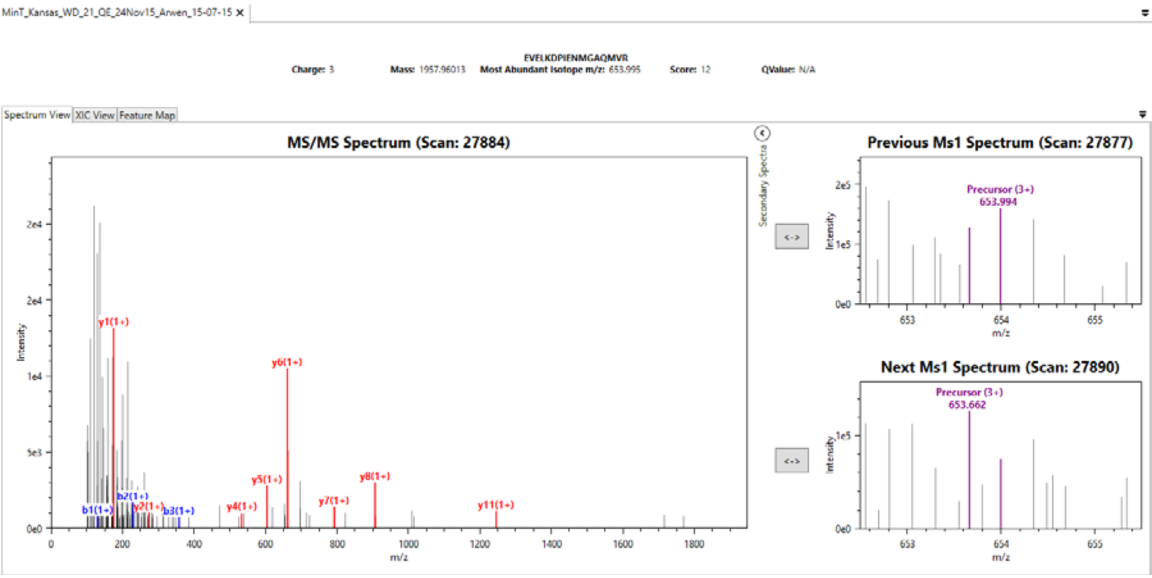
MinT_Kansas_WD_21_QE_24Nov15_Arwen_15-07-15
EVELKDPIENMGAQMVR
Charge: 3
Mass: 1957.96013
Most Abundant Isotope m/z: 653.995
Score: 12
QValue: N/A
Spectrum View
XIC View
Feature Map
MS/MS Spectrum (Scan: 27884)
Intensity
m/z
y1(1+)
y6(1+)
y5(1+)
y8(1+)
y7(1+)
y11(1+)
y4(1+)
y2(1+)
b1(1+)
b2(1+)
b3(1+)
Secondary Spectra
Previous Ms1 Spectrum (Scan: 27877)
Precursor (3+)
653.994
Next Ms1 Spectrum (Scan: 27890)
Precursor (3+)
653.662

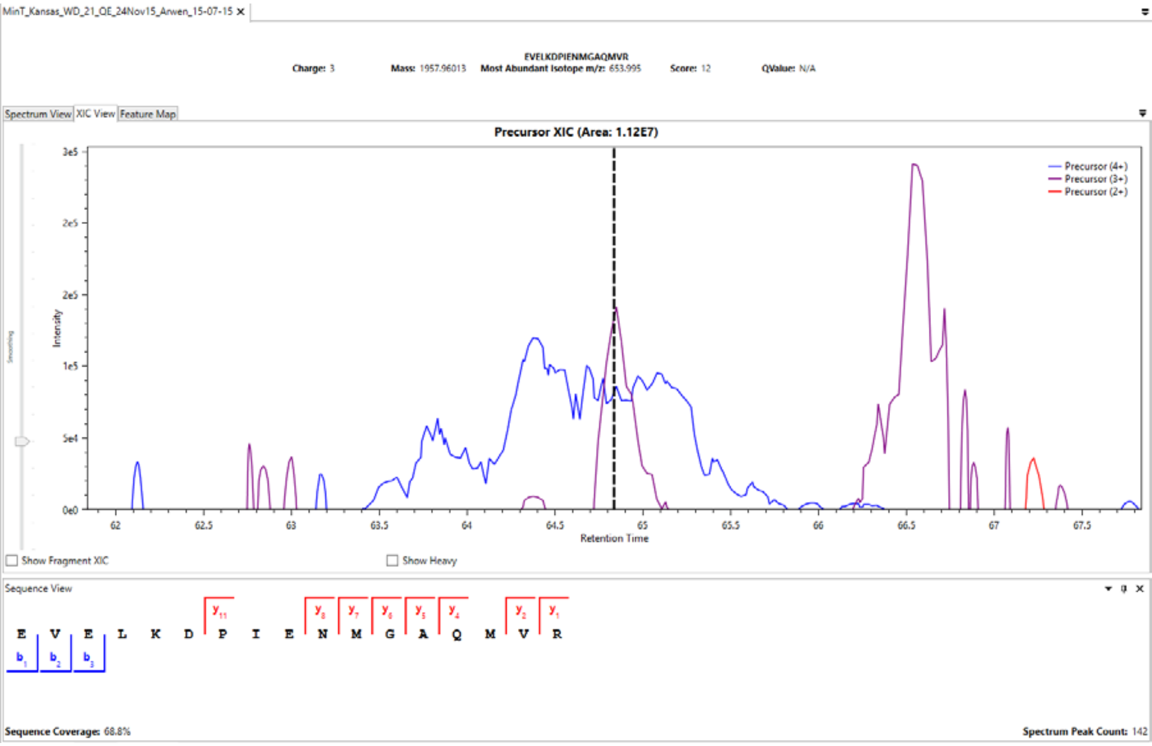
MinT_Kansas_WD_21_QE_24Nov15_Arwen_15-07-15
EVELKDPIENMGAQMVR
Charge: 3
Mass: 1957.96013
Most Abundant Isotope m/z: 653.995
Score: 12
QValue: N/A
Spectrum View
XIC View
Feature Map
Precursor XIC (Area: 1.12E7)
Intensity
Retention Time
Precursor (4+)
Precursor (3+)
Precursor (2+)
Smoothing
Show Fragment XIC
Show Heavy
Sequence View
E V E L K D P I E N M G A Q M V R
Sequence Coverage: 68.8%
Spectrum Peak Count: 142

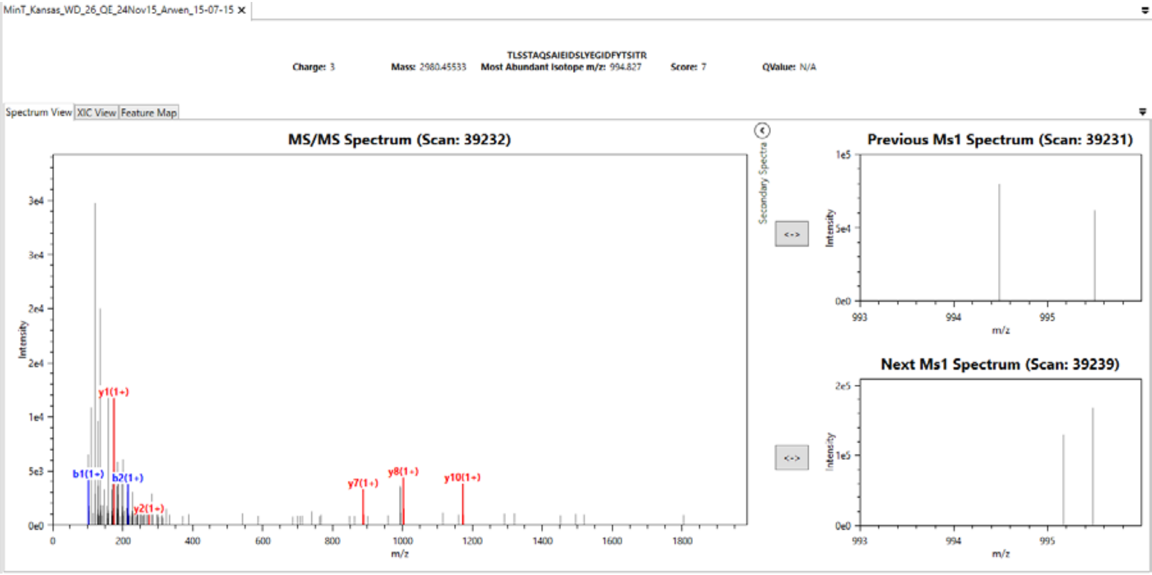
MinT_Kansas_WD_26_QE_24Nov15_Arwen_15-07-15
TLSSTAQSAIEIDSLYEGIDFYTSITR
Charge: 3
Mass: 2980.45533
Most Abundant Isotope m/z: 994.827
Score: 7
QValue: N/A
Spectrum View
XIC View
Feature Map
MS/MS Spectrum (Scan: 39232)
Intensity
m/z
b1(1+)
b2(1+)
y1(1+)
y2(1+)
y7(1+)
y8(1+)
y10(1+)
Secondary Spectra
Previous Ms1 Spectrum (Scan: 39231)
Next Ms1 Spectrum (Scan: 39239)

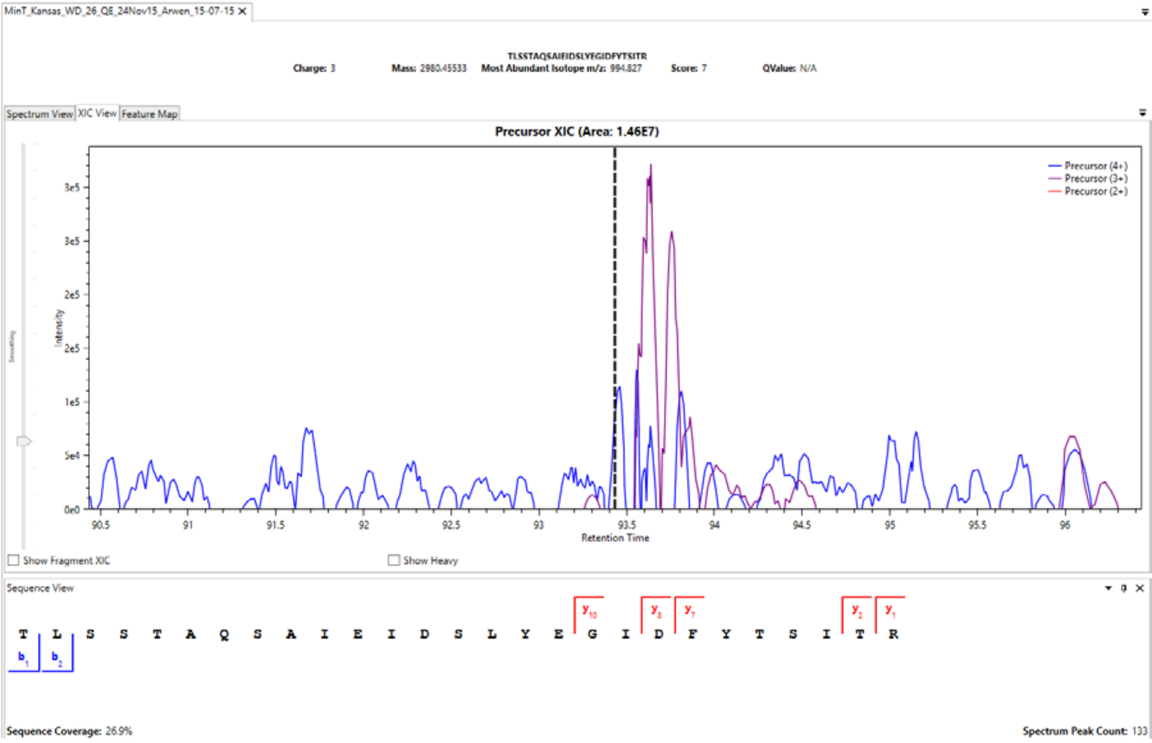
MinT_Kansas_WD_26_QE_24Nov15_Arwen_15-07-15
TLSSTAQSAIEIDSLYEGIDFYTSITR
Charge: 3
Mass: 2980.45533
Most Abundant Isotope m/z: 994.827
Score: 7
QValue: N/A
Spectrum View
XIC View
Feature Map
Precursor XIC (Area: 1.46E7)
Precursor (4+)
Precursor (3+)
Precursor (2+)
Intensity
Retention Time
Show Fragment XIC
Show Heavy
Sequence View
T L S S T A Q S A I E I D S L Y E G I D F Y T S I T R
Sequence Coverage: 26.9%
Spectrum Peak Count: 133

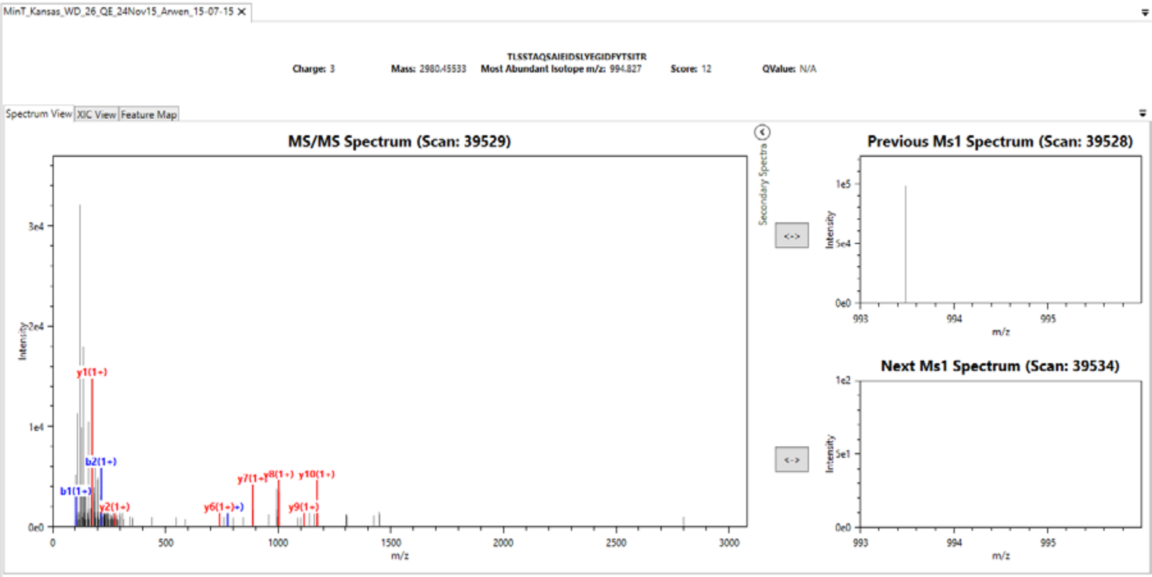
MinT_Kansas_WD_26_QE_24Nov15_Arwen_15-07-15
TLSSTAQSAIEIDSLYEGIDFYTSITR
Charge: 3
Mass: 2980.45533
Most Abundant Isotope m/z: 994.827
Score: 12
QValue: N/A
Spectrum View
XIC View
Feature Map
MS/MS Spectrum (Scan: 39529)
Intensity
m/z
Previous Ms1 Spectrum (Scan: 39528)
Next Ms1 Spectrum (Scan: 39534)
Secondary Spectra

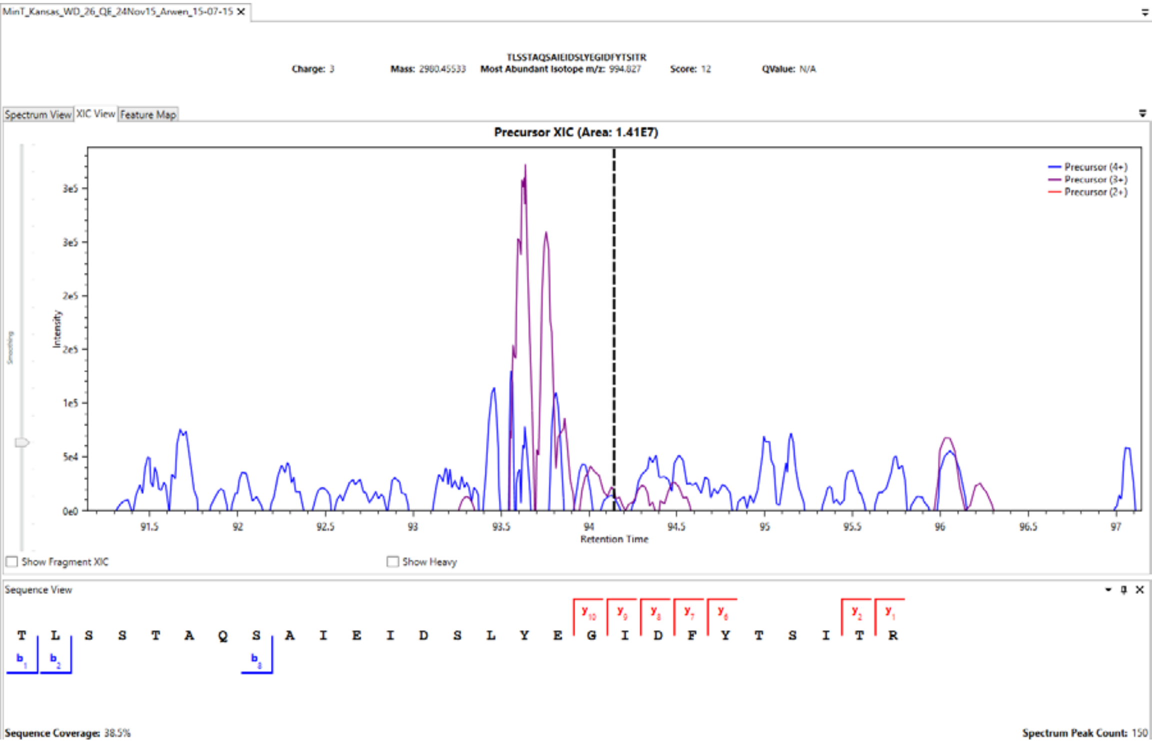
MinT_Kansas_WD_26_QE_24Nov15_Arwen_15-07-15
TLSSTAQSAIEIDSLYEGIDFYTSITR
Charge: 3
Mass: 2980.45533
Most Abundant Isotope m/z: 994.827
Score: 12
QValue: N/A
Spectrum View
XIC View
Feature Map
Precursor XIC (Area: 1.41E7)
Precursor (4+)
Precursor (3+)
Precursor (2+)
Intensity
Retention Time
Show Fragment XIC
Show Heavy
Sequence View
T L S S T A Q S A I E I D S L Y E G I D F Y T S I T R
Sequence Coverage: 38.5%
Spectrum Peak Count: 150

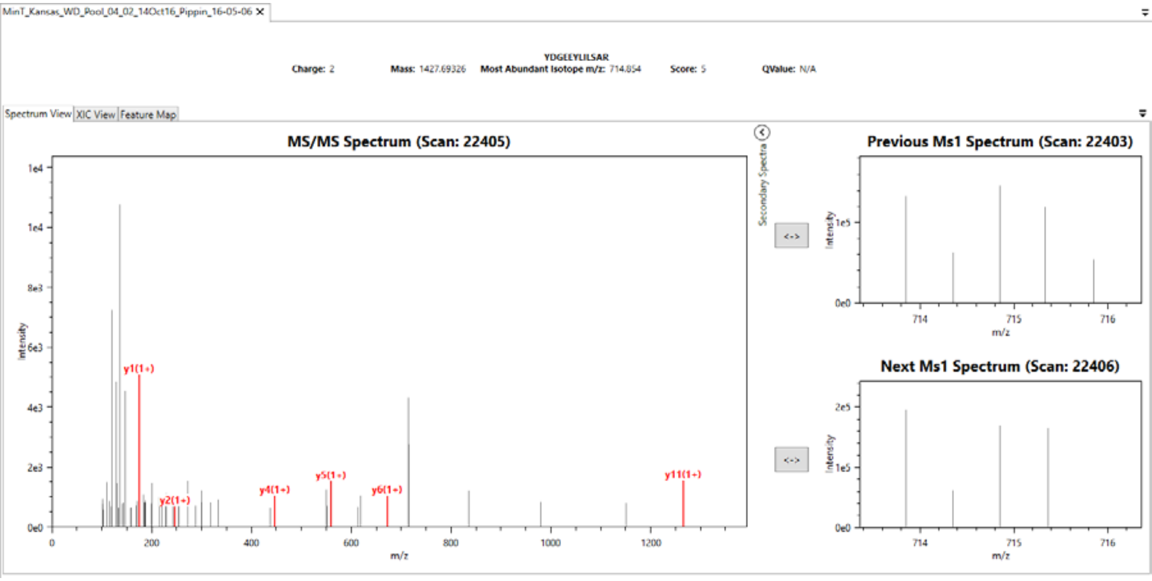
MinT_Kansas_WD_Pool_04_02_14Oct16_Pippin_16-05-06
YDGEEYLILSAR
Charge: 2
Mass: 1427.69326
Most Abundant Isotope m/z: 714.854
Score: 5
QValue: N/A
Spectrum View
XIC View
Feature Map
MS/MS Spectrum (Scan: 22405)
Intensity
m/z
y1(1+)
y2(1+)
y4(1+)
y5(1+)
y6(1+)
y11(1+)
Secondary Spectra
Previous Ms1 Spectrum (Scan: 22403)
Next Ms1 Spectrum (Scan: 22406)
<->

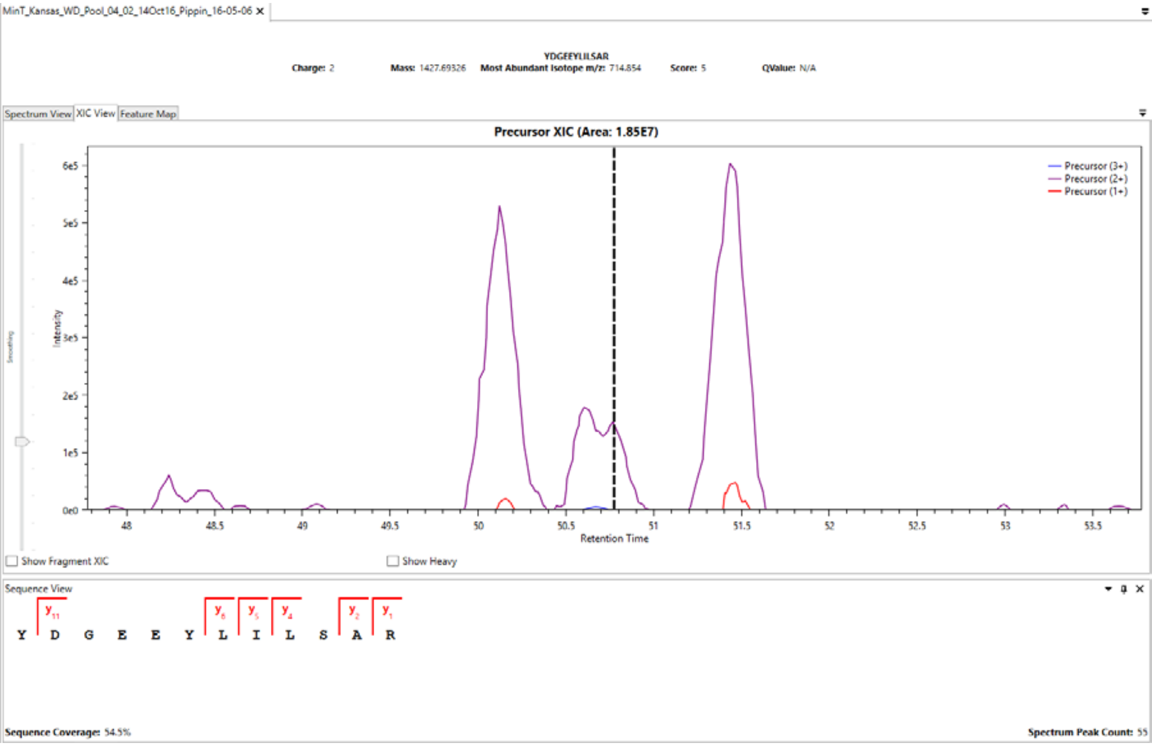
MinT_Kansas_WD_Pool_04_02_14Oct16_Pippin_16-05-06
YDGEEYLILSAR
Charge: 2
Mass: 1427.69326
Most Abundant Isotope m/z: 714.854
Score: 5
QValue: N/A
Spectrum View
XIC View
Feature Map
Precursor XIC (Area: 1.85E7)
Precursor (3+)
Precursor (2+)
Precursor (1+)
Intensity
Retention Time
Show Fragment XIC
Show Heavy
Sequence View
Y D G E E Y L I L S A R
Sequence Coverage: 54.5%
Spectrum Peak Count: 55

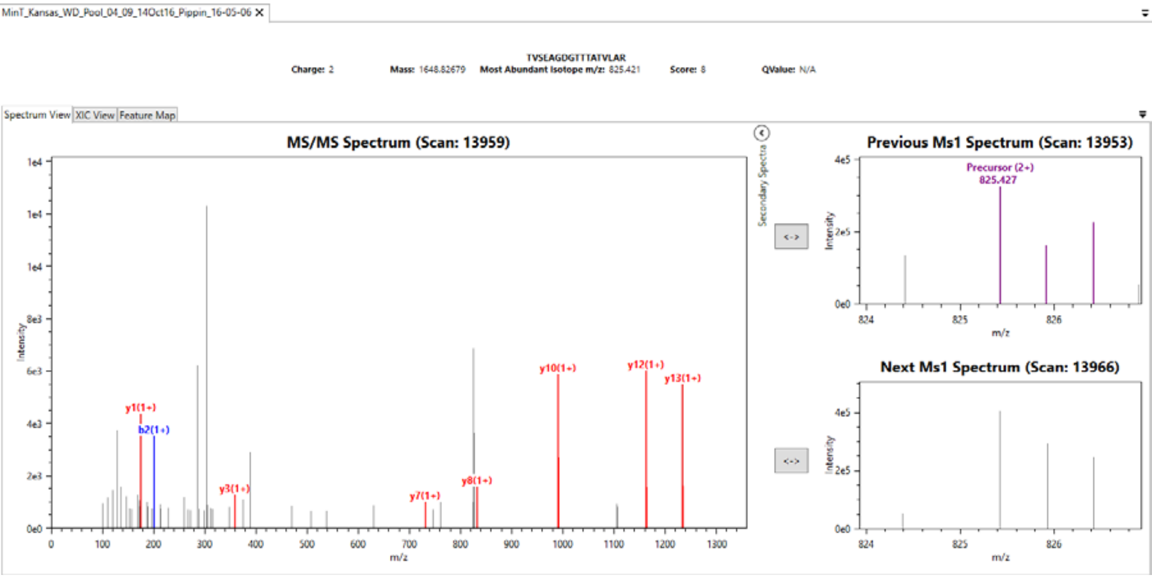
MinT_Kansas_WD_Pool_04_09_14Oct16_Pippin_16-05-06
TVSEAGDGTTTATVLAR
Charge: 2
Mass: 1648.82679
Most Abundant Isotope m/z: 825.421
Score: 8
QValue: N/A
Spectrum View
XIC View
Feature Map
MS/MS Spectrum (Scan: 13959)
Intensity
m/z
y1(1+)
b2(1+)
y3(1+)
y7(1+)
y8(1+)
y10(1+)
y12(1+)
y13(1+)
Secondary Spectra
Previous Ms1 Spectrum (Scan: 13953)
Precursor (2+)
825.427
Next Ms1 Spectrum (Scan: 13966)

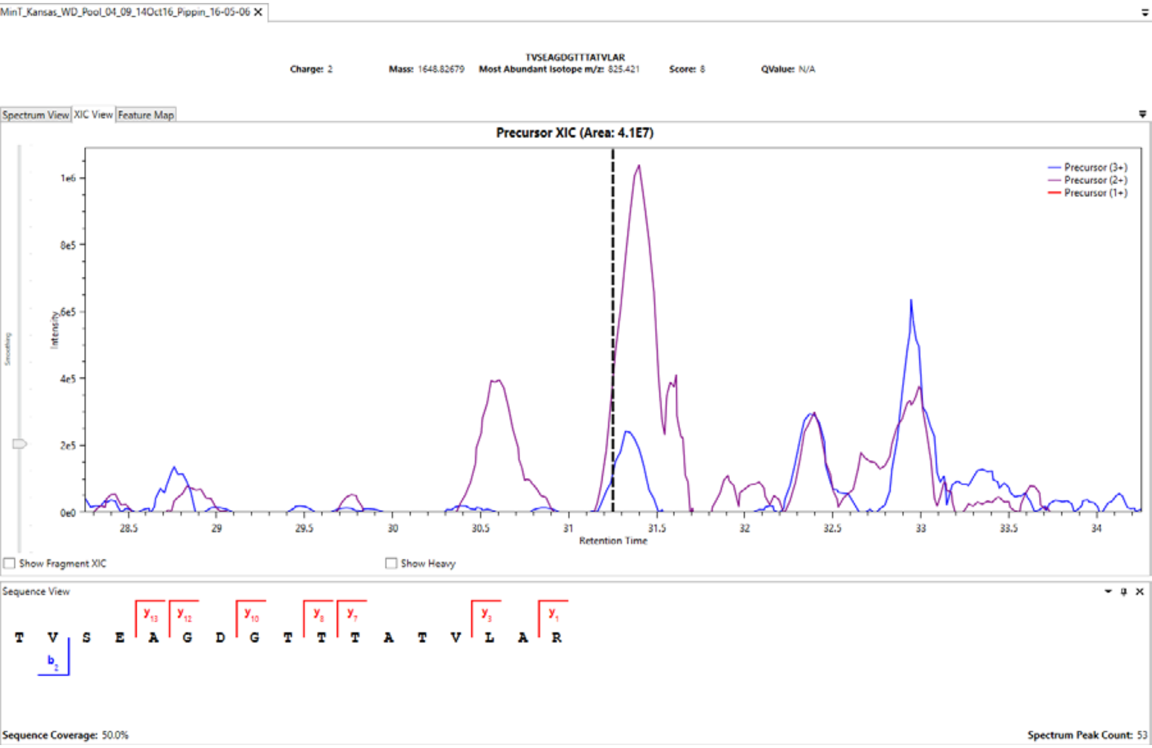
MinT_Kansas_WD_Pool_04_09_14Oct16_Pippin_16-05-06
TVSEAGDGTTTATVLAR
Charge: 2
Mass: 1648.82679
Most Abundant Isotope m/z: 825.421
Score: 8
QValue: N/A
Spectrum View
XIC View
Feature Map
Precursor XIC (Area: 4.1E7)
Intensity
Retention Time
Precursor (3+)
Precursor (2+)
Precursor (1+)
Show Fragment XIC
Show Heavy
Sequence View
T V S E A G D G T T T A T V L A R
Sequence Coverage: 50.0%
Spectrum Peak Count: 53

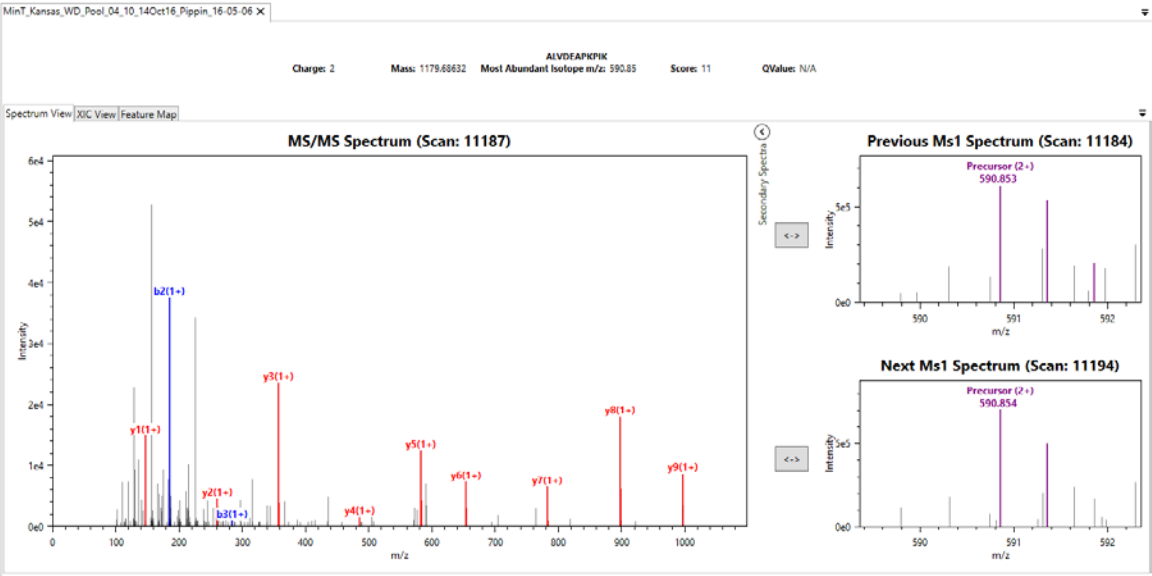
MinT_Kansas_WD_Pool_04_10_14Oct16_Pippin_16-05-06
ALVDEAPKPIK
Charge: 2
Mass: 1179.68632
Most Abundant Isotope m/z: 590.85
Score: 11
QValue: N/A
Spectrum View
XIC View
Feature Map
MS/MS Spectrum (Scan: 11187)
Intensity
m/z
b2(1+)
b3(1+)
y1(1+)
y2(1+)
y3(1+)
y4(1+)
y5(1+)
y6(1+)
y7(1+)
y8(1+)
y9(1+)
Secondary Spectra
Previous Ms1 Spectrum (Scan: 11184)
Precursor (2+)
590.853
Next Ms1 Spectrum (Scan: 11194)
Precursor (2+)
590.854

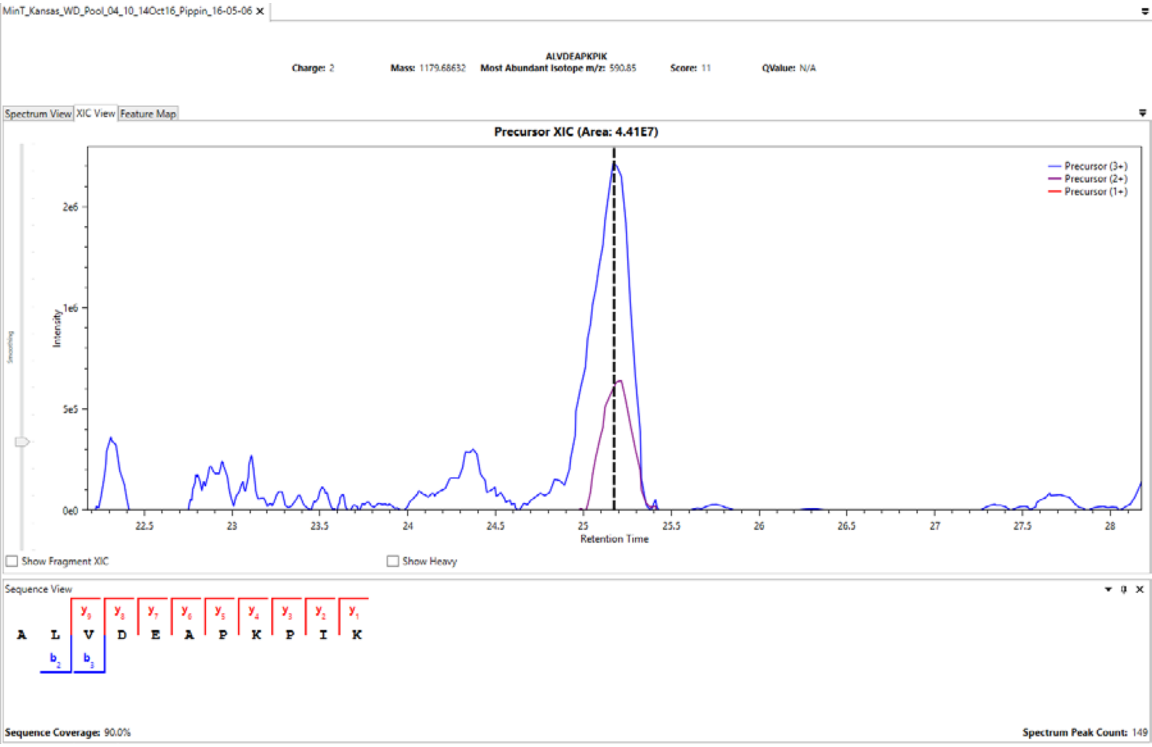
MinT_Kansas_WD_Pool_04_10_14Oct16_Pippin_16-05-06
ALVDEAPKPIK
Charge: 2
Mass: 1179.68632
Most Abundant Isotope m/z: 590.85
Score: 11
QValue: N/A
Spectrum View
XIC View
Feature Map
Precursor XIC (Area: 4.41E7)
Intensity
Retention Time
Precursor (3+)
Precursor (2+)
Precursor (1+)
Show Fragment XIC
Show Heavy
Sequence View
A L V D E A P K P I K
Sequence Coverage: 90.0%
Spectrum Peak Count: 149

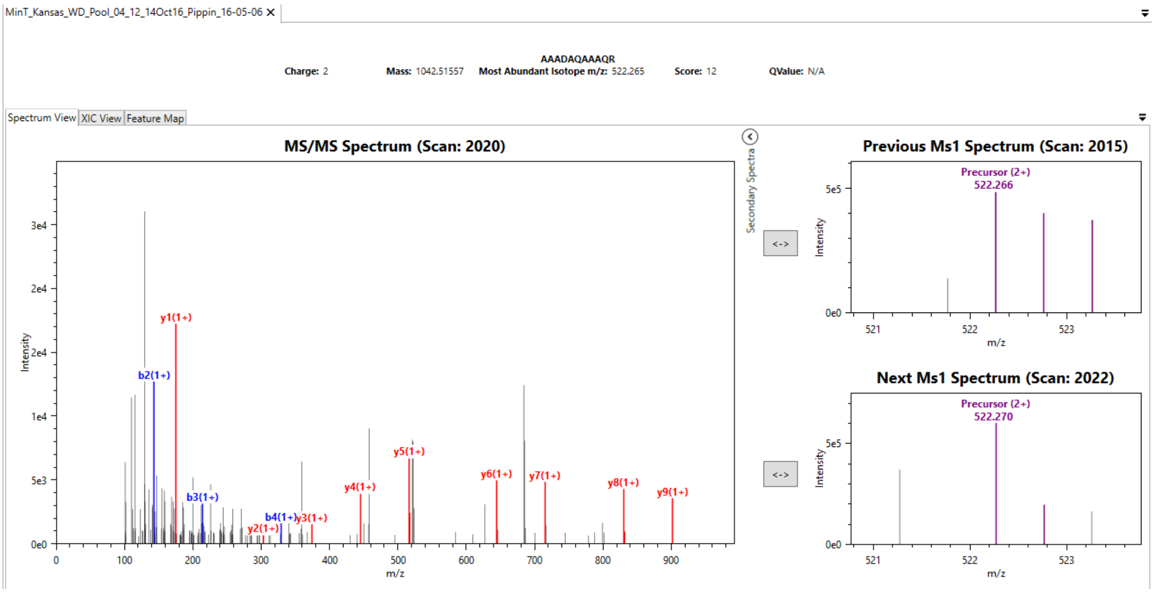
MinT_Kansas_WD_Pool_04_12_14Oct16_Pippin_16-05-06
AAADAQAAAQR
Charge: 2
Mass: 1042.51557
Most Abundant Isotope m/z: 522.265
Score: 12
QValue: N/A
Spectrum View
XIC View
Feature Map
MS/MS Spectrum (Scan: 2020)
Intensity
m/z
b2(1+)
b3(1+)
b4(1+)
y1(1+)
y2(1+)
y3(1+)
y4(1+)
y5(1+)
y6(1+)
y7(1+)
y8(1+)
y9(1+)
Secondary Spectra
Previous Ms1 Spectrum (Scan: 2015)
Precursor (2+)
522.266
Next Ms1 Spectrum (Scan: 2022)
Precursor (2+)
522.270

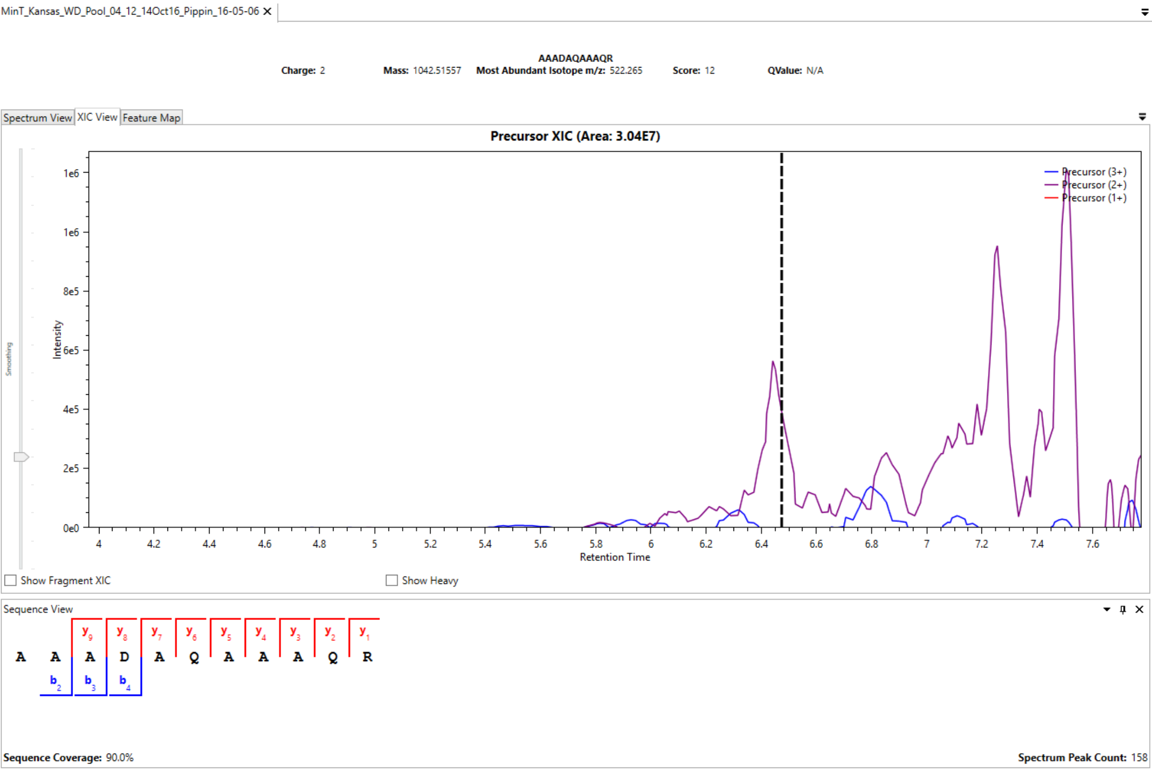
MinT_Kansas_WD_Pool_04_12_14Oct16_Pippin_16-05-06
AAADAQAAAQR
Charge: 2
Mass: 1042.51557
Most Abundant Isotope m/z: 522.265
Score: 12
QValue: N/A
Spectrum View
XIC View
Feature Map
Precursor XIC (Area: 3.04E7)
Intensity
Retention Time
Precursor (3+)
Precursor (2+)
Precursor (1+)
Smoothing
Show Fragment XIC
Show Heavy
Sequence View
A A A D A Q A A A Q R
Sequence Coverage: 90.0%
Spectrum Peak Count: 158

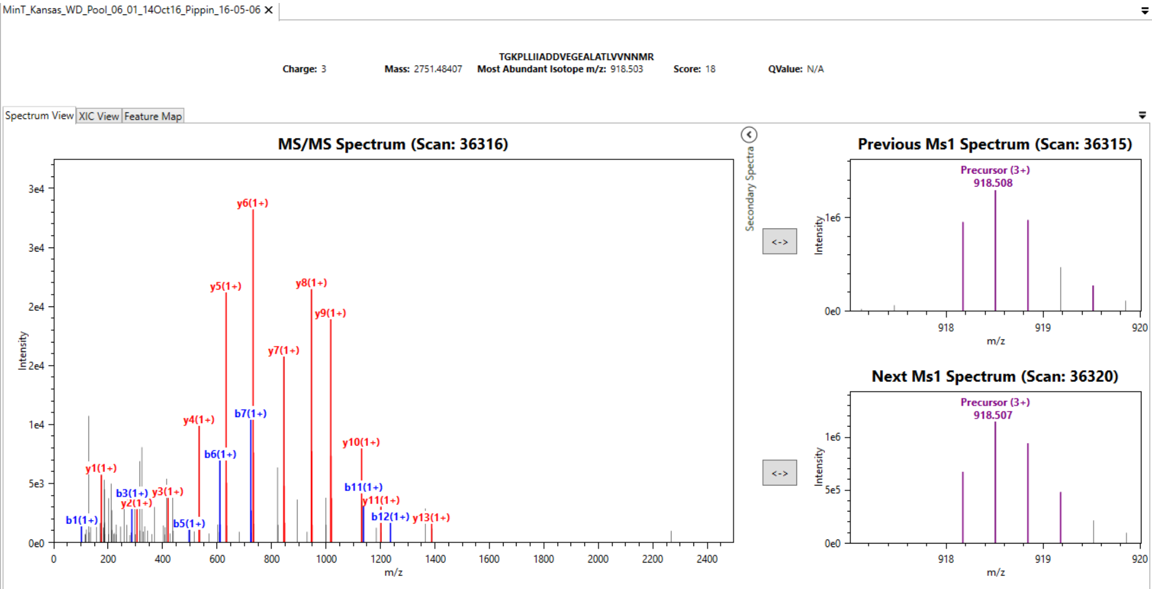
MinT_Kansas_WD_Pool_06_01_14Oct16_Pippin_16-05-06
TGKPLLIIADDVEGEALATLVVNNMR
Charge: 3
Mass: 2751.48407
Most Abundant Isotope m/z: 918.503
Score: 18
QValue: N/A
Spectrum View
XIC View
Feature Map
MS/MS Spectrum (Scan: 36316)
Intensity
m/z
y6(1+)
y5(1+)
y8(1+)
y9(1+)
y7(1+)
y4(1+)
b7(1+)
b6(1+)
y10(1+)
y1(1+)
b11(1+)
b3(1+)
y3(1+)
y2(1+)
y11(1+)
b1(1+)
b12(1+)
y13(1+)
b5(1+)
Secondary Spectra
Previous Ms1 Spectrum (Scan: 36315)
Precursor (3+)
918.508
Next Ms1 Spectrum (Scan: 36320)
Precursor (3+)
918.507

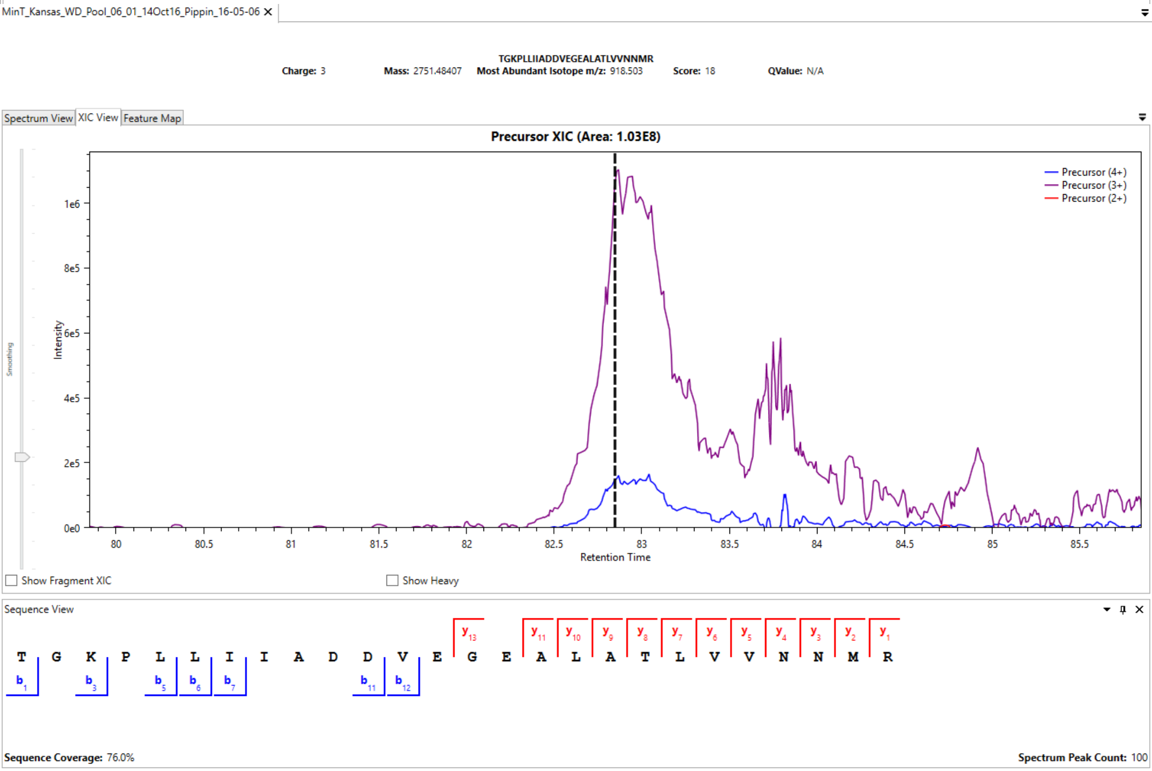
MinT_Kansas_WD_Pool_06_01_14Oct16_Pippin_16-05-06
TGKPLLIIADDVEGEALATLVVNNMR
Charge: 3
Mass: 2751.48407
Most Abundant Isotope m/z: 918.503
Score: 18
QValue: N/A
Spectrum View
XIC View
Feature Map
Precursor XIC (Area: 1.03E8)
Intensity
Retention Time
Precursor (4+)
Precursor (3+)
Precursor (2+)
Smoothing
Show Fragment XIC
Show Heavy
Sequence View
T G K P L L I I A D D V E G E A L A T L V V N N M R
Sequence Coverage: 76.0%
Spectrum Peak Count: 100

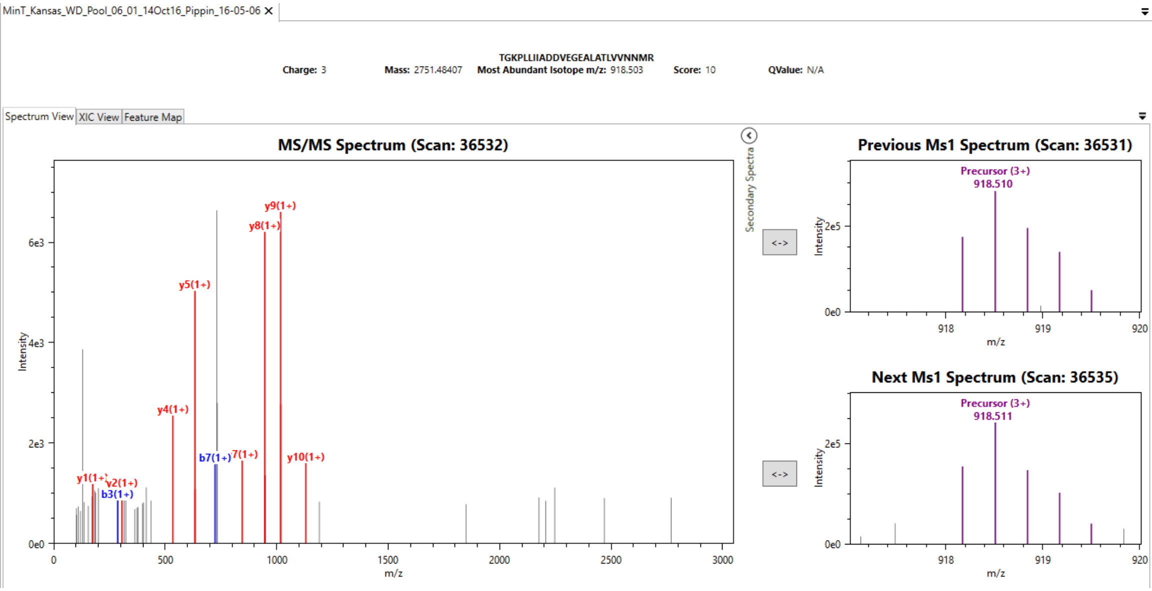
MinT_Kansas_WD_Pool_06_01_14Oct16_Pippin_16-05-06
TGKPLLIIADDVEGEALATLVVNNMR
Charge: 3
Mass: 2751.48407
Most Abundant Isotope m/z: 918.503
Score: 10
QValue: N/A
Spectrum View
XIC View
Feature Map
MS/MS Spectrum (Scan: 36532)
Intensity
m/z
y1(1+)
y2(1+)
b3(1+)
y4(1+)
y5(1+)
b7(1+)
y7(1+)
y8(1+)
y9(1+)
y10(1+)
Secondary Spectra
<->
Previous Ms1 Spectrum (Scan: 36531)
Precursor (3+)
918.510
Next Ms1 Spectrum (Scan: 36535)
Precursor (3+)
918.511

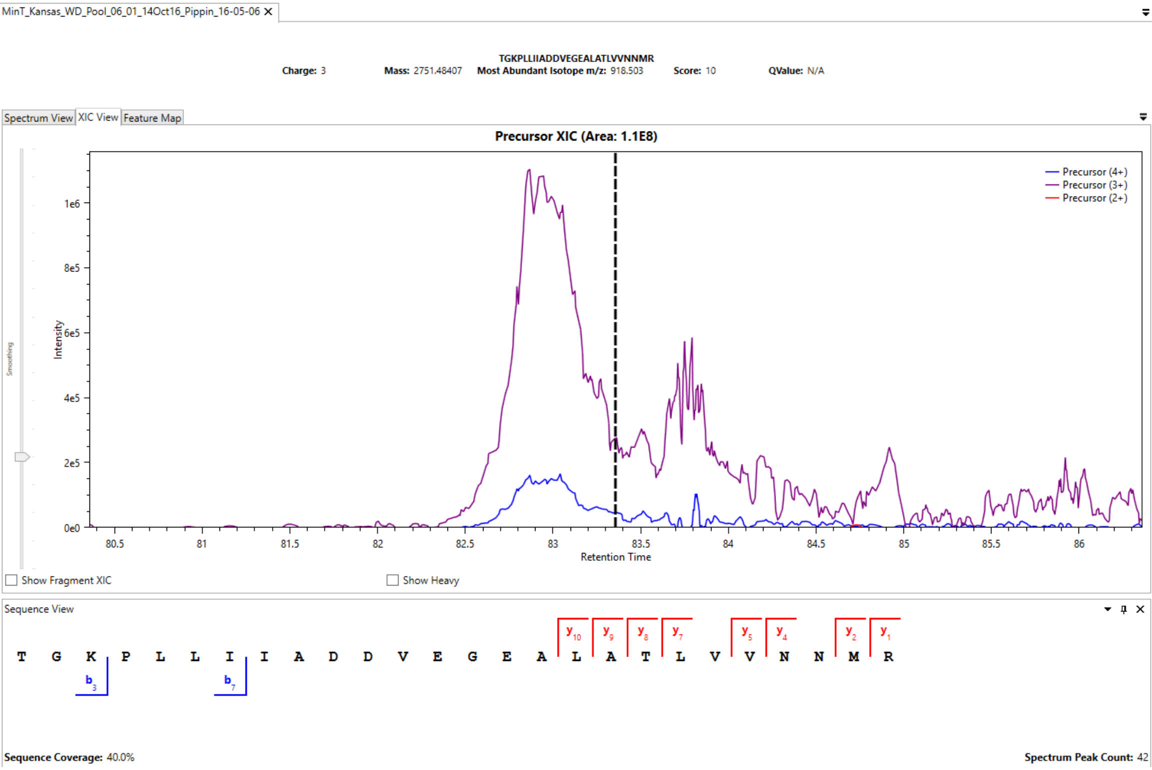
MinT_Kansas_WD_Pool_06_01_14Oct16_Pippin_16-05-06
TGKPLLIIADDVEGEALATLVVNNMR
Charge: 3
Mass: 2751.48407
Most Abundant Isotope m/z: 918.503
Score: 10
QValue: N/A
Spectrum View
XIC View
Feature Map
Precursor XIC (Area: 1.1E8)
Intensity
Retention Time
Precursor (4+)
Precursor (3+)
Precursor (2+)
Smoothing
Show Fragment XIC
Show Heavy
Sequence View
T G K P L L I I A D D V E G E A L A T L V V N N M R
b3
b7
y10
y9
y8
y7
y5
y4
y2
y1
Sequence Coverage: 40.0%
Spectrum Peak Count: 42

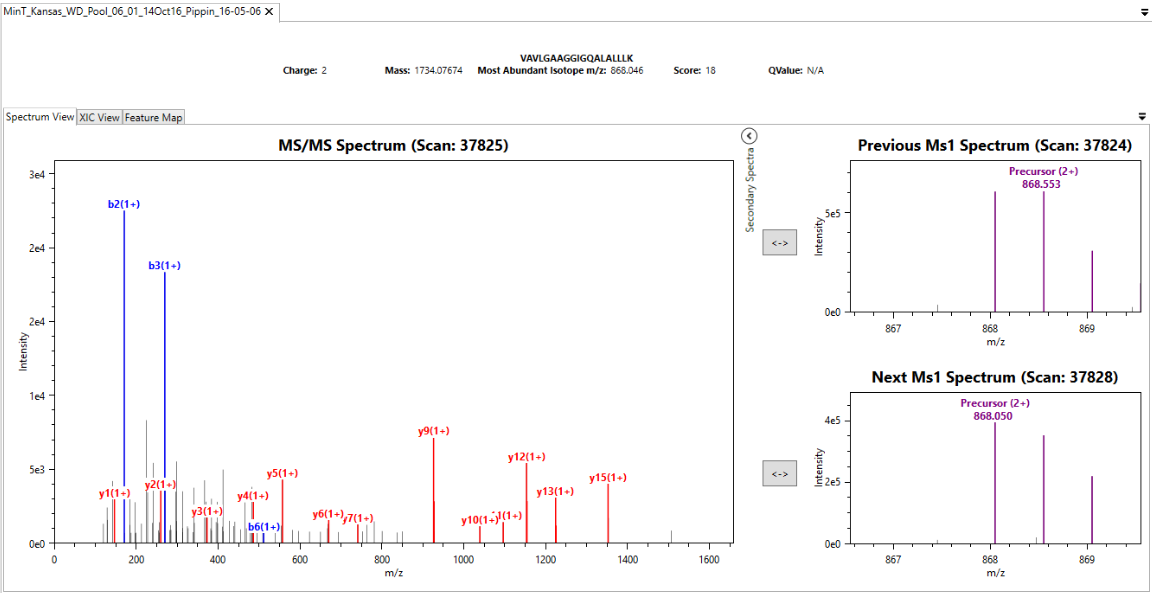

MinT_Kansas_WD_Pool_06_01_14Oct16_Pippin_16-05-06
VAVLGAAGGIGQALALLLK
Charge: 2
Mass: 1734.07674
Most Abundant Isotope m/z: 868.046
Score: 18
QValue: N/A
Spectrum View
XIC View
Feature Map
MS/MS Spectrum (Scan: 37825)
Intensity
m/z
b2(1+)
b3(1+)
y1(1+)
y2(1+)
y3(1+)
y4(1+)
y5(1+)
b6(1+)
y6(1+)
y7(1+)
y9(1+)
y10(1+)
y12(1+)
y13(1+)
y15(1+)
Secondary Spectra
Previous Ms1 Spectrum (Scan: 37824)
Precursor (2+)
868.553
Next Ms1 Spectrum (Scan: 37828)
Precursor (2+)
868.050

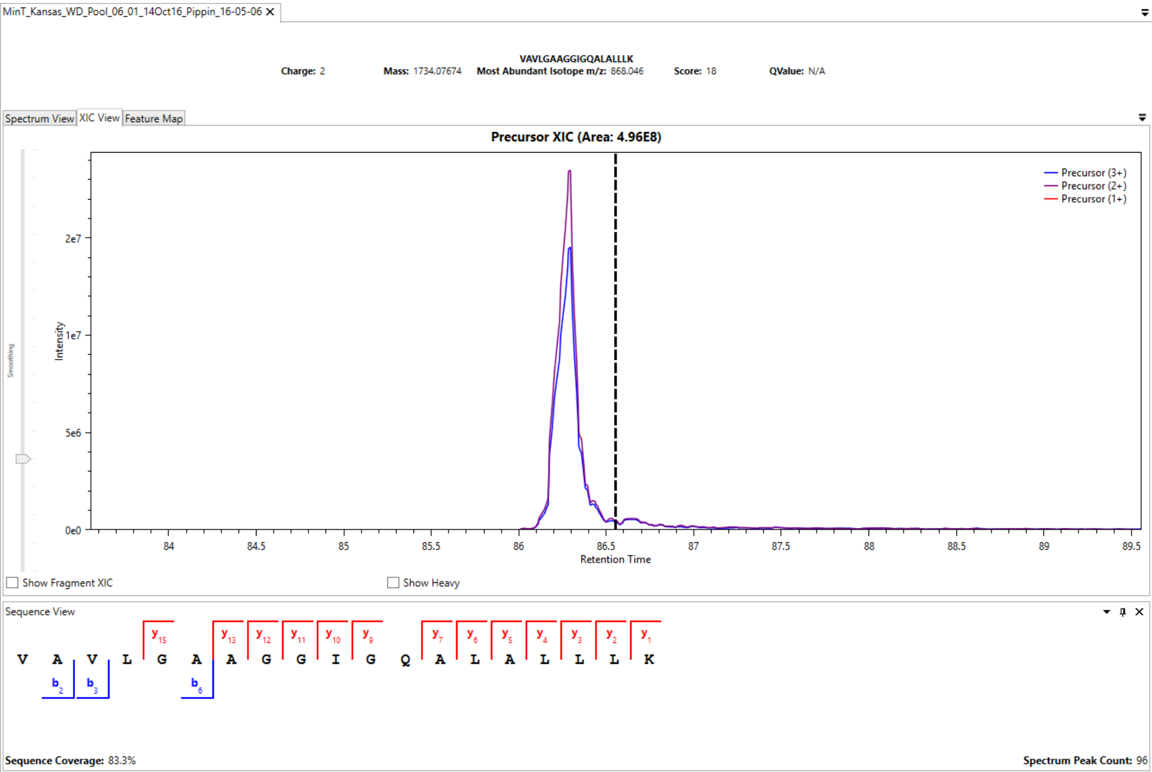

MinT_Kansas_WD_Pool_06_01_14Oct16_Pippin_16-05-06
VAVLGAAGGIGQALALLLK
Charge: 2
Mass: 1734.07674
Most Abundant Isotope m/z: 868.046
Score: 18
QValue: N/A
Spectrum View
XIC View
Feature Map
Precursor XIC (Area: 4.96E8)
Precursor (3+)
Precursor (2+)
Precursor (1+)
Intensity
Retention Time
Smoothing
Show Fragment XIC
Show Heavy
Sequence View
V A V L G A A G G I G Q A L A L L L K
Sequence Coverage: 83.3%
Spectrum Peak Count: 96

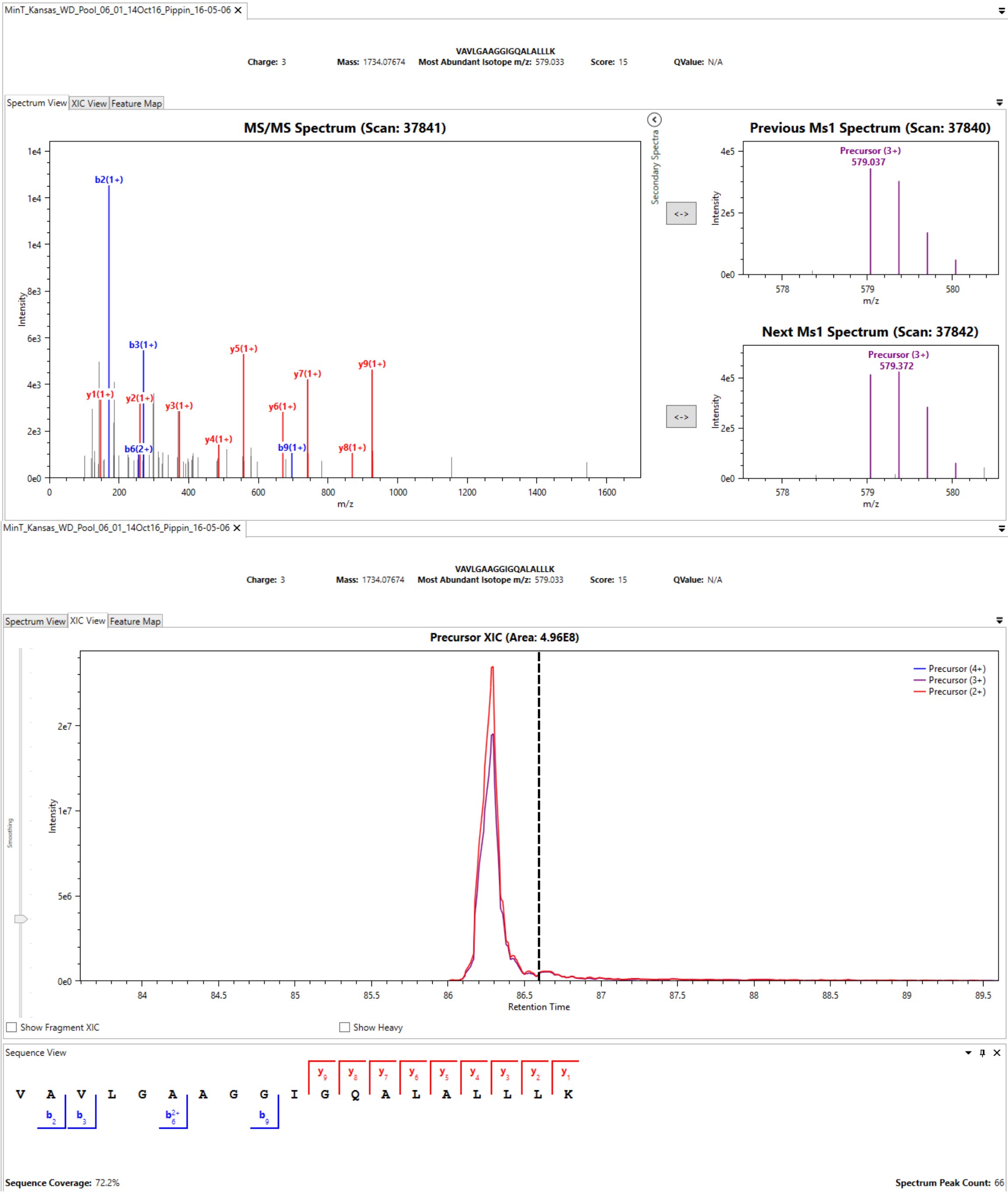
MinT_Kansas_WD_Pool_06_01_14Oct16_Pippin_16-05-06
VAVLGAAGGIGQALALLLK
Charge: 3
Mass: 1734.07674
Most Abundant Isotope m/z: 579.033
Score: 15
QValue: N/A
Spectrum View
XIC View
Feature Map
MS/MS Spectrum (Scan: 37841)
Intensity
m/z
b2(1+)
b3(1+)
b6(2+)
b9(1+)
y1(1+)
y2(1+)
y3(1+)
y4(1+)
y5(1+)
y6(1+)
y7(1+)
y8(1+)
y9(1+)
Secondary Spectra
Previous Ms1 Spectrum (Scan: 37840)
Precursor (3+)
579.037
Next Ms1 Spectrum (Scan: 37842)
Precursor (3+)
579.372
MinT_Kansas_WD_Pool_06_01_14Oct16_Pippin_16-05-06
VAVLGAAGGIGQALALLLK
Charge: 3
Mass: 1734.07674
Most Abundant Isotope m/z: 579.033
Score: 15
QValue: N/A
Spectrum View
XIC View
Feature Map
Precursor XIC (Area: 4.96E8)
Precursor (4+)
Precursor (3+)
Precursor (2+)
Intensity
Retention Time
Smoothing
Show Fragment XIC
Show Heavy
Sequence View
V A V L G A A G G I G Q A L A L L L K
Sequence Coverage: 72.2%
Spectrum Peak Count: 66

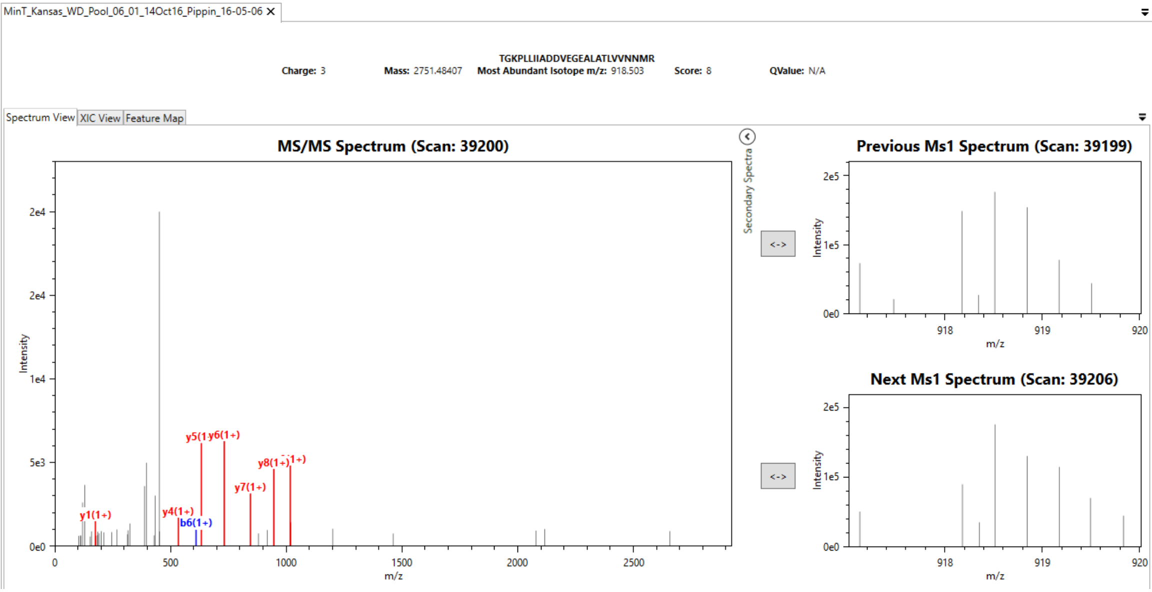
MinT_Kansas_WD_Pool_06_01_14Oct16_Pippin_16-05-06
TGKPLLIIADDVEGEALATLVVNNMR
Charge: 3
Mass: 2751.48407
Most Abundant Isotope m/z: 918.503
Score: 8
QValue: N/A
Spectrum View
XIC View
Feature Map
MS/MS Spectrum (Scan: 39200)
Intensity
m/z
y1(1+)
y4(1+)
b6(1+)
y5(1+)
y6(1+)
y7(1+)
y8(1+)
Secondary Spectra
Previous Ms1 Spectrum (Scan: 39199)
Next Ms1 Spectrum (Scan: 39206)

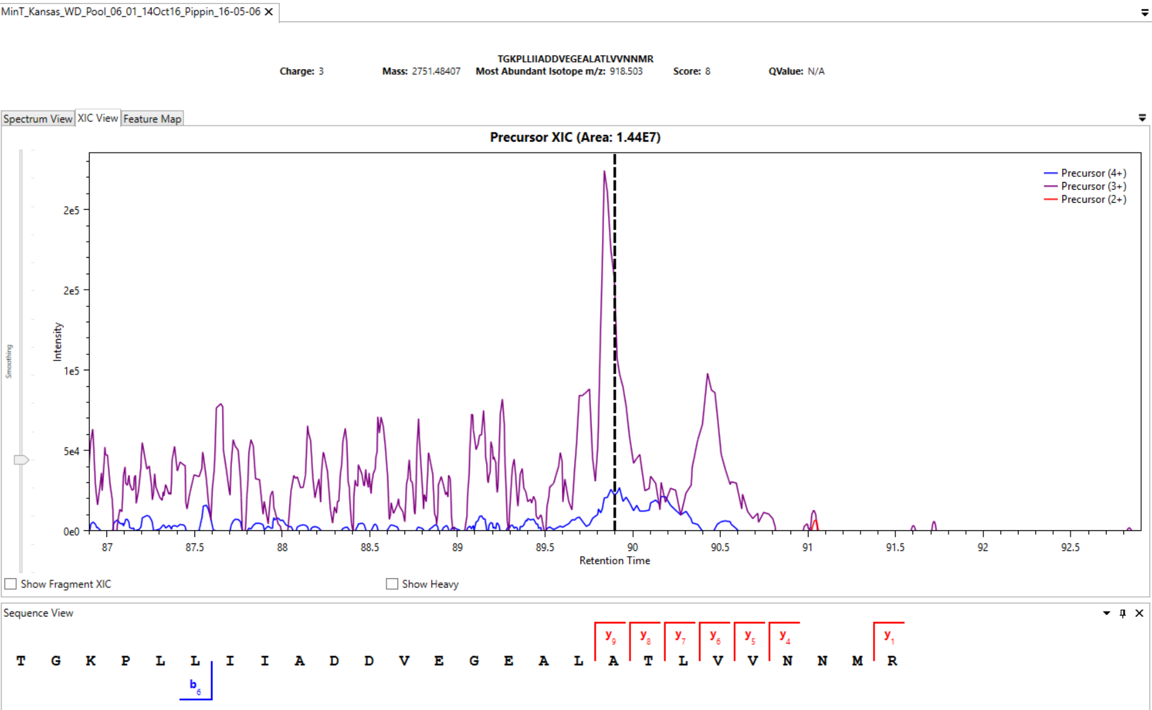
MinT_Kansas_WD_Pool_06_01_14Oct16_Pippin_16-05-06
TGKPLLIIADDVEGEALATLVVNNMR
Charge: 3
Mass: 2751.48407
Most Abundant Isotope m/z: 918.503
Score: 8
QValue: N/A
Spectrum View
XIC View
Feature Map
Precursor XIC (Area: 1.44E7)
Precursor (4+)
Precursor (3+)
Precursor (2+)
Intensity
Retention Time
Smoothing
Show Fragment XIC
Show Heavy
Sequence View
T G K P L L I I A D D V E G E A L A T L V V N N M R

MinT_Kansas_WD_Pool_06_02_14Oct16_Pippin_16-05-06

TGKPLLIIADDVEGEALATLVVNNMR

Charge: 3 **Mass:** 2751.48407 **Most Abundant Isotope m/z:** 918.503 **Score:** 15 **QValue:** N/A

Spectrum View | XIC View | Feature Map

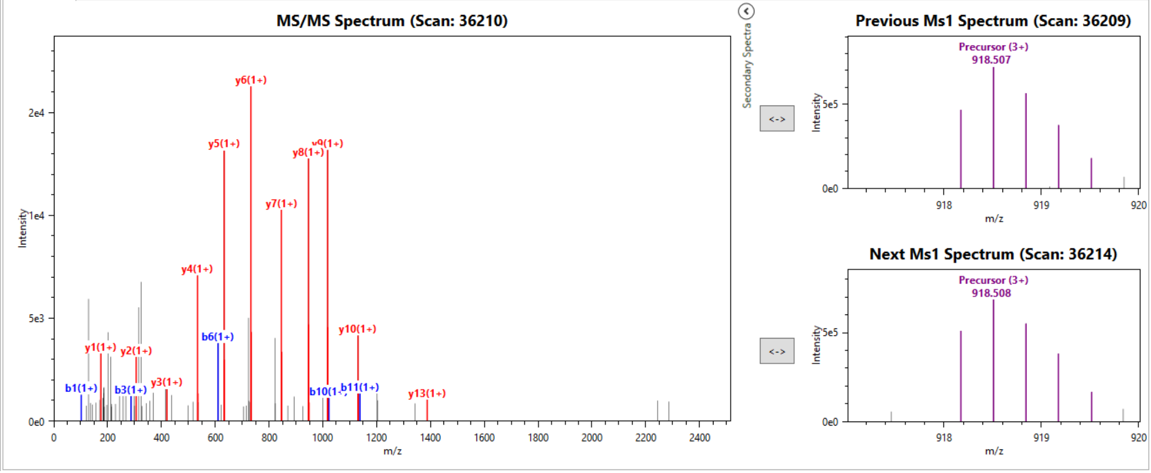

MinT_Kansas_WD_Pool_06_02_14Oct16_Pippin_16-05-06

TGKPLLIIADDVEGEALATLVVNNMR

Charge: 3 **Mass:** 2751.48407 **Most Abundant Isotope m/z:** 918.503 **Score:** 15 **QValue:** N/A

Spectrum View | XIC View | Feature Map

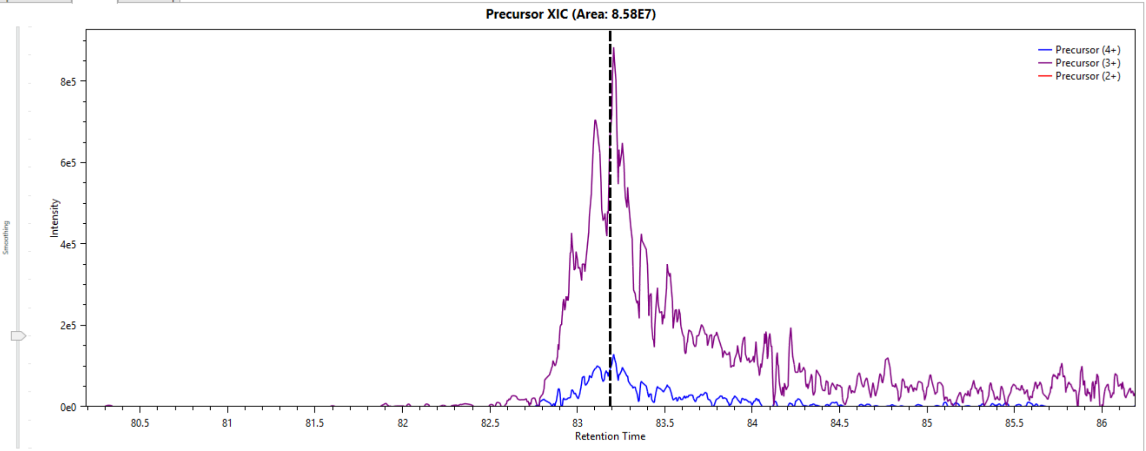

Show Fragment XIC   Show Heavy

Sequence View

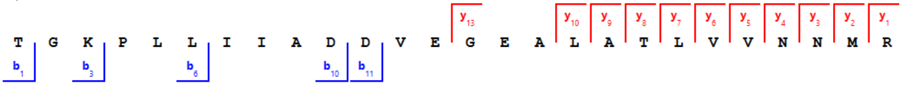

Sequence Coverage: 64.0%

Spectrum Peak Count: 77

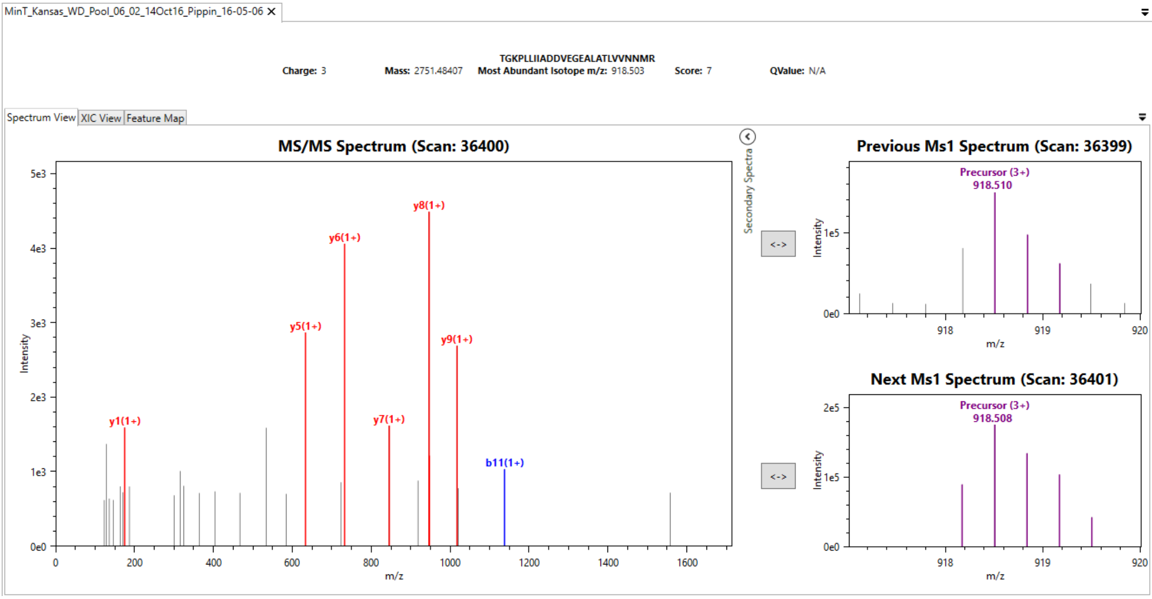
MinT_Kansas_WD_Pool_06_02_14Oct16_Pippin_16-05-06
TGKPLLIIADDVEGEALATLVVNNMR
Charge: 3
Mass: 2751.48407
Most Abundant Isotope m/z: 918.503
Score: 7
QValue: N/A
Spectrum View
XIC View
Feature Map
MS/MS Spectrum (Scan: 36400)
Intensity
m/z
y1(1+)
y5(1+)
y6(1+)
y7(1+)
y8(1+)
y9(1+)
b11(1+)
Secondary Spectra
Previous Ms1 Spectrum (Scan: 36399)
Precursor (3+)
918.510
Next Ms1 Spectrum (Scan: 36401)
Precursor (3+)
918.508

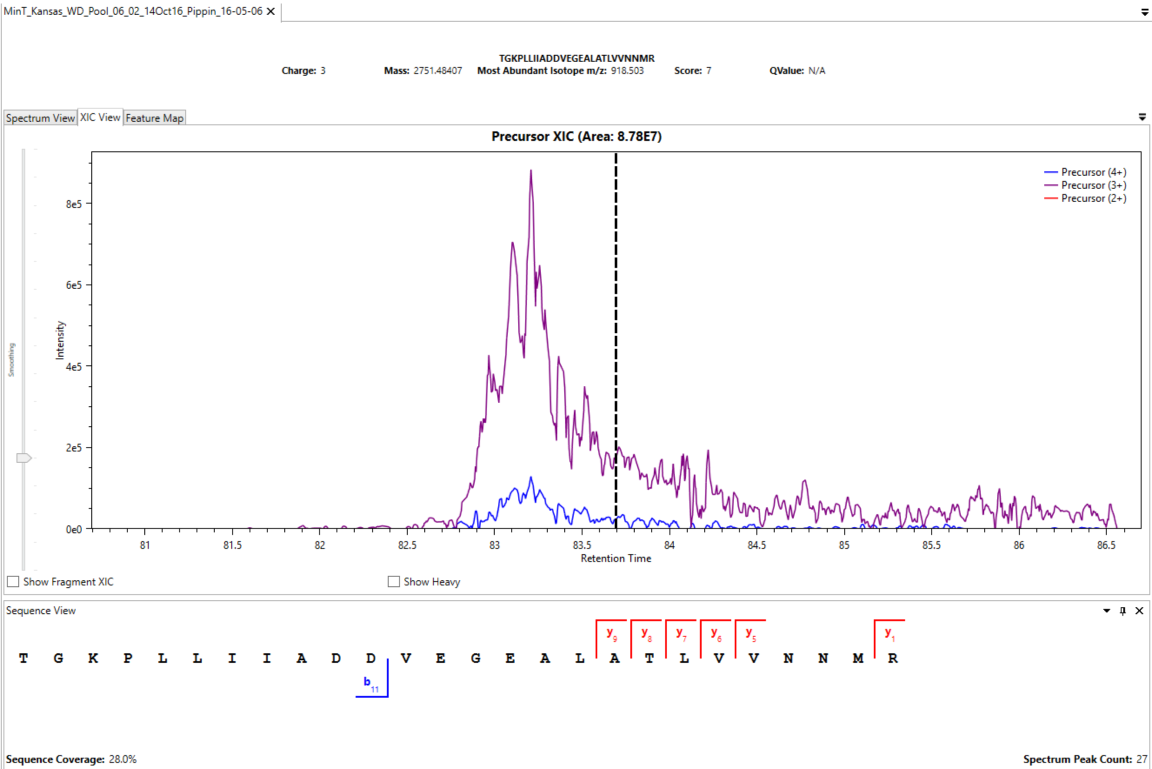
MinT_Kansas_WD_Pool_06_02_14Oct16_Pippin_16-05-06
TGKPLLIIADDVEGEALATLVVNNMR
Charge: 3
Mass: 2751.48407
Most Abundant Isotope m/z: 918.503
Score: 7
QValue: N/A
Spectrum View
XIC View
Feature Map
Precursor XIC (Area: 8.78E7)
Intensity
Retention Time
Precursor (4+)
Precursor (3+)
Precursor (2+)
Show Fragment XIC
Show Heavy
Sequence View
T G K P L L I I A D D V E G E A L A T L V V N N M R
Sequence Coverage: 28.0%
Spectrum Peak Count: 27

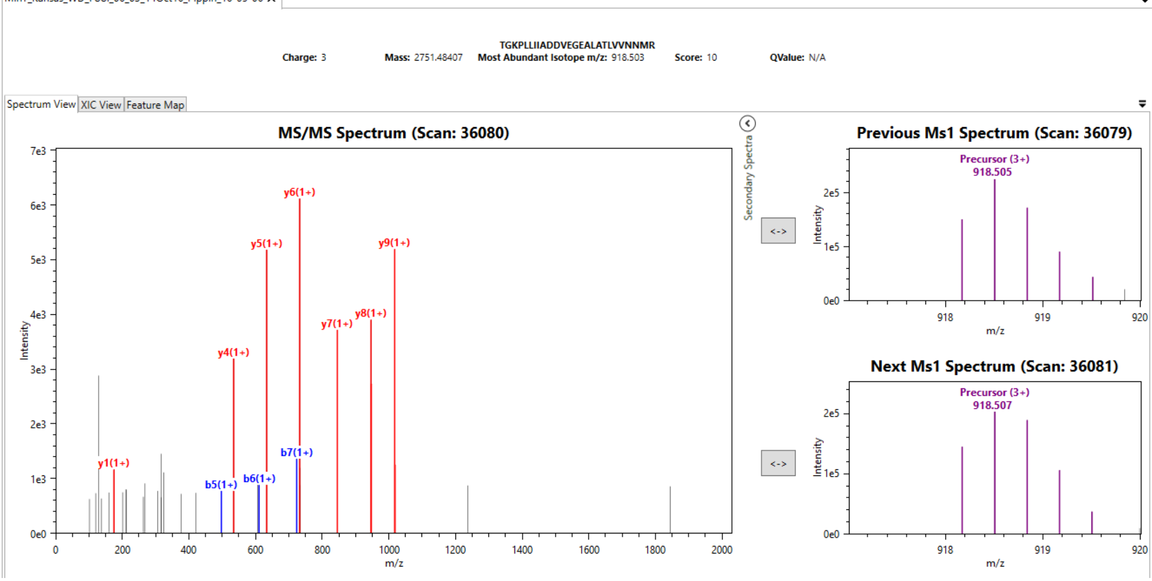
TGKPLLIIADDVEGEALATLVVNNMR
Charge: 3
Mass: 2751.48407
Most Abundant Isotope m/z: 918.503
Score: 10
QValue: N/A
Spectrum View
XIC View
Feature Map
MS/MS Spectrum (Scan: 36080)
Intensity
m/z
y1(1+)
y4(1+)
y5(1+)
y6(1+)
y7(1+)
y8(1+)
y9(1+)
b5(1+)
b6(1+)
b7(1+)
Secondary Spectra
Previous Ms1 Spectrum (Scan: 36079)
Precursor (3+)
918.505
Next Ms1 Spectrum (Scan: 36081)
Precursor (3+)
918.507

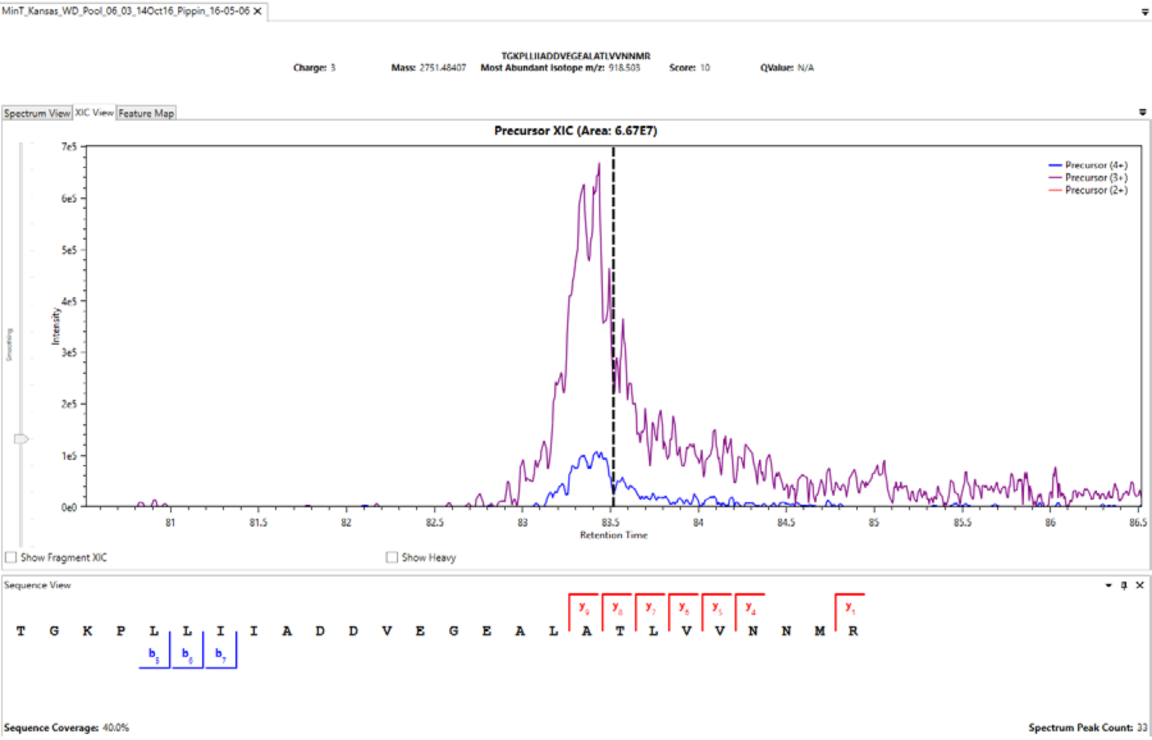
MinT_Kansas_WD_Pool_06_03_14Oct16_Pippin_16-05-06
TGKPLLIIADDVEGEALATLVVNNMR
Charge: 3
Mass: 2751.48407
Most Abundant Isotope m/z: 918.503
Score: 10
QValue: N/A
Spectrum View
XIC View
Feature Map
Precursor XIC (Area: 6.67E7)
Intensity
Retention Time
Precursor (4+)
Precursor (3+)
Precursor (2+)
Show Fragment XIC
Show Heavy
Sequence View
T G K P L L I I A D D V E G E A L A T L V V N N M R
Sequence Coverage: 40.0%
Spectrum Peak Count: 33

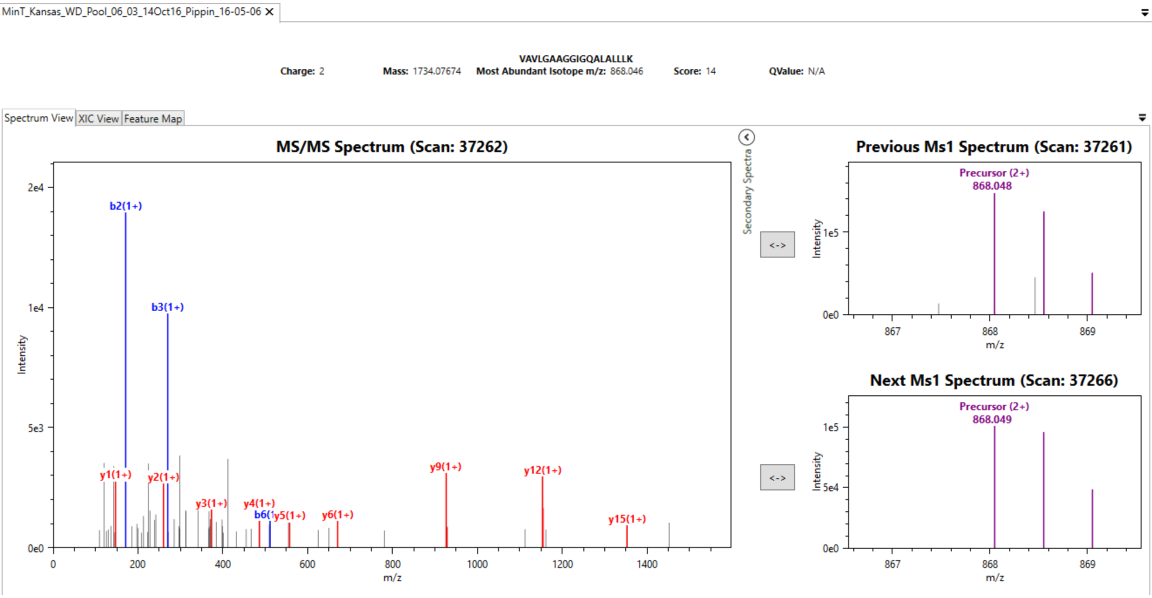
MinT_Kansas_WD_Pool_06_03_14Oct16_Pippin_16-05-06
VAVLGAAGGIGQALALLLK
Charge: 2
Mass: 1734.07674
Most Abundant Isotope m/z: 868.046
Score: 14
QValue: N/A
Spectrum View
XIC View
Feature Map
MS/MS Spectrum (Scan: 37262)
Intensity
m/z
b2(1+)
b3(1+)
b6(1+)
y1(1+)
y2(1+)
y3(1+)
y4(1+)
y5(1+)
y6(1+)
y9(1+)
y12(1+)
y15(1+)
Secondary Spectra
Previous Ms1 Spectrum (Scan: 37261)
Precursor (2+)
868.048
Next Ms1 Spectrum (Scan: 37266)
Precursor (2+)
868.049

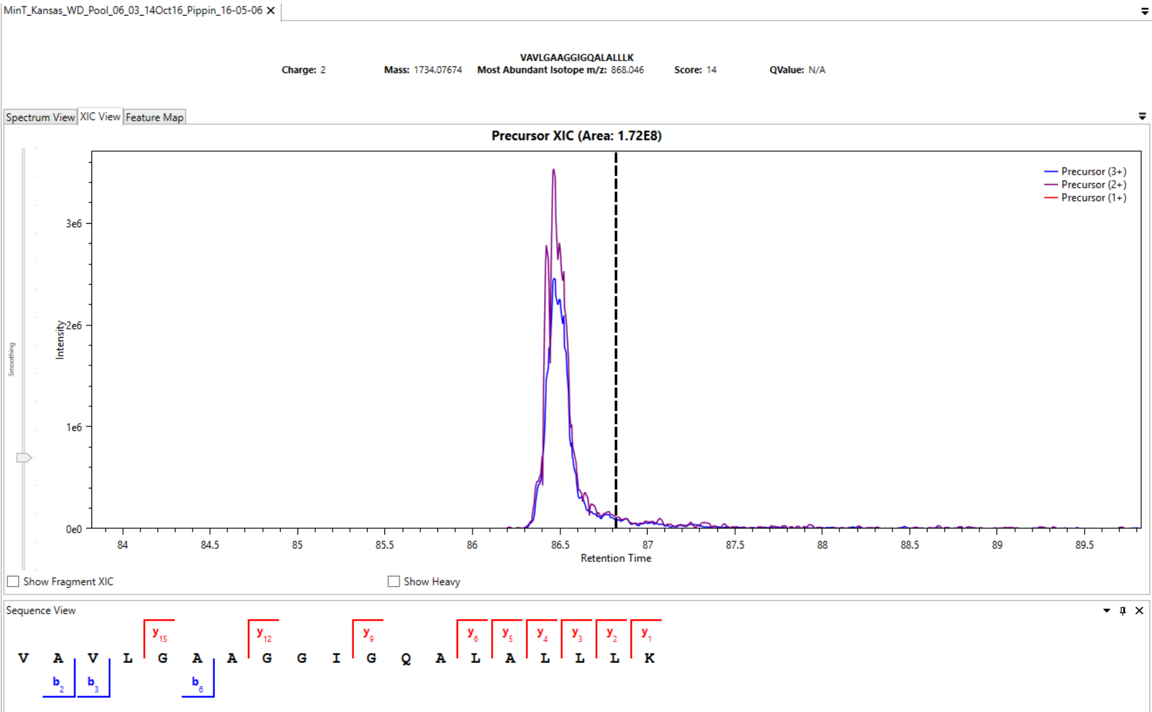
MinT_Kansas_WD_Pool_06_03_14Oct16_Pippin_16-05-06
VAVLGAAGGIGQALALLLK
Charge: 2
Mass: 1734.07674
Most Abundant Isotope m/z: 868.046
Score: 14
QValue: N/A
Spectrum View
XIC View
Feature Map
Precursor XIC (Area: 1.72E8)
Intensity
Retention Time
Precursor (3+)
Precursor (2+)
Precursor (1+)
Smoothing
Show Fragment XIC
Show Heavy
Sequence View
V A V L G A A G G I G Q A L A L L L K

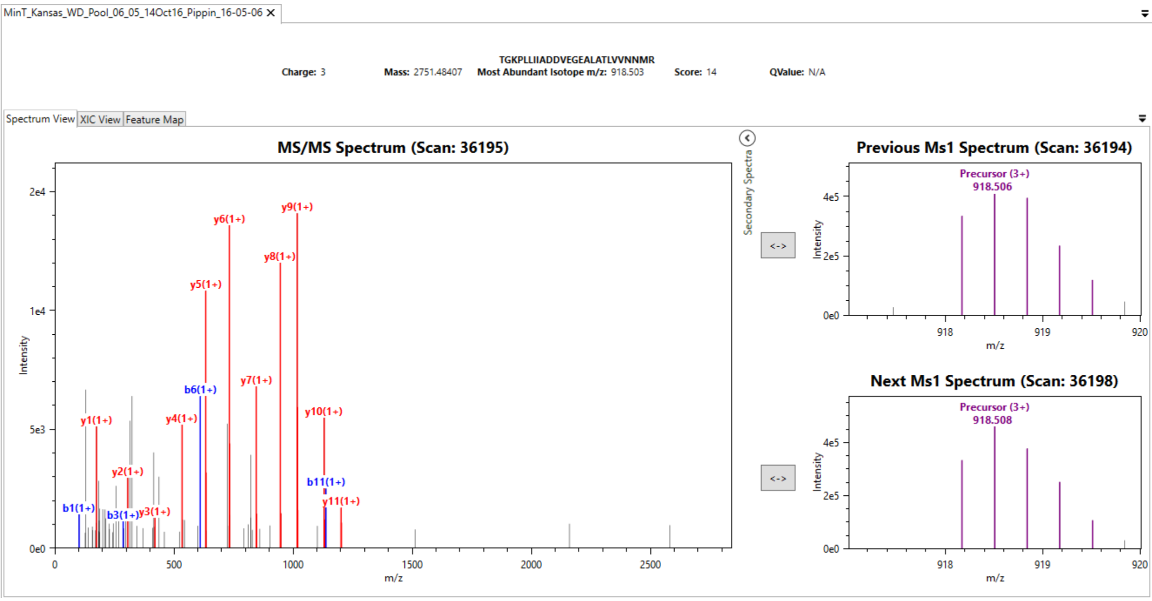
MinT_Kansas_WD_Pool_06_05_14Oct16_Pippin_16-05-06
TGKPLLIIADDVEGEALATLVVNNMR
Charge: 3
Mass: 2751.48407
Most Abundant Isotope m/z: 918.503
Score: 14
QValue: N/A
Spectrum View
XIC View
Feature Map
MS/MS Spectrum (Scan: 36195)
Intensity
m/z
y9(1+)
y6(1+)
y8(1+)
y5(1+)
y7(1+)
b6(1+)
y10(1+)
y1(1+)
y4(1+)
y2(1+)
b11(1+)
b1(1+)
y11(1+)
b3(1+)
y3(1+)
Secondary Spectra
Previous Ms1 Spectrum (Scan: 36194)
Precursor (3+)
918.506
Next Ms1 Spectrum (Scan: 36198)
Precursor (3+)
918.508

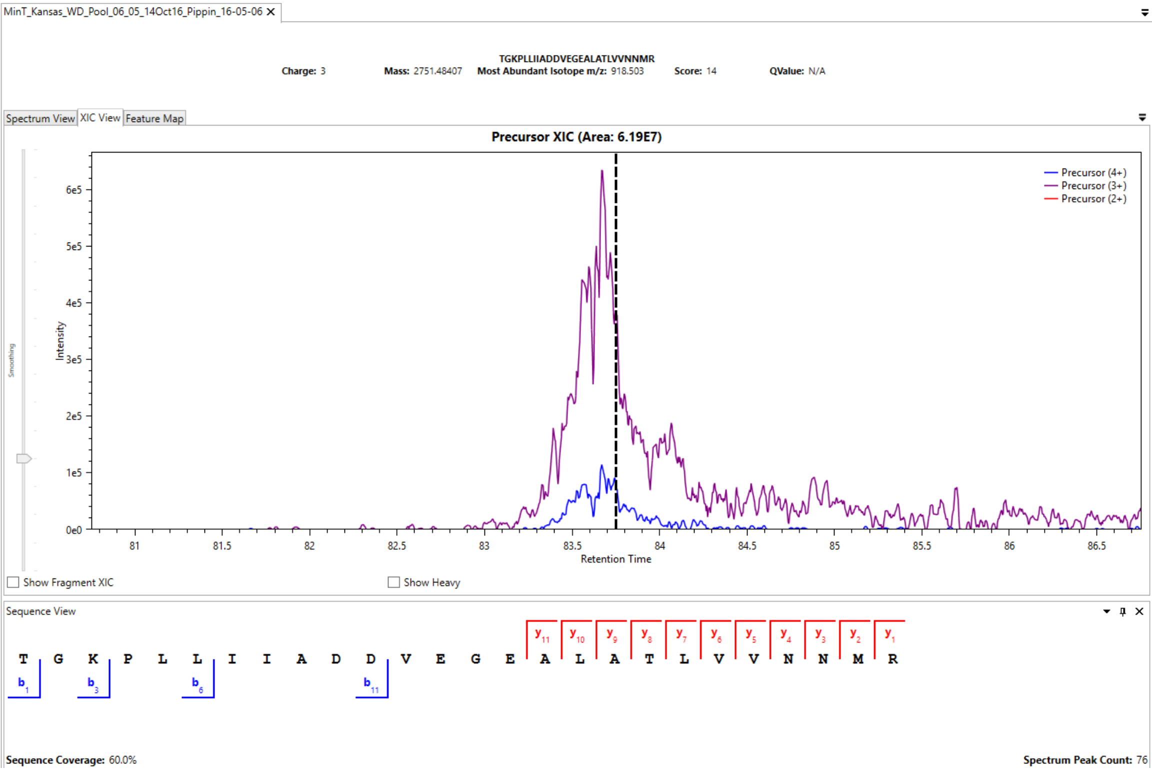
MinT_Kansas_WD_Pool_06_05_14Oct16_Pippin_16-05-06
TGKPLLIIADDVEGEALATLVVNNMR
Charge: 3
Mass: 2751.48407
Most Abundant Isotope m/z: 918.503
Score: 14
QValue: N/A
Spectrum View
XIC View
Feature Map
Precursor XIC (Area: 6.19E7)
Intensity
Retention Time
Precursor (4+)
Precursor (3+)
Precursor (2+)
Smoothing
Show Fragment XIC
Show Heavy
Sequence View
T G K P L L I I A D D V E G E A L A T L V V N N M R
Sequence Coverage: 60.0%
Spectrum Peak Count: 76

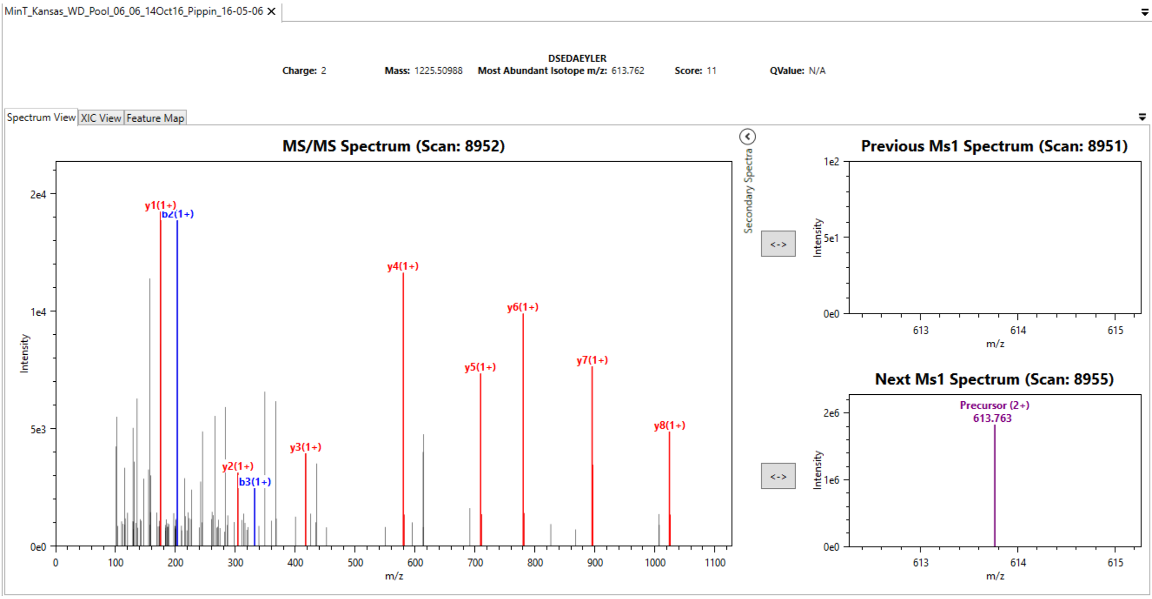
MinT_Kansas_WD_Pool_06_06_14Oct16_Pippin_16-05-06
DSEDAEYLER
Charge: 2
Mass: 1225.50988
Most Abundant Isotope m/z: 613.762
Score: 11
QValue: N/A
Spectrum View
XIC View
Feature Map
MS/MS Spectrum (Scan: 8952)
Intensity
m/z
y1(1+)
b2(1+)
y4(1+)
y6(1+)
y5(1+)
y7(1+)
y8(1+)
y3(1+)
y2(1+)
b3(1+)
Secondary Spectra
<->
Previous Ms1 Spectrum (Scan: 8951)
Next Ms1 Spectrum (Scan: 8955)
Precursor (2+)
613.763

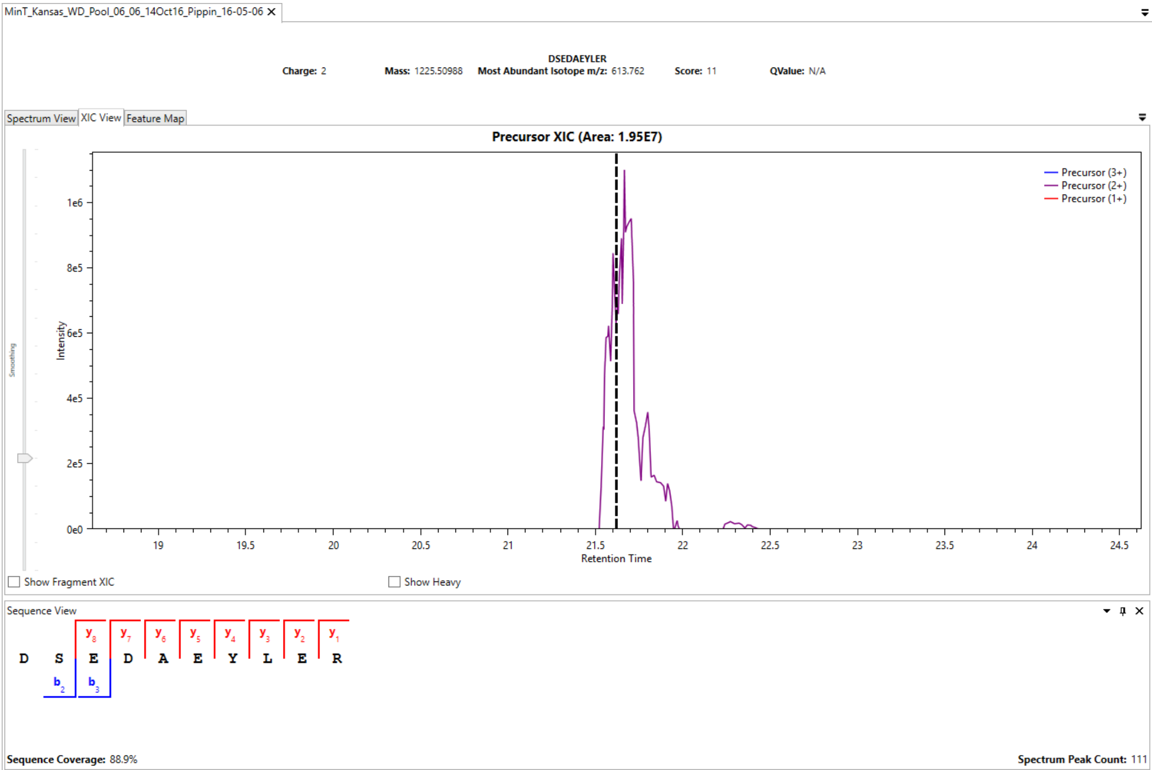
MinT_Kansas_WD_Pool_06_06_14Oct16_Pippin_16-05-06
DSEDAEYLER
Charge: 2
Mass: 1225.50988
Most Abundant Isotope m/z: 613.762
Score: 11
QValue: N/A
Spectrum View
XIC View
Feature Map
Precursor XIC (Area: 1.95E7)
Precursor (3+)
Precursor (2+)
Precursor (1+)
Intensity
Retention Time
Smoothing
Show Fragment XIC
Show Heavy
Sequence View
D S E D A E Y L E R
Sequence Coverage: 88.9%
Spectrum Peak Count: 111

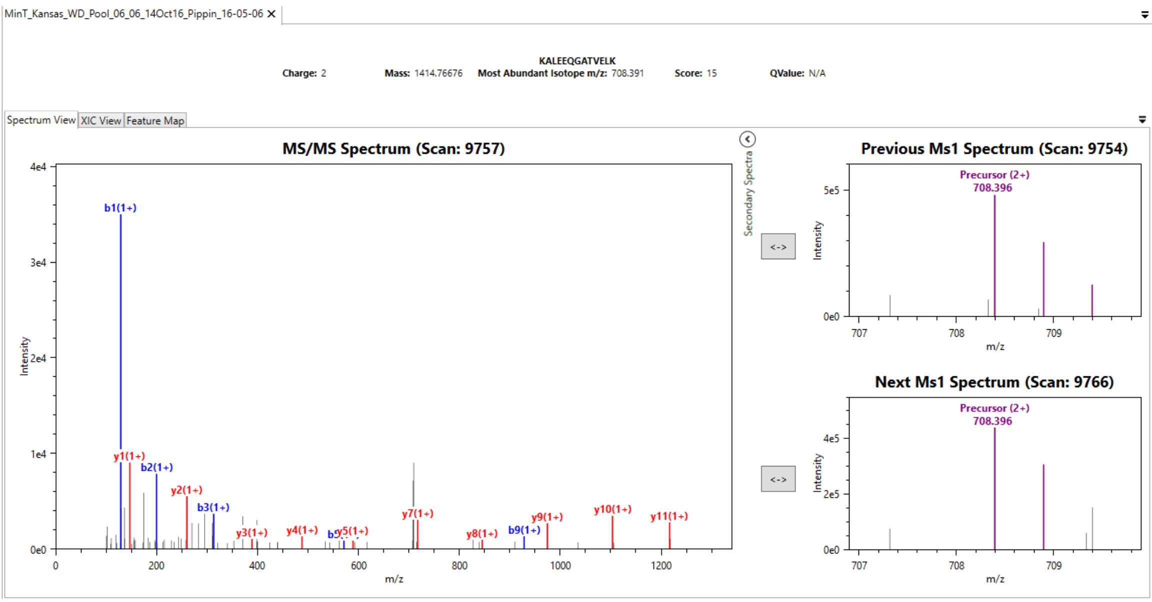
MinT_Kansas_WD_Pool_06_06_14Oct16_Pippin_16-05-06
KALEEQGATVELK
Charge: 2
Mass: 1414.76676
Most Abundant Isotope m/z: 708.391
Score: 15
QValue: N/A
Spectrum View
XIC View
Feature Map
MS/MS Spectrum (Scan: 9757)
Intensity
m/z
b1(1+)
y1(1+)
b2(1+)
y2(1+)
b3(1+)
y3(1+)
y4(1+)
y5(1+)
y7(1+)
y8(1+)
b9(1+)
y9(1+)
y10(1+)
y11(1+)
Secondary Spectra
<->
Previous Ms1 Spectrum (Scan: 9754)
Precursor (2+)
708.396
Next Ms1 Spectrum (Scan: 9766)
Precursor (2+)
708.396

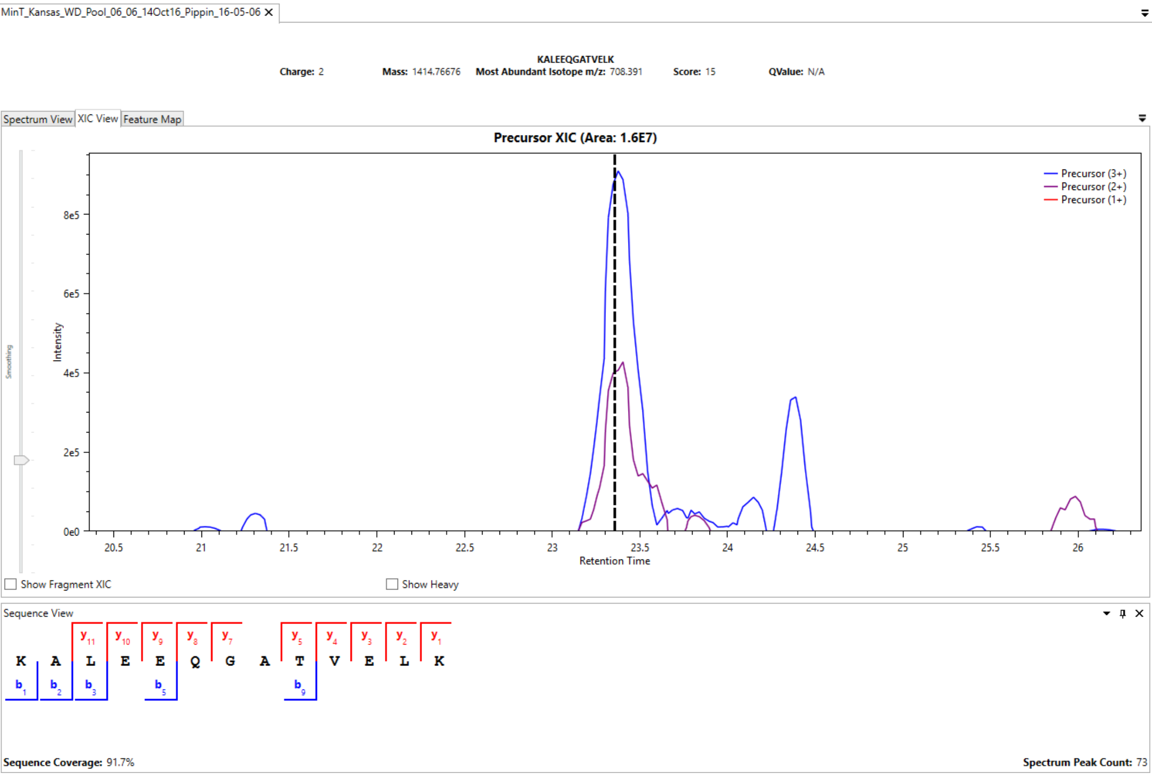
MinT_Kansas_WD_Pool_06_06_14Oct16_Pippin_16-05-06
KALEEQGATVELK
Charge: 2
Mass: 1414.76676
Most Abundant Isotope m/z: 708.391
Score: 15
QValue: N/A
Spectrum View
XIC View
Feature Map
Precursor XIC (Area: 1.6E7)
Intensity
Retention Time
Precursor (3+)
Precursor (2+)
Precursor (1+)
Show Fragment XIC
Show Heavy
Sequence View
K A L E E Q G A T V E L K
Sequence Coverage: 91.7%
Spectrum Peak Count: 73

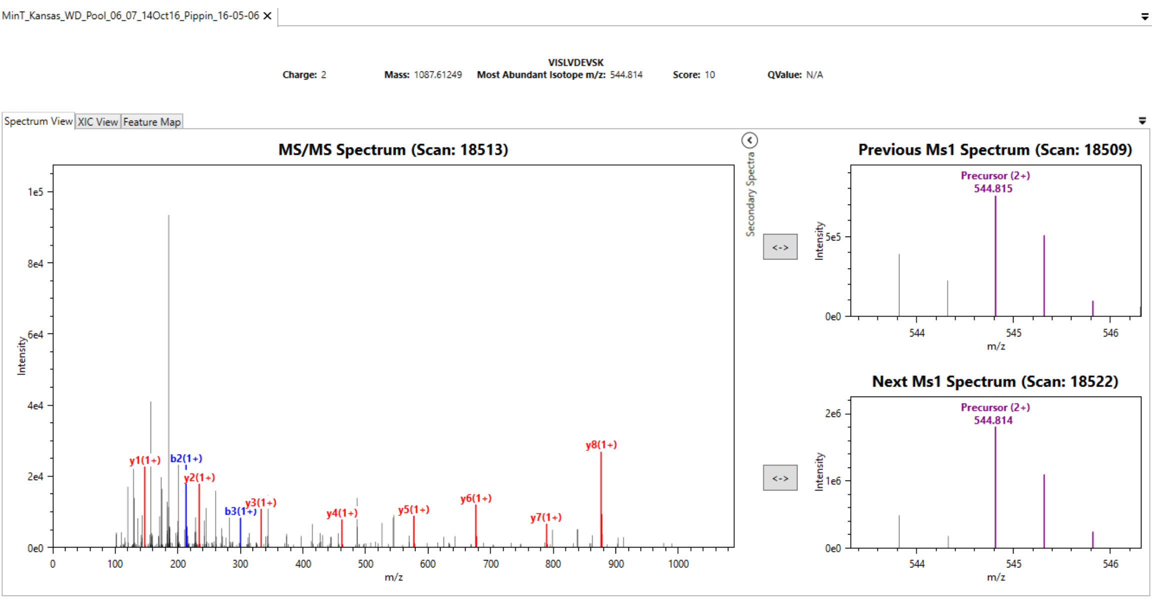
MinT_Kansas_WD_Pool_06_07_14Oct16_Pippin_16-05-06
VISLVDEVSK
Charge: 2
Mass: 1087.61249
Most Abundant Isotope m/z: 544.814
Score: 10
QValue: N/A
Spectrum View
XIC View
Feature Map
MS/MS Spectrum (Scan: 18513)
Intensity
m/z
y1(1+)
b2(1+)
y2(1+)
b3(1+)
y3(1+)
y4(1+)
y5(1+)
y6(1+)
y7(1+)
y8(1+)
Secondary Spectra
Previous Ms1 Spectrum (Scan: 18509)
Precursor (2+)
544.815
Next Ms1 Spectrum (Scan: 18522)
Precursor (2+)
544.814

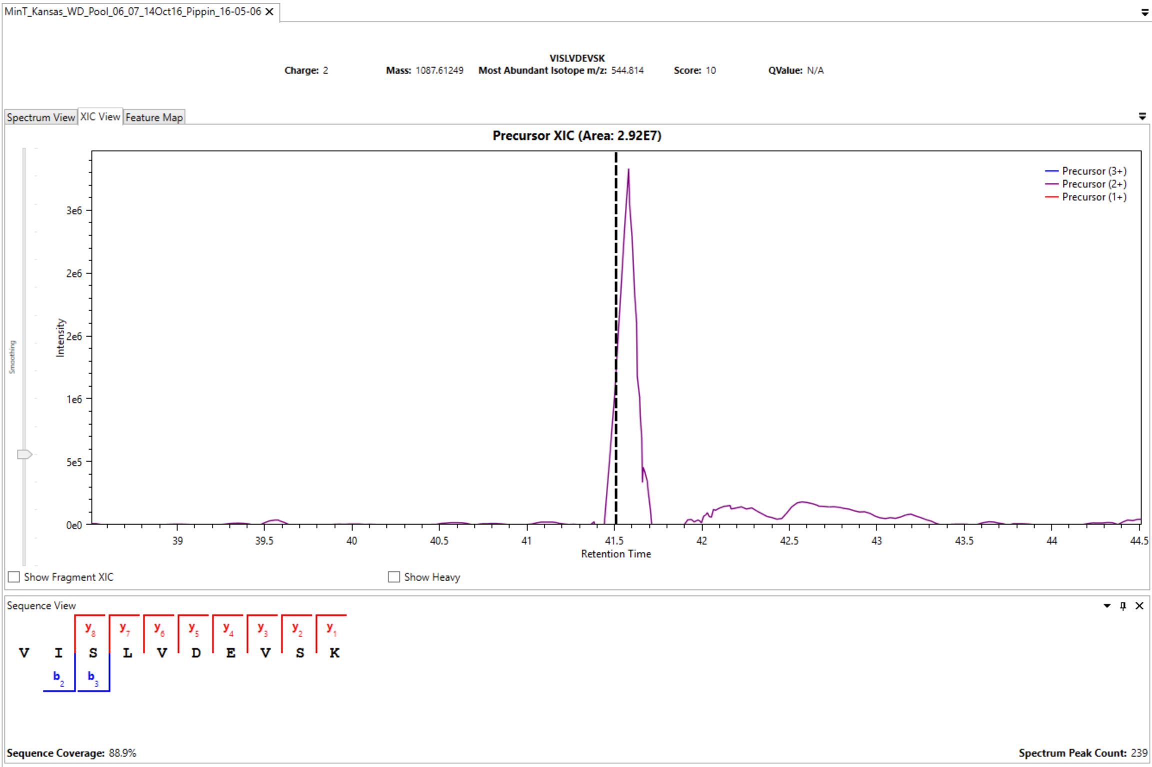
MinT_Kansas_WD_Pool_06_07_14Oct16_Pippin_16-05-06
VISLVDEVSK
Charge: 2
Mass: 1087.61249
Most Abundant Isotope m/z: 544.814
Score: 10
QValue: N/A
Spectrum View
XIC View
Feature Map
Precursor XIC (Area: 2.92E7)
Intensity
Retention Time
Precursor (3+)
Precursor (2+)
Precursor (1+)
Smoothing
Show Fragment XIC
Show Heavy
Sequence View
V I S L V D E V S K
Sequence Coverage: 88.9%
Spectrum Peak Count: 239

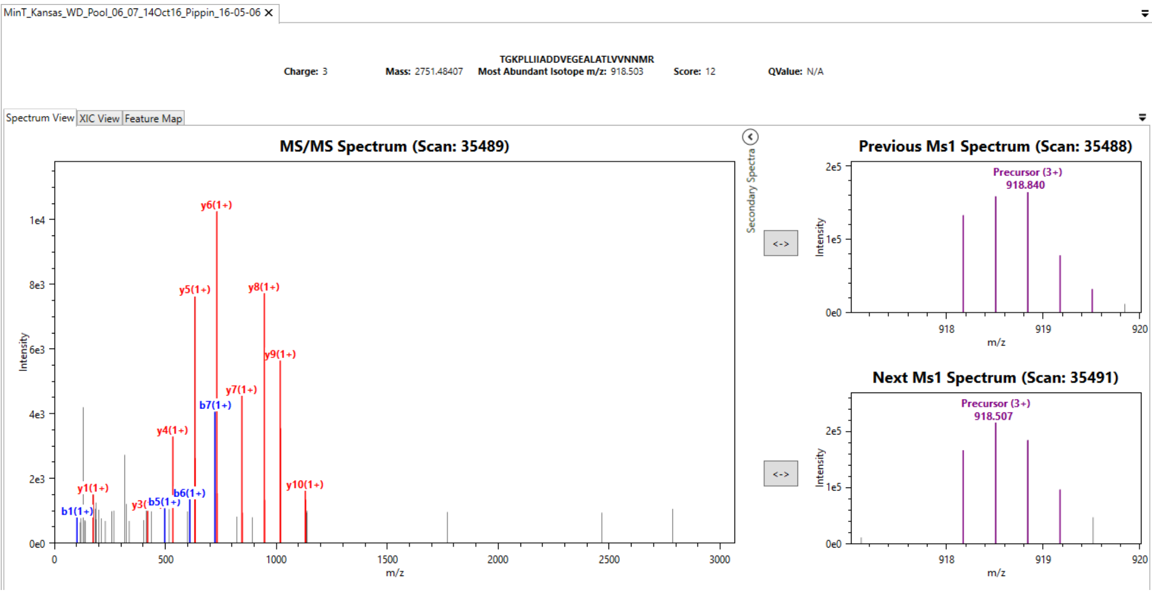
MinT_Kansas_WD_Pool_06_07_14Oct16_Pippin_16-05-06
TGKPLLIIADDVEGEALATLVVNNMR
Charge: 3
Mass: 2751.48407
Most Abundant Isotope m/z: 918.503
Score: 12
QValue: N/A
Spectrum View
XIC View
Feature Map
MS/MS Spectrum (Scan: 35489)
Secondary Spectra
Previous Ms1 Spectrum (Scan: 35488)
Precursor (3+)
918.840
Next Ms1 Spectrum (Scan: 35491)
Precursor (3+)
918.507
Intensity
m/z
y1(1+) y3(y4(1+) y5(1+) y6(1+) y7(1+) y8(1+) y9(1+) y10(1+)
b1(1+) b5(1+) b6(1+) b7(1+)

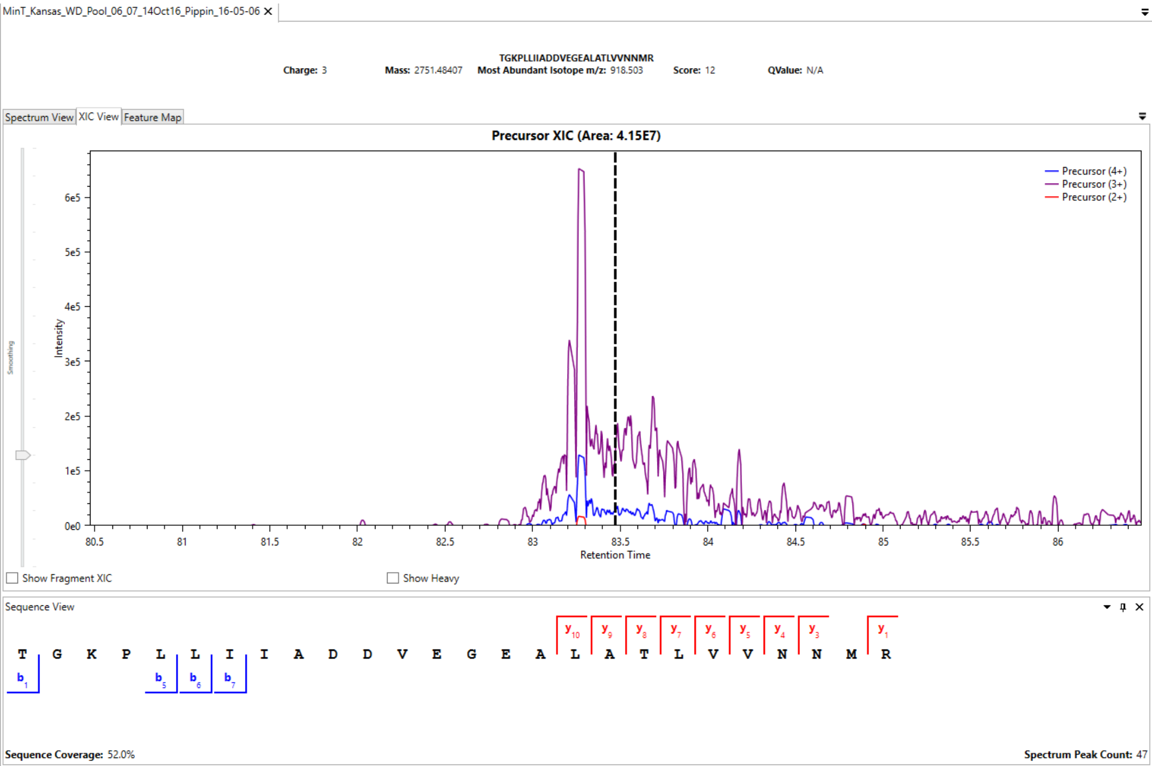
MinT_Kansas_WD_Pool_06_07_14Oct16_Pippin_16-05-06
TGKPLLIIADDVEGEALATLVVNNMR
Charge: 3
Mass: 2751.48407
Most Abundant Isotope m/z: 918.503
Score: 12
QValue: N/A
Spectrum View
XIC View
Feature Map
Precursor XIC (Area: 4.15E7)
Precursor (4+)
Precursor (3+)
Precursor (2+)
Intensity
Retention Time
Smoothing
Show Fragment XIC
Show Heavy
Sequence View
T G K P L L I I A D D V E G E A L A T L V V N N M R
Sequence Coverage: 52.0%
Spectrum Peak Count: 47

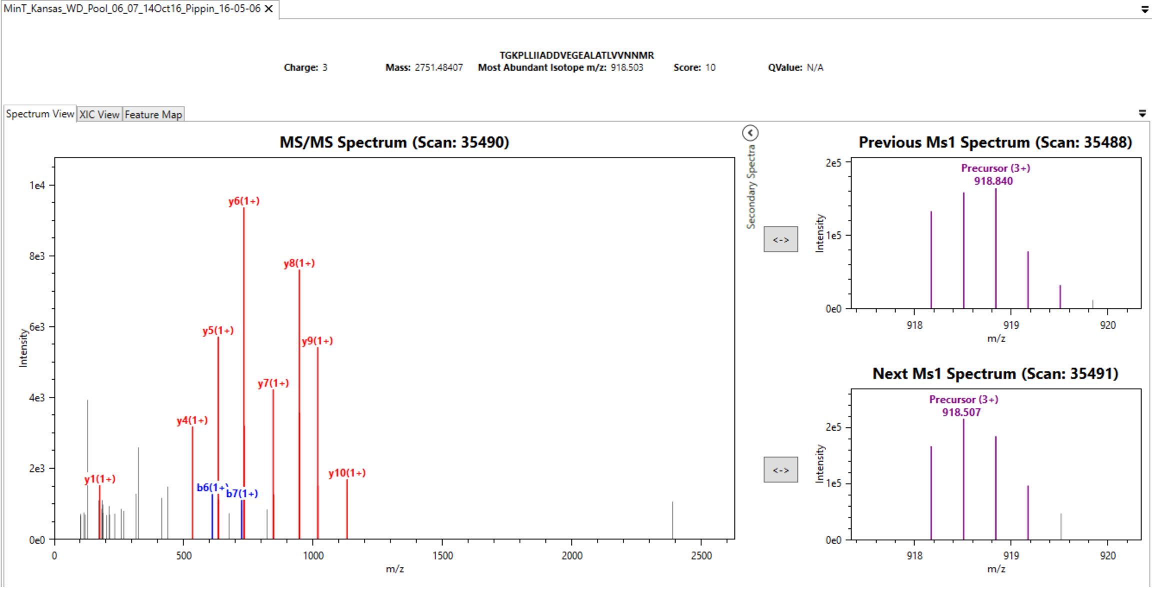
MinT_Kansas_WD_Pool_06_07_14Oct16_Pippin_16-05-06
TGKPLLIIADDVEGEALATLVVNNMR
Charge: 3
Mass: 2751.48407
Most Abundant Isotope m/z: 918.503
Score: 10
QValue: N/A
Spectrum View
XIC View
Feature Map
MS/MS Spectrum (Scan: 35490)
Intensity
m/z
y1(1+)
y4(1+)
y5(1+)
y6(1+)
y7(1+)
y8(1+)
y9(1+)
y10(1+)
b6(1+)
b7(1+)
Secondary Spectra
Previous Ms1 Spectrum (Scan: 35488)
Precursor (3+)
918.840
Next Ms1 Spectrum (Scan: 35491)
Precursor (3+)
918.507

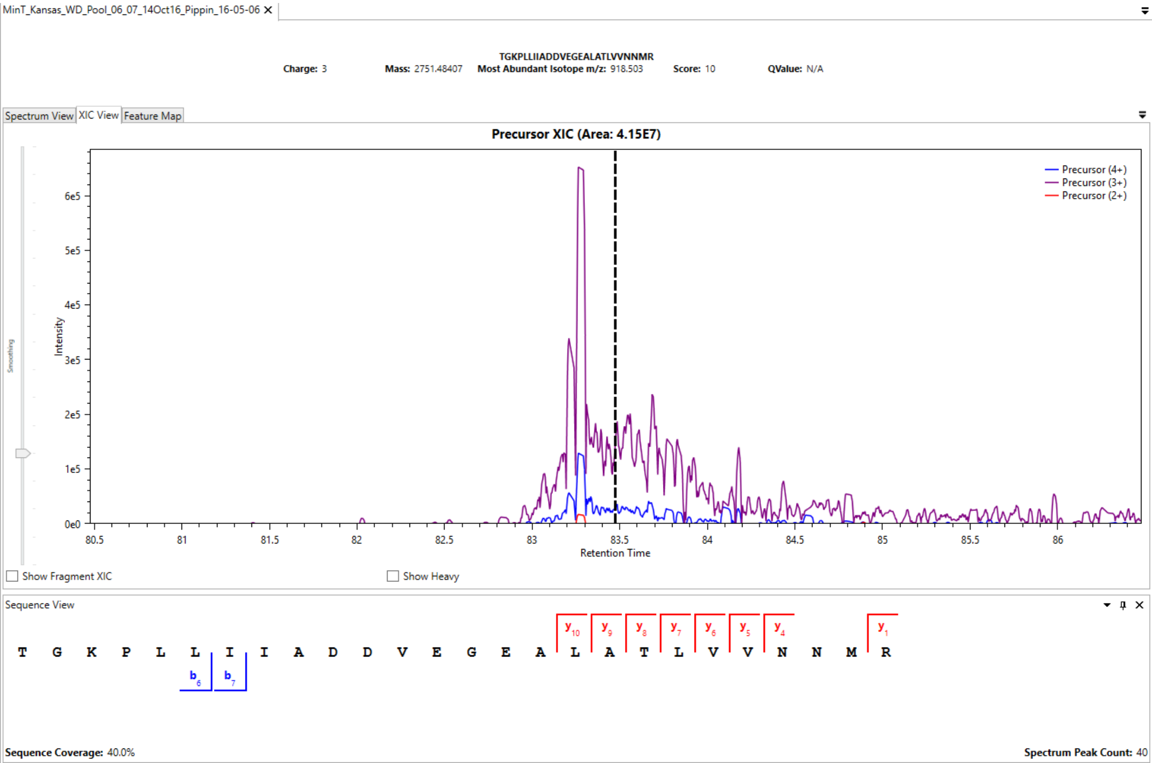
MinT_Kansas_WD_Pool_06_07_14Oct16_Pippin_16-05-06
TGKPLLIIADDVEGEALATLVVNNMR
Charge: 3
Mass: 2751.48407
Most Abundant Isotope m/z: 918.503
Score: 10
QValue: N/A
Spectrum View
XIC View
Feature Map
Precursor XIC (Area: 4.15E7)
Precursor (4+)
Precursor (3+)
Precursor (2+)
Intensity
Retention Time
Smoothing
Show Fragment XIC
Show Heavy
Sequence View
T G K P L L I I A D D V E G E A L A T L V V N N M R
Sequence Coverage: 40.0%
Spectrum Peak Count: 40

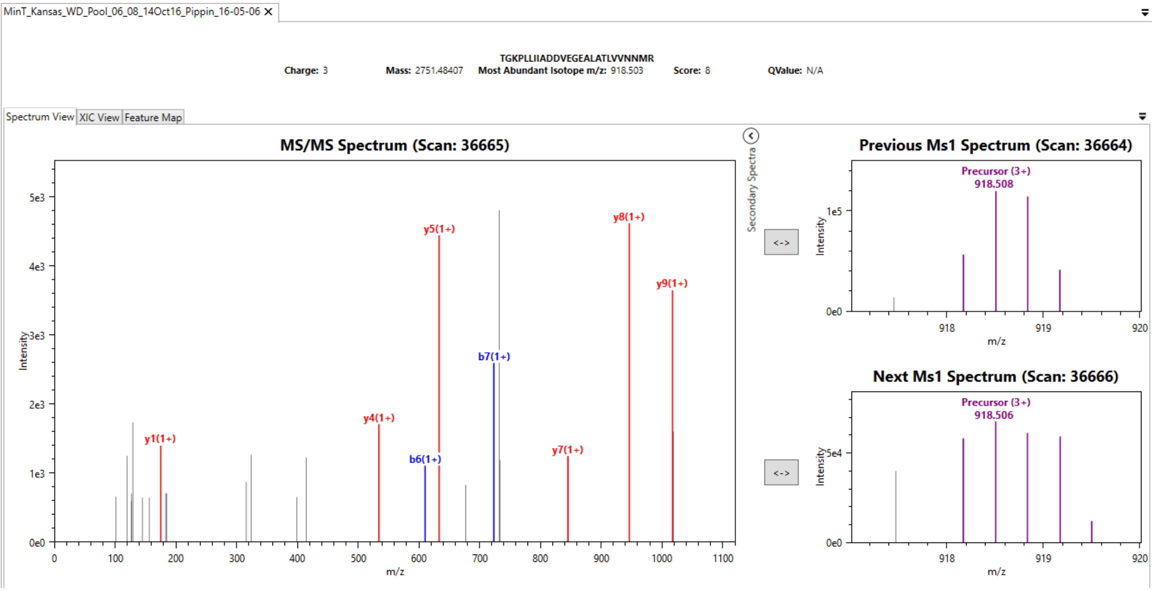
MinT_Kansas_WD_Pool_06_08_14Oct16_Pippin_16-05-06
TGKPLLIIADDVEGEALATLVVNNMR
Charge: 3
Mass: 2751.48407
Most Abundant Isotope m/z: 918.503
Score: 8
QValue: N/A
Spectrum View
XIC View
Feature Map
MS/MS Spectrum (Scan: 36665)
Intensity
m/z
y1(1+)
y4(1+)
b6(1+)
y5(1+)
b7(1+)
y7(1+)
y8(1+)
y9(1+)
Secondary Spectra
Previous Ms1 Spectrum (Scan: 36664)
Precursor (3+)
918.508
Next Ms1 Spectrum (Scan: 36666)
Precursor (3+)
918.506

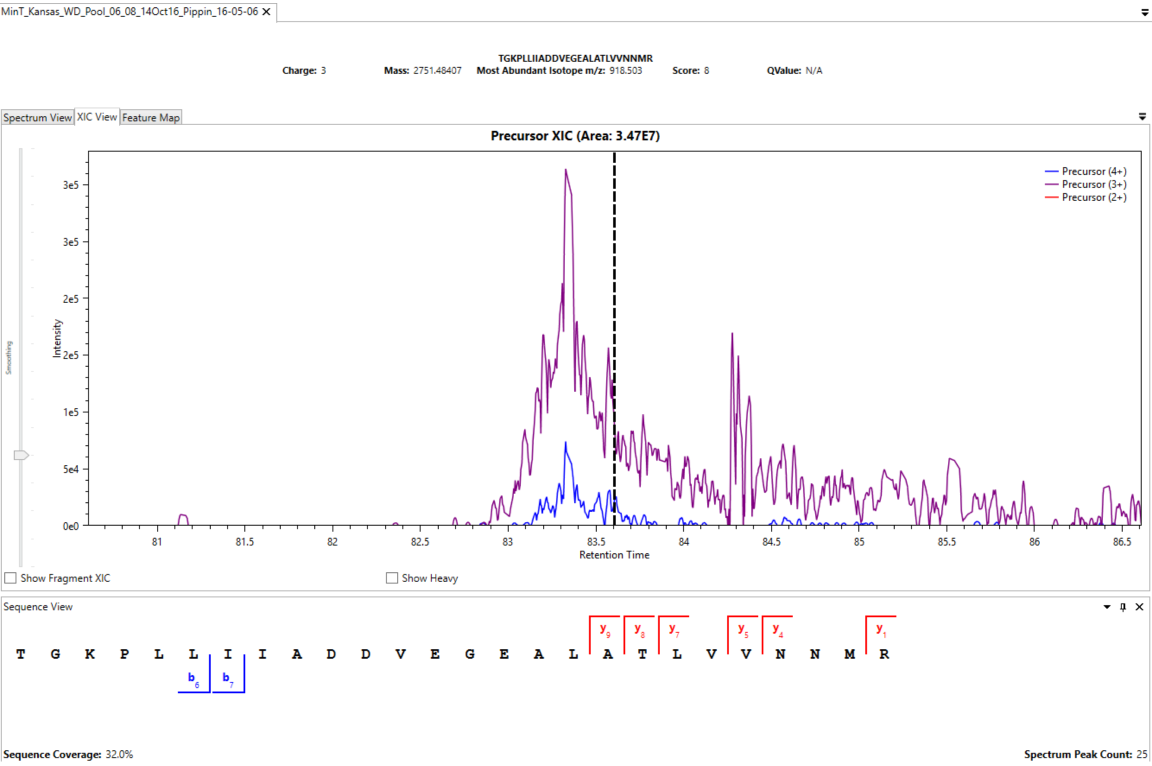
MinT_Kansas_WD_Pool_06_08_14Oct16_Pippin_16-05-06
TGKPLLIIADDVEGEALATLVVNNMR
Charge: 3
Mass: 2751.48407
Most Abundant Isotope m/z: 918.503
Score: 8
QValue: N/A
Spectrum View
XIC View
Feature Map
Precursor XIC (Area: 3.47E7)
Intensity
Retention Time
Precursor (4+)
Precursor (3+)
Precursor (2+)
Smoothing
Show Fragment XIC
Show Heavy
Sequence View
T G K P L L I I A D D V E G E A L A T L V V N N M R
Sequence Coverage: 32.0%
Spectrum Peak Count: 25

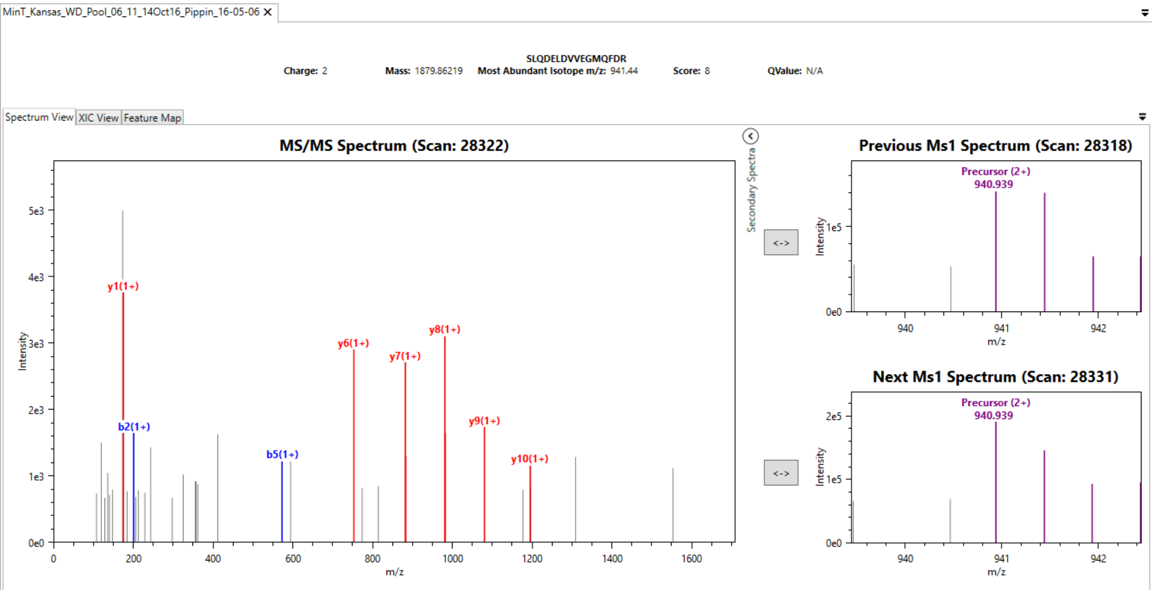
MinT_Kansas_WD_Pool_06_11_14Oct16_Pippin_16-05-06
SLQDELDVVEGMQFDR
Charge: 2
Mass: 1879.86219
Most Abundant Isotope m/z: 941.44
Score: 8
QValue: N/A
Spectrum View
XIC View
Feature Map
MS/MS Spectrum (Scan: 28322)
Intensity
m/z
y1(1+)
b2(1+)
b5(1+)
y6(1+)
y7(1+)
y8(1+)
y9(1+)
y10(1+)
Secondary Spectra
Previous Ms1 Spectrum (Scan: 28318)
Precursor (2+)
940.939
Next Ms1 Spectrum (Scan: 28331)
Precursor (2+)
940.939

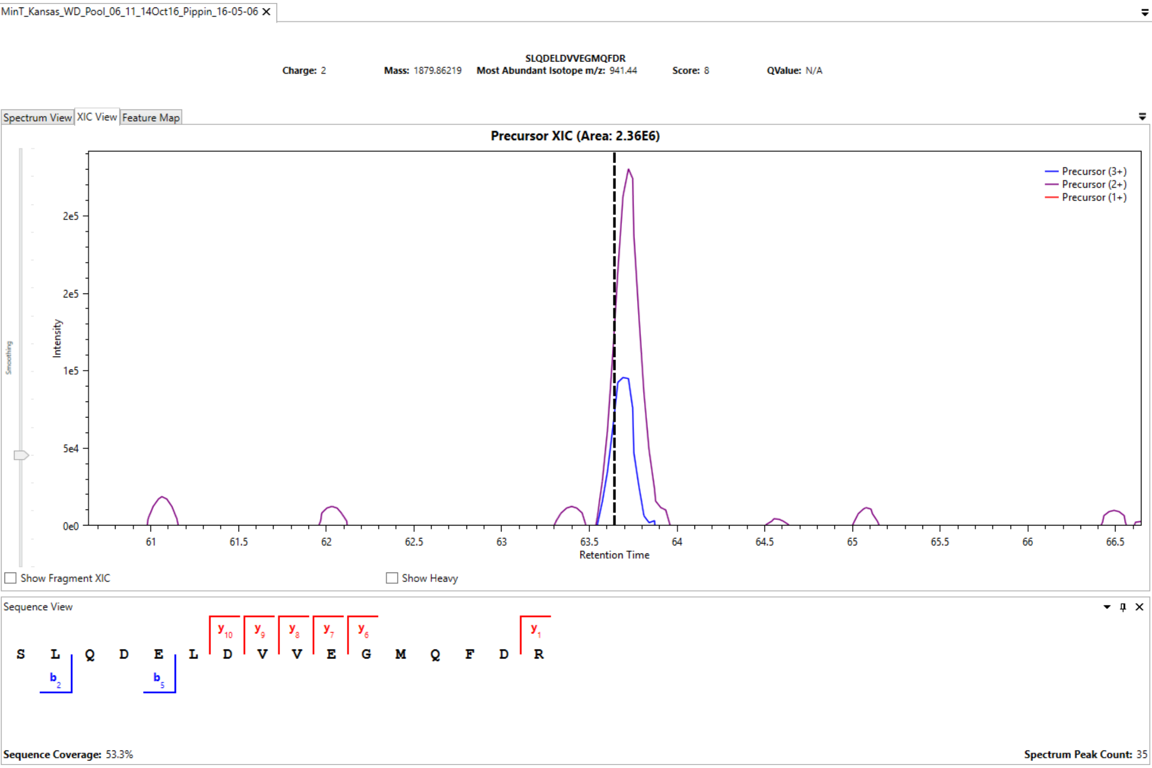
MinT_Kansas_WD_Pool_06_11_14Oct16_Pippin_16-05-06
SLQDELDVVEGMQFDR
Charge: 2
Mass: 1879.86219
Most Abundant Isotope m/z: 941.44
Score: 8
QValue: N/A
Spectrum View
XIC View
Feature Map
Precursor XIC (Area: 2.36E6)
Precursor (3+)
Precursor (2+)
Precursor (1+)
Intensity
Retention Time
Smoothing
Show Fragment XIC
Show Heavy
Sequence View
S L Q D E L D V V E G M Q F D R
Sequence Coverage: 53.3%
Spectrum Peak Count: 35

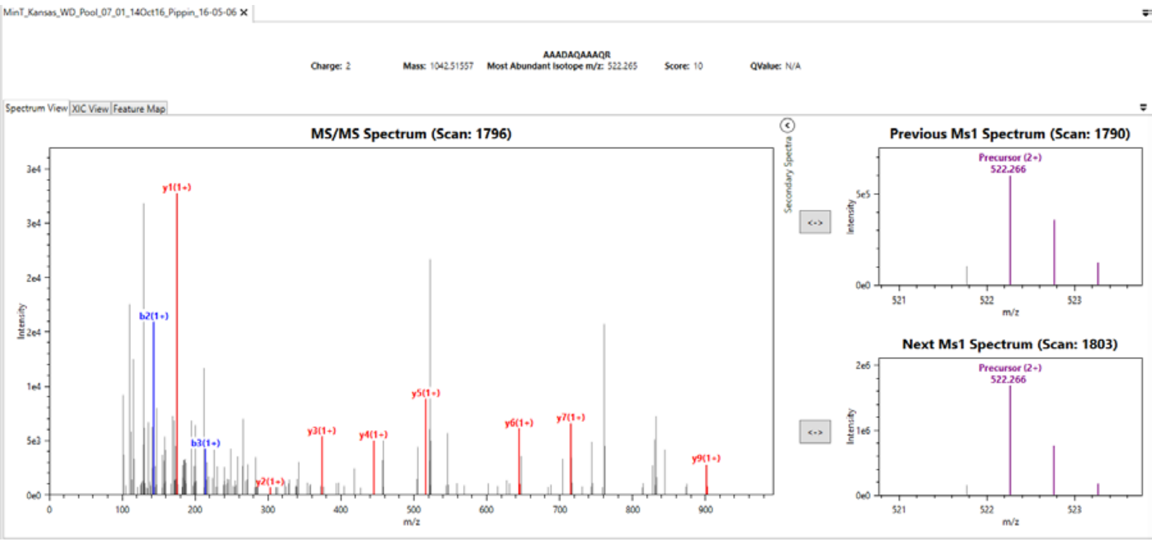
MinT_Kansas_WD_Pool_07_01_14Oct16_Pippin_16-05-06
AAADAQAAAQR
Charge: 2
Mass: 1042.51557
Most Abundant Isotope m/z: 522.265
Score: 10
QValue: N/A
Spectrum View
XIC View
Feature Map
MS/MS Spectrum (Scan: 1796)
y1(1+)
b2(1+)
b3(1+)
y2(1+)
y3(1+)
y4(1+)
y5(1+)
y6(1+)
y7(1+)
y9(1+)
Intensity
m/z
Secondary Spectra
Previous Ms1 Spectrum (Scan: 1790)
Precursor (2+)
522.266
Next Ms1 Spectrum (Scan: 1803)
Precursor (2+)
522.266

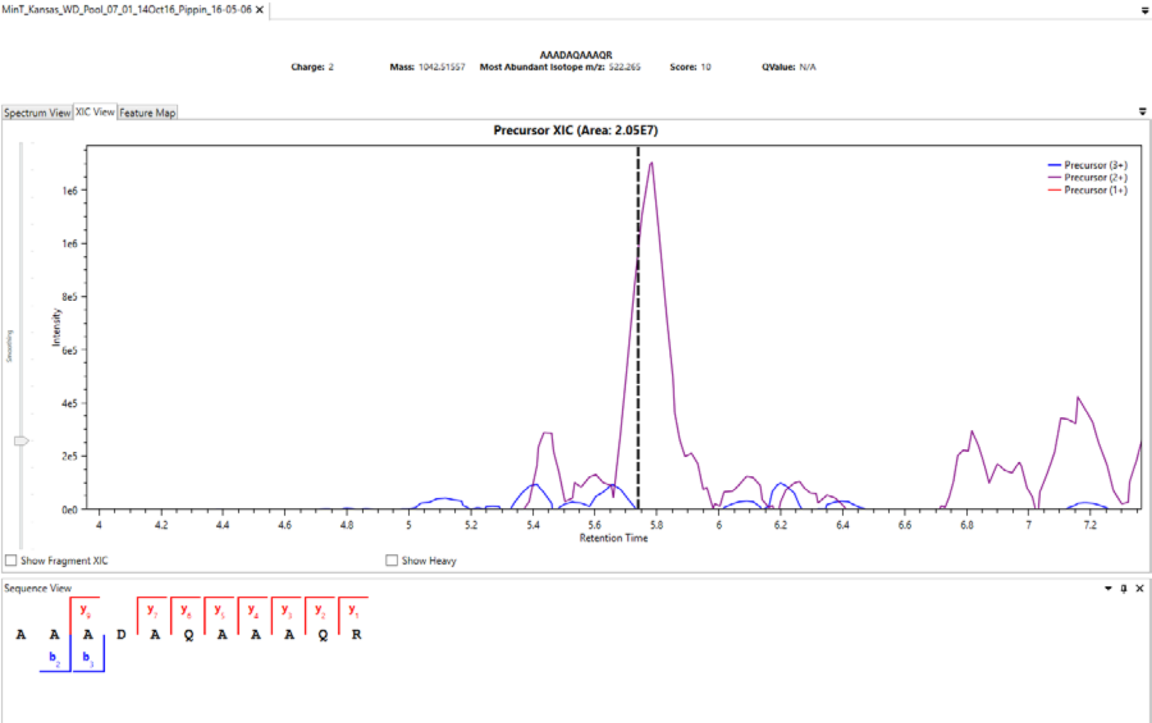
MinT_Kansas_WD_Pool_07_01_14Oct16_Pippin_16-05-06
AAADAQAAAQR
Charge: 2
Mass: 1042.51557
Most Abundant Isotope m/z: 522.265
Score: 10
QValue: N/A
Spectrum View
XIC View
Feature Map
Precursor XIC (Area: 2.05E7)
Precursor (3+)
Precursor (2+)
Precursor (1+)
Intensity
Retention Time
Show Fragment XIC
Show Heavy
Sequence View
A A A D A Q A A A Q R

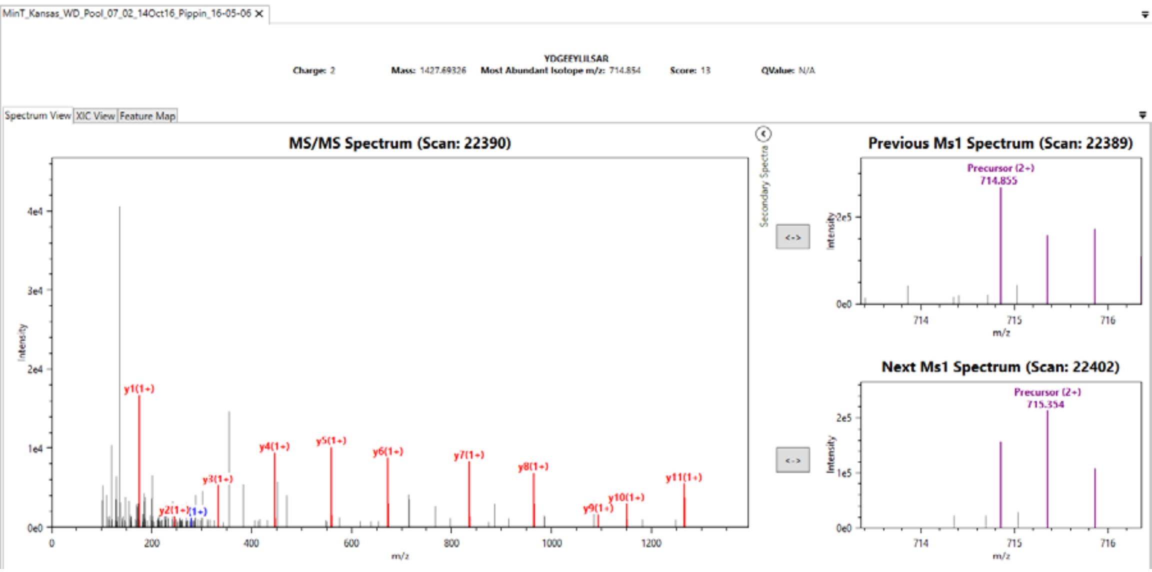
MinT_Kansas_WD_Pool_07_02_14Oct16_Pippin_16-05-06
YDGEEYLILSAR
Charge: 2
Mass: 1427.69326
Most Abundant Isotope m/z: 714.854
Score: 13
QValue: N/A
Spectrum View
XIC View
Feature Map
MS/MS Spectrum (Scan: 22390)
Intensity
m/z
y1(1+)
y2(1+)
y3(1+)
y4(1+)
y5(1+)
y6(1+)
y7(1+)
y8(1+)
y9(1+)
y10(1+)
y11(1+)
Secondary Spectra
Previous Ms1 Spectrum (Scan: 22389)
Precursor (2+)
714.855
Next Ms1 Spectrum (Scan: 22402)
Precursor (2+)
715.354

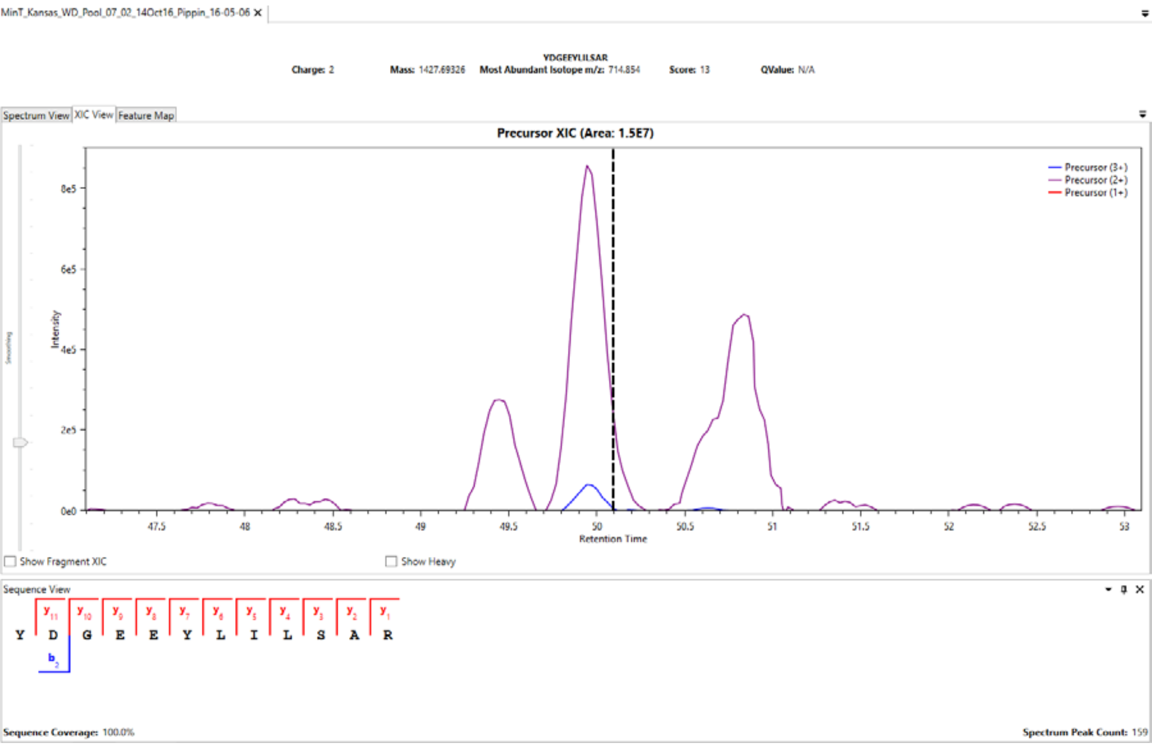
MinT_Kansas_WD_Pool_07_02_14Oct16_Pippin_16-05-06
YDGEEYLILSAR
Charge: 2
Mass: 1427.69326
Most Abundant Isotope m/z: 714.854
Score: 13
QValue: N/A
Spectrum View
XIC View
Feature Map
Precursor XIC (Area: 1.5E7)
Precursor (3+)
Precursor (2+)
Precursor (1+)
Intensity
Retention Time
Show Fragment XIC
Show Heavy
Sequence View
Y D G E E Y L I L S A R
Sequence Coverage: 100.0%
Spectrum Peak Count: 159

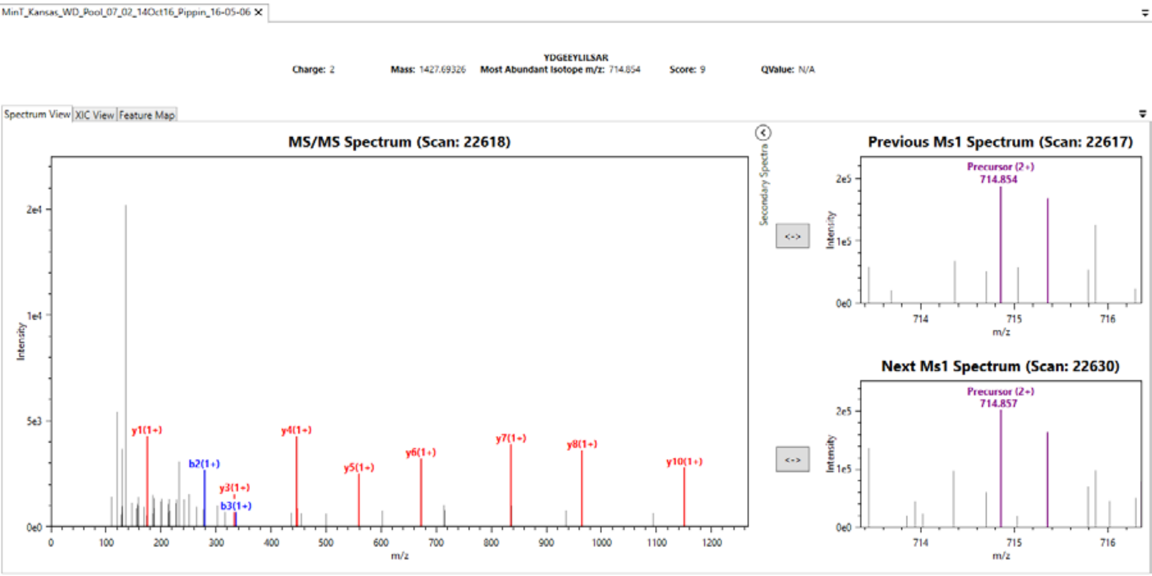
MinT_Kansas_WD_Pool_07_02_14Oct16_Pippin_16-05-06
YDGEEYLILSAR
Charge: 2
Mass: 1427.69326
Most Abundant Isotope m/z: 714.854
Score: 9
QValue: N/A
Spectrum View
XIC View
Feature Map
MS/MS Spectrum (Scan: 22618)
Intensity
m/z
y1(1+)
b2(1+)
y3(1+)
b3(1+)
y4(1+)
y5(1+)
y6(1+)
y7(1+)
y8(1+)
y10(1+)
Secondary Spectra
Previous Ms1 Spectrum (Scan: 22617)
Precursor (2+)
714.854
Next Ms1 Spectrum (Scan: 22630)
Precursor (2+)
714.857

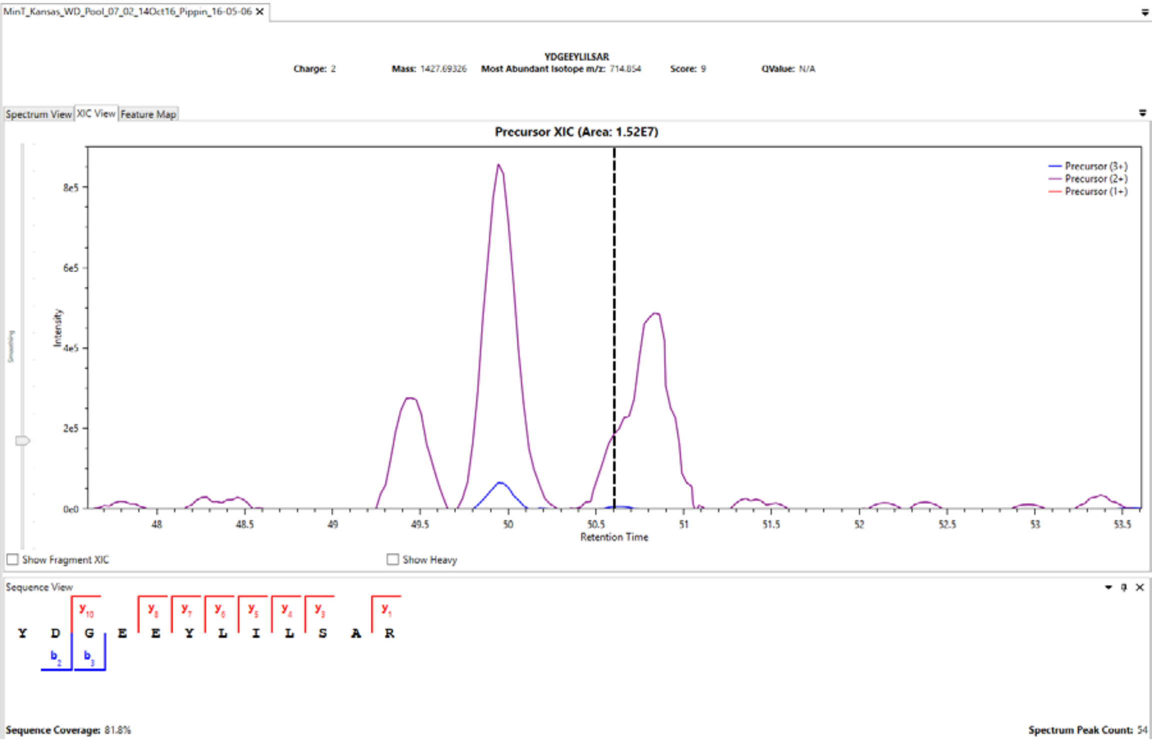
MinT_Kansas_WD_Pool_07_02_14Oct16_Pippin_16-05-06
YDGEEYLILSAR
Charge: 2
Mass: 1427.69326
Most Abundant Isotope m/z: 714.854
Score: 9
QValue: N/A
Spectrum View
XIC View
Feature Map
Precursor XIC (Area: 1.52E7)
Precursor (3+)
Precursor (2+)
Precursor (1+)
Intensity
Retention Time
Show Fragment XIC
Show Heavy
Sequence View
Y D G E E Y L I L S A R
Sequence Coverage: 81.8%
Spectrum Peak Count: 54

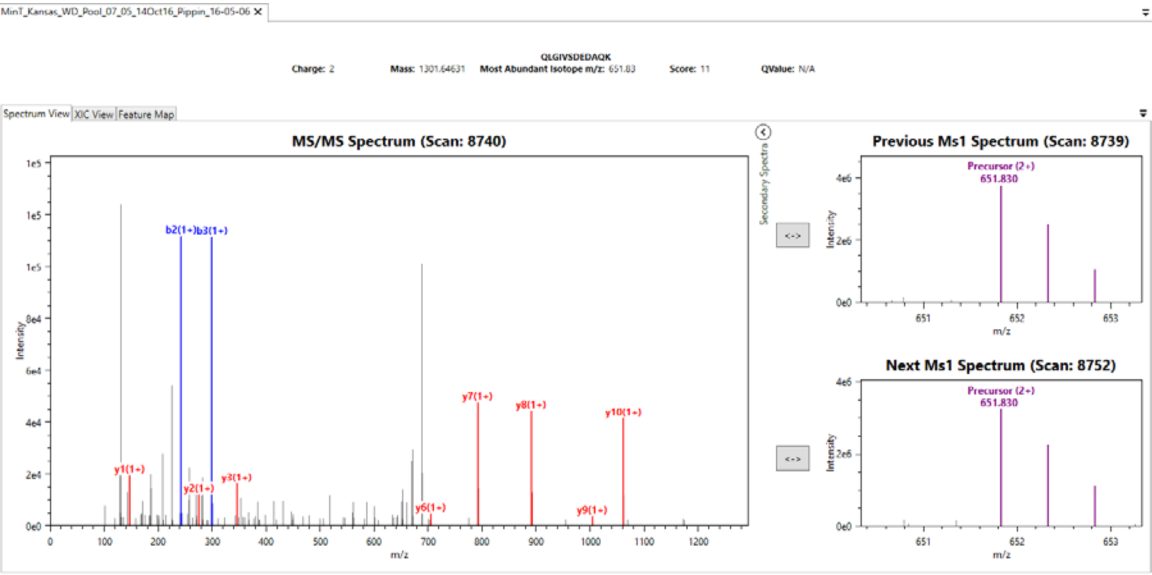
MinT_Kansas_WD_Pool_07_05_14Oct16_Pippin_16-05-06
QLGIVSDEDAQK
Charge: 2
Mass: 1301.64631
Most Abundant Isotope m/z: 651.83
Score: 11
QValue: N/A
Spectrum View
XIC View
Feature Map
MS/MS Spectrum (Scan: 8740)
Intensity
m/z
b2(1+)
b3(1+)
y1(1+)
y2(1+)
y3(1+)
y6(1+)
y7(1+)
y8(1+)
y9(1+)
y10(1+)
Secondary Spectra
Previous Ms1 Spectrum (Scan: 8739)
Next Ms1 Spectrum (Scan: 8752)
Precursor (2+)
651.830

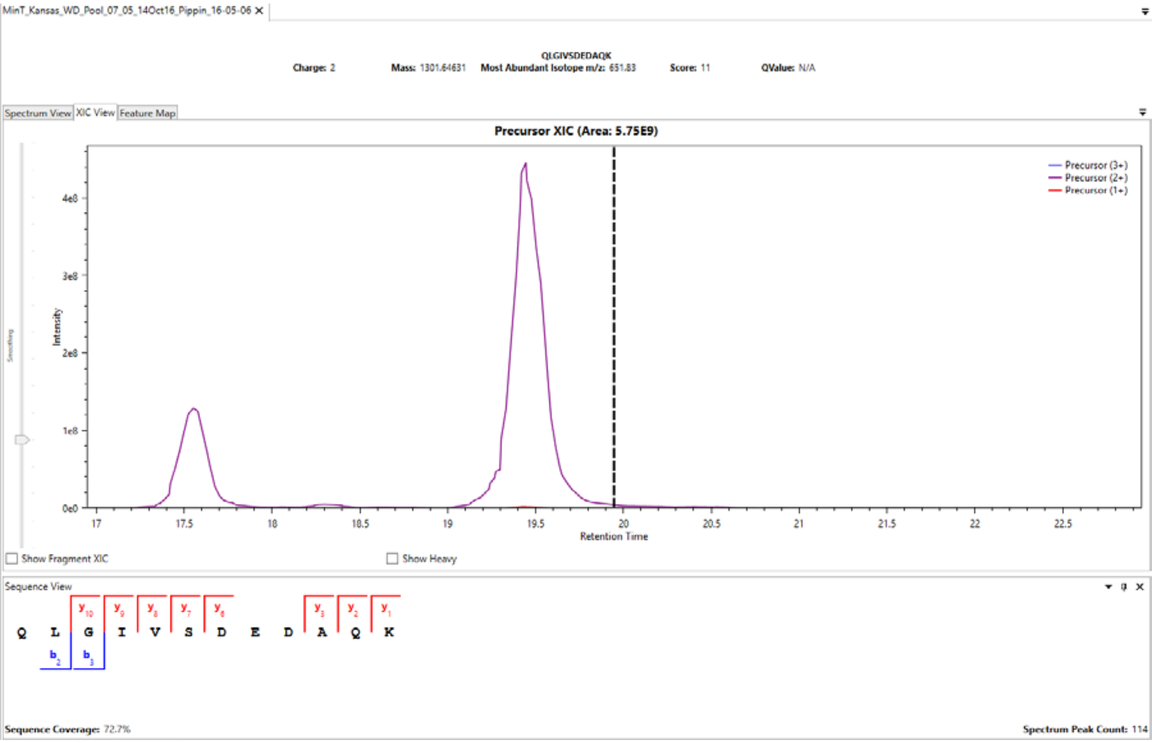
MinT_Kansas_WD_Pool_07_05_14Oct16_Pippin_16-05-06
QLGIVSDEDAQK
Charge: 2
Mass: 1301.64631
Most Abundant Isotope m/z: 651.83
Score: 11
QValue: N/A
Spectrum View
XIC View
Feature Map
Precursor XIC (Area: 5.75E9)
Intensity
Retention Time
Precursor (3+)
Precursor (2+)
Precursor (1+)
Show Fragment XIC
Show Heavy
Sequence View
Q L G I V S D E D A Q K
Sequence Coverage: 72.7%
Spectrum Peak Count: 114

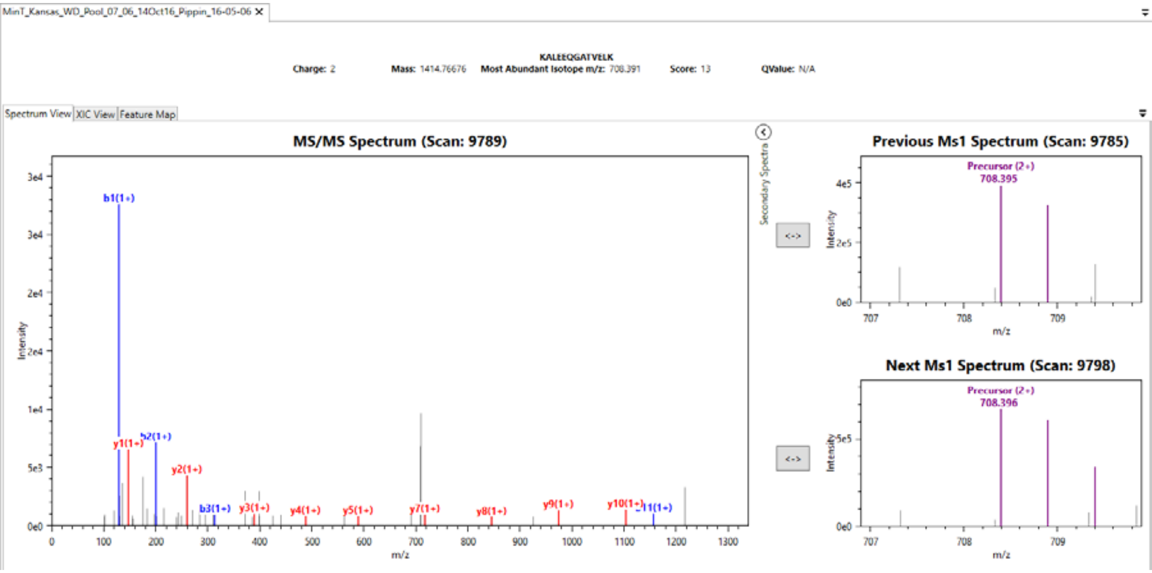
MinT_Kansas_WD_Pool_07_06_14Oct16_Pippin_16-05-06
KALEEQGATVELK
Charge: 2
Mass: 1414.76676
Most Abundant Isotope m/z: 708.391
Score: 13
QValue: N/A
Spectrum View
XIC View
Feature Map
MS/MS Spectrum (Scan: 9789)
Intensity
m/z
b1(1+)
y1(1+)
b2(1+)
y2(1+)
b3(1+)
y3(1+)
y4(1+)
y5(1+)
y7(1+)
y8(1+)
y9(1+)
y10(1+)
b11(1+)
Secondary Spectra
Previous Ms1 Spectrum (Scan: 9785)
Precursor (2+)
708.395
Next Ms1 Spectrum (Scan: 9798)
Precursor (2+)
708.396

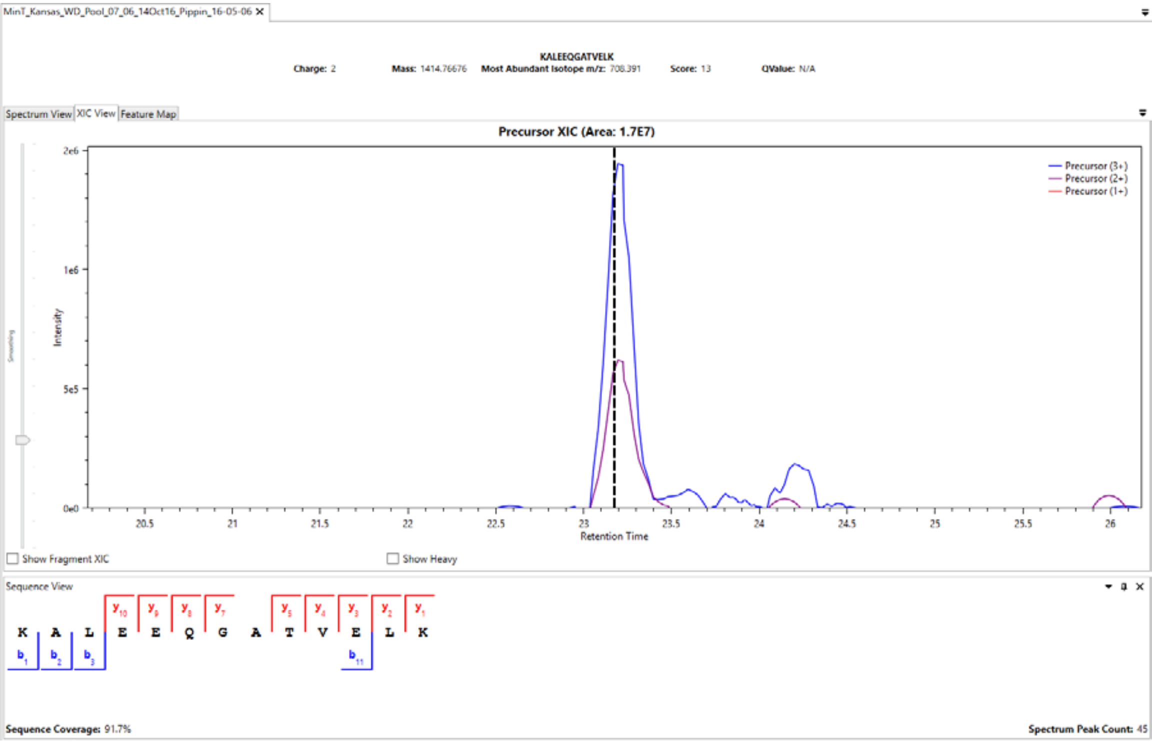
MinT_Kansas_WD_Pool_07_06_14Oct16_Pippin_16-05-06
KALEEQGATVELK
Charge: 2
Mass: 1414.76676
Most Abundant Isotope m/z: 708.391
Score: 13
QValue: N/A
Spectrum View
XIC View
Feature Map
Precursor XIC (Area: 1.7E7)
Intensity
Retention Time
Precursor (3+)
Precursor (2+)
Precursor (1+)
Show Fragment XIC
Show Heavy
Sequence View
K A L E E Q G A T V E L K
Sequence Coverage: 91.7%
Spectrum Peak Count: 45

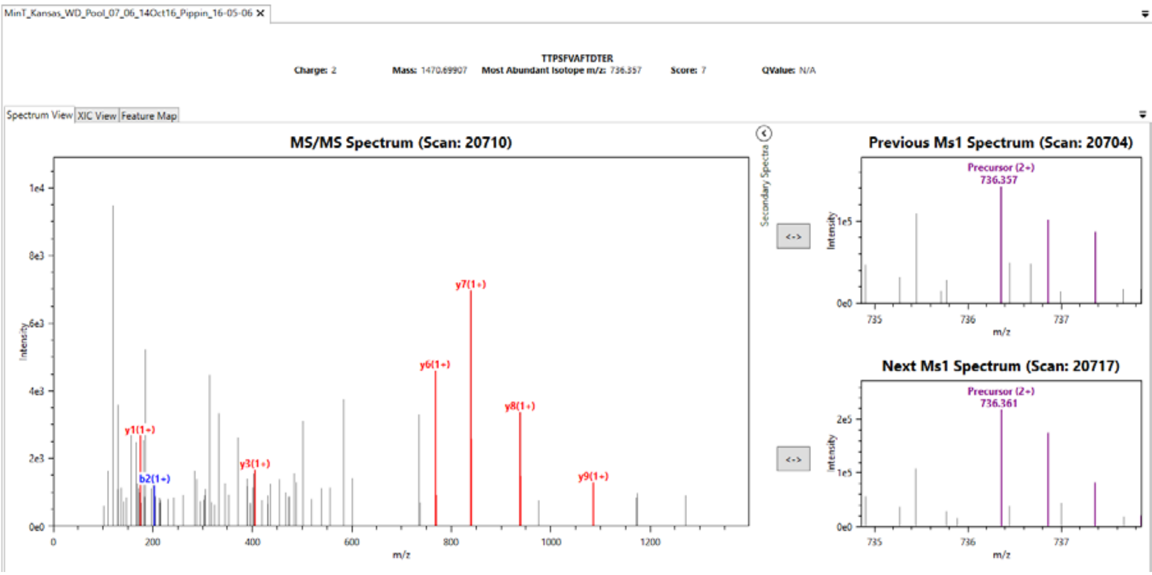
MinT_Kansas_WD_Pool_07_06_14Oct16_Pippin_16-05-06
TTPSFVAFTDTER
Charge: 2
Mass: 1470.69907
Most Abundant Isotope m/z: 736.357
Score: 7
QValue: N/A
Spectrum View
XIC View
Feature Map
MS/MS Spectrum (Scan: 20710)
Intensity
m/z
y1(1+)
b2(1+)
y3(1+)
y6(1+)
y7(1+)
y8(1+)
y9(1+)
Secondary Spectra
Previous Ms1 Spectrum (Scan: 20704)
Precursor (2+)
736.357
Next Ms1 Spectrum (Scan: 20717)
Precursor (2+)
736.361

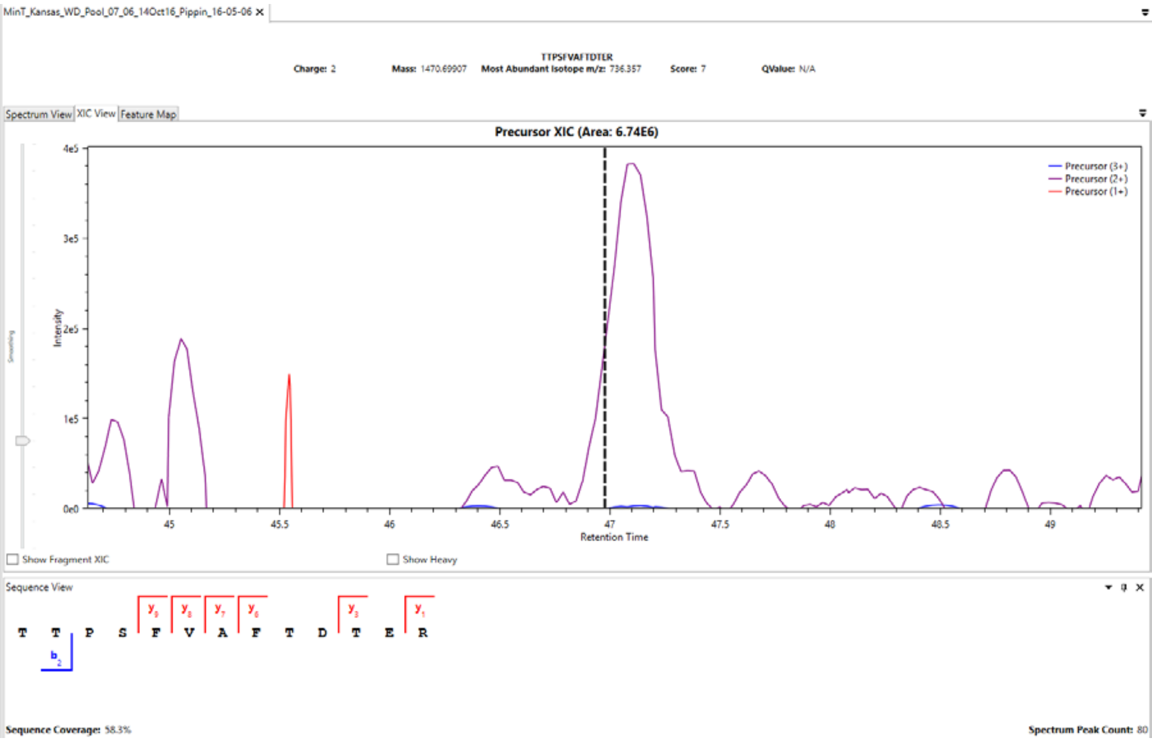
MinT_Kansas_WD_Pool_07_06_14Oct16_Pippin_16-05-06
TTPSFVAFTDTER
Charge: 2
Mass: 1470.69907
Most Abundant Isotope m/z: 736.357
Score: 7
QValue: N/A
Spectrum View
XIC View
Feature Map
Precursor XIC (Area: 6.74E6)
Intensity
Retention Time
Precursor (3+)
Precursor (2+)
Precursor (1+)
Show Fragment XIC
Show Heavy
Sequence View
T T P S F V A F T D T E R
Sequence Coverage: 58.3%
Spectrum Peak Count: 80

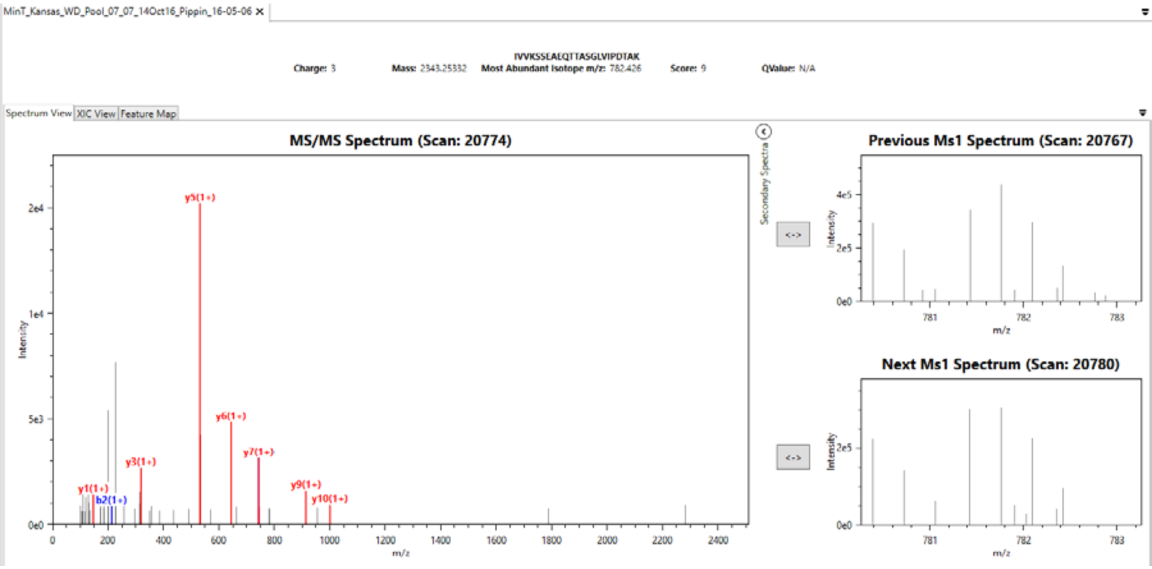
MinT_Kansas_WD_Pool_07_07_14Oct16_Pippin_16-05-06
IVVKSSEAEQTTASGLVIPDTAK
Charge: 3
Mass: 2343.25332
Most Abundant Isotope m/z: 782.426
Score: 9
QValue: N/A
Spectrum View
XIC View
Feature Map
MS/MS Spectrum (Scan: 20774)
Intensity
m/z
y1(1+)
b2(1+)
y3(1+)
y5(1+)
y6(1+)
y7(1+)
y9(1+)
y10(1+)
Secondary Spectra
Previous Ms1 Spectrum (Scan: 20767)
Next Ms1 Spectrum (Scan: 20780)

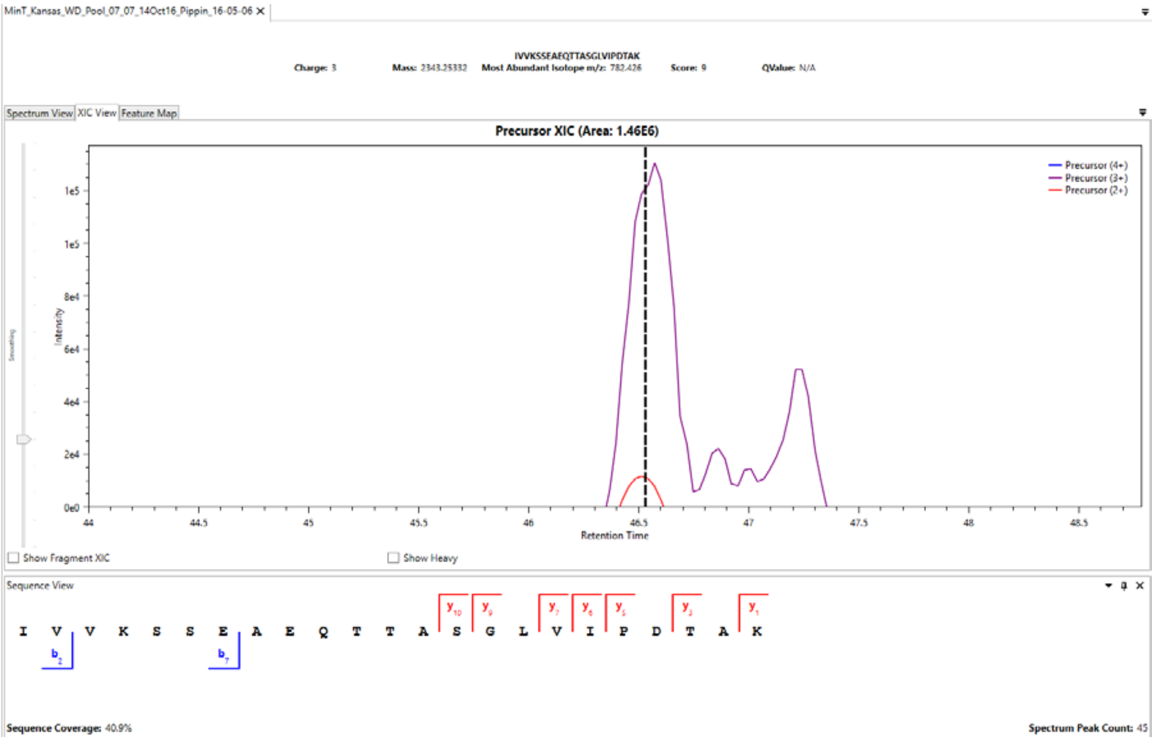
MinT_Kansas_WD_Pool_07_07_14Oct16_Pippin_16-05-06
IVVKSSEAEQTTASGLVIPDTAK
Charge: 3
Mass: 2343.25332
Most Abundant Isotope m/z: 782.426
Score: 9
QValue: N/A
Spectrum View
XIC View
Feature Map
Precursor XIC (Area: 1.46E6)
Precursor (4+)
Precursor (3+)
Precursor (2+)
Intensity
Retention Time
Smoothing
Show Fragment XIC
Show Heavy
Sequence View
I V V K S S E A E Q T T A S G L V I P D T A K
Sequence Coverage: 40.9%
Spectrum Peak Count: 45

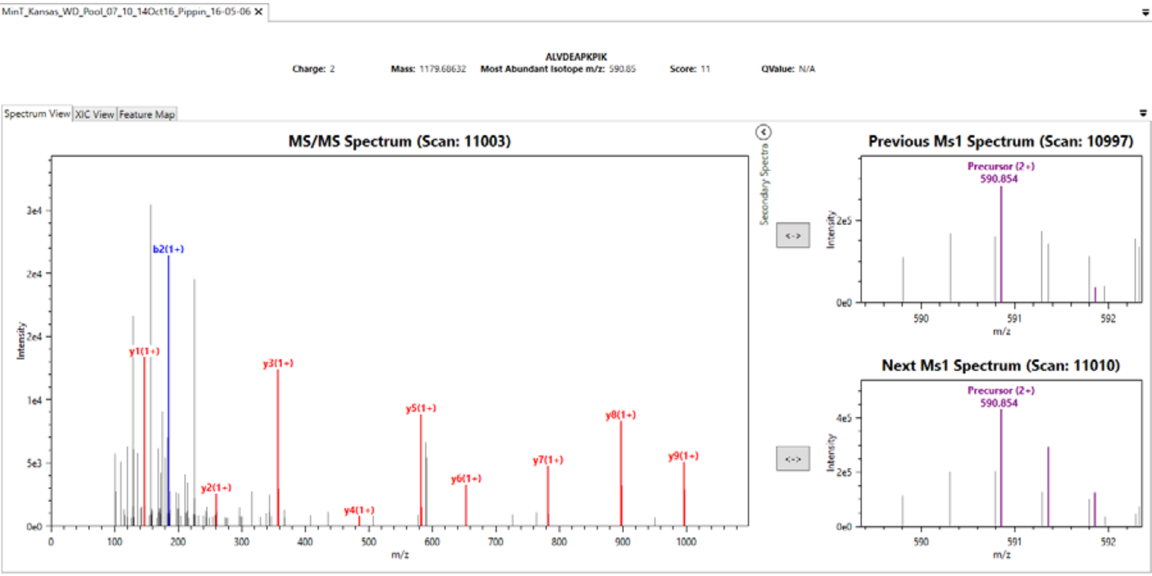
MinT_Kansas_WD_Pool_07_10_14Oct16_Pippin_16-05-06
ALVDEAPKPIK
Charge: 2
Mass: 1179.68632
Most Abundant Isotope m/z: 590.85
Score: 11
QValue: N/A
Spectrum View
XIC View
Feature Map
MS/MS Spectrum (Scan: 11003)
b2(1+)
y1(1+)
y3(1+)
y5(1+)
y8(1+)
y6(1+)
y7(1+)
y9(1+)
y2(1+)
y4(1+)
Intensity
m/z
Secondary Spectra
Previous Ms1 Spectrum (Scan: 10997)
Precursor (2+)
590.854
Next Ms1 Spectrum (Scan: 11010)
Precursor (2+)
590.854

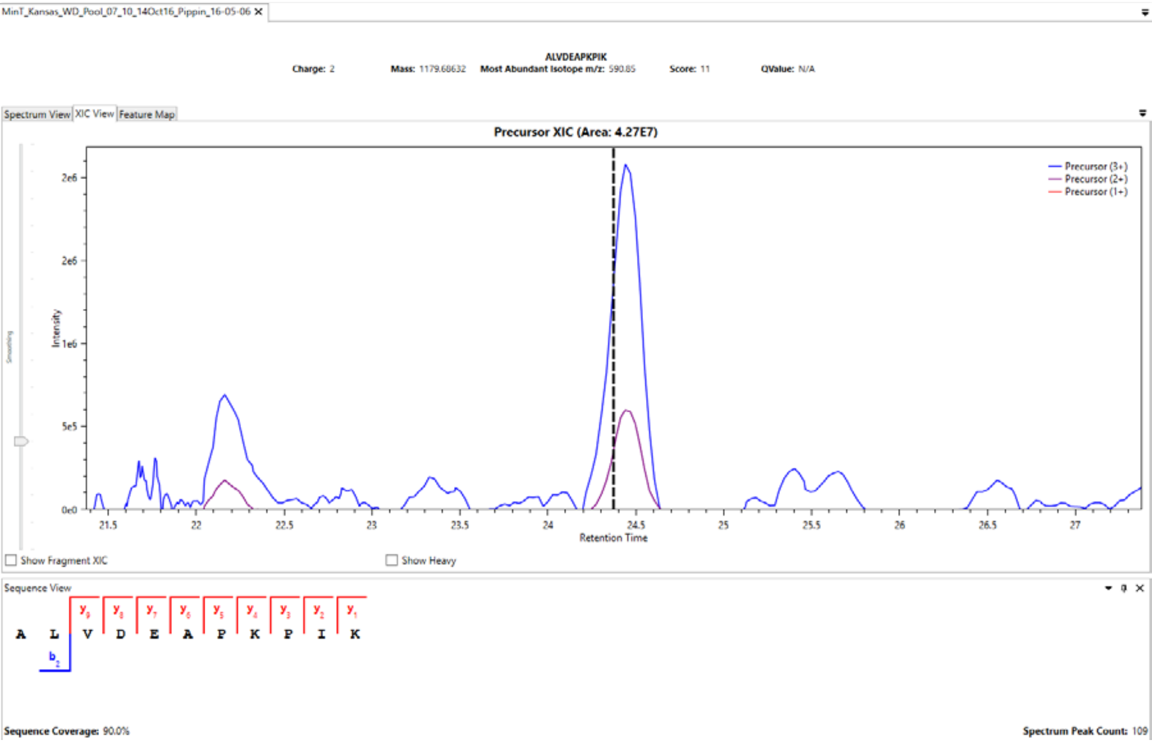
MinT_Kansas_WD_Pool_07_10_14Oct16_Pippin_16-05-06
ALVDEAPKPIK
Charge: 2
Mass: 1179.68632
Most Abundant Isotope m/z: 590.85
Score: 11
QValue: N/A
Spectrum View
XIC View
Feature Map
Precursor XIC (Area: 4.27E7)
Precursor (3+)
Precursor (2+)
Precursor (1+)
Intensity
Retention Time
Show Fragment XIC
Show Heavy
Sequence View
A L V D E A P K P I K
Sequence Coverage: 90.0%
Spectrum Peak Count: 109

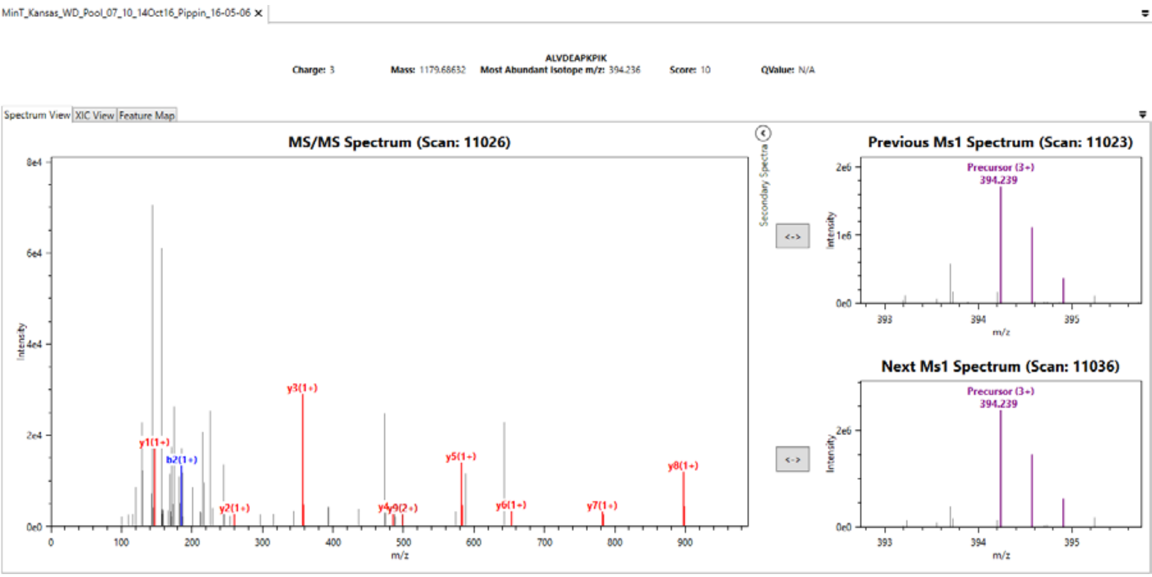
MinT_Kansas_WD_Pool_07_10_14Oct16_Pippin_16-05-06
ALVDEAPKPIK
Charge: 3
Mass: 1179.68632
Most Abundant Isotope m/z: 394.236
Score: 10
QValue: N/A
Spectrum View
XIC View
Feature Map
MS/MS Spectrum (Scan: 11026)
Intensity
m/z
y1(1+)
b2(1+)
y2(1+)
y3(1+)
y4
y9(2+)
y5(1+)
y6(1+)
y7(1+)
y8(1+)
Secondary Spectra
Previous Ms1 Spectrum (Scan: 11023)
Precursor (3+)
394.239
Next Ms1 Spectrum (Scan: 11036)
Precursor (3+)
394.239

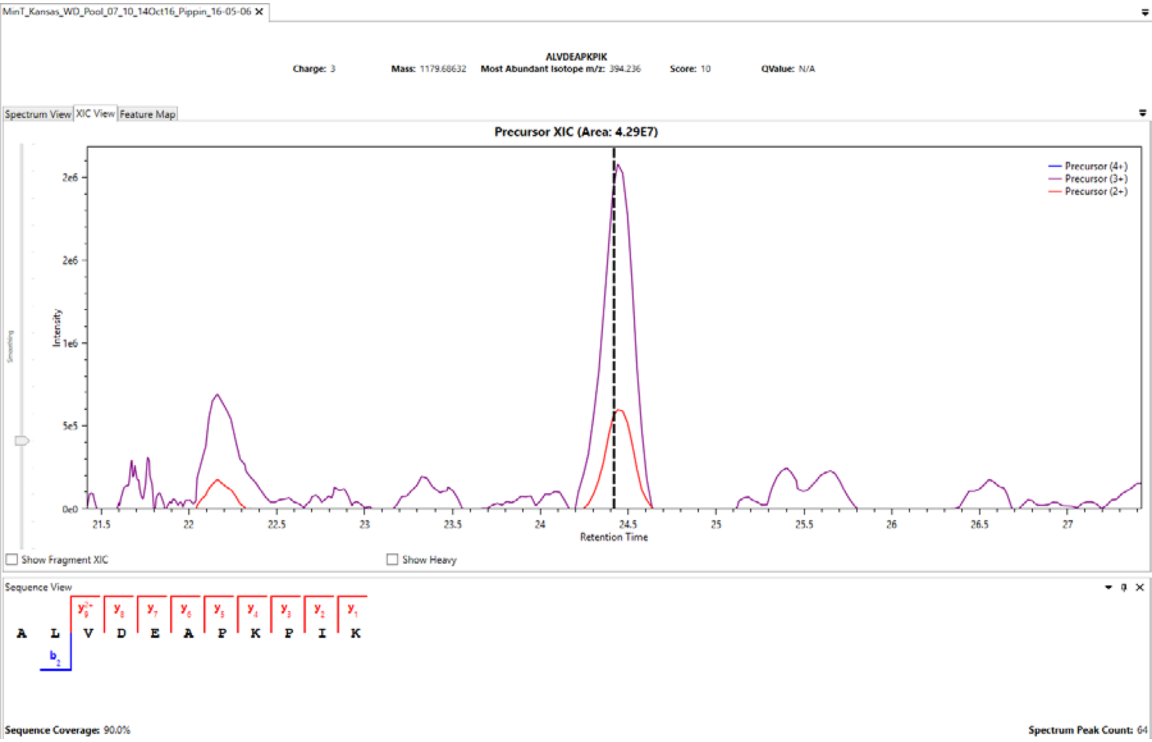
MinT_Kansas_WD_Pool_07_10_14Oct16_Pippin_16-05-06
ALVDEAPKPIK
Charge: 3
Mass: 1179.68632
Most Abundant Isotope m/z: 394.236
Score: 10
QValue: N/A
Spectrum View
XIC View
Feature Map
Precursor XIC (Area: 4.29E7)
Precursor (4+)
Precursor (3+)
Precursor (2+)
Intensity
Retention Time
Show Fragment XIC
Show Heavy
Sequence View
A L V D E A P K P I K
Sequence Coverage: 90.0%
Spectrum Peak Count: 64

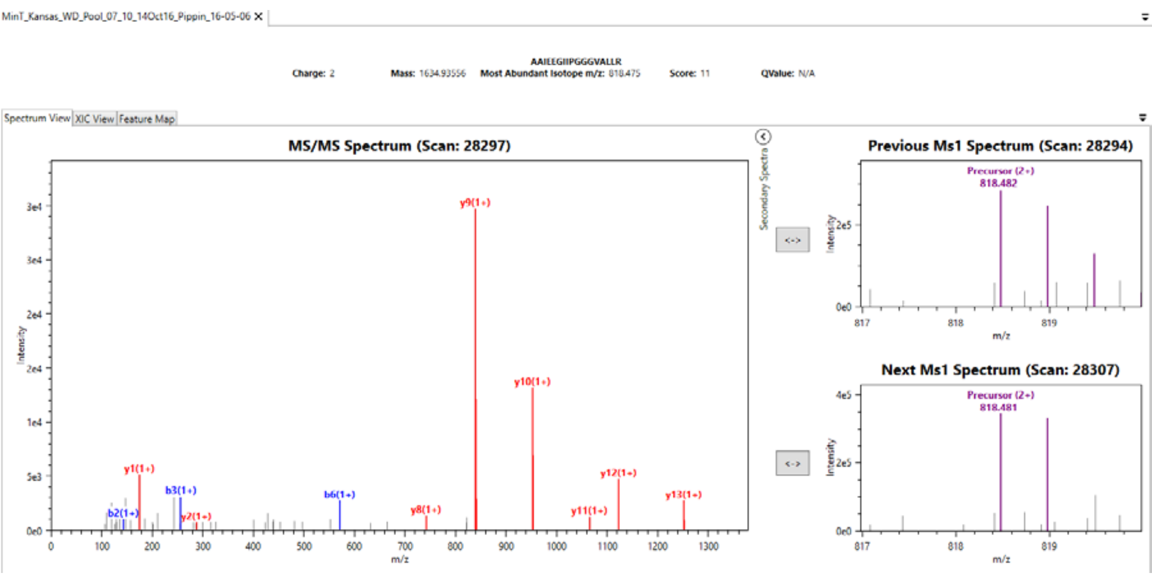
MinT_Kansas_WD_Pool_07_10_14Oct16_Pippin_16-05-06
AAIEEGIIPGGGVALLR
Charge: 2
Mass: 1634.93556
Most Abundant Isotope m/z: 818.475
Score: 11
QValue: N/A
Spectrum View
XIC View
Feature Map
MS/MS Spectrum (Scan: 28297)
Intensity
m/z
y9(1+)
y10(1+)
y1(1+)
b3(1+)
b6(1+)
b2(1+)
y2(1+)
y8(1+)
y11(1+)
y12(1+)
y13(1+)
Secondary Spectra
Previous Ms1 Spectrum (Scan: 28294)
Precursor (2+)
818.482
Next Ms1 Spectrum (Scan: 28307)
Precursor (2+)
818.481

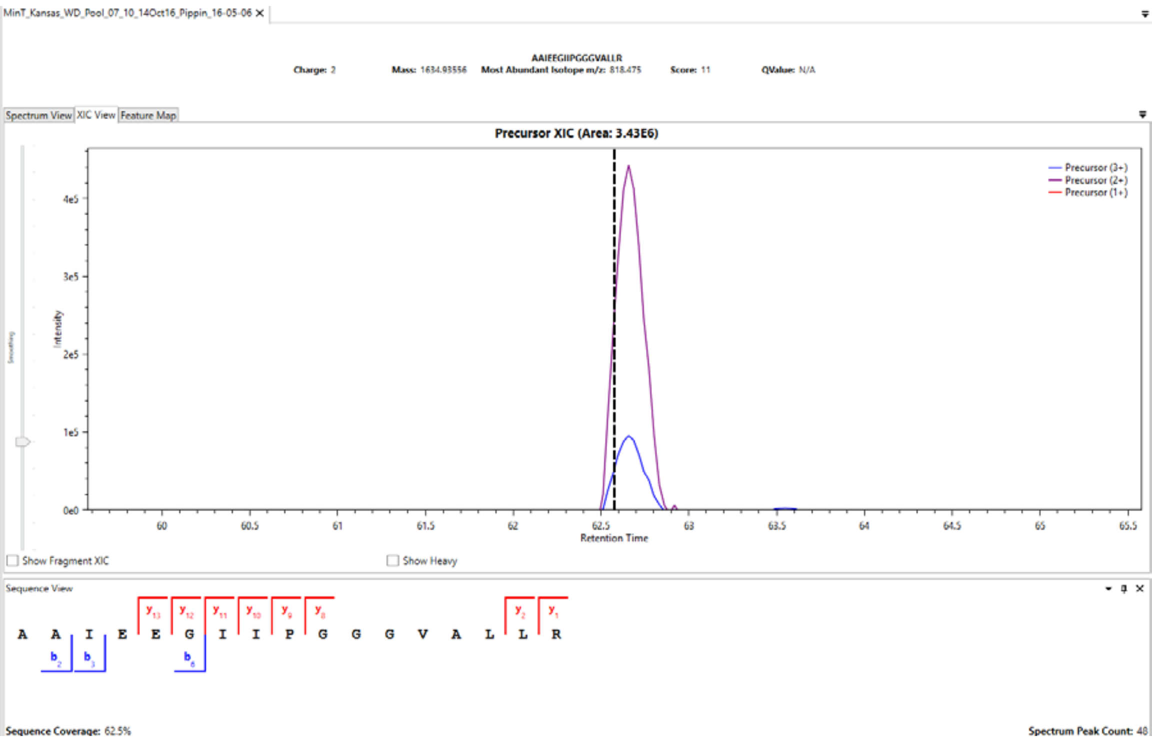
MinT_Kansas_WD_Pool_07_10_14Oct16_Pippin_16-05-06
AAIEEGIIPGGGVALLR
Charge: 2
Mass: 1634.93556
Most Abundant Isotope m/z: 818.475
Score: 11
QValue: N/A
Spectrum View
XIC View
Feature Map
Precursor XIC (Area: 3.43E6)
Intensity
Retention Time
Precursor (3+)
Precursor (2+)
Precursor (1+)
Show Fragment XIC
Show Heavy
Sequence View
A A I E E G I I P G G G V A L L R
Sequence Coverage: 62.5%
Spectrum Peak Count: 48
