# Supplementary figures and images for: Moisture modulates soil reservoirs of active DNA and RNA viruses
Source: Commun Biol. 2021 Aug 26;4:992. doi: 10.1038/s42003-021-02514-2 (PMC8390657; doi:10.1038/s42003-021-02514-2)

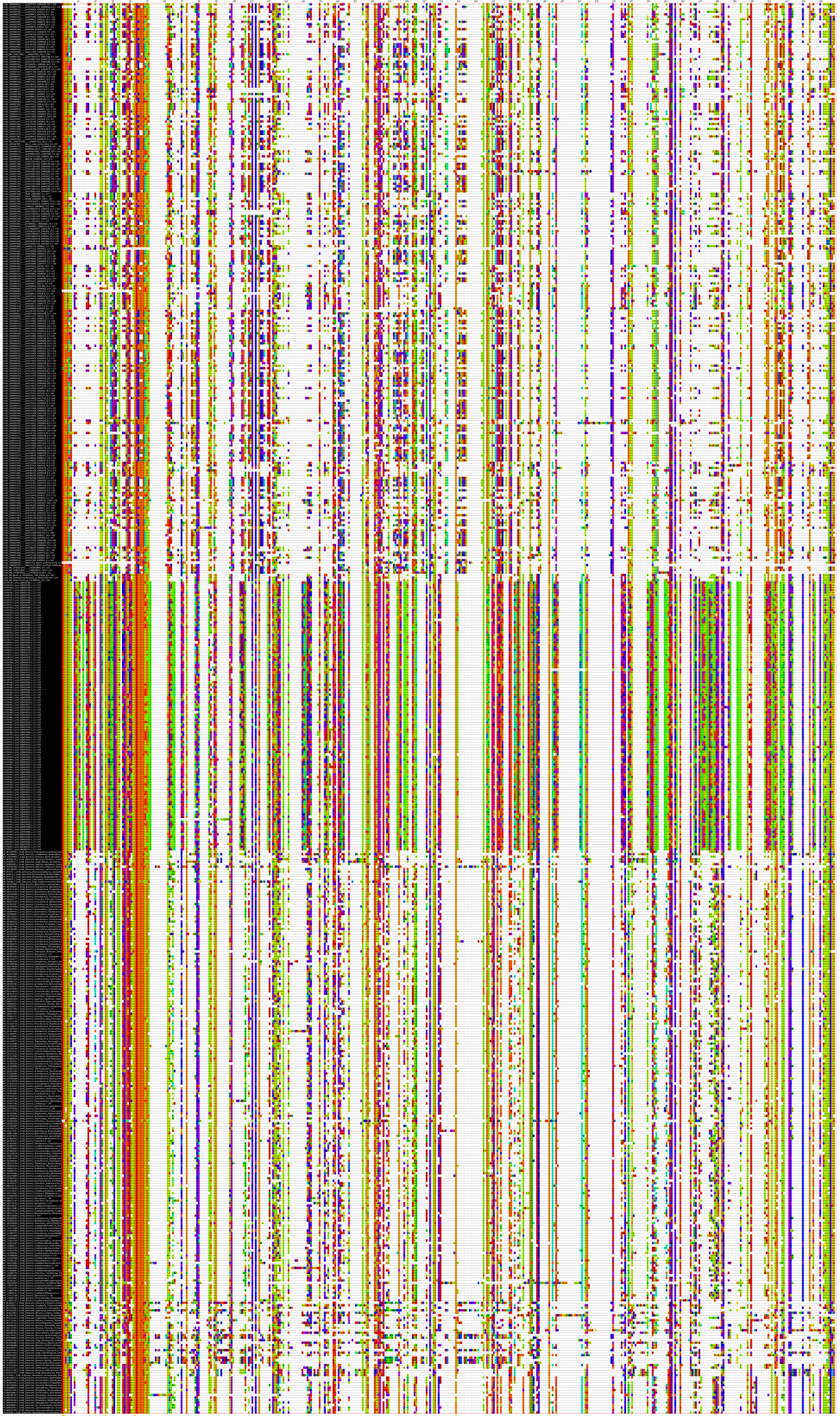

Supplement: Supplementary file 8 — Supplementary Data 6. The alignments of bacterial, eukaryotic, marine and soil viral chaperonin protein sequences. [file 42003_2021_2514_MOESM8_ESM.pdf]
