# Supplementary material for: Moisture modulates soil reservoirs of active DNA and RNA viruses
Source: Commun Biol. 2021 Aug 26;4:992. doi: 10.1038/s42003-021-02514-2 (PMC8390657; doi:10.1038/s42003-021-02514-2)
Supplement: Supplementary file 11 — Reporting summary. [file 42003_2021_2514_MOESM11_ESM.pdf]

nature research

Corresponding author(s): Janet K. Jansson

Last updated by author(s): Jun 11, 2021

# Reporting Summary

Nature Research wishes to improve the reproducibility of the work that we publish. This form provides structure for consistency and transparency in reporting. For further information on Nature Research policies, see our Editorial Policies and the Editorial Policy Checklist.

## Statistics

For all statistical analyses, confirm that the following items are present in the figure legend, table legend, main text, or Methods section.

| n/a | Confirmed | |
|---|---|---|
| ☐ | ☒ | The exact sample size (*n*) for each experimental group/condition, given as a discrete number and unit of measurement |
| ☐ | ☒ | A statement on whether measurements were taken from distinct samples or whether the same sample was measured repeatedly |
| ☐ | ☒ | The statistical test(s) used AND whether they are one- or two-sided<br>*Only common tests should be described solely by name; describe more complex techniques in the Methods section.* |
| ☐ | ☒ | A description of all covariates tested |
| ☐ | ☒ | A description of any assumptions or corrections, such as tests of normality and adjustment for multiple comparisons |
| ☐ | ☒ | A full description of the statistical parameters including central tendency (e.g. means) or other basic estimates (e.g. regression coefficient) AND variation (e.g. standard deviation) or associated estimates of uncertainty (e.g. confidence intervals) |
| ☐ | ☒ | For null hypothesis testing, the test statistic (e.g. *F*, *t*, *r*) with confidence intervals, effect sizes, degrees of freedom and *P* value noted<br>*Give P values as exact values whenever suitable.* |
| ☒ | ☐ | For Bayesian analysis, information on the choice of priors and Markov chain Monte Carlo settings |
| ☐ | ☒ | For hierarchical and complex designs, identification of the appropriate level for tests and full reporting of outcomes |
| ☒ | ☐ | Estimates of effect sizes (e.g. Cohen's *d*, Pearson's *r*), indicating how they were calculated |

*Our web collection on statistics for biologists contains articles on many of the points above.*

## Software and code

Policy information about availability of computer code

| | |
|---|---|
| Data collection | No software was used to collect the data. |
| Data analysis | the listed softwares were used to analyze to data which are all public available: Prodigal (v2.6.3), Hmmer (v3.1b2), EggNOG (Version 3), JGI IMG_VR (2018-07-01_4), metaHipMer (v1.2.1), RefseqVirus (v69), BBDuk (BBmap, v38.73, Burrows-Wheeler Aligner (0.7.17), SAMtools (v1.10), VirSorter (1.0.6), VirFinder (v1.1), vConTACT (v.2.0), pyani (v0.2.9), dbCAN HMMdb (release 7.0), Phyre2 (version 2), functional ontology assignments for metagenomes database (FOAM) (release 2014), SortMeRNA (v2.1b), BamM v1.7.3, samtools v1.9, Trinity (v2.8.5), Contig Annotation Tool (CAT, v4.6), VSEARCH (v2.13.4),MAFFT (v7), Jalview (v2.11.1.0), FastTree (v2.1.10), MS-GF+ search engine. |

For manuscripts utilizing custom algorithms or software that are central to the research but not yet described in published literature, software must be made available to editors and reviewers. We strongly encourage code deposition in a community repository (e.g. GitHub). See the Nature Research guidelines for submitting code & software for further information.

## Data

Policy information about availability of data

All manuscripts must include a data availability statement. This statement should provide the following information, where applicable:

- Accession codes, unique identifiers, or web links for publicly available datasets
- A list of figures that have associated raw data
- A description of any restrictions on data availability

The two published metagenomes can be downloaded from the PNNL Data Hub (https://data.pnnl.gov/project/12620) and White, R. A. et al 15. The metatranscriptome sequence data and the 16S rRNA sequence data are available in Chowdhury et al.13. The MS proteomics data have been deposited to the ProteomeXchange Consortium via the MASSIVE partner repository with the accession number of MSV000086144 (https://massive.ucsd.edu/ProteoSAFe/private-dataset.jsp?task=500afc8e54e1481a9d90fcd35b556511; Username: MSV000086144; password: Soil3653). All of the curated databases, including the reference

viral structural proteins, viral lysogenic gene markers and the curated DNA viral database, source data that were used to plot the figures, Supplementary Data 6, Supplementary Data 7 are available at https://osf.io/3pvm6/files/.

# Field-specific reporting

Please select the one below that is the best fit for your research. If you are not sure, read the appropriate sections before making your selection.

☐ Life sciences ☐ Behavioural & social sciences ☒ Ecological, evolutionary & environmental sciences

For a reference copy of the document with all sections, see nature.com/documents/nr-reporting-summary-flat.pdf

# Ecological, evolutionary & environmental sciences study design

All studies must disclose on these points even when the disclosure is negative.

| | |
|---|---|
| Study description | This study demonstrates that extreme shifts in soil moisture have dramatic impacts on the composition, activity and potential functions of DNA and RNA soil viruses by integrating multi-omics data (metagenomes, metatranscriptomes and metaproteomes). |
| Research sample | We sampled surface soil (top 5-15cm) from the two sites since generally more active microbial processes and viral populations are predicted to reside in surface soil. |
| Sampling strategy | Surface soil samples (top 0-15 cm) were collected from native grassland locations at the Konza Experimental Field Station in Kansas. Soil microcosms were prepared for each site in three replicates and were either air dried (dry treatment) or wet to saturation (wet treatment) and incubated at 21oC for 15 days. |
| Data collection | DNA, RNA and protein were extracted from 0.25 g, 2 g and 10 g, respectively, of soils in each microcosm using PowerSoil DNA and RNA extraction kits according to the manufacturers' instructions and MPLex system. |
| Timing and spatial scale | The soil samples were collected from native grassland field locations in Kansas (39°N 06'12"N and 96°36'50"W) in the Fall of 2017 |
| Data exclusions | No data were excluded. |
| Reproducibility | The step-by-step data analysis procedures were clearly described in the main text which were highly reproducible by using the same parameters. |
| Randomization | not applicable |
| Blinding | The workflow is clearly described step by step complementary with figures |

Did the study involve field work? ☒ Yes ☐ No

## Field work, collection and transport

| | |
|---|---|
| Field conditions | Samples were collected Fall 2017 from the top 5-15 cm of surface soils. No actual field study was performed. The samples are native grasslands and the field conditions were chosen to represent peak growing seasons at the site. The samples were shipped to PNNL for analyses. |
| Location | Kansas grassland (39°N 06'12"N and 96°36'50"W) |
| Access & import/export | A sterile soil corer with 2 cm diameter and 20 cm length was used to collect samples. The collected samples were immediately transferred to PNNL on ice for processing. PNNL has the necessary APHIS permits to receive soils. |
| Disturbance | Upon receipt at PNNL the soil samples were homogenized and aseptically processed through a 4 mm sieve to remove large rocks and plant material, prior to DNA extraction. All sample handling was in compliance with the APHIS permit regulations at PNNL. |

# Reporting for specific materials, systems and methods

We require information from authors about some types of materials, experimental systems and methods used in many studies. Here, indicate whether each material, system or method listed is relevant to your study. If you are not sure if a list item applies to your research, read the appropriate section before selecting a response.

## Materials & experimental systems

| n/a | Involved in the study |
|---|---|
| [x] | [ ] Antibodies |
| [x] | [ ] Eukaryotic cell lines |
| [x] | [ ] Palaeontology and archaeology |
| [x] | [ ] Animals and other organisms |
| [x] | [ ] Human research participants |
| [x] | [ ] Clinical data |
| [x] | [ ] Dual use research of concern |

## Methods

| n/a | Involved in the study |
|---|---|
| [x] | [ ] ChIP-seq |
| [x] | [ ] Flow cytometry |
| [x] | [ ] MRI-based neuroimaging |
